# Computational study of the effect of Lewis base additives and molecular spin state in SmI_2_-chemistry

**DOI:** 10.1039/d5sc08336b

**Published:** 2026-03-10

**Authors:** Song Yu, Ciro Romano, David J. Procter, Nikolas Kaltsoyannis

**Affiliations:** a College of Life Sciences, Huzhou University Huzhou Zhejiang 313000 P. R. China; b Department of Chemistry, School of Natural Sciences, The University of Manchester Manchester M13 9PL UK nikolas.kaltsoyannis@manchester.ac.uk

## Abstract

SmI_2_ has become a crucial reagent in organic chemistry due to its ability to facilitate single-electron transfer (SET) reactions under mild conditions, enabling the construction of complex molecular architectures. Its versatility can be further enhanced by the use of additives, particularly Lewis base (LB) additives. In this study, we provide a computational understanding of the multifaceted role of LB additives in SmI_2_ chemistry. We first identify the critical interplay between the basicity of the LB and the coordination geometry, which together dictate the reducing power of SmI_2_(LB)_*n*_ complexes. For example, the relatively weak LB tetrahydrofuran (THF), a less bulky ligand than analogous ethers such as tetrahydropyran (THP), can achieve tight coordination to Sm, thereby enhancing electron donation and the reducing power of SmI_2_. Additionally, we investigate the SET reduction of various ketones by SmI_2_, showing that both steric and electronic effects from the LB and the substrate play pivotal roles in governing the SET reduction reactivity of SmI_2_, suggesting that SET reactivity cannot be solely determined by the commonly used reduction power of SmI_2_. In the final part of our study, we examine the influence of LB additives on practical SmI_2_-catalysed coupling reactions, finding a revised reaction mechanism that proceeds along a novel septet pathway that outperforms the conventional quintet route. We find that strongly coordinating additives can markedly reshape the reaction profile by accelerating key steps and altering the rate-determining stationary points. These results highlight how LB additives can intricately modulate reactivity and selectivity, offering valuable insights for the rational design of more efficient and selective SmI_2_-mediated transformations.

## Abstract 2

Computational study reveals the role of Lewis bases in SmI_2_ chemistry, from tuning reducing power and modulating single-electron transfer to shaping reaction potential energy surfaces.

## Graphical abstract

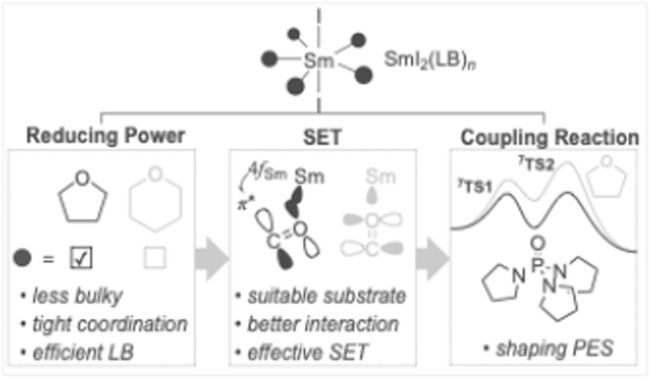

## Introduction

Samarium diiodide (SmI_2_), a versatile single-electron transfer (SET) reductive reagent, has become an indispensable tool in organic chemistry.^1^ Its high selectivity and ability to mediate a range of transformations have made it highly valuable for constructing complex molecular architectures.^2^ Notably, the use of additives has been shown to significantly enhance the performance of SmI_2_ in various reactions.^3^ For example, Lewis base (LB) additives, such as ethers, phosphoramides and other electron-donating ligands, can coordinate to Sm, thereby increasing its reduction power.^2*a*^ This enables SmI_2_ to reduce substrates with higher reduction thresholds. Table 1 illustrates the role of a LB additive, tetrahydrofuran (THF), in a model reaction: the SmI_2_-mediated reduction of acetone. The oxidation of pristine SmI_2_ in THF is energetically demanding, with a computed Gibbs energy of 119.6 kcal mol^−1^. In contrast, the oxidation of SmI_2_ saturated with THF, namely, SmI_2_(thf)_5_, requires 38 kcal mol^−1^ less energy, thereby significantly facilitating the reaction. This large energy decrease can be attributed to the electron-donating ability of the LB, which elevates the energy of the HOMO of the metal centre, thereby enhancing its reducing power. Moreover, the close coordination of THF stabilises the charged metal centre, further decreasing the reaction energy barrier.

**Table 1:** Reactions and associated Gibbs energies, computed with both explicit and implicit (PCM) THF, for the reduction of acetone

Reaction	Δ*G*/kcal mol^−1^
SmI_2_ → SmI_2_^+^ + e^−^	119.6
SmI_2_(thf)_5_ → SmI_2_(thf)_5_^+^ + e^−^	81.6
OC(CH_3_)_2_ + e^−^ → –OC·(CH_3_)_2_	−22.5
SmI_2_(thf)_5_ + OC(CH_3_)_2_ → SmI_2_(thf)_5_^+^ + –OC·(CH_3_)_2_	59.1
SmI_2_(thf)_4_[OC(CH_3_)_2_] → [SmI_2_(thf)_4_^+^][–OC·(CH_3_)_2_]	16.4

To illustrate the role of LB additives, the reduction of acetone was considered. This half-reaction releases 22.5 kcal mol^−1^ (Table 1). Consequently, the single-electron transfer (SET) reduction of acetone by SmI_2_(thf)_5_ requires 59.1 kcal mol^−1^, still thermodynamically unfeasible under standard conditions. Notably, when the SET reaction occurs *via* an inner-sphere mechanism, wherein electron transfer takes place within the SmI_2_(thf)_4_[OC(CH_3_)_2_] complex, the energy requirement is dramatically reduced to only 16.4 kcal mol^−1^. This significant reduction makes the reaction feasible, highlighting the pivotal role of LB additives in SmI_2_-mediated reductions as well as the importance of the inner-sphere mechanism in determining the reaction pathway.

**Scheme 1:**
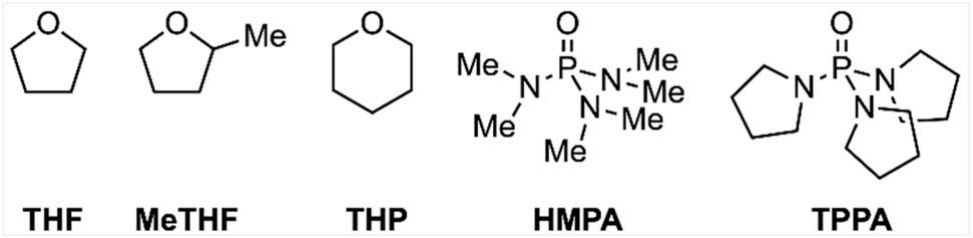
Lewis base additives studied in this work.

Other types of additives are also frequently employed in SmI_2_-mediated reactions. For example, protic additives,^2*a*^ such as water and alcohols, have been shown to expand the functional versatility of SmI_2_ by facilitating proton-coupled electron transfer mechanisms.^4^ Similarly, inorganic additives, including metal ion salts or complexes such as LiCl, FeCl_3_, NiI_2_ and LiBr, are widely utilised in SmI_2_ chemistry.^2*a*^ However, in this study, we focus on a comprehensive exploration of the role of various LB additives used in conjunction with SmI_2_. Not only are LBs one of the most commonly employed additives, but they are likely to have a singular role, in contrast to protic additives, which may also engage in proton transfer and hydrogen bonding with the substrate; thus, LB additives provide a clearer framework for elucidating their specific impact on reactivity. Specifically, we investigated five LB additives: THF, the most frequently used additive,^5^ which also serves as the solvent for most reactions; 2-methyl-THF (MeTHF), a recently reported ligand proven to enhance SmI_2_-catalytic performance;^6^ tetrahydropyran (THP), another cyclic ether with a larger ring size; hexamethylphosphoramide (HMPA), a highly reactive yet toxic additive in SmI_2_ chemistry;^7^ and tripyrrolidinophosphoramide, (TPPA), a potential less toxic alternative to HMPA (Scheme 1).^8^

We also examine model SET reduction reactions and potential energy surfaces (PES) of SmI_2_-catalysed coupling reactions. By revisiting the reaction mechanism of SmI_2_-catalysed intermolecular coupling reactions, we identify a previously overlooked septet pathway that offers a significant kinetic advantage over the conventional quintet mechanism. This mechanistic insight sets the stage to examine how additives modulate not only the initial electron transfer step but the full PES in these important transformations. Through a systematic analysis of the roles of LB additives, we offer valuable guidance for the future rational design of SmI_2_-mediated reactions.

## Computational details

Geometry optimisations were performed by using Gaussian 16, Revision C.01,^9^ employing the PBE0 functional^10^ and Dunning's Correlation-Consistent Double-Zeta + polarisation (cc-pVDZ)^11^ basis sets for C, H and O. For Sm and I, Stuttgart-Köln Effective Core Potentials (ECPs) and associated valence basis sets were used.^12^ The dispersion corrections from Grimme's D3 model^13^ with Becke-Johnson damping factors^14^ were incorporated during geometry optimizations. Harmonic vibrational frequency calculations were performed using the same level of theory to confirm the nature of stationary points as either true minima or transition states and to provide thermodynamic corrections.

Subsequent single-point calculations were carried out on the optimised geometries, including the Douglas–Kroll–Hess 2nd order scalar relativistic Hamiltonian,^15^ and utilizing the all-electron SARC basis set for Sm, Jorge's for I^16^ and Dunning's Correlation-Consistent Triple-Zeta + polarisation (cc-pVTZ)^11^ for the remaining elements. These calculations were performed with the inclusion of respective solvent using PCM.^17^ Specifically, THF is used as the solvent for computations involving the additives THF, HMPA and TPPA; MeTHF is used as the solvent for computations involving the additive MeTHF and THP is used as the solvent for computations involving the additive THP. This is based on the fact that HMPA and TPPA are typically used exclusively as additives, while solvents like THF are employed separately. In contrast, THF, THP and MeTHF act as both solvents and additives in reactions. Note that the dielectric constant and the square of the refractive index are used to represent the static and dynamic parameters for the PCM computations involving MeTHF and THP, respectively, as they are not incorporated in Gaussian16. The energies obtained from single point calculations with solvent effects and the enthalpic corrections derived from frequency calculations collectively yielded the solvation enthalpies, *H*_sol_. The entropic contributions were determined from frequency calculations and adjusted by using the quasi-harmonic approximation proposed by Grimme.^18^ Specifically, the – TS terms were corrected directly using GoodVibes,^19^ with a cut-off frequency set to 100 cm^−1^. Energetic Span Model analyses have been conducted to understand the chemical kinetics of serial catalytic cycles, utilizing the AUTOF program developed by S. Kozuch and S. Shaik.^20^

## Results and discussion

### Basicity of LB and reducing power of SmI_2_(LB)_*n*_

Given the critical role of additives in determining the reactivity of SmI_2_, we first evaluated the reducing power of SmI_2_ in the presence of various LB additives, forming SmI_2_(LB)_*n*_ complexes. It is important to note here that the solution chemistry of SmI_2_ with strong LBs such as HMPA is complex and subject to multiple interpretations. Electrochemical and conductivity studies have suggested that iodides occupy outer-sphere positions due to repulsion by the strongly coordinating HMPA, forming the separated ion pairs [Sm(HMPA)_4_(thf)_2_]I_2_.^21^ In contrast, crystallographic and mass spectrometric studies indicate that the traditional inner-sphere coordination structure, SmI_2_(HMPA)_4_, is the dominant species.^22^ We have computationally evaluated the relative stability of [Sm(HMPA)_4_(thf)_2_]I_2_ and SmI_2_(HMPA)_4_, and found that complete displacement of iodides to the outer sphere is energetically disfavoured under typical conditions (see Fig. S1). Thus, SmI_2_(HMPA)_4_ provides a chemically reasonable and experimentally supported model that will be employed in the following studies.

**Fig. 1:**
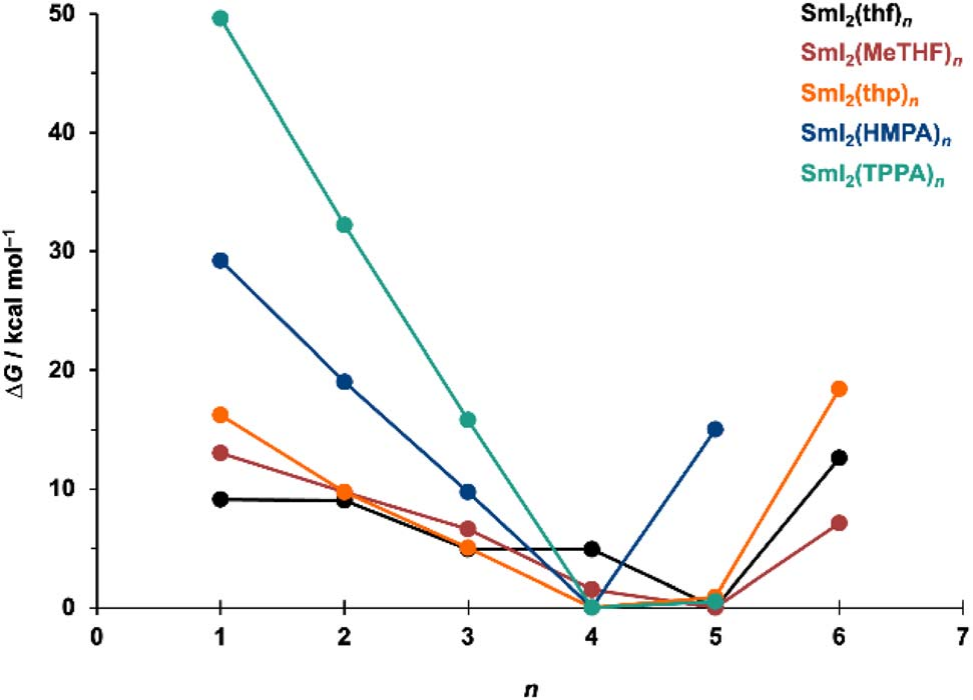
Formation Gibbs energies of SmI_2_(LB)_*n*_ complexes from SmI_2_ and LB, that is, the Gibbs free energies associated with the transformation SmI_2_ + *n* LB to SmI_2_(LB)_*n*_, calculated in solvent using the PCM model. The values are referenced to *n* = 4 for HMPA and TPPA, or *n* = 5 for THF, MeTHF, and THP, respectively. These energies indicate the thermodynamic feasibility of complex formation; for stepwise ligand addition, G[SmI_2_(LB)_*n*_] – G[SmI_2_(LB)_*n*−1_] reflects the relative stability of SmI_2_(LB)_*n*_*vs.* SmI_2_(LB)_*n*−1_, with negative values (downward trends) indicating greater stability of the *n*-coordinate complex.

To identify the most abundant SmI_2_(LB)_*n*_ species, we compare the computed Gibbs energies of SmI_2_(LB)_*n*_ in solution as the coordination number, *n*, increases (Fig. 1). The results indicate that saturation occurs at *n* = 5 for the smaller LB additives THF, MeTHF and THP, whereas saturation is achieved at *n* = 4 for the bulkier LB additives HMPA and TPPA. Our subsequent analyses focus exclusively on the species with these optimal coordination numbers and structures.

Next, we evaluated the basicity of the studied LBs by computing their protonation energies and explored potential correlations between LB basicity and the reducing power of the corresponding SmI_2_(LB)_*n*_ complexes. Stronger LBs can donate electron density more effectively, typically reflected in their higher-lying HOMOs. Upon coordination, such LBs are therefore expected to increase electron density at the Sm centre and raise the energy of the metal-centred occupied orbitals, thereby enhancing the reducing power (SET ability) of the SmI_2_(LB)_*n*_ complex. The reducing power is quantified based on the oxidation energies of SmI_2_(LB)_*n*_ to SmI_2_(LB)_*n*_^+^. The results, summarised in Table 2, reveal that cyclic ethers (THF, MeTHF and THP) are significantly weaker bases compared to the phosphoramides HMPA and TPPA, consistent with the experimentally determined trends in gas-phase basicities and proton affinities.^23^ Consequently, SmI_2_ reagents with HMPA and TPPA additives exhibit superior reducing power, consistent with previous experimental findings. Notably, TPPA displays even greater basicity than HMPA, and SmI_2_(TPPA)_4_ demonstrates higher reducing power than SmI_2_(HMPA)_4_. This highlights TPPA as an effective alternative to HMPA in SmI_2_-mediated reactions.

**Table 2:** Computed basicity of the studied LBs and reducing power of corresponding SmI_2_(LB)_*n*_ complexes, where LB = THF, MeTHF, THP, HMPA, TPPA and 2,2,5,5-tetramethyl-THF (TMTHF)

LB	Basicity^a^ of LB (Δ*G*_protonation_/kcal mol^−1^)	Reducing power^b^ of SmI_2_(LB)_*n*_ (Δ*G*_oxidation_/kcal mol^−1^)
THF	−147.9	81.2
MeTHF	−149.5	88.3
THP	−149.8	87.6
HMPA	−168.9	59.2
TPPA	−172.5	55.3
TMTHF	−157.1	108.9

a Basicity was evaluated based on the protonation reaction energy: LB + H^+^ → LBH^+^.

b Reducing power was assessed by the oxidation of SmI_2_(LB)_*n*_ to SmI_2_(LB)_*n*_^+^.

Interestingly, while the basicity of the ethers increases slightly from THF to MeTHF and THP, as indicated by their more negative protonation energies, the reducing power of the corresponding SmI_2_(LB)_*n*_ complexes does not follow this trend. Specifically, SmI_2_(thf)_5_ exhibits the highest reducing power among the three ethers, despite THF being the weakest base in this set. Although the computed basicities of THF, MeTHF, and THP are close and may fall within the systematic error of calculations, the trend aligns with chemical intuition and experiment: THP is slightly more basic than THF, and Me substitution in MeTHF modestly increases basicity. Importantly, the computed differences in reducing power, with THF at 81.2 kcal mol^−1^ and MeTHF/THP at 88.3 and 87.6 kcal mol^−1^, are far larger than the computational uncertainty and underpin our mechanistic discussion. This finding also supports the widespread use of THF as both an additive and solvent in SmI_2_-mediated reactions.

**Fig. 2:**
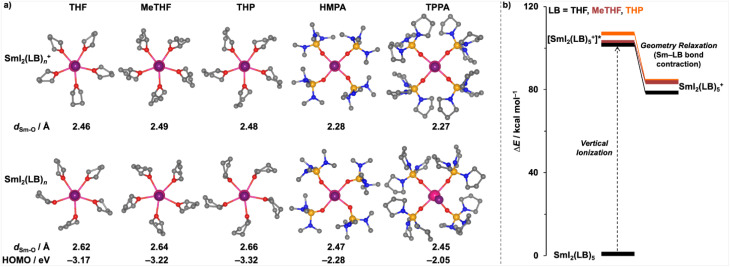
(a) Optimised geometries of SmI_2_(LB)_*n*_ (bottom) and the oxidised counterparts SmI_2_(LB)_*n*_^+^ (top), with the average Sm–O distances (*d*_Sm–O_) and the HOMO energies of SmI_2_(LB)_*n*_; (b) computed vertical ionisation energies for SmI_2_(LB)_5_ to [SmI_2_(LB)_5_^+^]* and the relaxation energies to SmI_2_(LB)_5_^+^.

To understand why THF, despite being a weaker LB, is a good additive for efficient SmI_2_ reduction chemistry, we analysed the optimised geometries of SmI_2_(LB)_*n*_ complexes and their oxidised counterparts, SmI_2_(LB)_*n*_^+^, with various LBs (Fig. 2a). The results reveal that THF is positioned closer to the metal centre (average *d*_Sm–O_ = 2.62 Å) compared to the bulkier ethers MeTHF and THP (*d*_Sm–O_ = 2.64 Å and 2.66 Å, respectively). The shorter Sm–O distance in SmI_2_(thf)_5_ should facilitate more efficient electron donation from the LB to the metal centre. This leads to a higher HOMO energy for SmI_2_(thf)_5_ (−3.17 eV, Fig. 2a) and, consequently, greater reducing power. Further support for this comes from the computed vertical ionisation energies of these complexes, as shown in Fig. 2b. SmI_2_(thf)_5_ requires less energy for vertical ionisation than SmI_2_(MeTHF)_5_ and SmI_2_(thp)_5_, indicating enhanced reducing power. Additionally, the oxidation of SmI_2_(LB)_*n*_ to SmI_2_(LB)_*n*_^+^ is accompanied by a contraction of the metal-additive distance. Notably, SmI_2_(thf)_5_ undergoes a greater bond contraction upon oxidation as compared to SmI_2_(MeTHF)_5_ and SmI_2_(thp)_5_, owing to the smaller size of THF. This greater contraction results in enhanced stabilisation (Fig. 2b), reducing the energy required for oxidation and further improving the reducing power of SmI_2_(thf)_5_.

These findings suggest that both the basicity and steric size of the LB are critical factors in determining its electron-donating ability and the corresponding reducing power of SmI_2_(LB)_*n*_ complexes. This conclusion is validated by examining an extremely bulky LB, 2,2,5,5-tetramethyl-THF (TMTHF).^24^ Despite its significantly higher basicity (*i.e.*, proton affinity) compared to the other ethers, the corresponding SmI_2_(TMTHF)_4_ is the least efficient reductant (Table 2). This highlights the detrimental effect of excessive steric hindrance on reducing power.

### Single-electron transfer (SET) of SmI_2_(LB)_*n*−1_-ketone *via* an inner-sphere mechanism

**Fig. 3:**
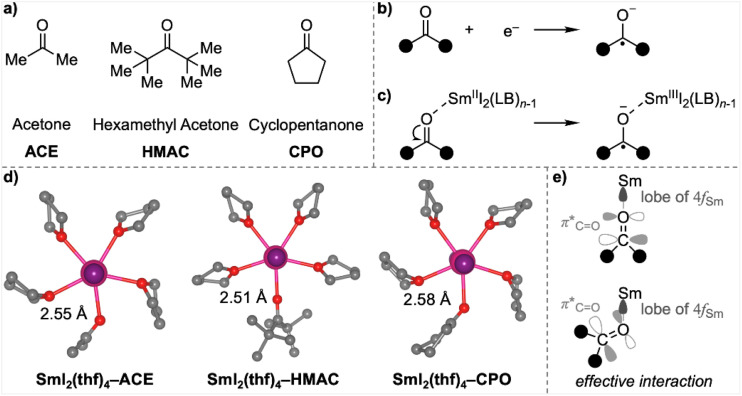
(a) Structures of the ketones studied; (b) SET reduction reaction of ketones; (c) SET within the SmI_2_(LB)_*n*−1_-ketone complexes *via* an inner-sphere mechanism; (d) optimised structures of SmI_2_(thf)_4_-ketone complexes; (e) schematic illustration of the orbital interaction between the 4f-orbital of Sm and the π*-orbital of the carbonyl group.

Having demonstrated the size effect of LB on the reducing power of SmI_2_(LB)_*n*_, we extended our analysis to single-electron transfer (SET) reductions by SmI_2_(LB)_*n*_ for three representative ketones (Fig. 3a): a simple ketone, acetone (ACE), a highly bulky ketone, hexamethyl acetone (HMAC), and a cyclic ketone, cyclopentanone (CPO). As previously discussed in Table 1, SET reductions of ketones within SmI_2_(LB)_*n*−1_-ketone complexes *via* an inner-sphere mechanism are highly favourable due to the strong interaction between the negatively charged ketyl and the positively charged Sm,^25^ which significantly stabilises the complex.

Interestingly, despite the nearly identical oxidising abilities of these three ketones (as reflected in their similar electronic energies (Δ*E*) and Gibbs energies (Δ*G*) for the reductions depicted in Fig. 3b; see entries 1–3 in Table 3), their inner-sphere SET energies within SmI_2_(LB)_*n*−1_-ketone complexes (depicted in Fig. 3c) exhibit notable differences (see entries 4–12 in Table 3). Specifically, the bulky ketone HMAC is the most resistant to reduction, with its reaction energy significantly higher than that of ACE and CPO. This difference is amplified in reactions involving bulkier additives such as MeTHF and THP. The higher SET Gibbs energy for HMAC can be attributed to two factors: a greater electronic reduction energy (Δ*E*) and a larger entropic contribution (−TΔS). The larger Δ*E* arises because HMAC, despite binding to SmI_2_ at a shorter distance compared to ACE, adopts a head-to-head orientation of its oxygen atom with Sm due to steric repulsion surrounding the molecule (Fig. 3d). This binding geometry is less conducive to efficient electron transfer from the 4f-orbital of Sm to the π*-orbital of the carbonyl, as the latter is oriented perpendicularly to the Sm–O bond (Fig. 3e). Additionally, the larger −TΔS term for HMAC than the other ketones reflects more substantial geometric changes upon bond contraction during the SET. These changes lead to greater vibrational entropic losses, further contributing to HMAC's reduced reactivity.

**Table 3:** Computed electronic energies (Δ*E*), enthalpies (Δ*H*), entropic contributions (−TΔS) and Gibbs energies (Δ*G*) for the reductions of ketones ACE, HMAC and CPO (entries 1–3), as well as for the SET reductions (Fig. 3b) of the SmI_2_(LB)_*n*−1_-ketone complexes *via* an inner-sphere mechanism, where LB = THF (entries 4–6), MeTHF (entries 7–9) and THP (entries 10–12)

Entry	LB	Ketone	Δ*E*	Δ*H*	−TΔS	Δ*G*
			kcal mol^−1^			
1		ACE	−19.1	−22.1	−0.4	−22.0
2		HMAC	−19.5	−22.6	−0.2	−22.8
3		CPO	−18.5	−21.9	−0.8	−22.8
4	THF	ACE	14.2	13.4	2.9	18.3
5		HMAC	15.4	14.9	3.5	21.1
6		CPO	15.3	14.5	1.8	17.5
7	MeTHF	ACE	15.9	15.3	2.1	18.6
8		HMAC	18.8	18.2	2.9	23.4
9		CPO	17.7	17.2	1.5	20.0
10	THP	ACE	15.6	14.9	1.9	17.8
11		HMAC	19.6	18.8	2.8	23.7
12		CPO	16.8	16.0	1.5	18.4

The cyclic ketone CPO is more difficult to reduce than ACE when bulkier LBs such as MeTHF or THP are employed, but less so than HMAC (Table 3). The higher SET electronic energy for CPO compared to ACE stems from a longer Sm–O bond distance, resulting in a less favourable interaction within the CPO complex (Fig. 3d). However, the SET reaction for CPO has a smaller increase in the −TΔS term, which partially offsets the higher Δ*E*. Consequently, the Gibbs energy for SET in CPO is only slightly higher than that for ACE and, in the case of THF, even marginally lower than ACE. This reduction in −TΔS for CPO can be attributed to the more organised and compact structure of the cyclic structure, which undergoes fewer geometric and vibrational entropic changes during the SET reaction. This observation aligns with findings from our earlier combined experimental and computational studies, further emphasising the critical role of structural rigidity in modulating entropic contributions during SET processes.^26^

These analyses suggest that the efficiency of SmI_2_-mediated reductions cannot be solely attributed to the reagent's reducing power in the presence of additives, as assumed in many previous studies. Instead, a thorough examination of the specific reagent-substrate complexes is crucial, as multiple factors can simultaneously influence reduction reactivity.

### Beyond the reduction

We next extended our investigation of additive influences on SmI_2_ catalysis beyond the single reduction reaction. Specifically, we explored the effect of additives on the SmI_2_-catalysed coupling reactions of cyclopropyl ketones with aryl alkynes, a well-established transformation that has been extensively studied.^26,27^ This reaction proceeds *via* a radical relay mechanism and addresses a key limitation of SmI_2_ chemistry: the requirement for stoichiometric quantities of the Sm-reagent.^28^

**Fig. 4:**
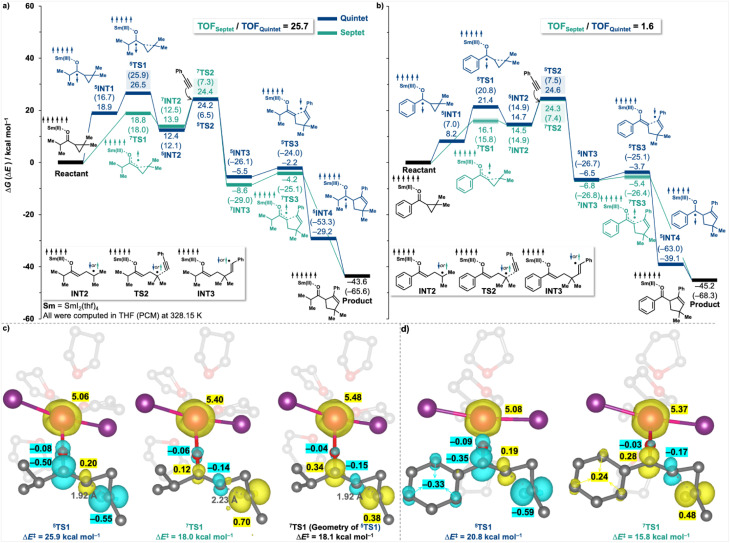
Computed Gibbs energy profiles (Δ*G*) and electronic energies (Δ*E*, in parentheses) for the SmI_2_-catalysed intermolecular coupling reactions: (a) *i*-Propyl dimethyl cyclopropyl ketone with phenylacetylene *via* the quintet (blue) and septet (green) pathways; (b) phenyl dimethyl cyclopropyl ketone with phenylacetylene *via* the quintet (blue) and septet (green) pathways. (c) and (d) show the computed geometries and spin density distributions of key transition states along respective pathways.

We first revisited the reaction mechanism using the representative coupling between *i*-propyl dimethyl cyclopropyl ketone and phenylacetylene. In the previously established framework of SmI_2_-catalysed coupling reactions (depicted in blue in Fig. 4a), the process is initiated by a single-electron transfer (SET) from SmI_2_ to the ketone, followed by a spin flip that generates a quintet ketyl radical intermediate (^5^INT1). This radical then undergoes cyclopropyl ring opening *via* transition state ^5^TS1, yielding intermediate ^5^INT2, which is subsequently trapped by a coupling partner—here, phenylacetylene. The catalytic cycle is completed through a radical rebound ring-closing process, during which the electron is returned to SmI_2_, thereby regenerating the catalyst. Here, we compared this mechanism with an alternative pathway that circumvents the formation of the quintet ketyl radical, as previously discussed by Reissig^29^ (illustrated in green in Fig. 4a). In this revised mechanistic pathway, the initial complex undergoes a concerted SET and cyclopropyl fragmentation *via* a septet transition state (^7^TS1). The resulting septet intermediate (^7^INT2) then proceeds through a sequence analogous to that of the quintet pathway, reacting with the coupling partner to afford ^7^INT3. However, this alternative route then also bypasses the conventional discrete ring-closing and electron-return steps, instead featuring a concerted ring-closing event that simultaneously returns the electron to SmI_2_, thereby completing the catalytic cycle. Strikingly, this mechanism exhibits a significant kinetic advantage: the energy barrier for SET-concerted cyclopropyl ring opening reaction *via* the septet transition state ^7^TS1 (Δ*G*^‡^ = 18.8 kcal mol^−1^) is substantially lower than that of its quintet counterpart ^5^TS1 (Δ*G*^‡^ = 26.5 kcal mol^−1^). Note that we checked the electronic properties, including computed multiplicity and spin density distribution, for every stationary point and eliminated any potential errors, such as those arising from spin contamination. Consequently, the rate-determining step in the new septet pathway becomes its subsequent radical-trapping step (Δ*G*^‡^ = 24.4 kcal mol^−1^), which remains lower in energy than ^5^TS1, the rate-determining step along the old quintet pathway. The computed turnover frequency (TOF), determined using the Energetic Span Model (ESM, *vide infra*),^20^ is 25.7 times higher for the septet pathway compared to the quintet mechanism.

Spin density analysis of TS1 offers insight into the origin of the energetic preference for the septet pathway (Fig. 4c). The septet transition state ^7^TS1 exhibits enhanced delocalisation of the unpaired electron density, with spin distributed across the cyclopropyl carbon that is undergoing bond cleavage (0.70), the carbonyl carbon (0.12), and some partially retained on the metal centre (∼0.40, in addition to its 5 unpaired f-electrons). In contrast, the quintet transition state ^5^TS1 displays more localised radical character, with spin density concentrated primarily on the cyclopropyl fragment (0.55) and carbonyl carbon (0.50). Furthermore, the geometry of ^7^TS1 is significantly more advanced along the reaction coordinate than that of ^5^TS1, as indicated by the greater elongation of the C–C bond undergoing cleavage (2.23 Å *vs.* 1.92 Å, respectively). To confirm that the observed energetic difference is not solely attributable to geometric factors, a single-point energy calculation was performed for the septet spin state using the geometry of ^5^TS1 (right panel, Fig. 4c). The resulting energy closely matched that of the fully optimised ^7^TS1, and the spin density remained similarly delocalised. This indicates that the higher stability of ^7^TS1 *vs.*^5^TS1 is predominantly attributable to electronic factors, rather than to differences in geometry.

A crucial additional example is the coupling of a phenyl-substituted dimethyl cyclopropyl ketone (Fig. 4b). In this case, attempts to optimise the geometry of septet transition state ^7^TS1 were unsuccessful, as all optimisations reverted to either the initial complex or the septet intermediate ^7^INT2. Nevertheless, a single-point energy calculation of the septet spin state, using the optimised geometry of the corresponding quintet transition state, yielded a structure with only one imaginary frequency—indicating that ^7^TS1 is geometrically similar to ^5^TS1. Given our earlier observation that the energy of the septet spin state is relatively insensitive to geometric variation (Fig. 4c), the ^5^TS1 geometry was considered a reasonable structural approximation for ^7^TS1. The difficulty in locating the optimised ^7^TS1 structure likely arises from a very shallow energy barrier separating it from ^7^INT2, a consequence of conjugative stabilisation of the radical intermediate by the phenyl substituent. This mechanistic feature likely accounts for the inability to identify a septet transition state in such systems in previous studies, which instead focused on the quintet pathway *via*^5^TS1. Importantly, our revised mechanism maintains the reaction kinetics for phenyl-substituted cyclopropyl ketones; specifically, the key rate-determining transition state remains at TS2, and the computed overall TOF remains nearly unchanged (relative TOF = 1.6, see Fig. 4b), thereby affirming the consistency of our findings with prior computational analyses.

**Fig. 5:**
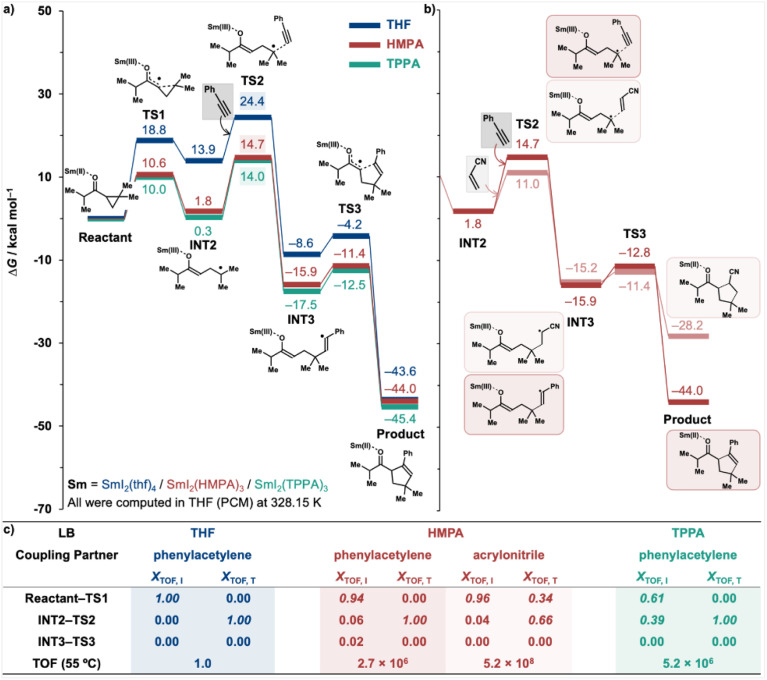
(a) Computed Gibbs energy profiles for the SmI_2_-catalysed coupling reaction between *i*-propyl dimethyl cyclopropyl ketone and phenylacetylene in the presence of different additives: THF (blue), HMPA (red), and TPPA (green); (b) Gibbs energy profiles for the radical-trapping step and subsequent reactions in the coupling of *i*-propyl dimethyl cyclopropyl ketone with phenylacetylene and acrylonitrile (shown in faded red), catalysed by SmI_2_ in the presence of HMPA; (c) degree of TOF control for intermediates (*X*_TOF, I_) and transition states (*X*_TOF, T_) in the SmI_2_-catalysed coupling reactions of *i*-propyl dimethyl cyclopropyl ketone with phenylacetylene and acrylonitrile.

Having established a revised reaction mechanism for the SmI_2_-catalysed coupling reaction of cyclopropyl ketones, we next returned to the effect of LB additives, focusing on the intermolecular coupling of *i*-propyl cyclopropyl ketone with phenylacetylene in the presence of the commonly used THF and the more strongly coordinating LBs, HMPA and TPPA (Fig. 5a). While noting that certain additives could suppress catalytic activity under particular experimental conditions, we here restricted our discussion to their promoting effects on the model reactions under idealised conditions. The results clearly demonstrate that efficient LB additives such as HMPA and TPPA significantly enhance the reduction reaction by lowering the energy barrier associated with the transition state TS1. This effect extends across the entire potential energy surface (PES), resulting in computed TOF increases of 2.7 × 10^6^ and 5.2 × 10^6^ for HMPA and TPPA, respectively.

An energetic span model analysis^20^ was subsequently performed to identify the key stationary points (intermediates and transition states) governing the TOF and to enable a comparative evaluation of reaction kinetics when using different additives (Fig. 5c). This model provides a robust framework for interpreting catalytic efficiency, in which the TOF-determining transition state (TDTS) and the TOF-determining intermediate (TDI) define the apparent activation barrier of the catalytic cycle. The degree of TOF control (*X*_TOF_), adapted from the classical concept of rate control in physical organic chemistry, quantifies the extent to which each stationary point influences the TOF: a larger *X*_TOF_ value indicates a greater contribution to reaction kinetics. Importantly, the TDTS and TDI are not necessarily the highest- or lowest-energy states, respectively, and multiple stationary points may collectively affect the overall kinetics. Under THF and HMPA conditions, the TDI is the reactant, as reflected by *X*_TOF, I_ value of 1.00 and 0.94, respectively (Fig. 5c). In contrast, under TPPA, the PES is substantially stabilised, bringing intermediate INT2 (0.3 kcal mol^−1^) close in energy to the reactant (Fig. 5a). Consequently, the TDI is shared between the reactant (*X*_TOF, I_ = 0.61) and INT2 (*X*_TOF, I_ = 0.39). This suggests that, in reactions promoted by strongly coordinating LB additives, the TDI may not be governed solely by the reactant but may also involve the radical intermediate INT2. Additional stabilisation of this radical—arising from the intrinsic properties of the reactant—may inadvertently increase the effective energetic span, thereby diminishing the overall reaction kinetics relative to other reactants under the same additive conditions.

Moreover, in the presence of strong LBs such as HMPA, TS2 is stabilised significantly and the energy gap between TS1 and TS2 decreases (Fig. 5a). Furthermore, when the coupling partner is changed from phenylacetylene to acrylonitrile, the energy barrier for the radical-trapping transition state TS2 drops markedly from 14.7 to 11.0 kcal mol^−1^ (Fig. 5b), approaching that of TS1 at 10.6 kcal mol^−1^. As a result, both TS1 and TS2 become kinetically significant in determining the reaction rate. This is corroborated by the computed *X*_TOF, T_ values under HMPA for the acrylonitrile reaction (Fig. 5c): 0.34 and 0.66 for TS1 and TS2, respectively. These findings suggest that for highly reactive coupling partners under strongly coordinating LBs, TS1 may once again emerge as the rate-determining transition state.

Collectively, these findings reveal that the influence of LB additives extends beyond the SET, modulating the entire potential energy surface of SmI_2_-catalysed reactions. This modulation can have profound implications for both the chemical reactivity and selectivity of SmI_2_-mediated processes.

## Conclusions

In this contribution, we have provided a systematic computational understanding of the role of Lewis base (LB) additives in SmI_2_ chemistry, focusing on their influence on the reducing power of SmI_2_, the efficiency of the single electron transfer (SET) reduction and the impact on an exemplar SmI_2_-catalysed coupling reaction. First, our findings demonstrate that both the basicity and coordination geometry of the LB additives with the reagent SmI_2_ are critical in determining the electronic properties and reducing power of the SmI_2_(LB)_*n*_. For instance, strong LBs such as hexamethylphosphoramide (HMPA) and tripyrrolidinophosphoramide (TPPA) outperform commonly used ethers due to their strong electron-donating effects toward the metal, enhancing the reducing power of SmI_2_. On the other hand, while the classical cyclic ether additive tetrahydrofuran (THF) exhibits a weaker basicity compared to analogues like 2-methyl-THF (MeTHF) and tetrahydropyran (THP), it can still significantly enhance the reducing power of SmI_2_ by adopting an optimal coordination geometry that can facilitate electron donation to Sm. This underscores the importance of coordination geometry, where less bulky LBs can achieve closer coordination to the metal, improving electron donation and, consequently, the reducing power of SmI_2_.

We also show that both steric and electronic effects from the LB and the substrate play pivotal roles in governing the SET reduction reactivity of SmI_2_. By calculating the Gibbs energies for the SET reduction of various ketones, we find that substrates such as acetone are more readily reduced compared to sterically hindered substrates, such as highly substituted ketones, which require higher energy for SET reduction, and the overall enhanced reactivity is determined by a combination of electronic effects and entropic contributions.

Lastly, we extend our study to SmI_2_-catalysed intermolecular coupling reactions, completing our investigation into the influence of LB additives in practical catalytic systems. We reveal a revised reaction mechanism for the coupling of cyclopropyl ketones with aryl alkynes (and electron-deficient alkenes), in which a lower-energy septet pathway offers, for certain coupling partners at least, enhanced efficiency compared to the conventional quintet route. Electronic structure analysis shows that this advantage arises from improved radical delocalisation. Importantly, we find that strongly coordinating additives can markedly reshape the potential energy surface—accelerating key steps and shifting the rate-determining stationary points depending on the coupling partner. These studies demonstrate that LB additives can modulate the entire catalytic landscape, providing a valuable foundation for the rational design of SmI_2_-mediated transformations.

In conclusion, our results provide valuable insights into the complex interplay between LB basicity, coordination geometry, and reducing power in SmI_2_ chemistry. This work highlights the multifaceted role of LB additives in reaction systems, offering a mechanistic understanding of their impact on reaction kinetics and providing practical guidelines for designing more efficient and selective transformations.

## Author contributions

Conceptualization: SY, DJP, NK. Investigation: SY. Formal analysis: SY, NK. Draft writing: SY, NK. Funding acquisition: DJP, NK. Resources: NK. Supervision: NK, DJP. Reviewing and editing: all authors.

## Conflicts of interest

The authors declare no conflict of interest.

## Supplementary Material

SC-OLF-D5SC08336B-s001

## Data Availability

The data supporting this article have been included as part of the supplementary information (SI). Supplementary information: Cartesian coordinates and energies of all stationary points, along with additional figures and analyses supporting this work. See DOI: https://doi.org/10.1039/d5sc08336b.
