# Supplementary material for: Computational study of the effect of Lewis base additives and molecular spin state in SmI2-chemistry
Source: Chem Sci. 2026 Mar 10;17(17):8744–52. doi: 10.1039/d5sc08336b (PMC12983404; doi:10.1039/d5sc08336b)
Supplement: SC-017-D5SC08336B-s001 [file SC-017-D5SC08336B-s001.pdf]

Supplementary Information (SI) for Chemical Science.
This journal is © The Royal Society of Chemistry 2026

Supplementary Information

Computational Study of the Effect of Lewis Base Additives and Molecular Spin State in SmI_2-Chemistry

Song Yu,[ab] Ciro Romano,[b] David J. Procter[b] and Nikolas Kaltsoyannis*[b]

[a]*College of Life Sciences, Huzhou University, Huzhou, Zhejiang, 313000 P. R. China*

[b]*Department of Chemistry, School of Natural Sciences, The University of Manchester, Manchester, M13 9PL, U.K.*

**E-mail: nikolas.kaltsoyannis@manchester.ac.uk*

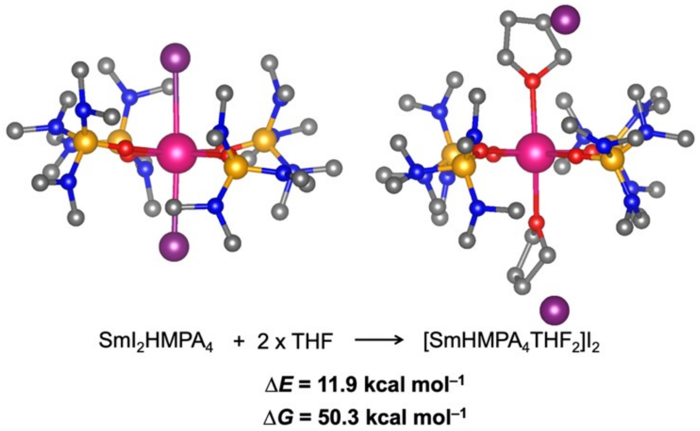

Figure S1. Relative energies of $SmI_2(HMPA)_4$ and the outer-sphere structure $[Sm(HMPA)_4(thf)_2]I_2$, optimised and computed using the implicit PCM model in THF solvent.

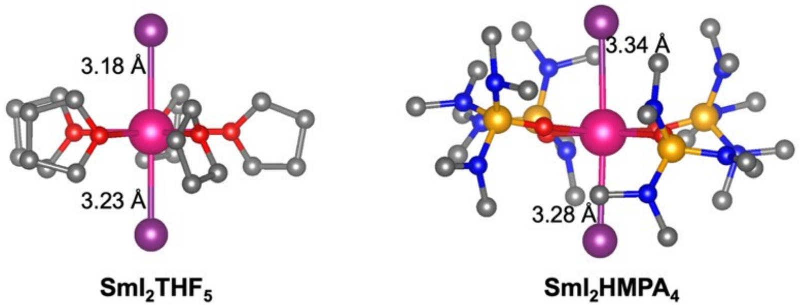

Figure S2. Optimised structures with corresponding Sm–I bond distances for $SmI_2(THF)_5$ and $SmI_2(HMPA)_4$.

Table S1. Experimentally determined proton affinities and gas-phase basicities of representative Lewis bases (*J. Phys. Chem. Ref. Data*, 1998, **27**, 413–656.)

Lewis bases	Proton affinity	Gas basicity

	kJ/mol	kJ/mol
THF	822.1	794.7
THP	822.8	795.4
HMPA	958.6	928.7

Cartesian coordinates (in Å) of geometries optimised at the PBE0 functional with dispersion corrections from D3-BJ, using cc-pVDZ basis sets for C, H, and O, and effective core potentials (ECPs) for Sm and I. Enthalpy corrections, H_{corr}, and $-TS$ terms obtained from frequency calculations and corrected using Grimme's method. Single-point energies, E_{sol}, computed using the Douglas-Kroll-Hess 2nd order scalar relativistic Hamiltonian, with the all-electron SARC basis set for Sm, Jorge for I and cc-pVTZ for the remaining elements. Single-point calculations were performed with the inclusion of the respective solvent using PCM. Gibbs free energies, G_{sol}, computed by incorporating above energy terms. All energies are given in Hartree.

[Sm(HMPA)$_4$(thf)$_2$]I$_2$

H_{corr} = 1.3662
$-TS$ = -0.2013
$E_{sol(THF)}$ = -28384.0958
$G_{sol(THF)}$ = -28382.9309

H	4.380945	3.872983	1.202711
H	6.135309	3.882720	0.901794
C	6.316514	1.274027	0.256522
H	0.548793	0.740762	-3.788934
N	3.108365	2.233871	2.546979
C	2.010683	3.041802	2.039760
H	3.310687	0.372641	-6.201024
C	5.184857	3.390640	0.635286
P	-1.431459	1.304826	2.999745
O	-0.305994	0.590768	2.238254
H	2.322556	4.090849	1.872085
H	6.217691	0.192266	0.412655
Sm	0.466854	-0.094089	0.009148
N	-2.574486	0.186816	3.497799
C	-2.142493	-1.161340	3.834917
H	4.434455	2.031099	4.168979
H	4.316614	3.697299	3.529381
C	-3.814652	0.595353	4.126703
I	-3.322946	4.755646	-1.621157
I	-2.177404	-4.888150	1.928693
N	-0.687335	2.162290	4.268641
C	0.411135	1.482349	4.932537
H	3.934600	-1.097351	-5.418005
H	2.445651	-1.174711	-6.410483
C	-1.535795	2.910211	5.179657
N	4.658010	-0.073795	2.419115
N	2.059546	-2.926843	-3.608378
C	3.960454	-1.328401	2.620181
H	6.289236	1.473374	-0.828476
H	7.302627	1.594104	0.635949
N	-2.321088	2.487456	2.215572
C	-1.586859	3.694503	1.830199
H	1.645703	2.637110	1.089195
H	1.177492	3.037121	2.757838
C	-3.248219	2.021889	1.174075
H	1.051340	0.999146	4.183614
H	1.007254	2.225385	5.487941
H	5.000745	3.566177	-0.440631
H	-2.021577	-1.287166	4.927016
H	-3.766193	0.503808	5.227545
H	0.067268	0.720829	5.660065
H	-2.363640	3.372580	4.627010
H	-1.956970	2.278748	5.985201
H	6.293591	1.083395	3.044998
H	5.596053	0.034806	4.317230
H	-0.945437	3.712071	5.652505
H	4.664398	-2.166095	2.480016
H	-2.280755	4.375172	1.316282
C	5.836074	0.105967	3.240463
H	3.536054	-1.405878	3.637393
H	3.154719	-1.432636	1.884728
H	-1.162447	4.186469	2.715027
H	-0.769502	3.481327	1.118855
H	-2.734674	1.494027	0.354131
H	6.579461	-0.677871	3.012428
C	3.750739	2.766474	3.728880
H	2.981259	2.995407	4.481919
H	-3.997861	1.346215	1.602350
H	-3.743790	2.900456	0.737584
H	-2.868977	-1.906899	3.476673
H	-1.190835	-1.375722	3.341043
H	5.639456	-0.147293	-2.708725
N	5.257243	1.979217	0.951574
H	5.323317	-2.365654	-1.638255
H	2.818116	-3.709251	-5.437334
H	-4.053266	1.634510	3.868603
H	-4.635715	-0.049486	3.771656
H	0.599546	-4.412854	-3.361463
H	1.330226	1.505452	-5.203207
O	1.188024	-0.902769	-2.213011
P	2.274136	-1.309864	-3.199124
N	3.882028	-1.278643	-2.702119
C	4.552290	0.010065	-2.618040
H	4.041743	-3.270405	-4.205728
H	3.042873	-4.732980	-3.993627
C	4.242325	-2.156178	-1.593688
H	4.219695	0.662633	-3.433121
H	4.341292	0.503922	-1.655020
C	3.039400	-3.690637	-4.353979
H	0.008255	-2.735056	-3.202825
H	0.382722	-3.408550	-4.823882

C	0.690070	-3.389769	-3.761113
H	4.009999	-1.690450	-0.619376
H	3.703431	-3.108412	-1.668681
N	2.250817	-0.239376	-4.502009
C	1.051822	0.543865	-4.741888
O	2.929327	0.559036	0.545814
P	3.915744	1.149256	1.550882
C	3.016827	-0.558828	-5.690153
H	0.343170	0.032190	-5.420104
P	-2.998344	-0.998414	-1.485149
O	-1.804574	-0.584673	-0.613714
N	-2.542533	-2.338427	-2.423991
C	-2.188423	-3.499127	-1.612090
C	-3.235499	-2.688967	-3.647945
N	-4.316820	-1.328174	-0.511482
C	-4.147779	-1.522989	0.920223
C	-5.443431	-2.032965	-1.091437
N	-3.531716	0.115319	-2.604903
C	-4.627249	1.041468	-2.336677
C	-2.566295	0.603011	-3.575140
H	-3.282586	-0.949163	1.266020
H	-3.065589	-4.123345	-1.367275
H	-4.076130	-3.385762	-3.468783
H	-3.987150	-2.584505	1.182039
H	-5.528181	-1.809626	-2.164031
H	-5.364128	-3.127033	-0.955723
H	-6.375461	-1.696426	-0.607956
H	-5.302815	1.072936	-3.208629
H	-5.193990	0.701564	-1.462494
H	-4.251662	2.063432	-2.145330
H	-2.246220	1.627981	-3.320187
H	-1.688903	-0.057912	-3.600502
H	-3.013672	0.628045	-4.584302
H	-1.462258	-4.130309	-2.146266
H	-1.744010	-3.192978	-0.656808
H	-3.626590	-1.790968	-4.140871
H	-2.532145	-3.188822	-4.336442
H	-5.049445	-1.149292	1.436046
H	2.206140	2.327683	-2.791725
H	2.909274	2.242570	-1.150451
C	2.098981	2.676140	-1.748344
O	0.873038	2.174547	-1.219976
H	2.398761	4.666926	-2.588706
C	1.982519	4.203084	-1.682834
H	-0.330485	2.850827	-2.773999
C	-0.139650	3.073711	-1.709256
H	2.539030	4.607229	-0.824328
C	0.468659	4.454441	-1.533973
H	-1.073433	2.921668	-1.155436
H	0.054154	5.164285	-2.258998
H	0.222817	4.845359	-0.536531
H	-0.184688	-3.022922	2.451867
H	1.191766	-1.957733	2.959392
C	0.886442	-2.797045	2.320046
O	1.094842	-2.383589	0.951908
H	1.050279	-4.857415	2.924563
C	1.703171	-4.082542	2.498391
H	0.212688	-4.079275	0.147430
C	1.208858	-3.609112	0.212408
H	2.569741	-3.945813	3.161786
C	2.133619	-4.455187	1.068470
H	1.584045	-3.357965	-0.788592
H	2.015393	-5.527325	0.860359
H	3.185102	-4.179080	0.884712

Coordination Number

THF

H_{corr} = 0.1228

$-TS$ = -0.0336

$E_{sol(THF)}$ = -232.3735

$G_{sol(THF)}$ = -232.2843

H	-1.51924500	-0.46776000	1.17979000
H	-1.95048600	-0.82238200	-0.51542700
H	1.51923300	-0.46777500	-1.17979200
H	1.95047000	-0.82242100	0.51542300
H	-0.77382200	1.13187500	-1.32585900
H	-1.34611900	1.76205400	0.24006500
H	1.34614100	1.76203400	-0.24007300
H	0.77385200	1.13187200	1.32586000
O	-0.00001000	-1.24332500	0.00000100
C	-1.15399400	-0.42644700	0.13504400
C	-0.72596800	0.98805300	-0.23399700
C	0.72598400	0.98804300	0.23399900
C	1.15398700	-0.42646500	-0.13504500

$SmI_2(thf)_1$

H_{corr} = 0.1322

$-TS$ = -0.0572

$E_{sol(THF)}$ = -24868.3985

$G_{sol(THF)}$ = -24868.3235

H	0.94587300	2.87698700	-1.75734400
H	0.94627200	4.48025300	-0.99839100
I	-2.74606700	-0.88368500	-0.41643200
I	2.81960800	-0.74651500	-0.41396300
O	-0.06865400	1.97294900	0.80760600
C	-1.25688600	2.74001100	0.51983900
C	-0.97374300	3.38788300	-0.83036400
C	0.56811900	3.45356200	-0.90147600
C	1.02981600	2.81968400	0.40679200
H	-1.38166200	3.48585500	1.32367800
H	-2.10772500	2.04500700	0.51885500
H	1.92178400	2.18425500	0.31620300
Sm	0.02839800	-0.47694900	0.71484600
H	1.18611500	3.56973400	1.20093800
H	-1.37705700	2.76239900	-1.63903600
H	-1.44654200	4.37652900	-0.90400800

$SmI_2(thf)_2$

H_{corr} = 0.2571

$-TS$ = -0.0715

$E_{sol(THF)}$ = -25100.7936

$G_{sol(THF)}$ = -25100.6079

H	-1.10759500	-3.75613500	3.06709500
H	2.15540500	-1.33670300	1.91454500
O	0.13136100	-1.09116300	1.57269800
H	1.34544100	-2.99733400	3.97548900
H	1.23249000	-3.57112800	2.29284500
C	-0.97117100	-2.00801200	1.76490400
C	-0.64157500	-2.76188700	3.03966300
C	0.88374900	-2.79985700	2.99866900
C	1.21491100	-1.41532300	2.47685000
H	-1.32185400	-0.98050400	-3.43194300
H	-2.21900900	-1.15658900	-1.88951200
H	0.98855200	-2.53113700	-0.77917100

Sm	0.03654800	0.75491800	-0.07379800
H	1.79926900	-1.63503800	-2.09403300
H	-1.53588400	-3.50183100	-1.69130000
H	-1.76750500	-3.38267400	-3.45412700
H	0.68891500	-4.14966200	-2.55558600
H	0.61537500	-2.87982100	-3.79983100
I	-3.00403700	1.22939900	0.16642500
I	3.09852600	1.06760000	-0.16169600
O	-0.16467700	-1.08615100	-1.70872300
C	-1.33621600	-1.48985200	-2.45340400
C	-1.19188300	-2.99018400	-2.60501900
C	0.31833700	-3.13844200	-2.77146000
C	0.85601400	-2.10996900	-1.78998700
H	-0.98860100	-2.19639300	3.91875300
H	-1.01015500	-2.68255100	0.89160900
H	-1.90015600	-1.42097900	1.79948200
H	1.22497400	-0.66773000	3.28747500

$SmI_2(thf)_3$

H_{corr} = 0.3827

$-TS$ = -0.0848

$E_{sol(THF)}$ = -25333.1966

$G_{sol(THF)}$ = -25332.8987

H	-4.72688700	-2.62074700	-0.73726800
H	-1.01728200	-2.43966500	2.59714300
H	-0.44055700	-2.29047500	4.26876100
H	-4.18302900	0.02423700	-1.93944000
H	-5.44060400	-0.33025900	-0.74124500
H	-2.39875500	-0.59360700	2.89433800
H	1.42964500	-0.92764200	3.60994400
H	1.22746300	-1.82713300	2.06136000
H	-1.78242300	-0.32299300	4.53790400
H	-1.15454500	1.40241100	2.36573800
H	-0.09314900	1.17496600	3.78756800
H	4.74743600	2.56604000	-0.30012500
Sm	0.06422400	0.11614200	-0.36969900
O	0.35548100	0.03223500	2.12592400
C	0.72701500	-1.18043400	2.79581600
C	-0.58735300	-1.74097400	3.32896800
C	-1.48451500	-0.49502300	3.49456800
C	-0.62257000	0.65411600	2.97054500
I	0.88428100	-2.85169900	-0.97441200
I	-0.88829000	3.07241000	-0.64769000
O	2.48316800	0.79226900	-0.28503000
C	3.64858700	0.08878400	-0.75757000
C	4.74220300	0.46124800	0.22250200
C	4.39918800	1.91856900	0.51991200
C	2.87904500	1.89201900	0.56700900
H	-3.49211000	-2.20123800	-1.93862400
H	-3.24620400	-2.23590300	1.09818100
H	-1.97545300	-2.61820500	-0.11876600
H	-3.17773500	1.23170000	-0.10945600
H	-4.00629900	0.18920400	1.09925100
O	-2.33477600	-0.61846200	0.18684800
C	-2.82777900	-1.96422000	0.11112200
C	-3.91159500	-1.91958600	-0.96259300
C	-4.37067800	-0.44476100	-0.96325100
C	-3.50711600	0.20758400	0.11261400
H	3.88525400	0.43872200	-1.77767700
H	3.39859800	-0.98088700	-0.79410900
H	2.49854600	1.67950900	1.57984100
H	2.39685300	2.80781400	0.19587700
H	4.65995200	-0.15233800	1.13423600
H	5.74832200	0.32311100	-0.19643400
H	4.83595600	2.29264100	1.45595800

$SmI_2(thf)_4$

H_{corr} = 0.5078

$-TS$ = -0.0980

$E_{sol(THF)}$ = -25565.5929

$G_{sol(THF)}$ = -25565.1831

H	-2.02062500	-2.86870400	-4.08641200
H	-1.58923000	1.98974600	-3.28927700
H	-1.23654600	3.70124700	-3.58709300
H	0.23013300	-4.12393900	-3.16646700
H	0.40221500	-2.88226100	-4.42589700
H	0.64959700	1.37994100	-3.55765500
H	-0.88660000	4.15790100	-1.28745000
H	-1.83391600	2.65290300	-1.00437800
H	1.01332700	3.03518800	-4.09760500
H	2.08421500	2.02466100	-1.72309600
H	1.56386000	3.74081700	-1.84218800
O	0.25154800	1.07920700	2.47230100
Sm	0.12670600	0.08481500	0.09485600
O	0.20809400	2.43132300	-0.98654900
C	-0.95843000	3.07896200	-1.51353200
C	-0.91500500	2.81553700	-3.02158800
C	0.55909400	2.44312100	-3.29127400
C	1.23907700	2.68985200	-1.95065900
I	-3.07881800	0.17952800	0.19907400
I	3.28290100	-0.18052700	-0.04557700
O	0.04434400	-2.24809800	1.09291700
C	-1.06285400	-3.16556200	1.06624400
C	-1.16125900	-3.69017100	2.48410500
C	0.31079500	-3.77507500	2.87918300
C	0.89698400	-2.53137800	2.22738600
H	-1.83252300	-3.05752500	-2.32208900
C	-0.89404000	1.60481600	3.15592000
C	1.27803100	2.06080900	2.67564300
C	0.57325900	3.39601900	2.45260600
C	-0.89641000	3.09724500	2.82430900
O	-0.04290900	-1.00048700	-2.21715300
C	-1.21832600	-1.12471200	-3.03451900
C	-1.38396800	-2.61694700	-3.22718300
C	0.06759200	-3.06100300	-3.39202000
C	0.81448600	-2.14943900	-2.42329100
H	-0.83606500	-3.97660400	0.34995900
H	-1.94815500	-2.60900300	0.72528500
H	0.86268800	-1.65593100	2.89700600
H	1.92694300	-2.64836400	1.86247500
H	-1.69433400	-2.96092800	3.11489100
H	-1.68970400	-4.65146500	2.54455200
H	0.47510000	-3.78939900	3.96532300
H	0.76672900	-4.68385800	2.45519400
H	2.09844000	1.83592200	1.97889800
H	1.65434200	1.96267900	3.70992900
H	0.65328000	3.68793200	1.39583200
H	1.01687900	4.19572400	3.06164000
H	-1.56697400	3.29383500	1.97520800
H	-1.25117200	3.69589900	3.67478900
H	-0.76309200	1.42689600	4.23855400
H	-1.77735200	1.06231700	2.79318000
H	-1.04518600	-0.60920400	-3.99736900
H	-2.04655200	-0.64068800	-2.49722400

H	0.97741500	-2.61925300	-1.44019400
H	1.78791600	-1.80005400	-2.79485200

$SmI_2(thf)_5$
H_{corr} = 0.6327
$-TS$ = -0.1093
$E_{sol(THF)}$ = -25797.9987
$G_{sol(THF)}$ = -25797.4752

H	-3.94229200	3.43377200	-2.25238600
H	-4.71215700	-0.81676600	-1.52287900
H	-5.03744700	-2.35698900	-0.71438600
H	-3.31319400	4.53043600	0.16764200
H	-4.28562800	3.03872000	0.15740700
H	-4.13330200	0.27213600	0.53499500
H	-2.84640100	-3.04708400	-1.06985200
H	-2.41931700	-1.49583600	-1.83990600
H	-5.22549000	-0.95497300	1.23183700
H	-2.60904800	-0.85574500	2.06949400
H	-3.31563400	-2.45814100	1.66344200
O	0.80935200	-2.52001700	0.61424800
Sm	0.03580600	-0.07136300	0.10264100
O	-2.16269900	-1.54953100	0.19176400
C	-2.90908300	-1.94951300	-0.96648000
C	-4.35847100	-1.49178600	-0.73083600
C	-4.31023900	-0.80831700	0.64197900
C	-3.08904500	-1.44557000	1.27534100
I	0.26989100	-0.60462100	-3.07494800
I	-0.19293100	0.49246400	3.23210500
O	0.99519600	2.33250100	-0.18923800
C	1.00400800	3.06044700	-1.42494300
C	2.25198300	3.92081300	-1.36813400
C	2.31632700	4.26123600	0.11843100
C	1.87786200	2.95535600	0.76258100
O	2.64149700	-0.14232100	0.27568500
C	3.54376000	0.01376900	-0.82610200
C	4.65073200	-0.98637300	-0.55962100
C	4.78055600	-0.88832500	0.95835800
C	3.33201400	-0.72183800	1.40094300
O	-1.93066700	1.58998400	-0.40034800
C	-2.61202600	1.80542000	-1.64786600
C	-3.06062200	3.25594900	-1.62125400
C	-3.31500800	3.47344300	-0.13241800
C	-2.17352100	2.69238500	0.49359400
H	0.09357000	3.68588100	-1.47877800
H	0.98221400	2.33255700	-2.24951200
H	2.72513700	2.27193400	0.93313000
H	1.33506400	3.07101400	1.71156200
H	3.13335400	3.33200100	-1.66965800
H	2.18885800	4.80160200	-2.02198800
H	3.31202000	4.57976900	0.45672800
H	1.60394900	5.06826800	0.35447000
H	3.93634800	1.04871900	-0.83285200
H	2.97625400	-0.15862500	-1.75225900
H	2.84597000	-1.68362200	1.62271400
H	3.20044500	-0.06147800	2.27062500
H	4.32422900	-1.99549800	-0.85886800
H	5.57855300	-0.74977000	-1.09858600
H	5.25678200	-1.76529700	1.41817200
H	5.37511800	-0.00117000	1.22958200
H	-3.47921400	1.12360800	-1.69619000
H	-1.92292800	1.54774100	-2.46508300
H	-1.25072500	3.29277700	0.55943500
H	-2.38386800	2.28515700	1.49248800
H	-2.25161000	3.91631600	-1.97328500
C	1.26378300	-3.40707500	-0.40710500
C	0.16427700	-3.34908200	1.58138800
C	-0.58930100	-4.40656300	0.77113300
C	0.11077600	-4.38529200	-0.60410300
H	-0.46100300	-2.70130900	2.20946400
H	0.93955500	-3.80912000	2.22144700
H	-1.64933500	-4.13663300	0.67369100
H	-0.53599700	-5.39100200	1.25682500
H	-0.56297900	-4.00247700	-1.38412700
H	0.46417400	-5.37586700	-0.92223300
H	2.17919100	-3.91979600	-0.05407400
H	1.49391000	-2.80585400	-1.29748700

$SmI_2(thf)_6$
H_{corr} = 0.7586
$-TS$ = -0.1213
$E_{sol(THF)}$ = -26030.3767
$G_{sol(THF)}$ = -26029.7394

I	0.01619300	-0.00837900	-3.24486300
H	1.47350200	3.71429800	-0.76724000
H	2.04319900	2.17123800	-1.46012500
H	3.68157400	4.31335200	1.73529300
H	4.23405800	2.63299600	1.78045600
H	3.79604800	4.22045700	-0.65272600
O	1.61716000	2.12039100	0.54390700
C	2.06607900	2.84324300	1.68589400
C	3.48407600	3.30667400	1.34118200
C	3.52740700	3.25564600	-0.20031700
C	2.11128400	2.83167400	-0.58470100
H	0.83444800	-2.82174900	2.54798100
Sm	-0.00300200	0.00128500	0.00083500
H	4.26288100	2.51553700	-0.54761000
H	2.46974900	-3.06841800	1.87315900
H	-2.50937500	3.05918100	-1.88196700
H	1.98379500	2.17278000	2.55042200
H	1.39910500	3.71050500	1.85211700
I	0.01733100	0.00102000	3.24663300
H	3.90957000	0.61827500	-1.85530700
H	2.87235800	-0.66641400	-2.53811900
H	5.55214200	-1.18410600	0.67169700
H	4.30467200	-2.43723100	0.57327400
H	5.57770400	-1.04882500	-1.71707400
O	2.64506800	-0.35335800	-0.53450300
C	3.50933000	-0.41346700	0.59371200
C	4.58228800	-1.43375200	0.21920800
C	4.60757600	-1.38204600	-1.32265400
C	3.49435400	-0.39185400	-1.67706200
H	-0.88455300	2.78547600	-2.57523900
H	4.40420300	-2.37244900	-1.75512900
H	-1.76327300	5.41023000	0.59784600
H	-0.05223500	4.96308700	0.50912200
H	2.90431000	-0.67357700	1.47317400
H	3.95666600	0.58158000	0.76403400
H	-3.96720400	-0.57501800	-0.72190700
H	-2.93080200	0.69119900	-1.43430900
H	-5.55702900	1.05729300	1.80236400
H	-4.37178800	2.37126400	1.81481000
H	-5.57390300	1.17285300	-0.58731400
O	-2.64422000	0.35661800	0.56674800
C	-3.47961200	0.38508100	1.71979500

C	-4.59260700	1.38227600	1.38768500
C	-4.59771900	1.43274900	-0.15460000
C	-3.52327100	0.42099000	-0.54958400
H	-2.48479800	3.14781700	0.73195100
H	-4.33766400	2.43849100	-0.51545900
H	-0.15072200	4.96775200	-1.82007800
H	-0.86652000	2.87593900	1.43496300
H	-2.84627700	0.64857300	2.57602200
H	-3.89608300	-0.62579000	1.89154400
H	-1.42340000	-3.68200000	-1.88191000
H	-2.01071000	-2.13858900	-2.56429700
H	-3.80215200	-4.23054200	0.62006900
H	-4.28024600	-2.52735600	0.54464300
H	-3.70814900	-4.28423700	-1.76997600
O	-1.63964000	-2.10455200	-0.55800400
C	-2.12584200	-2.83102600	0.56424200
C	-3.54206900	-3.25708700	0.18136300
C	-3.50712800	-3.28420300	-1.36103000
C	-2.09056800	-2.81683100	-1.70571100
C	-1.05814800	4.69149500	0.15746200
H	-4.25844200	-2.60258200	-1.78559000
C	-1.11357100	4.66391500	-1.38433300
C	-1.42451800	3.20186600	-1.71572300
H	-2.05715500	-2.18049200	1.44690700
H	-1.48346700	-3.71275900	0.73387000
H	2.47394000	-3.13006600	-0.74583900
H	0.86276100	-2.85477800	-1.46308400
H	1.87445600	-5.35059500	1.73225800
H	0.13916500	-5.00667300	1.76337600
H	1.75356000	-5.39849300	-0.65682700
O	0.99864300	-2.46898700	0.54504800
C	1.38921800	-3.22073300	1.68974300
C	1.09831300	-4.68183300	1.33463500
C	1.04700500	-4.68675700	-0.20749200
C	1.38837100	-3.24588600	-0.58102800
H	-1.88101700	5.33730900	-1.79087900
H	0.04196200	-4.95334500	-0.56556100
O	-1.02663100	2.46230900	-0.56563400
C	-1.40067500	3.25646500	0.55367900

MeTHF
H_{corr} = 0.1520
$–TS$ = -0.0367
$E_{sol(MeTHF)}$ = -271.6739
$G_{sol(MeTHF)}$ = -271.5586

H	-1.49263300	-1.04583400	1.28765700
H	-2.00333200	-1.48947500	-0.36257500
C	-1.32904900	-0.83468000	0.21361600
C	0.73439100	0.06776700	-0.40623000
C	-0.13324800	1.19539200	0.14282800
C	-1.53498900	0.64563600	-0.08904800
H	0.81819100	0.17835400	-1.50753300
C	2.12042800	-0.03420500	0.18823400
H	0.05540200	2.15826100	-0.35297800
O	0.01646300	-1.13358600	-0.12581000
H	0.05921200	1.32116900	1.22221000
H	-1.82872900	0.78660500	-1.14187300
H	-2.30615200	1.10872700	0.54284100
H	2.05958300	-0.13971900	1.28255200
H	2.71305700	0.86296300	-0.04842000
H	2.64849600	-0.91182600	-0.21179600

$SmI_2(MeTHF)_1$
H_{corr} = 0.1612
$–TS$ = -0.0594
$E_{sol(MeTHF)}$ = -24907.6991
$G_{sol(MeTHF)}$ = -24907.5973

H	0.99591500	3.41845900	-1.08944100
H	1.75690400	2.26325900	0.05858300
I	3.04872600	-0.62902900	0.25126500
I	-2.56696500	-1.44324800	0.39643300
C	0.85020400	2.84879900	-0.15548700
C	-1.49821400	2.51681100	0.12717700
C	-1.09614400	3.89448500	0.64459500
C	0.38362800	3.73911700	0.97509100
H	-1.84615600	1.87063100	0.94956400
C	-2.51089400	2.51199600	-0.99055300
H	-1.70853100	4.19491700	1.50534500
O	-0.24072300	1.91743400	-0.34525100
Sm	0.13787300	-0.49086200	-0.57420000
H	-1.23335300	4.64829000	-0.14827000
H	0.52124700	3.22611300	1.93981700
H	0.92760600	4.69261700	1.01065800
H	-2.13597400	3.07254700	-1.86089100
H	-3.44185400	2.98702200	-0.64567000
H	-2.76295400	1.48353200	-1.29018500

$SmI_2(MeTHF)_2$
H_{corr} = 0.3156
$–TS$ = -0.0760
$E_{sol(MeTHF)}$ = -25179.4008
$G_{sol(MeTHF)}$ = -25179.1613

H	-1.49325800	-4.33174500	2.57437200
C	1.22824700	2.34185200	1.70871300
O	-0.01958900	-1.80901600	1.08157900
H	1.22606000	-4.52819900	2.47436900
H	0.33780700	-4.52851900	0.92823800
C	-1.22858400	-2.34562600	1.70481800
C	-0.74187400	-3.53177900	2.53185700
C	0.55061200	-3.94256500	1.83613600
C	1.12826000	-2.59353300	1.46383600
H	-0.33833100	4.52555700	0.93452900
H	-1.22580900	4.52411800	2.48111700
H	1.49356000	4.32734000	2.57954000
Sm	0.00005600	0.00026400	-0.60557800
H	0.53350400	3.20281400	3.56989800
H	-1.25508700	-0.83147100	3.24455200
C	-1.92694500	-1.25539200	2.48178900
H	1.61978500	-2.11081400	2.32651100
H	1.83118000	-2.59660600	0.61899200
I	-2.98118500	0.14014600	-1.34909400
I	2.98139000	-0.13702000	-1.34908800
H	-0.53282200	-3.20780500	3.56504100
H	-1.61953200	2.10672300	2.33158700
H	-1.83213500	2.59398900	0.62462300
H	1.87317600	2.68020500	0.88051300
C	1.92638600	1.25076000	2.48468500
O	0.01896200	1.80612100	1.08520800
C	-1.12864800	2.59024500	1.46899200
C	-0.55073700	3.93892300	1.84208200
C	0.74207100	3.52748300	2.53683500
H	1.25459600	0.82649000	3.24730600
H	2.81283500	1.66429800	2.98928500
H	2.27724500	0.45211700	1.81284100

H	-2.81308000	-1.66965600	2.98634500
H	-2.27825700	-0.45635200	1.81065300
H	-1.87353100	-2.68355000	0.87645100

$SmI_2(MeTHF)_3$

H_{corr} = 0.4701

$-TS$ = -0.0912

$E_{sol(MeTHF)}$ = -25451.1036

$G_{sol(MeTHF)}$ = -25450.7247

H	-5.37906500	-0.88053800	-1.15775200
C	-0.11396900	-0.12857700	3.27672100
O	-2.28321100	-1.08154000	-0.09760600
H	-4.41641600	-3.43386300	-1.00043700
H	-3.62535700	-2.31205900	-2.13953200
C	-3.50262400	-0.28476100	-0.20920000
C	-4.61252600	-1.29554600	-0.48927300
C	-3.86574400	-2.48639700	-1.07887200
C	-2.59804400	-2.47743700	-0.25166500
H	-0.39014100	2.85462500	4.33361700
H	-1.75597400	2.61811000	3.23029700
H	-0.81383700	0.65536700	5.19050300
Sm	0.06462000	-0.09892300	-0.35934200
H	-2.08217500	0.43743100	3.96754000
H	-3.78497500	-0.10197800	1.92823700
C	-3.68702400	0.54269600	1.04025300
H	-2.76491100	-2.92300900	0.74605000
H	-1.72919200	-2.96960000	-0.71068500
I	-1.11759800	2.54428900	-1.54941000
I	1.35122100	-2.92146900	-0.71630700
H	-5.10663900	-1.58589700	0.45320100
H	-0.07324200	2.58380500	1.52198300
H	1.20306400	2.15773300	2.71380200
H	0.89229600	-0.16720100	3.73707600
C	-0.60469500	-1.52300800	2.98143100
O	0.01358400	0.60366600	2.04596100
C	0.15574500	1.97943200	2.41157500
C	-0.81246800	2.17652700	3.57949800
C	-1.03844500	0.75084000	4.11926200
H	-1.58617300	-1.48393400	2.48549800
H	-0.70696900	-2.08972300	3.91818500
H	0.09492900	-2.07240000	2.33060100
H	-4.59867000	1.15295000	0.95042200
H	-2.84086200	1.23118400	1.17922300
H	-3.34762100	0.38505000	-1.07056000
C	3.56738300	0.22944700	0.23179200
H	4.17358200	1.69004500	-2.07328700
H	4.68233300	3.11016600	-1.12084600
H	5.62552100	0.66036800	-0.36275800
H	4.97491500	1.74064300	0.89320800
H	2.65459300	2.99297800	0.25754000
H	1.99843400	2.59509800	-1.35496500
H	3.61186300	-0.63791700	-0.44708500
C	3.44766000	-0.25505100	1.65724800
O	2.36743800	0.97057900	-0.14180700
C	2.72603200	2.28855100	-0.59125200
C	4.15501500	2.14754300	-1.07155800
C	4.72341800	1.19157400	-0.02995700
H	3.29706300	0.59065100	2.34678900
H	4.36852100	-0.78352100	1.94782300
H	2.61588000	-0.96742100	1.76010000

$SmI_2(MeTHF)_4$

H_{corr} = 0.6244

$-TS$ = -0.1069

$E_{sol(MeTHF)}$ = -25722.8090

$G_{sol(MeTHF)}$ = -25722.2914

H	-3.95200800	2.07945100	-3.15226000
C	-2.57874800	-2.33172500	0.58403200
C	-3.22404700	-3.40246600	-0.30200500
H	-3.29499300	3.86562000	-1.10959000
H	-4.68830200	2.77810800	-0.97207800
C	-2.29451800	-3.48786800	-1.50872900
H	-3.04196200	-1.34687400	0.41014500
C	-2.57383500	-2.64281600	2.06231100
H	-4.26009200	-3.14672900	-0.56406400
H	-3.24346800	-4.36625600	0.23298300
H	-2.50862100	-2.68814300	-2.23532500
O	-1.21292200	-2.20954200	0.10092100
Sm	0.06913400	0.01044400	0.06674500
H	-2.34386500	-4.45223900	-2.03291400
H	-0.56098800	-4.13333100	-0.35544200
H	-0.18761900	-2.84629800	-1.56207300
H	-2.25751300	2.49262100	-2.80127500
C	-0.94736500	-3.23186400	-0.86686600
I	0.70775800	-0.34430900	-3.05240300
I	-0.41866000	0.80273200	3.10429300
O	1.79656900	1.89932500	-0.00596100
C	1.87271300	2.89288400	-1.06311600
C	2.88828700	3.92121100	-0.56216300
C	2.81702000	3.76777800	0.95450000
C	2.64253300	2.27070700	1.09454000
O	1.80322600	-1.73957300	0.64833000
C	2.90553200	-2.19457500	-0.18161000
C	3.44984300	-3.42682800	0.54257500
C	3.05513700	-3.17729700	1.99572100
C	1.68814500	-2.54641000	1.83021400
O	-2.25477200	0.91029700	-0.59150300
C	-2.88765700	0.67470700	-1.85421200
C	-3.17796400	2.06794100	-2.37250900
C	-3.59492300	2.80875100	-1.09890300
C	-2.91762500	2.03390000	0.04723400
C	0.48743300	3.45648900	-1.30044500
H	2.21533500	2.36847400	-1.96825800
H	3.60783000	1.74188500	0.99820000
H	2.13998400	1.95319300	2.01954200
H	3.89745300	3.66641900	-0.92111900
H	2.65159100	4.93518700	-0.91372400
H	3.71028400	4.14026600	1.47440900
H	1.93815600	4.28877800	1.36565700
C	3.89972500	-1.06453600	-0.33701100
H	2.48118200	-2.44324100	-1.16699000
H	0.90415100	-3.30801000	1.67002000
H	1.37558000	-1.88865900	2.65412500
H	2.95679600	-4.33794300	0.16905100
H	4.53232200	-3.54433100	0.39459200
H	3.02793900	-4.09116000	2.60486400
H	3.74691500	-2.46710300	2.47523200
H	-3.81536000	0.09151000	-1.70090800
H	-2.18402100	0.09957400	-2.47282800
H	-2.11266100	2.61615600	0.52197100
C	-3.87167600	1.53834700	1.11096200
H	-2.05996500	-1.85111500	2.62850800
H	-2.06967800	-3.60353700	2.25359800
H	-3.60797800	-2.71744000	2.43356200

H	0.11150300	3.97845400	-0.40637400
H	-0.21461000	2.65229200	-1.56973900
H	0.50564700	4.16357300	-2.14351800
H	-3.32512900	0.98565000	1.88875500
H	-4.64728100	0.89098200	0.67047500
H	-4.37436400	2.39290200	1.58984100
H	4.35916300	-0.80470300	0.62971200
H	3.40078500	-0.17376900	-0.74662900
H	4.69448800	-1.35624700	-1.03994500

$SmI_2(MeTHF)_5$
H_{corr} = 0.7794
$-TS$ = -0.1194
$E_{sol(MeTHF)}$ = -25994.5124
$G_{sol(MeTHF)}$ = -25993.8524

H	3.13404300	4.13050500	2.16689600
H	4.05278700	-0.13994600	2.10072300
H	4.91131600	-1.69770000	2.13902100
H	1.54814600	5.20357700	0.15416800
H	3.20097600	4.65871600	-0.18274700
H	5.43084200	0.14582000	0.12470100
H	3.08413800	-2.80039100	0.92975300
H	2.17846900	-1.66761600	2.00005300
H	5.42873900	-1.59465600	-0.21054300
H	3.36711500	0.43114000	-1.01406600
C	3.61683100	-1.45917600	-2.00787200
O	-0.21913600	-2.63083200	0.07929100
Sm	-0.00094700	-0.00224300	-0.03187800
O	2.44083500	-1.01328900	0.07684000
C	2.93299700	-1.73857500	1.20393000
C	4.24785400	-1.06281400	1.53535800
C	4.80626600	-0.75832200	0.14533100
C	3.56322500	-0.61826200	-0.75171400
I	-0.01491200	-0.00003400	3.22534200
I	0.01331500	-0.00304300	-3.22707800
O	-1.37120000	2.25616700	0.05897500
C	-1.34710600	3.14056000	1.17986600
C	-2.80917200	3.36363300	1.50773000
C	-3.43744000	3.43659900	0.11614400
C	-2.51033400	2.58997100	-0.77436900
O	-2.57688700	-0.59979100	0.07585200
C	-3.40862200	-0.30117100	1.19736200
C	-4.07100300	-1.62236600	1.52996300
C	-4.33824900	-2.19903800	0.14004900
C	-3.24174400	-1.58869100	-0.75074900
O	1.72189100	1.99979100	0.09511800
C	2.56641700	2.23985000	1.22103500
C	2.32726300	3.69693700	1.55959400
C	2.21192600	4.32770500	0.17210200
C	1.69073100	3.19328500	-0.72821000
H	-0.84873200	4.08840200	0.89923100
H	-0.77772600	2.64796300	1.98048000
H	-2.96589300	1.62348600	-1.03256200
C	-2.05753000	3.29636500	-2.03306800
H	-3.19167400	2.50349100	2.07634900
H	-2.97704700	4.26955100	2.10672700
H	-4.47328300	3.06961800	0.09537700
H	-3.45112100	4.47713800	-0.24534800
H	-4.15679900	0.46457500	0.91579000
H	-2.76226300	0.09049400	1.99532400
H	-2.46237400	-2.32398700	-0.99685100
C	-3.76500300	-0.95313700	-2.01969700
H	-3.36880900	-2.25070400	2.09723100
H	-4.98264300	-1.50167000	2.13163600
H	-4.31763900	-3.29779900	0.12113000
H	-5.32952700	-1.88332800	-0.22250500
H	3.62259800	2.05967500	0.94275900
H	2.26858600	1.53956700	2.01398200
H	0.63137600	3.33265800	-0.98624400
C	2.50189200	2.98920200	-1.98862200
H	1.38767700	3.79165100	2.12334200
C	-0.76248200	-3.32346300	1.20404800
C	0.51396000	-3.57558600	-0.74215800
C	0.75379500	-4.80161100	0.15667400
C	0.28889100	-4.35960700	1.54384400
H	1.45421900	-3.06406700	-0.99264400
C	-0.25455200	-3.88452000	-2.00781700
H	1.80400900	-5.12544000	0.13886300
H	0.14456000	-5.64754500	-0.19983100
H	1.10359500	-3.88230600	2.10786900
H	-0.10802100	-5.18493600	2.15125300
H	-1.72121900	-3.80037600	0.92324900
H	-0.93670700	-2.58279600	1.99706700
H	-1.22326300	-4.35499000	-1.77240900
H	0.31996900	-4.58898200	-2.62968500
H	-0.42563300	-2.96955600	-2.59405300
H	-4.24968600	-1.71786400	-2.64686400
H	-2.94429900	-0.50402800	-2.59844600
H	-4.51621500	-0.18006100	-1.78918000
H	-2.92982000	3.52867800	-2.66410500
H	-1.37385000	2.65942700	-2.61349800
H	-1.55483200	4.24675600	-1.78972000
H	2.10695700	2.14607000	-2.57457300
H	3.56111000	2.80273800	-1.74699300
H	2.45338400	3.89486400	-2.61348800
H	2.69154800	-1.34767200	-2.59222200
H	3.76781400	-2.52279000	-1.76060500
H	4.46156500	-1.13622800	-2.63651800

$SmI_2(MeTHF)_6$
H_{corr} = 0.9335
$-TS$ = -0.1365
$E_{sol(MeTHF)}$ = -26266.1967
$G_{sol(MeTHF)}$ = -26265.3997

H	0.32447900	5.06562800	-2.22535800
H	-4.24132000	2.58657800	1.16297300
H	-3.87378900	1.49141100	2.51427000
H	2.99259600	4.48850100	-1.66226900
H	2.00726900	5.45451000	-0.55061900
H	-2.14712500	3.81275200	1.48806700
H	-3.00989000	0.17038200	0.68193500
H	-2.52084400	1.61170700	-0.23349800
H	-2.65197900	3.52915700	3.17164100
H	-0.20918600	2.63595100	2.21713300
C	-1.09758800	1.44250600	3.76985400
O	-5.11159600	-0.48034800	-0.33616500
Sm	0.69910900	-0.02346400	0.10476000
O	-1.29912000	1.12218900	1.37029600
C	-2.60919200	1.18642700	0.78044500
C	-3.42645400	2.07842400	1.69677800
C	-2.36537800	3.03291800	2.23408900
C	-1.15773100	2.11667000	2.41538000
I	-0.94927700	0.09651700	-2.71597200
I	2.53883300	0.04980000	2.72315100

O	2.84438900	0.44857800	-1.38081600
C	2.82008300	1.08585500	-2.65791100
C	3.56617500	0.12495600	-3.56165500
C	4.67864800	-0.38979100	-2.64403500
C	4.14229200	-0.17290200	-1.21460900
O	-0.98459000	-1.92801400	0.90941200
C	-1.63493700	-2.80010100	-0.02453900
C	-3.04191400	-2.97328000	0.50960600
C	-2.79016000	-3.00754100	2.01237400
C	-1.62185800	-2.03961300	2.21038200
O	0.92473600	2.60381400	-0.12919000
C	-0.01841300	3.41279400	-0.83023200
C	0.82267600	4.16424200	-1.84177000
C	2.08784700	4.47023800	-1.03842800
C	2.14847600	3.36317000	0.03132600
H	3.33138500	2.06571800	-2.59853600
H	1.76721500	1.23368100	-2.93386100
H	3.93873000	-1.12297500	-0.70098500
C	5.04086200	0.69196500	-0.35684400
H	2.88901100	-0.68658300	-3.86529300
H	3.94638900	0.60788200	-4.47265700
H	4.92681300	-1.44358500	-2.83420600
H	5.60218900	0.19246500	-2.78677600
H	-1.09903900	-3.76724600	-0.05137500
H	-1.58058500	-2.32818100	-1.01638800
H	-1.98613500	-1.02815600	2.44923900
C	-0.61786200	-2.47794100	3.25064000
H	-3.67618500	-2.11832400	0.23256100
H	-3.51999900	-3.88840500	0.13149200
H	-3.66663400	-2.71444300	2.60754800
H	-2.49080200	-4.01957400	2.33284800
H	-0.51770400	4.10482800	-0.12561200
H	-0.76269300	2.74264200	-1.28205100
H	2.95655800	2.64550600	-0.16672800
C	2.26477100	3.88656300	1.44617800
H	1.03731900	3.50380500	-2.69474500
C	-4.94233100	-0.09114500	-1.69633600
C	-6.30673300	0.10479800	0.19557100
C	-6.87324000	0.99327100	-0.91795300
C	-5.66077200	1.24063500	-1.80965900
H	-6.00237400	0.72811800	1.05702700
C	-7.24550400	-0.98175600	0.67535600
H	-7.33319100	1.91132400	-0.52530400
H	-7.64860500	0.44339400	-1.47764900
H	-5.02833100	2.04534000	-1.39988900
H	-5.91994000	1.50492400	-2.84439200
H	-5.40130900	-0.84285700	-2.36790100
H	-3.86475500	-0.04631500	-1.91347800
O	1.67120700	-2.35683700	-0.65915900
C	1.77115400	-2.73855300	-2.03175300
C	1.15392100	-4.12108000	-2.08103800
C	1.64738600	-4.73393400	-0.77063700
C	1.84801300	-3.53258700	0.17037700
H	2.83443800	-2.76248800	-2.33692000
H	1.23498700	-1.98521500	-2.62583700
H	1.05015600	-3.47043100	0.92539100
C	3.19961000	-3.50493600	0.84889200
H	0.05785900	-4.03307000	-2.09620900
H	1.46221100	-4.69166000	-2.96824400
H	0.94694600	-5.47034500	-0.35199500
H	2.60954900	-5.24762900	-0.92551800
H	-0.24166000	-3.48922500	3.02640800
H	-1.09708300	-2.50585300	4.24198700
H	0.23961600	-1.79000900	3.30179600
H	3.31299700	-4.39322600	1.49024500
H	3.30501700	-2.60840300	1.47785700
H	4.01089800	-3.52183500	0.10280300
H	6.01853800	0.20126300	-0.22926700
H	4.59891900	0.84196300	0.63886400
H	5.21594100	1.67050700	-0.83335900
H	2.28235300	3.05486100	2.16575700
H	1.42703100	4.56207000	1.68492700
H	3.20025000	4.45718000	1.55726200
H	-0.27743000	0.71081200	3.79406100
H	-2.04532900	0.93494700	4.01024300
H	-0.89861100	2.19213500	4.55122400
H	-7.53705200	-1.63472800	-0.16216700
H	-8.15809300	-0.54438700	1.11017600
H	-6.76253400	-1.60353400	1.44367200

THP

H_{corr} = 0.1531

$-TS$ = -0.0357

$E_{sol(THP)}$ = -271.6651

$G_{sol(THP)}$ = -271.5477

H	-0.41542500	-1.24753400	1.38633900
H	-1.24018700	-2.06325700	0.04120300
H	0.58821000	1.15209100	1.46094100
H	1.46204900	1.94515300	0.14850400
H	-2.03763000	0.39148600	0.96489500
O	0.50064400	-1.17563700	-0.46992000
C	1.43775000	-0.22045000	-0.00998600
C	0.77889700	1.11184900	0.37448300
C	-1.48610900	0.11604200	0.05002400
C	-0.67393800	-1.16053600	0.31102400
C	-0.54327400	1.24923500	-0.37009000
H	-2.23815900	-0.05706600	-0.73535300
H	-0.35192700	1.18732200	-1.45397300
H	-1.00486000	2.23078900	-0.18407300
H	2.13939800	-0.07915100	-0.84640500
H	2.01342200	-0.63158300	0.84456000

$SmI_2(thp)_1$

H_{corr} = 0.1622

$-TS$ = -0.0582

$E_{sol(THP)}$ = -24907.6830

$G_{sol(THP)}$ = -24907.5790

I	-2.90878500	-0.66261700	-0.38944400
H	-0.82393200	3.65498900	1.13807300
H	-1.84907000	2.23848400	0.77018000
H	1.34807100	4.43818800	0.05366300
H	2.68169600	3.41089700	-0.48663900
H	-1.15106800	3.98872100	-1.18420400
O	0.14247500	1.83909100	0.99019500
C	1.43936800	2.48022200	0.99960100
C	1.61818200	3.40100200	-0.20627100
C	-0.73620900	2.99313400	-0.95801600
C	-0.89555100	2.73257800	0.53734000
C	0.74341700	2.91604400	-1.35601700
Sm	-0.09073100	-0.58614900	0.70095700
H	-1.32958700	2.26139700	-1.52788300
H	1.02358600	1.87750700	-1.60328900
H	0.92970600	3.50634300	-2.26443000
H	2.16465800	1.65265900	0.96851600

H	1.54662400	3.01939400	1.95638700
I	2.66217300	-1.14036200	-0.42755800

$SmI_2(thp)_2$
H_{corr} = 0.3178
$-TS$ = -0.0740
$E_{sol(THP)}$ = -25179.3808
$G_{sol(THP)}$ = -25179.1370

I	-2.88411500	-0.87794400	1.09831600
H	-1.05435200	-0.75168800	-3.49961100
H	-1.93784600	-0.71601500	-1.94386200
H	1.01902900	-2.09558800	-4.17347300
H	2.43722700	-2.40188700	-3.16369700
H	-1.43988500	-3.07036000	-3.20961900
O	0.08379500	-0.48358300	-1.80089000
C	1.30619000	-0.67970800	-2.54333700
C	1.38350700	-2.08792300	-3.13248600
C	-0.93310300	-2.61773900	-2.34199300
C	-1.04157400	-1.09871000	-2.45183000
C	0.54065000	-3.04106500	-2.29360000
Sm	0.05142600	-0.05795700	0.65798500
H	-1.46675600	-2.96323500	-1.44302900
H	0.91112900	-3.02543100	-1.25397300
H	0.65694000	-4.07734500	-2.64203800
H	2.11085000	-0.50884800	-1.81206000
H	1.36182700	0.09922300	-3.32436700
I	3.08508900	-0.32566000	1.08339000
H	-1.57253100	3.96739900	0.49458700
H	-2.29217300	2.34057400	0.65413200
H	0.02118000	4.88820800	-1.28386300
H	1.36531400	4.03175700	-2.04126200
H	-2.54984000	3.76399200	-1.65095100
O	-0.28223300	2.36762400	0.32612100
C	0.79123700	3.21051900	-0.13361800
C	0.44240900	3.88450600	-1.46066800
C	-1.87333000	2.92618000	-1.41677100
C	-1.57478400	2.94442400	0.08088100
C	-0.57066600	3.03559400	-2.22046000
H	-2.40997000	2.00159200	-1.68051600
H	-0.15099500	2.02880400	-2.38011200
H	-0.76502700	3.45838700	-3.21683200
H	1.66038600	2.54030900	-0.23037100
H	1.01975100	3.94571700	0.65688500

$SmI_2(thp)_3$
H_{corr} = 0.4733
$-TS$ = -0.0894
$E_{sol(THP)}$ = -25451.0761
$G_{sol(THP)}$ = -25450.6922

I	0.97132000	-2.82649600	-1.05158100
H	-3.30223300	-2.50471000	1.05662100
H	-1.71903600	-2.79233400	0.27245400
H	-5.26295500	-1.01347200	0.32366900
H	-5.10232500	0.61223300	-0.35455300
H	-4.03994800	-3.09841800	-1.11302100
O	-2.23666400	-0.83088500	0.49583800
C	-3.38304500	-0.00933900	0.78657900
C	-4.55128800	-0.31824000	-0.15295700
C	-3.31749200	-2.26603500	-1.12659700
C	-2.63404700	-2.18563800	0.23688600
C	-4.02312500	-0.94066700	-1.43996300
Sm	-0.00005200	0.09329000	-0.41626600
H	-2.57251300	-2.50367500	-1.90220500
H	-3.32113900	-0.23343600	-1.91393600
H	-4.83681700	-1.09541100	-2.16346600
H	-3.03268800	1.02443300	0.65537000
H	-3.65869000	-0.16470400	1.84584400
I	-1.29297100	2.93675900	-0.66627800
H	3.99924100	0.70478500	-2.21659000
H	3.04052200	-0.75478600	-1.83047500
H	4.79616400	2.59826100	-0.69004300
H	3.85337900	3.35553800	0.59551900
H	5.29957300	-0.15219000	-0.43586000
O	2.27062700	0.99721800	-1.13268300
C	2.63857400	2.36570000	-0.88820500
C	3.91207300	2.46233500	-0.04465000
C	4.24209900	-0.01437000	-0.15678600
C	3.42004700	0.19352800	-1.42748200
C	4.08025600	1.19043000	0.77840900
H	3.92238200	-0.94031200	0.34620100
H	3.18602300	1.05313700	1.40837100
H	4.94005700	1.27304900	1.45958700
H	1.77114800	2.81099200	-0.37894400
H	2.74917500	2.87669000	-1.86063600
H	2.15448100	-0.65869400	3.29945300
H	1.89407500	-1.57952700	1.78899600
H	0.44991100	0.32511300	4.92586900
H	-1.08579700	1.09327700	4.51683700
H	0.75053500	-2.38123300	4.11342700
O	0.81118400	0.13537400	1.95068200
C	0.33532300	1.10674000	2.89914600
C	-0.29523900	0.43856100	4.12057700
C	0.30210400	-1.84025800	3.26432000
C	1.39066400	-1.01347100	2.58525000
C	-0.84078800	-0.93153100	3.73468500
H	-0.07577500	-2.60010300	2.56320800
H	-1.56902100	-0.81120300	2.91727400
H	-1.37852600	-1.38925400	4.57778300
H	-0.39522300	1.71545000	2.34312300
H	1.17892000	1.76239200	3.17913200

$SmI_2(thp)_4$
H_{corr} = 0.6289
$-TS$ = -0.1027
$E_{sol(THP)}$ = -25722.7741
$G_{sol(THP)}$ = -25722.2479

I	-0.00877500	0.00429400	-3.12092400
H	-2.62844600	-3.42460400	-1.00890400
H	-1.64633100	-2.25500600	-1.94277400
H	-4.52279900	-2.96984600	0.69880100
H	-4.50232600	-1.70055900	1.92711400
H	-4.36922300	-2.15063500	-1.97091100
O	-1.76373300	-1.88735000	0.05722600
C	-2.53670800	-2.23601100	1.21851700
C	-4.03445600	-2.02275800	0.98469400
C	-3.64084600	-1.52191100	-1.43284200
C	-2.39223800	-2.35348700	-1.14256700
C	-4.23968900	-0.99623400	-0.12324300
Sm	0.00000800	0.00043700	0.08703100
H	-3.38147500	-0.68729100	-2.10230100
H	-3.73890400	-0.05614200	0.15989300
H	-5.30741500	-0.76365300	-0.25015900
H	-2.15164200	-1.59124800	2.02214800
H	-2.30997100	-3.28461400	1.48399500

I	0.00861700	-0.00333200	3.29349100
H	-3.44039300	2.62060000	-1.01246700
H	-2.27198100	1.63782400	-1.94746700
H	-2.97624100	4.50347700	0.71476700
H	-1.71202200	4.45844700	1.94790900
H	-2.15110400	4.36240600	-1.95092200
O	-1.91437800	1.73826700	0.05517400
C	-2.26616100	2.50483800	1.21958500
C	-2.03569500	4.00243600	1.00022300
C	-1.52901900	3.62418700	-1.41861500
C	-2.37013700	2.37847000	-1.14241700
C	-1.00273000	4.20674500	-0.10194300
H	-0.69447600	3.36564100	-2.08856600
H	-0.06768500	3.69594400	0.18080100
H	-0.75955900	5.27324100	-0.21904800
H	-1.63230400	2.10720100	2.02586300
H	-3.31952600	2.28650200	1.47315000
H	3.43333500	-2.61724700	-1.03824900
H	2.25769100	-1.63409000	-1.96382000
H	2.98182700	-4.50390300	0.68898100
H	1.72521500	-4.46219100	1.92999900
H	2.14023700	-4.35898700	-1.97118700
O	1.91325900	-1.73855500	0.04081700
C	2.27306200	-2.50680200	1.20162700
C	2.04259800	-4.00424200	0.98112300
C	1.52058900	-3.62235600	-1.43385900
C	2.36197500	-2.37613400	-1.16082800
C	1.00302000	-4.20764000	-0.11495700
H	0.68165600	-3.36376300	-2.09827200
H	0.06931800	-3.69810500	0.17437200
H	0.76010300	-5.27416100	-0.23236600
H	1.64391400	-2.11113600	2.01254600
H	3.32783100	-2.28800900	1.44890900
H	2.62547700	3.42553100	-1.01499500
H	1.63755100	2.25849400	-1.94596900
H	4.52902500	2.96504500	0.68234200
H	4.51321600	1.69289800	1.90776600
H	4.36043400	2.15245200	-1.98826400
O	1.76526900	1.88677700	0.05262300
C	2.54466400	2.23253900	1.21048600
C	4.04093500	2.01791000	0.96851600
C	3.63424100	1.52298700	-1.44811400
C	2.38777600	2.35485900	-1.14954200
C	4.23921400	0.99369800	-0.14282500
H	3.37090800	0.69016200	-2.11825700
H	3.73875400	0.05357100	0.14071900
H	5.30598900	0.76013200	-0.27579600
H	2.16294100	1.58688800	2.01499300
H	2.32056600	3.28097500	1.47885900

$SmI_2(thp)_5$
H_{corr} = 0.6530
$-TS$ = -0.1177
$E_{sol(THP)}$ = -25994.4608
$G_{sol(THP)}$ = -25993.7942

I	0.00715100	-0.00482600	-3.17078400
H	-4.23445300	-0.46409900	-1.01714800
H	-2.80096900	0.00033500	-1.97564400
H	-5.49947000	0.79691100	0.75091300
H	-4.84583600	1.87414000	1.98669100
H	-5.02329000	1.54608800	-1.94087700
O	-2.63407200	0.33207600	0.01835700
C	-3.39664100	0.47710700	1.22241600
C	-4.60919700	1.38649700	1.02886900
C	-4.07496000	1.68218100	-1.39535200
C	-3.45454400	0.30827200	-1.14750800
C	-4.31514900	2.40422400	-0.06462500
Sm	0.00102900	-0.00289800	0.05463700
H	-3.41986700	2.28173700	-2.04483100
H	-3.41272400	2.96982600	0.21726600
H	-5.13173500	3.13562500	-0.15698500
H	-2.69359500	0.88779800	1.96145900
H	-3.70170600	-0.52342400	1.58068500
I	-0.00773400	-0.00538600	3.26608900
H	-1.76426300	3.85421500	-0.95927400
H	-0.88649600	2.61478300	-1.89838100
H	-0.92510800	5.49182900	0.77450400
H	0.30678600	5.20887800	2.00621700
H	-0.10179100	5.20894900	-1.91262400
O	-0.50869300	2.59962900	0.09635700
C	-0.60649100	3.39666000	1.28189700
C	-0.09671600	4.82065200	1.05873600
C	0.32461600	4.35509200	-1.36075800
C	-0.79001200	3.34745500	-1.08463100
C	0.95369900	4.82228700	-0.04382700
H	1.08787900	3.90594200	-2.01335700
H	1.76474900	4.13372500	0.24175600
H	1.40807500	5.81774900	-0.15756700
H	-0.01154700	2.86300600	2.03680300
H	-1.65585900	3.39856600	1.63082800
H	-0.86665600	-4.17419800	-1.00788300
H	-0.85135700	-2.66049300	-1.95586100
H	-2.49113800	-4.98194600	0.75584200
H	-3.29783800	-4.01417200	1.99187300
H	-3.02302600	-4.28475200	-1.92890000
O	-1.11817100	-2.41271800	0.04020900
C	-1.49758300	-3.10313400	1.23640800
C	-2.75572200	-3.94813500	1.03638600
C	-2.85143900	-3.34043400	-1.38643300
C	-1.35240500	-3.18909000	-1.13276400
C	-3.61661600	-3.33311700	-0.05870200
H	-3.20852000	-2.53069400	-2.04002900
H	-3.85501700	-2.29535500	0.22345000
H	-4.57529000	-3.86405900	-0.15550900
H	-1.65013700	-2.31494300	1.98785200
H	-0.65006400	-3.72484800	1.57970600
H	3.71158900	-2.08765300	-0.95761700
H	2.28141600	-1.59440900	-1.90667200
H	3.97447600	-3.90188000	0.78311800
H	2.80073700	-4.38850900	2.00797200
H	3.15462300	-4.15949900	-1.91142400
O	1.95312600	-1.79790700	0.08619900
C	2.48906700	-2.38581500	1.27689900
C	2.90816000	-3.83994900	1.05992800
C	2.30821700	-3.71308200	-1.36392000
C	2.62537300	-2.24407000	-1.08951200
C	2.06325200	-4.45690800	-0.04657700
H	1.42888700	-3.79412600	-2.02011200
H	1.00134300	-4.37073600	0.23307800
H	2.27631700	-5.53058200	-0.15687600
H	1.68878600	-2.29769900	2.02569500
H	3.33973700	-1.77403100	1.63048200
H	3.15534500	2.86968900	-1.02712000
H	2.27785000	1.64799000	-1.99036800

H	4.90670300	2.59633700	0.75537700
H	5.00400600	1.34164300	1.99283500
H	4.98430500	1.70915900	-1.93498900
O	2.32328600	1.28500700	0.00394600
C	3.01581300	1.61603800	1.21326900
C	4.53279200	1.59554500	1.03121500
C	4.29385400	1.04084800	-1.39452000
C	2.98124600	1.78584700	-1.15732700
C	4.90302700	0.59937900	-0.05893300
H	4.12278600	0.17009400	-2.04487300
H	4.50489300	-0.38914700	0.22030600
H	5.99443800	0.48944600	-0.14285600
H	2.68505800	0.86919000	1.94967600
H	2.67041700	2.60377300	1.57034100

$SmI_2(thp)_6$
H_{corr} = 0.9404
$-TS$ = -0.1304
$E_{sol(THP)}$ = -26266.1241
$G_{sol(THP)}$ = -26265.3140

I	0.00153700	0.00032000	-3.16007800
H	1.72868400	3.60946700	-1.10852100
H	1.93370600	2.08336900	-1.99552600
H	3.26877000	4.66159000	0.74728500
H	4.04775100	3.73888300	2.03395000
H	3.90080500	3.99159700	-1.86297600
O	2.12306300	1.93437500	0.02239600
C	2.32311600	2.74753300	1.17925000
C	3.56257500	3.64170800	1.05087400
C	3.81665400	3.01888100	-1.35074900
C	2.33183500	2.68791000	-1.16821500
C	4.51767200	3.07438300	0.00926800
Sm	-0.00024900	-0.00002000	0.06572800
H	4.30517900	2.27989700	-2.00457600
H	4.82647600	2.06045800	0.31451700
H	5.43767400	3.67509300	-0.05076700
H	2.40779900	2.04404700	2.01919300
H	1.42056000	3.35504000	1.34791700
I	-0.00456000	0.00073800	3.22467600
H	3.97703000	0.31973200	-1.05878700
H	2.76241300	-0.63127500	-1.93882700
H	5.67589400	-0.47572400	0.78255500
H	5.29142300	-1.61891700	2.07073700
H	5.40766800	-1.35279400	-1.82301200
O	2.74337900	-0.87349200	0.07891400
C	3.55101100	-0.64185300	1.23326900
C	4.95167000	-1.24965800	1.09122200
C	4.52993300	-1.77767400	-1.30845500
C	3.49087700	-0.66818000	-1.11615500
C	4.94134900	-2.35811800	0.04707600
H	4.13985900	-2.57167700	-1.96381200
H	4.22594900	-3.13951100	0.35422600
H	5.92542800	-2.84586700	-0.02030600
H	2.99391200	-1.08426700	2.07094300
H	3.61368700	0.44247200	1.41564900
H	-3.99029400	-0.31453300	-1.11286000
H	-2.77057600	0.62171900	-2.00383700
H	-5.66908800	0.49719900	0.73733800
H	-5.26343800	1.63811300	2.02087000
H	-5.40553600	1.37337000	-1.87597600
O	-2.73599200	0.86886600	0.01291600
C	-3.53949900	0.64155900	1.17156600
C	-4.93474300	1.26415100	1.03934300
C	-4.52164800	1.78814100	-1.36377300
C	-3.49318800	0.66805800	-1.17670600
C	-4.92172200	2.37156400	-0.00586800
H	-4.12481200	2.57863700	-2.01941800
H	-4.19897400	3.14765700	0.29727800
H	-5.90242200	2.86674400	-0.06811100
H	-2.97330300	1.07404400	2.00818200
H	-3.61143500	-0.44275500	1.34803200
H	-1.71138100	-3.60057600	-1.05696000
H	-1.92921100	-2.07072300	-1.93217200
H	-3.25442900	-4.67896900	0.78513500
H	-4.04954600	-3.77369000	2.07449600
H	-3.87563300	-4.00356900	-1.81831100
O	-2.12758800	-1.93955000	0.08625900
C	-2.33026000	-2.75884900	1.23774800
C	-3.55972300	-3.66433400	1.09503400
C	-3.80484500	-3.03107700	-1.30358400
C	-2.32443900	-2.68583500	-1.11104300
C	-4.51338600	-3.09772400	0.05174400
H	-4.29756000	-2.29624900	-1.95882500
H	-4.83055600	-2.08733700	0.36030300
H	-5.42893300	-3.70432700	-0.01657800
H	-2.43033600	-2.05839600	2.07865400
H	-1.42392800	-3.35897500	1.41520700
H	2.26238200	-3.29350500	-1.12434800
H	0.83797800	-2.70414600	-2.00836800
H	2.41084400	-5.15523900	0.71603200
H	1.22793400	-5.38361300	2.00533100
H	1.50628100	-5.36065100	-1.89361900
O	0.61377700	-2.80391000	0.00895600
C	1.21696600	-3.38939200	1.16368600
C	1.38023600	-4.90776500	1.02468700
C	0.70748400	-4.80568100	-1.37442100
C	1.16269500	-3.35526700	-1.18436400
C	0.41097900	-5.44968800	-0.01727400
H	-0.17930400	-4.85557700	-2.02508500
H	-0.62168500	-5.21610400	0.29206400
H	0.47551900	-6.54616900	-0.08364000
H	0.56059800	-3.12187200	2.00342600
H	2.19080600	-2.90729000	1.33998500
H	-2.25963000	3.27889100	-1.07426100
H	-0.82528100	2.69857800	-1.94590500
H	-2.43161800	5.15129200	0.75325400
H	-1.25891100	5.40087300	2.04811200
H	-1.52192300	5.34943200	-1.84730700
O	-0.61415100	2.81138500	0.07240800
C	-1.22229900	3.40106400	1.22198500
C	-1.40055100	4.91632300	1.07015700
C	-0.71831300	4.80484500	-1.32448600
C	-1.16074600	3.35134500	-1.12730300
C	-0.43036200	5.45885700	0.02942600
H	0.16852400	4.86052200	-1.97449000
H	0.60266800	5.23358200	0.34387200
H	-0.50180900	6.55447500	-0.04371600
H	-0.56137100	3.14828600	2.06284900
H	-2.19151000	2.91167300	1.40621800

HMPA
H_{corr} = 0.2709
$-TS$ = -0.0564
$E_{sol(THF)}$ = -820.7999

$G_{sol(THF)}$ = -820.5854

H	-2.09343700	1.70043800	0.04257300
C	-1.20980200	2.34365600	-0.04782300
H	1.81914100	-1.63814200	-1.58759700
H	3.19619800	-1.56167900	-0.44219800
H	-1.17455900	0.24762500	2.07091000
H	-2.75346600	-0.52773600	1.80067400
H	2.08878100	1.70630500	0.04327200
H	-1.84307000	-2.70553900	-0.14174800
P	0.00008000	-0.01685200	-0.49346600
O	0.00069000	-0.23945600	-1.98256300
H	-1.31959500	-1.53699700	2.12759600
H	1.85143800	-2.70024600	-0.14124400
H	-1.81404100	-1.64335900	-1.58809200
N	-1.41881100	-0.64469100	0.19225800
C	-2.09851200	-1.69992500	-0.53015200
H	1.17311900	0.25062900	2.07139300
H	1.32259500	-1.53364200	2.12794100
C	-1.66904400	-0.61968400	1.61439500
N	-0.00214600	1.55751400	0.14626600
C	1.20336100	2.34692600	-0.04807400
N	1.42042500	-0.64084700	0.19274100
C	2.10366500	-1.69381200	-0.52962400
H	1.22550400	2.83191200	-1.04211600
H	-1.23294400	2.82944400	-1.04145300
C	1.66996200	-0.61539600	1.61499500
H	2.75406800	-0.52075200	1.80173700
H	-1.26210200	3.13149800	0.72172400
H	1.25306400	3.13555700	0.72082500
H	-3.19146600	-1.57130600	-0.44285900

$SmI_2(HMPA)_1$
H_{corr} = 0.2803
$-TS$ = -0.0774
$E_{sol(THF)}$ = -25456.8427
$G_{sol(THF)}$ = -25456.6398

H	0.94246100	0.76119400	-1.99119700
C	0.95251800	-0.29625100	-2.28741000
H	2.32949700	-1.67896500	2.56157600
H	3.62891800	-2.87808600	2.29984500
H	3.60076700	0.48444600	-2.12974200
H	3.26579400	2.22601900	-1.95393700
H	2.25163600	-3.05828400	-0.83129200
H	3.79924100	2.36283200	1.06610300
P	2.19603000	-0.44730100	0.09847700
O	1.08584800	-0.27586300	1.16120200
H	4.68700200	1.50631900	-1.14138000
H	4.04552200	-1.18292600	2.68725500
Sm	-1.19470700	0.19428700	0.93600300
N	2.80630200	1.06127700	-0.27352600
C	2.77896100	2.12413200	0.71538700
H	4.46818500	-1.52881900	-1.03003200
H	5.33853400	-0.95816900	0.42719000
C	3.63515800	1.32816300	-1.43009300
I	-2.32224300	-2.30482700	-0.38522500
I	-1.00288000	2.93747200	-0.37459500
N	3.30833000	-1.54327100	0.72112300
C	3.33052200	-1.82859600	2.14295300
H	0.60307400	-2.83628500	-1.49017100
H	-0.08536200	-0.67275700	-2.27706800
C	4.58681900	-1.67820100	0.05168300
H	4.97926200	-2.69587500	0.20834300
H	1.33756100	-0.37319900	-3.31761700
H	2.02471800	-2.78486700	-2.58504000
H	2.32131700	3.02789500	0.28284500
H	2.16781800	1.81890600	1.57184800
N	1.78897600	-1.08348300	-1.38513400
C	1.65683800	-2.52267600	-1.57973100

$SmI_2(HMPA)_2$
H_{corr} = 0.5536
$-TS$ = -0.1116
$E_{sol(THF)}$ = -26277.6834
$G_{sol(THF)}$ = -26277.2415

H	5.26510500	2.24995500	2.24127300
H	2.94618200	-3.60114700	0.32667600
H	6.56649000	0.12194700	-0.89538100
H	3.55625100	2.42167800	1.72326900
H	-4.45636200	-2.71559600	-0.58755100
H	-3.40319700	-3.51798500	-1.78065800
H	4.59379300	-0.29000100	4.05923800
H	4.18727200	-0.76285300	-2.49349500
P	-2.49032900	-1.38821800	0.89073900
O	-1.08321500	-0.77375100	1.01337600
H	3.93897000	2.10663600	3.43479200
O	2.24533700	0.88498200	0.38549700
Sm	-0.03046700	0.92223200	-0.32604400
N	-2.41284100	-2.72323700	-0.11401500
C	-1.18394900	-3.48331600	-0.22522900
P	3.41610400	-0.04562700	0.72711700
N	3.02098500	-1.64786100	1.05787300
C	-3.57326000	-3.35539100	-0.70261100
I	-1.78117200	3.43745400	0.14029400
I	0.21527500	-0.89567000	-2.85898400
N	-3.02687700	-1.71715500	2.45359400
C	-2.08477900	-1.71782500	3.55432100
H	4.82951400	-1.43488400	2.70710400
H	6.00043400	-0.10568000	2.97819800
C	-4.16702500	-2.59371200	2.62009200
C	2.02590700	-1.86286400	2.10278600
N	4.51223100	-0.01994900	-0.53139600
H	6.13704700	-0.52456300	0.70278900
H	5.97238200	-1.55520500	-0.74743800
C	2.90235900	-2.57222300	-0.06348300
N	-3.71790800	-0.48095200	0.22529200
C	-4.47363400	0.48073500	1.01899100
H	2.16460800	-1.12607600	2.90588700
H	0.99126600	-1.77030100	1.72731200
C	-3.64360200	-0.13708700	-1.19242000
H	-1.27047200	-1.01771200	3.33694400
H	-2.59778200	-1.39197500	4.47422500
C	5.85496600	-0.52618100	-0.35736100
H	-1.26951100	-4.46149200	0.28382400
H	-3.78250400	-4.33553400	-0.23570200
H	-1.65368100	-2.72090700	3.73983000
H	-4.87454300	-2.45895200	1.79069800
H	-3.87921700	-3.66175500	2.66668100
H	3.03845300	0.47482700	-1.94941500
H	4.70753500	0.94810400	-2.38455800
H	-4.69520700	-2.34081700	3.55379800
C	4.08973600	0.16863500	-1.90823000
H	-5.52589600	0.46951100	0.69006500
H	1.96453500	-2.44237700	-0.63390100
H	3.74231500	-2.43656700	-0.75619800

H	2.16173900	-2.86933700	2.53048700
H	-4.42891200	0.20701600	2.07925400
H	-4.06768500	1.49890000	0.89395700
H	-3.33915800	0.91721300	-1.31499700
N	4.13183000	0.45676700	2.15079700
C	4.23437000	1.88278000	2.39623000
C	4.93102700	-0.38626100	3.01236300
H	-2.91632000	-0.77555400	-1.71286500
H	-4.63207200	-0.26906700	-1.66331200
H	-0.94057300	-3.64600200	-1.28716900
H	-0.35753100	-2.91562200	0.21506200

$SmI_2(HMPA)_3$
H_{corr} = 0.8264
$-TS$ = -0.1447
$E_{sol(THF)}$ = -27098.5234
$G_{sol(THF)}$ = -27097.8417

H	-0.88584100	4.88559500	-0.53360500
H	0.57297500	5.26558400	-1.47466800
C	1.97860000	3.10175600	-0.75830500
H	3.67220600	0.94772500	-2.51669900
N	-1.40778300	3.44937200	1.44947700
C	-2.53141900	2.93497700	0.68861800
H	5.70788500	2.34747200	0.25482100
C	-0.03403200	4.44225800	-1.06289100
P	-3.81036100	-0.97393200	0.21426300
O	-2.29126300	-0.87517800	0.25229600
H	-2.83213500	3.61933500	-0.12563500
H	2.54319300	2.50184400	-0.03254400
Sm	0.01264800	-0.59811200	-0.41708700
N	-4.25344400	-2.59095200	0.28078700
C	-3.37292000	-3.54535700	0.93342900
H	-0.75175500	4.97306100	2.73042400
H	-1.81222900	5.54409800	1.40931400
C	-5.62223900	-3.03167200	0.12397400
I	-0.99182200	1.06356400	-2.97735400
I	0.70906000	-3.28775800	1.20773500
N	-4.41090100	-0.01573300	1.47755200
C	-3.55189200	0.17047600	2.63489500
H	5.82168100	0.75064700	1.05249900
H	6.78158400	1.09352800	-0.42129200
C	-5.82212200	-0.09088900	1.79714900
N	1.13321300	3.24193900	2.43497900
N	4.56128700	-1.89328500	-1.15522100
C	1.03425700	2.45587100	3.64806800
H	1.71386700	2.45170900	-1.60961300
H	2.63079800	3.91630700	-1.11643300
N	-4.65384700	-0.41345900	-1.12012500
C	-4.81282500	1.02194300	-1.32015800
H	-2.27063900	1.96643700	0.24485100
H	-3.39484000	2.78548100	1.35700500
C	-4.45372100	-1.12894800	-2.37862700
H	-2.50827700	0.26060700	2.31179300
H	-3.84492400	1.09935200	3.15212600
H	-0.40849000	3.81963900	-1.89392100
H	-3.73530100	-3.80452800	1.94562700
H	-6.10324500	-3.25022600	1.09600900
H	-3.63085800	-0.66228700	3.36008000
H	-6.41367300	-0.19977800	0.87833900
H	-6.06355300	-0.93281700	2.47463200
H	1.86038000	4.99925900	1.57908400
H	1.47399000	5.13942500	3.32396200
H	-6.13657100	0.84188700	2.29415700
H	2.04277500	2.21939500	4.02710500
H	-5.71974400	1.19655300	-1.92189200
C	1.88209700	4.47212400	2.54065000
H	0.48519100	2.99332200	4.44377500
H	0.52031300	1.51123200	3.43051700
H	-4.93088300	1.52450300	-0.35324200
H	-3.94547300	1.45084600	-1.85239800
H	-3.62060700	-0.68994000	-2.95411200
H	2.93329400	4.25605800	2.80038400
C	-1.62227700	4.71192700	2.11514200
H	-2.49732300	4.63352500	2.78248900
H	-4.22940100	-2.18419700	-2.17956000
H	-5.37715300	-1.07146300	-2.97794800
H	-3.32753700	-4.47271400	0.33852300
H	-2.35437100	-3.14409500	1.01275400
H	3.65938500	0.26346600	3.05822800
N	0.79021700	3.67321100	-0.14292700
H	5.02242500	-1.86040400	2.78685300
H	6.16487300	-2.28052400	-2.44418800
H	-6.21172800	-2.26799200	-0.39873800
H	-5.64830000	-3.95659900	-0.47785100
H	4.26647900	-3.96359600	-0.82405000
H	4.53947300	2.41875100	-2.00577400
O	2.39411100	-0.44189500	-0.85983900
P	3.82666800	-0.61848500	-0.36491100
N	3.99964400	-0.81800900	1.28994500
C	3.11364700	-0.05401500	2.15286700
H	6.43351300	-0.95651500	-1.28518200
H	6.50096900	-2.65362600	-0.73158200
C	4.45070800	-2.07303200	1.86760400
H	2.74546200	0.84214800	1.63667000
H	2.23849800	-0.65730400	2.45330500
C	5.98040100	-1.94824600	-1.40802300
H	2.78446800	-3.01545600	-1.06553900
H	3.84624200	-3.37732500	-2.46501500
C	3.82749700	-3.12351100	-1.39130000
H	3.60042000	-2.73295700	2.11277200
H	5.11416400	-2.59719600	1.16915700
N	4.69793400	0.77949000	-0.70299700
C	4.59114800	1.31809300	-2.04432700
O	0.06009200	1.32516300	1.03660200
P	0.12767800	2.84672200	1.15261900
C	5.80923900	1.26154600	0.08191100
H	5.45812600	1.03886000	-2.67294400

$SmI_2(HMPA)_4$
H_{corr} = 1.0996
$-TS$ = -0.1743
$E_{sol(THF)}$ = -27919.3678
$G_{sol(THF)}$ = -27918.4425

H	-2.99618100	3.93109100	2.76551700
H	-4.32741400	3.00714500	3.49076300
C	-4.74372600	1.33631000	1.51304200
H	-1.86964800	-1.94227700	2.52115000
N	-1.85922900	3.96916200	0.44179100
C	-0.68604200	3.90360100	1.29499500
H	-5.46105500	-1.59282700	1.74042900
C	-3.46625500	2.94049600	2.80765600
P	2.64886900	2.67333300	-0.44032900
O	1.29261000	2.01296000	-0.62601700
H	-0.85028800	4.43931000	2.24848600

H	-5.12315800	1.11483700	0.50583400
Sm	0.00717400	-0.02983700	-0.15572500
N	3.78031700	1.91595500	-1.43778200
C	3.31035600	1.39150000	-2.71103800
H	-3.15233300	5.27633600	-0.57044200
H	-2.73694000	5.80188500	1.08904200
C	5.17086800	2.31651200	-1.43468800
I	0.19371100	0.43432700	3.14807600
I	-0.36973100	-0.37954600	-3.39364600
N	2.44311600	4.32872900	-0.74331900
C	1.39107600	4.71421000	-1.66711400
H	-5.34762700	-2.64596900	0.30257300
H	-5.29567100	-3.35442500	1.94989700
C	3.58394700	5.21930300	-0.73459300
N	-3.80527400	2.85453100	-1.12357500
N	-2.35584200	-4.47662600	0.03166000
C	-3.24097700	2.57650400	-2.43251200
H	-4.15285300	0.46887900	1.85857300
H	-5.60701000	1.47435300	2.18328000
N	3.40533800	2.66083900	1.05594400
C	2.87064700	3.49710200	2.12492200
H	-0.43411900	2.86149700	1.53092200
H	0.17500400	4.35814600	0.78176800
C	3.89885800	1.38107600	1.55874000
H	0.57301000	3.98554900	-1.61422600
H	1.00639300	5.70968600	-1.38569300
H	-2.73800500	2.21886800	3.22336000
H	3.44215700	2.12357500	-3.53031700
H	5.39066000	3.08911300	-2.19627000
H	1.75084900	4.77190900	-2.71243900
H	4.33244000	4.86889700	-0.01195000
H	4.06465400	5.30492200	-1.72826300
H	-5.36510100	3.77324100	-0.08143800
H	-4.98783300	4.53066800	-1.66229200
H	3.26288300	6.22983600	-0.42953500
H	-3.96560700	2.00790300	-3.03877800
H	3.68850000	3.74933200	2.81997300
C	-5.05962800	3.57081500	-1.11550600
H	-2.99362200	3.50796700	-2.97445900
H	-2.33315500	1.96445400	-2.34146300
H	2.46322900	4.42704100	1.71145300
H	2.08015400	2.96955500	2.68736600
H	3.13684700	0.88764600	2.18593700
H	-5.84541400	2.96894600	-1.60575500
C	-2.35182000	5.30038900	0.17925000
H	-1.53216500	5.91908400	-0.22349100
H	4.14327300	0.71435500	0.72597400
H	4.80567900	1.55552200	2.16214000
H	3.88465400	0.48645100	-2.96836600
H	2.25052200	1.11231800	-2.64962800
H	-4.90210600	-0.95695400	-1.84576600
N	-3.95686200	2.56183300	1.48714400
H	-4.21110000	-3.14828800	-3.12045500
H	-3.24873000	-6.22508800	0.76221400
H	5.45424600	2.70285400	-0.44732100
H	5.80600200	1.44172200	-1.65617000
H	-1.20771200	-5.52111200	-1.41860000
H	-3.40904900	-1.35790300	3.16810500
O	-1.17629300	-2.17967600	0.19576400
P	-2.55844200	-2.80504800	0.04440200
N	-3.49400800	-2.42393500	-1.29405300
C	-3.85922000	-1.02425700	-1.48898700
H	-4.36499900	-4.86744600	0.49877800
H	-3.74950600	-5.79042300	-0.89638300
C	-3.27002800	-3.13276000	-2.54592600
H	-3.76485000	-0.47202700	-0.54691600
H	-3.18955200	-0.54886900	-2.22503200
C	-3.48148800	-5.37661000	0.09566000
H	-0.33720300	-4.28644900	-0.45188200
H	-0.79037000	-5.85083100	0.28866500
C	-1.11186700	-5.06233400	-0.41482300
H	-2.48891900	-2.64300300	-3.15348200
H	-2.97552500	-4.17131800	-2.34932600
N	-3.54495700	-2.32991200	1.31658600
C	-2.93299600	-2.19079500	2.62632200
O	-1.99054700	1.36775300	0.06154800
P	-2.83323100	2.62265500	0.21625900
C	-4.98060600	-2.49596500	1.32552300
H	-3.04399000	-3.10921800	3.23319100
P	2.82713200	-2.45682500	0.32027900
O	2.22806500	-1.15609600	-0.19766600
N	2.19587300	-3.74604600	-0.57537400
C	1.93227800	-3.49985000	-1.98607000
C	2.59972800	-5.10648200	-0.28541000
N	4.51139400	-2.31264200	0.23399700
C	5.07259300	-1.42927400	-0.76792400
C	5.37365900	-3.40869600	0.61894800
N	2.58141300	-2.94289500	1.90140500
C	3.27536800	-2.24233300	2.97614500
C	1.25707700	-3.40993500	2.30161300
H	4.34232500	-0.64789800	-1.01105100
H	2.82088700	-3.70328700	-2.61457700
H	3.50503200	-5.40920600	-0.84606100
H	5.34561200	-1.96880300	-1.69561800
H	4.88243000	-4.02873900	1.37968100
H	5.64793400	-4.05217100	-0.23906000
H	6.30794300	-3.01280100	1.05313600
H	3.50769700	-2.95775500	3.78289900
H	4.21411000	-1.81501400	2.60372300
H	2.64701400	-1.43268100	3.38591900
H	0.69951500	-2.59982300	2.80101600
H	0.68060700	-3.72770100	1.42678800
H	1.36278300	-4.25958100	2.99778200
H	1.11915000	-4.16162400	-2.32410800
H	1.60677000	-2.46409100	-2.14620700
H	2.79266000	-5.22746400	0.78811600
H	1.78767200	-5.79715000	-0.56844700
H	5.98643400	-0.95347800	-0.37153400

$SmI_2(HMPA)_5$
H_{corr} = 1.3734
$-TS$ = -0.2023
$E_{sol(THF)}$ = -28740.1751
$G_{sol(THF)}$ = -28739.0040

H	2.21628800	-3.55392500	-3.78615900
H	1.20449200	-5.01394700	-3.84780000
C	0.75066800	-5.26561900	-1.28066700
H	-1.95172700	-2.57786000	-2.15940000
N	3.49708400	-2.37976400	-1.92516300
C	3.23112500	-1.20985900	-2.73591300
H	-2.21490900	-6.13146400	-1.41146500
C	1.38897600	-4.08039500	-3.29372500
P	3.92806600	0.76591600	0.82844300
O	2.56988900	0.13389600	0.60512000

H	3.12028300	-1.46377600	-3.80675400
H	1.08694800	-5.56020200	-0.27882400
Sm	-0.04898400	0.05072000	0.09923900
N	3.85415300	1.85703600	2.10871700
C	2.77689400	1.80831500	3.07791000
H	4.86239600	-3.94676500	-1.60861200
H	4.46101000	-3.66818900	-3.32722000
C	4.98255700	2.67210000	2.48721100
I	-0.46822800	0.07692400	-3.22302100
I	-0.46737000	-0.24212300	3.39073800
N	5.23919100	-0.22632700	1.22511400
C	5.79724200	-1.13631600	0.24793200
H	-2.75829200	-5.87794600	0.26242900
H	-3.91569100	-5.69974100	-1.08759100
C	5.54804300	-0.55943100	2.59821900
N	2.95607400	-3.84049300	0.33738400
N	-3.95635600	-3.07925500	1.13590800
C	3.04440600	-3.10700800	1.58404100
H	-0.22679900	-4.76566600	-1.17562900
H	0.62619000	-6.18082200	-1.88172400
N	4.50447600	1.47511900	-0.58764700
C	5.86264700	1.93446100	-0.76243400
H	2.30369200	-0.72340500	-2.41192600
H	4.06605400	-0.49710600	-2.63423600
C	3.57458700	2.01622700	-1.56036400
H	5.46298400	-0.85256600	-0.75621300
H	6.90118400	-1.10282000	0.28049100
H	0.49118700	-3.43932400	-3.35953200
H	3.14214400	1.47133500	4.06617500
H	5.47875400	2.29835700	3.40375700
H	5.47661700	-2.17711800	0.42665100
H	4.97812900	0.07572600	3.28602800
H	5.29902400	-1.60992700	2.82838000
H	3.48232600	-5.60954200	-0.64824200
H	4.67472800	-5.05052800	0.56702300
H	6.62520500	-0.41546000	2.79785600
H	2.49127800	-3.62629900	2.38460700
H	5.93872400	3.03660100	-0.68699300
C	3.59577200	-5.13354600	0.33360200
H	4.09746200	-3.02416300	1.90442400
H	2.63144900	-2.09675000	1.46484700
H	6.52338300	1.48263300	-0.01226400
H	6.23052000	1.64659400	-1.76359100
H	3.88106800	1.71513900	-2.57607000
H	3.14355900	-5.79347200	1.09724500
C	4.62540200	-3.17310800	-2.34978200
H	5.51270200	-2.52510000	-2.44962300
H	2.56351100	1.64465700	-1.36844600
H	3.54496400	3.11824400	-1.51907200
H	2.33132800	2.81053300	3.20214500
H	1.98709000	1.12093600	2.75318700
H	0.34762800	-4.79112300	1.86947000
N	1.74995800	-4.41839400	-1.92011700
H	-1.74881700	-5.83824300	2.72010500
H	-5.76049900	-2.44075800	0.24717100
H	5.73234900	2.71229200	1.68832000
H	4.65054000	3.70530400	2.69011000
H	-4.63466400	-3.03385700	3.14076000
H	-1.84980100	-4.21729800	-2.84725600
O	-1.72639100	-1.85143900	0.14659300
P	-2.39551800	-3.16122600	0.51760200
N	-1.59248500	-3.99544200	1.72252100

C	-0.16687800	-3.81244100	1.88974200
H	-4.90239200	-3.74124300	-0.61223900
H	-5.78268500	-4.10358900	0.89882000
C	-2.21580100	-4.83582900	2.71549100
H	0.22707800	-3.18890100	1.07927300
H	0.04852500	-3.30174100	2.84291500
C	-5.15418700	-3.35432800	0.38257400
H	-3.18478800	-2.03412800	2.79236400
H	-4.77905600	-1.49361700	2.23379700
C	-4.14972300	-2.38129600	2.39222300
H	-2.10085600	-4.40458900	3.72652300
H	-3.28572300	-4.95304600	2.50591200
N	-2.48959900	-4.13034300	-0.85971600
C	-2.44343500	-3.55947600	-2.18974000
O	1.22976300	-2.07491200	-0.60534500
P	2.28148200	-3.08948300	-1.00206600
C	-2.87093600	-5.51793800	-0.76891600
H	-3.44861900	-3.45277600	-2.64147500
P	-3.75194500	1.61101900	-0.14298700
O	-2.25902900	1.64856500	0.14487100
N	-4.54843800	0.80202700	1.12595800
C	-4.03033100	1.12127500	2.45118000
C	-5.99607200	0.73405900	1.08645000
N	-4.36616500	3.18751800	-0.33750700
C	-3.84271100	4.21824300	0.53178400
C	-5.73001000	3.43535500	-0.75779100
N	-4.34931800	0.85825900	-1.51592700
C	-4.18387000	1.48968400	-2.82001600
C	-4.23770100	-0.59093500	-1.58435900
H	-2.80824300	3.97365600	0.79732600
H	-4.37471900	2.11283500	2.80738700
H	-6.48125800	1.66506500	1.43934300
H	-4.44694800	4.33855400	1.45235900
H	-6.12725800	2.57787600	-1.31286100
H	-6.39881200	3.63731700	0.10036700
H	-5.76045500	4.32009700	-1.41863700
H	-5.04403200	1.22356600	-3.45816600
H	-4.15031700	2.58039000	-2.71746500
H	-3.25455100	1.14909600	-3.30849300
H	-3.34420900	-0.87842400	-2.15934600
H	-4.14425200	-1.00428700	-0.57459100
H	-5.13563700	-1.00814600	-2.07320900
H	-4.38500300	0.36819900	3.17035700
H	-2.93366900	1.08621500	2.45084000
H	-6.34050800	0.52240700	0.06573600
H	-6.34090400	-0.08295300	1.74238100
H	-3.84380000	5.18692000	0.00132900
P	0.53429500	3.91275100	-0.34857600
O	0.86889200	2.49118400	0.07751900
N	-0.58360100	4.56223400	0.74654700
C	-0.59675300	3.99714700	2.08831800
N	-0.10264000	4.22704800	-1.86638200
C	0.74304100	3.92786600	-3.02053400
H	0.46246700	4.60003100	-3.84862400
H	0.61019800	2.88227900	-3.34937500
H	-1.58061400	4.18591500	2.54801100
H	-0.44154100	2.91316400	2.05182500
C	-0.81718600	5.99000200	0.70803200
C	2.00348900	6.16653700	-1.00024800
C	-1.50379200	3.93535200	-2.13325800
H	0.17342800	4.45097500	2.74165700
H	-0.04694600	6.56660300	1.25703500

H	1.19120000	6.29042200	-1.72582500
H	1.92482400	6.96859100	-0.24172000
H	2.96462500	6.30041500	-1.52832200
H	-1.63088700	2.89847600	-2.48382500
H	-2.09732600	4.07416300	-1.22813300
H	-1.86565100	4.62654500	-2.91343400
H	-0.84725300	6.34284500	-0.33177900
H	-1.79253000	6.21457800	1.17012800
N	1.95302200	4.84769000	-0.40717000
C	2.92485300	4.64150500	0.64221500
H	2.85122500	3.61469900	1.01898100
H	2.79229200	5.35384700	1.48098200
H	3.94462500	4.78566100	0.24230500
H	1.79858000	4.09874900	-2.77780800

TPPA
H_{corr} = 0.3850
$-TS$ = -0.0621
$E_{sol(THF)}$ = -1052.9733
$G_{sol(THF)}$ = -1052.6504

H	-1.23903700	-2.16730700	1.86514600
H	-3.13340200	-1.01226600	0.92089000
H	-3.63283400	-2.65800600	0.49437100
H	-2.92013000	-0.49874100	-1.32748200
H	-3.23313900	-2.17231700	-1.81473000
H	-0.66847600	-0.98417600	-2.02609000
H	-0.94059400	-2.72659800	-1.85726700
H	2.20024800	1.83781400	-0.92861300
P	0.17027200	-0.30627200	0.52633900
O	0.34885400	-0.35842700	2.02223400
H	2.05053700	1.46475500	0.80704500
H	4.39928300	1.09539800	0.59972400
H	0.10291300	1.48237600	-1.77503200
N	-0.50297100	-1.75330400	-0.05972700
C	-1.47179800	-2.40509500	0.81963800
C	-2.84448500	-1.90555500	0.34639100
C	-2.63418400	-1.54381300	-1.13939000
C	-1.12511800	-1.74764500	-1.38147900
H	-1.67477900	1.37820200	-1.78019500
H	-1.38465500	-3.49718000	0.68122800
N	-0.74444000	1.04584700	0.10894700
C	-1.37627200	1.86012000	1.15369400
C	-1.85637400	3.10707200	0.40487500
C	-0.94060500	3.17760600	-0.81437300
C	-0.79780000	1.70758000	-1.18693700
H	4.26460800	0.67971900	-1.12199000
H	3.42800400	-1.08647900	1.24431400
H	4.53487100	-1.52912100	-0.09013900
H	1.90596200	-2.21627900	-0.31213100
H	2.67317000	-1.42269700	-1.70845400
N	1.52102800	-0.14162600	-0.48501700
C	2.34330400	1.04941000	-0.17135400
C	3.79181100	0.54819200	-0.13487800
C	3.64597800	-0.93798000	0.17423000
C	2.41372300	-1.30098800	-0.64076000
H	-2.21286500	1.33042800	1.63863600
H	-0.64763500	2.09774800	1.94462300
H	-1.82291100	4.00839200	1.03296900
H	-2.89919600	2.97184900	0.07379300
H	0.04476700	3.58252500	-0.52993300
H	-1.34401900	3.79361300	-1.63099200

$SmI_2(TPPA)_1$
H_{corr} = 0.3943
$-TS$ = -0.0824
$E_{sol(THF)}$ = -25689.0196
$G_{sol(THF)}$ = -25688.7077

H	0.51970800	2.58385300	-1.45744200
H	0.57272500	3.69439500	0.65364400
H	1.34151100	4.99416900	-0.26173000
H	2.55948500	3.22072000	1.76958500
H	3.36124500	4.57070600	0.95161600
H	4.12450700	1.92885100	0.45064500
H	4.18046100	3.17484000	-0.80973500
H	3.83273400	-2.26163800	0.46783900
P	1.90744600	0.49837700	-0.31249100
O	0.58595100	0.20529200	-1.05489700
H	2.06574600	-2.04730500	0.42371800
H	1.98416400	-3.28953600	-1.56995600
Sm	-1.58746700	-0.49723500	-0.76490600
N	2.47097700	1.97692500	-0.87980400
C	1.49106100	3.02294700	-1.20119800
C	1.44557100	3.93998800	0.03087700
C	2.76506300	3.66264900	0.78297800
C	3.51237600	2.65480700	-0.10236100
I	-1.01834300	-3.20489400	0.57464500
I	-2.86071600	2.21685000	-0.13365300
N	1.63224400	0.38611900	1.32522100
C	0.28645100	0.52610000	1.90002900
C	0.55338500	0.69390900	3.39126800
C	1.82611000	-0.12031500	3.59853500
C	2.65150300	0.23366800	2.36771300
H	3.74160100	-3.50512800	-1.48912800
H	2.16345000	-1.37237400	-2.97370200
H	3.71481800	-2.10834800	-3.44425600
H	3.71461400	0.41221800	-2.38168200
H	4.90594600	-0.75722700	-1.75268200
N	3.19855500	-0.53233500	-0.55870800
C	2.98870800	-1.94260300	-0.16454800
C	2.93876000	-2.75343000	-1.47653200
C	3.13678700	-1.71977400	-2.59364100
C	3.82789300	-0.55959100	-1.88551500
H	-0.26938300	1.37569600	1.47377200
H	-0.28907300	-0.40814500	1.73705300
H	-0.29405900	0.35405700	4.00108700
H	0.73672700	1.75570400	3.62342700
H	1.58902500	-1.19624400	3.59021900
H	2.35136400	0.11122700	4.53537600
H	3.38154800	-0.54214300	2.10020700
H	3.20507800	1.17667300	2.53207000
H	1.86541700	3.56682100	-2.08438100

$SmI_2(TPPA)_2$
H_{corr} = 0.7814
$-TS$ = -0.1222
$E_{sol(THF)}$ = -26742.0450
$G_{sol(THF)}$ = -26741.3858

C	-4.06672800	2.71171700	1.33433900
C	-5.35154700	3.16582500	2.01959500
C	-6.43950500	2.57826400	1.12504300
C	-5.88808200	1.19851900	0.78107100
H	-3.23001900	2.58502800	2.03866900
H	-3.74223100	3.42926400	0.56205700
H	-5.41019100	4.25792800	2.12499900

H	-5.41771800	2.72520200	3.02838800
P	2.69059400	-0.40706100	1.33556100
O	1.30572400	-0.12784200	0.73592700
H	-6.53674500	3.17999300	0.20678200
H	-7.42695500	2.52829600	1.60458700
Sm	0.10170900	0.65829600	-1.12282500
N	2.57651200	-1.85853500	2.19027400
C	1.74942300	-2.92664100	1.61461300
C	2.72517900	-3.88412400	0.90878400
C	4.11328700	-3.54119700	1.48857700
C	3.82680000	-2.50978200	2.58776400
I	1.92329500	3.14872500	-1.67808500
I	-0.32209200	-2.30003200	-2.12938700
N	3.83649900	-0.36956200	0.13110900
C	3.47745700	-0.60400000	-1.27757200
C	4.82902900	-0.79290700	-1.95509400
C	5.74648600	0.10102700	-1.12727500
C	5.27739100	-0.16982500	0.29715000
H	-6.27165800	0.81158500	-0.17401900
H	-6.15444400	0.46636600	1.56589400
N	-2.87609300	-0.53097200	1.75949900
C	-1.57845100	-1.20764300	1.91236000
C	-1.84414800	-2.22665300	3.01493400
N	3.28619300	0.65995000	2.48279200
C	3.45905700	2.05460200	2.02630300
C	2.29814700	2.86005800	2.64544000
C	1.56782400	1.87036100	3.56775000
C	2.57925900	0.74324300	3.76651300
H	2.81150900	-1.47180300	-1.39956400
H	2.97921800	0.29381100	-1.68924300
H	4.79502300	-0.52329600	-3.01916700
H	5.15039100	-1.84504600	-1.87880900
H	5.56517900	1.15762300	-1.38130200
H	6.81578500	-0.11253900	-1.26414100
H	5.48965700	0.66049800	0.98422200
H	5.76857900	-1.07525300	0.70121300
C	-2.92779300	-1.55660200	3.85516800
C	-3.82548900	-0.92026200	2.79889400
H	-1.25020900	-1.68194900	0.97351500
H	-0.79584100	-0.48838500	2.20205200
H	-0.93565700	-2.46929800	3.58322600
H	-2.23149200	-3.16187500	2.57857400
H	-2.48362300	-0.76969200	4.48633600
H	-3.47435300	-2.25066300	4.50874100
H	4.43376400	2.41946400	2.38900000
H	3.45876200	2.10957100	0.92801100
H	1.63965800	3.25291100	1.85805300
H	2.68719500	3.72118900	3.20807700
H	0.67570100	1.46627600	3.06435000
H	1.24542700	2.32203300	4.51728300
H	2.13579800	-0.22758000	4.02129100
H	3.31578600	1.00261100	4.54722600
H	1.23304500	-3.43696700	2.44545200
H	0.98975200	-2.51134200	0.94094700
H	2.69514700	-3.72407600	-0.17880500
H	2.44620200	-4.93279600	1.08439300
H	4.75286700	-3.09434700	0.71264900
H	4.64348900	-4.42003500	1.88289200
H	4.62449900	-1.76815400	2.73446300
H	3.66012600	-3.00901400	3.55814500
H	-4.37559200	-0.04609600	3.18031000
H	-4.56953800	-1.65196000	2.42890100
O	-2.04131100	0.98833800	-0.18862500
P	-3.28872000	0.29154500	0.37269200
N	-4.06669800	-0.76844700	-0.64706500
C	-4.36786900	-0.37604300	-2.02717200
C	-5.10931600	-1.59228300	-2.55888400
C	-4.33532700	-2.74222900	-1.92051000
C	-3.99782300	-2.23510700	-0.51725300
H	-4.96394200	0.54971700	-2.04656200
H	-3.44143200	-0.20342100	-2.60416700
H	-5.11744700	-1.63208800	-3.65692400
H	-6.15397400	-1.57749600	-2.20561100
H	-3.39726600	-2.91018900	-2.47186600
H	-4.89574300	-3.68705800	-1.89567700
H	-2.99827900	-2.57939900	-0.21328800
H	-4.73412700	-2.57823800	0.22998500
N	-4.44510400	1.43392600	0.71828500

$SmI_2(TPPA)_3$

H_{corr} = 1.1686

$-TS$ = -0.1571

$E_{sol(THF)}$ = -27795.0739

$G_{sol(THF)}$ = -27794.0624

H	-0.22085500	3.86176900	-1.33544100
H	0.46233900	5.34629900	-0.59354000
C	2.57628800	3.10610200	0.02268300
C	3.06648900	4.07599200	-1.04210300
H	1.68188700	3.37793200	-2.56030200
H	3.93341100	3.68599500	-1.59324200
C	1.82863600	4.25684300	-1.91155700
C	0.68538200	4.31007500	-0.90344100
P	-3.88071600	-1.20447500	-0.42478100
O	-2.41808300	-1.32773800	-0.83880000
C	2.15211600	3.25896900	4.55328000
H	3.16273200	3.15646500	0.95347500
Sm	-0.09917900	-0.67558300	-0.74431700
N	-4.53043100	-2.75517200	-0.34522800
C	-3.67433400	-3.86902400	0.07541400
C	-4.09131400	-4.18883400	1.52269600
C	-5.45004800	-3.48248800	1.71613200
C	-5.81339300	-2.97051300	0.31722500
I	-0.68280800	1.39655700	-3.17555200
I	0.14348900	-3.19120700	1.34122500
N	-3.95494700	-0.29024700	0.98030200
C	-2.87875900	-0.41710600	1.98384600
C	-3.56258900	-0.04101900	3.29335000
C	-4.64999800	0.93153900	2.84694700
C	-5.16569800	0.28337600	1.56669900
N	0.87796200	2.68795100	2.70370200
H	3.35979100	5.03121100	-0.57534800
C	0.49408400	1.70757800	3.72912500
H	2.61539800	2.06549600	-0.34369200
H	2.40740200	4.02360600	5.30039400
N	-4.94293500	-0.39466600	-1.43706000
C	-4.55117400	1.00293900	-1.75152800
C	-4.85190000	1.18234100	-3.23860500
C	-4.72366400	-0.23301000	-3.78789500
C	-5.36540200	-1.06079600	-2.68540800
H	-2.44333200	-1.42789300	2.00412100
H	-2.05837000	0.28091700	1.74952400
H	-2.85957300	0.38765800	4.02167800
H	-4.02082400	-0.93099900	3.75575300
H	-4.20381500	1.91357000	2.61777600

H	-5.44564100	1.08256800	3.59037600
H	-5.63816300	1.00035600	0.87930100
H	-5.92055800	-0.48605100	1.81462900
H	2.27583800	4.27103700	2.59701600
H	0.82376900	4.65348300	3.55120800
H	0.29066800	2.84155700	5.57163700
H	1.45039100	1.50573300	5.69776600
H	3.07312800	2.71352100	4.29157000
C	1.53737200	3.84754400	3.29142600
H	-0.60080000	1.58772900	3.78808800
H	0.91237000	0.72011500	3.47956300
H	-5.10721700	1.71501500	-1.12065100
H	-3.47358500	1.14556800	-1.57897400
H	-4.14881500	1.88862000	-3.70122800
H	-5.87820900	1.55806100	-3.38523800
H	-3.65724700	-0.49510500	-3.88053400
H	-5.20745200	-0.37049500	-4.76563600
H	-5.06212100	-2.11524500	-2.67849300
H	-6.46751900	-1.02698700	-2.75574000
H	-3.88115100	-4.72471600	-0.58962200
H	-2.61306900	-3.60700300	-0.02204200
H	-3.33630000	-3.81183500	2.22814900
H	-4.16264700	-5.27432800	1.68168600
H	-5.34591900	-2.63164400	2.40759500
H	-6.22658900	-4.14365000	2.12786000
H	-6.42249500	-2.05583400	0.30710000
H	-6.36984000	-3.74157300	-0.24516500
C	1.07081100	2.28784700	5.02529100
H	1.86317200	5.15105800	-2.54914700
O	2.28508500	-0.82351100	-1.03420200
P	3.70862500	-1.05839100	-0.53634300
N	4.01767900	-1.01115400	1.10768100
C	3.66574800	0.19588400	1.87107300
C	3.82545300	-0.25382900	3.31562700
C	3.30816600	-1.68589900	3.26625500
C	3.85837300	-2.21756000	1.94725700
H	4.32888700	1.03370600	1.60543800
H	2.62618400	0.51117100	1.67298500
H	3.27322500	0.38821600	4.01617400
H	4.89055200	-0.23310800	3.60116000
H	2.20710900	-1.69705000	3.21648700
H	3.61743900	-2.29876300	4.12427200
H	3.15875300	-2.94168000	1.50512400
H	4.84025100	-2.70396200	2.08515200
N	4.66952000	0.18265800	-1.10902800
C	4.41913700	0.70180300	-2.46379000
C	5.71567200	1.43359700	-2.82240500
C	6.36103900	1.73135500	-1.47025500
C	6.02725400	0.48264700	-0.66677800
H	4.19228100	-0.10757900	-3.17582500
H	3.54435500	1.37102600	-2.45340600
H	5.53251800	2.33269500	-3.42757900
H	6.37443900	0.77147500	-3.40684500
H	5.88740800	2.60853600	-1.00027800
H	7.44202500	1.92030500	-1.53350300
H	6.06020700	0.63870500	0.42068000
H	6.74143500	-0.32791000	-0.90740300
N	4.22211900	-2.54479800	-1.08905600
C	3.28100900	-3.64619800	-1.35124000
C	4.06681300	-4.87733000	-0.92339300
C	5.49991200	-4.51126100	-1.29759300
C	5.59337800	-3.03045500	-0.92661900
H	2.34502100	-3.52148500	-0.78699200
H	3.03203600	-3.68639500	-2.42663800
H	3.70935800	-5.79575100	-1.40939800
H	3.97684500	-5.01347300	0.16667100
H	5.64622500	-4.63318600	-2.38319100
H	6.26183500	-5.11650900	-0.78649500
H	6.29196600	-2.48744100	-1.58356700
H	5.94603000	-2.90184100	0.11308500
O	0.02378900	1.12547400	0.82299100
P	0.18174100	2.60069700	1.19200200
N	-1.32675300	3.31194100	1.20635200
C	-2.35322900	2.95003000	0.21119400
C	-3.10766700	4.25462900	-0.00741900
C	-3.00240100	4.94470800	1.34942900
C	-1.56981400	4.63744400	1.77707600
H	-1.90583200	2.55824600	-0.71603900
H	-3.01188400	2.16399300	0.61403700
H	-4.14326200	4.09029500	-0.33650600
H	-2.60101900	4.85710200	-0.77899700
H	-3.70919200	4.48709200	2.06144100
H	-3.20692900	6.02407300	1.31767100
H	-1.45375800	4.62485800	2.87234100
H	-0.87000400	5.39385700	1.37417100
N	1.19161200	3.54528100	0.25597300

$SmI_2(TPPA)_4$

H_{corr} = 1.5553

$-TS$ = -0.1967

$E_{sol(THF)}$ = -28848.0967

$G_{sol(THF)}$ = -28846.7381

H	1.58456000	-2.59519600	3.15277600
H	3.01391500	-3.56487500	3.63545700
C	4.12816600	-1.41910700	1.62393200
C	4.55033000	-1.13800300	3.05753100
H	2.55514300	-0.56281500	3.69415200
H	4.94012100	-0.11867000	3.18570100
C	3.26072700	-1.39964000	3.82516400
C	2.68407500	-2.63465300	3.14008600
P	-3.35594700	-2.05495100	-0.66958500
O	-2.25317400	-1.11660300	-0.20771400
C	5.35973800	-4.06193500	-1.80605700
H	4.97586700	-1.65699300	0.96286700
Sm	-0.08199300	-0.02716300	-0.00483600
N	-4.51145400	-1.15940000	-1.51189400
C	-4.09418000	0.06977800	-2.20152500
C	-4.19119400	-0.23062600	-3.71224100
C	-4.76507600	-1.65449300	-3.79880300
C	-5.40460500	-1.86487600	-2.42769900
I	-0.65979300	-0.52145900	3.23705800
I	-0.15433000	0.46523000	-3.31002200
N	-2.66625100	-3.33400500	-1.49493600
C	-1.34522400	-3.20107000	-2.13964400
C	-1.20103700	-4.50507800	-2.92362200
C	-2.09932900	-5.48484800	-2.17277900
C	-3.29363800	-4.61030000	-1.81461100
N	3.43022900	-3.45215400	-0.68253700
H	5.33783300	-1.84631600	3.36543800
C	3.00696100	-3.57092700	-2.08537200
H	3.59344400	-0.55233900	1.19873400
H	6.16525400	-4.79831800	-1.93750000
N	-4.26374200	-2.84732500	0.50367200
C	-3.43673600	-3.61019700	1.47089100

C	-4.09847500	-3.38763500	2.82926200
C	-4.75501000	-2.02303300	2.66455700
C	-5.29576500	-2.10179000	1.24457600
H	-1.28169600	-2.31383900	-2.78841100
H	-0.55766200	-3.09754000	-1.37676500
H	-0.15375600	-4.83234500	-2.99035300
H	-1.57363600	-4.37439600	-3.95286200
H	-1.60299100	-5.83024500	-1.25033800
H	-2.38134400	-6.36853700	-2.76325300
H	-3.88119300	-4.98322800	-0.96371700
H	-3.97513700	-4.53418100	-2.68505000
H	5.24817600	-3.69407900	0.36772600
H	4.52246600	-5.22462600	-0.17734600
H	3.98802600	-5.33955500	-2.88407100
H	4.38328100	-3.85975800	-3.77606300
H	5.79094000	-3.05486300	-1.92504400
C	4.68036000	-4.16211300	-0.44805500
H	2.08093500	-4.16250400	-2.18313500
H	2.79365200	-2.57298800	-2.49813400
H	-3.38699800	-4.67558700	1.19306200
H	-2.41446300	-3.20476600	1.48792700
H	-3.36247600	-3.41581700	3.64458600
H	-4.86169000	-4.16008000	3.02088500
H	-3.98047000	-1.24144200	2.72765400
H	-5.53240100	-1.81550900	3.41436800
H	-5.47516900	-1.12738200	0.77239300
H	-6.24486200	-2.66727600	1.21516300
H	-4.78857800	0.87859300	-1.91596200
H	-3.08529700	0.36356500	-1.88449100
H	-3.20138300	-0.15513300	-4.18472200
H	-4.85478800	0.49411800	-4.20821100
H	-3.95059100	-2.38534500	-3.92912800
H	-5.47389600	-1.78905500	-4.62912500
H	-5.52334900	-2.91705700	-2.13244500
H	-6.40493100	-1.39647600	-2.38376500
C	4.19194800	-4.26285000	-2.77150000
H	3.40750000	-1.55246000	4.90332600
O	2.00804900	1.27638000	0.18971000
P	3.13465100	2.07963000	-0.44600800
N	3.91620600	1.42075700	-1.77179500
C	4.39527700	0.03381900	-1.68771900
C	4.82544400	-0.26455000	-3.11596200
C	3.76221600	0.46053700	-3.93179400
C	3.52930000	1.75465000	-3.15720500
H	5.22099300	-0.04952300	-0.96314400
H	3.59212500	-0.65051700	-1.36345200
H	4.87232800	-1.34324400	-3.32171600
H	5.82450400	0.16285000	-3.30541300
H	2.82303500	-0.11607200	-3.93677400
H	4.05544800	0.64626900	-4.97446600
H	2.47472200	2.05661600	-3.23566500
H	4.16681700	2.57505500	-3.53249500
N	4.37615200	2.23462500	0.66357400
C	4.04976100	2.51040700	2.06691100
C	5.40750500	2.82617100	2.70857200
C	6.43128100	2.25683600	1.72657600
C	5.77477300	2.52038400	0.37871100
H	3.35089000	3.35882600	2.16341900
H	3.55470200	1.63529500	2.51524900
H	5.49702300	2.40825200	3.72138500
H	5.54170900	3.91643200	2.79122700
H	6.54217600	1.17005300	1.87201500

H	7.42475200	2.71859100	1.81751200
H	6.14773900	1.87656700	-0.42920000
H	5.94044400	3.57173900	0.06999900
N	2.58962800	3.60124600	-0.86475800
C	1.24355300	3.83594000	-1.42098000
C	1.48191100	4.93067800	-2.45092400
C	2.60240200	5.74954400	-1.81744800
C	3.51416000	4.67946200	-1.22046600
H	0.81009100	2.92095200	-1.85199000
H	0.56176400	4.17968200	-0.62575700
H	0.57681800	5.51826200	-2.65957900
H	1.82255900	4.48734000	-3.40086100
H	2.19381900	6.38229500	-1.01186300
H	3.13329900	6.40386900	-2.52328400
H	4.06137600	5.04384100	-0.33587200
H	4.26439100	4.34626700	-1.96189600
O	1.31929300	-2.04283100	-0.14043400
P	2.28964800	-3.04646000	0.46832600
N	1.41974800	-4.38862100	0.95274500
C	0.02315500	-4.26576100	1.39866400
C	-0.08613800	-5.30633700	2.50412100
C	0.86921300	-6.39969800	2.03514800
C	2.04476100	-5.60897200	1.46242200
H	-0.20400900	-3.24759300	1.75009800
H	-0.66466400	-4.49289200	0.56584800
H	-1.11695000	-5.65859400	2.65153800
H	0.26309800	-4.87501500	3.45630200
H	0.39579500	-6.99358400	1.23581400
H	1.17941300	-7.09219600	2.83032000
H	2.56005900	-6.15992900	0.65932200
H	2.79244700	-5.38632400	2.24621000
N	3.23713500	-2.58314700	1.76804700
P	-2.07536500	3.20821900	0.68435100
N	-3.31939200	2.81027300	1.73164800
C	-4.26761500	1.76119600	1.33161800
C	-5.12764400	1.60800900	2.57567700
C	-4.09582300	1.72871000	3.69227000
C	-3.14018100	2.81420500	3.19620200
H	-4.84186600	2.07367300	0.44590600
H	-5.68047800	0.65897200	2.59317800
H	-5.85937300	2.43167200	2.62855700
H	-3.53661500	0.78483300	3.79034100
H	-4.53257500	1.97349300	4.67077300
H	-2.10737600	2.57993100	3.49230700
H	-3.40299900	3.80812600	3.59854200
N	-2.80312500	4.10037900	-0.52683400
C	-2.38055400	3.93190000	-1.92492300
C	-3.65260200	4.20007100	-2.75089800
C	-4.79381700	4.20297600	-1.73180500
C	-4.11600300	4.72260400	-0.46880800
H	-3.78746100	3.44730600	-3.54034100
H	-3.58787000	5.18314200	-3.24320000
H	-5.15140900	3.17567800	-1.55483100
H	-5.65336800	4.81362000	-2.04495400
H	-4.62477700	4.45360700	0.46636600
H	-4.03147500	5.82683500	-0.50300400
H	-1.98968200	5.73470600	2.64702700
O	-1.29829300	2.05256000	0.07642700
H	-3.74281100	0.82084100	1.08429300
H	-1.58644200	4.65074500	-2.18780600
H	-1.97141900	2.92219200	-2.07583600
C	-1.24866900	5.59129100	1.83529500

H	-1.61744700	6.12550500	0.94607200
N	-1.02707500	4.18556600	1.53540100
C	0.18512100	3.65652500	2.18168500
C	0.65409100	4.82983300	3.03921600
C	0.13195200	6.05292300	2.28849700
H	-0.01984800	2.75197400	2.77664300
H	0.93181000	3.37697100	1.42206400
H	1.74461500	4.83467800	3.17991200
H	0.18895900	4.77986900	4.03740300
H	0.76253300	6.25188600	1.40560300
H	0.09693800	6.96599200	2.89976900

$SmI_2(TPPA)_5$
H_{corr} = 1.9432
$-TS$ = -0.2273
$E_{sol(THF)}$ = -29901.1036
$G_{sol(THF)}$ = -29899.3877

H	1.88660600	2.90066200	2.86875100
H	3.55919200	3.49682900	3.00996700
C	1.49074300	4.61345900	0.65734300
C	1.01420600	5.39946700	1.89225500
H	1.59221000	5.06518200	3.99833900
H	0.01415000	5.05265500	2.18496400
C	2.03228900	5.07710200	2.99078000
C	2.57370800	3.71820200	2.57653900
P	2.90711000	-2.93413400	0.23419100
O	1.77827400	-1.91513000	0.20431700
C	4.35103800	4.84266200	-2.81248000
H	1.76931400	5.27682900	-0.17652500
Sm	0.17221300	-0.02528400	-0.04488800
N	2.39343300	-4.30476400	-0.61690900
C	1.48934100	-4.11320200	-1.75652100
C	2.33929100	-4.34372600	-3.02386400
C	3.68623800	-4.88518500	-2.51133100
C	3.39574400	-5.26960100	-1.05940700
I	1.07596800	-0.11138700	3.50894300
I	1.15301200	-0.20286200	-3.44985700
N	4.31569800	-2.21572600	-0.31297600
C	4.25434600	-1.17131000	-1.35349100
C	5.67038500	-1.14940700	-1.92088600
C	6.52836900	-1.63547500	-0.75681200
C	5.66868700	-2.74373900	-0.16215900
N	3.66636800	3.31916800	-1.21813500
H	0.93931000	6.47830200	1.69474200
C	4.07608500	2.47072500	-2.34719000
H	0.70707900	3.92771100	0.29076300
H	5.07223200	5.61582100	-3.11465200
N	3.40809500	-3.60853200	1.68309800
C	3.88046100	-2.69905200	2.74784600
C	3.36383000	-3.32603200	4.03795400
C	3.25692100	-4.80534800	3.69091600
C	2.71808000	-4.76492200	2.26864500
H	3.49511200	-1.38513000	-2.11990200
H	3.97067000	-0.21019500	-0.89997200
H	5.95543300	-0.15519200	-2.29255700
H	5.75446100	-1.85303300	-2.76598000
H	6.65740100	-0.82730200	-0.01797200
H	7.52532800	-1.99026000	-1.05570200
H	5.89908500	-2.95499800	0.89159600
H	5.82354500	-3.68071800	-0.73162900
H	3.76174400	5.38048200	-0.75419500
H	5.35489800	4.60410400	-0.90390100
H	5.78282400	3.32394600	-3.39899000
H	4.30466600	3.29291400	-4.37433100
H	3.35049100	5.15449200	-3.14983700
C	4.32301000	4.61669000	-1.30814300
H	4.82251600	1.72229200	-2.02801200
H	3.21005800	1.91421300	-2.73733100
H	4.98437800	-2.64426800	2.73608600
H	3.47123300	-1.68701600	2.62139900
H	2.37005600	-2.90444200	4.25972700
H	4.01767800	-3.11618600	4.89597700
H	2.60101300	-5.37277000	4.36706500
H	4.25228900	-5.28060600	3.70263800
H	1.62521600	-4.62169000	2.28128400
H	2.93145400	-5.68122300	1.69980700
H	0.67495200	-4.85196700	-1.68406900
H	1.03552000	-3.11558700	-1.71649800
H	2.46105300	-3.40479100	-3.58358200
H	1.84854800	-5.06399700	-3.69569500
H	4.44979900	-4.09218500	-2.53214600
H	4.06962900	-5.72911500	-3.10365000
H	4.27581300	-5.25658800	-0.40066500
H	2.95737800	-6.28345500	-1.00715400
C	4.69010800	3.45398800	-3.35826100
H	2.85019000	5.81459300	2.98351600
O	-0.96867500	2.21011100	-0.68042400
P	-1.79477300	2.97769300	-1.70765100
N	-0.80144000	3.60093000	-2.89217300
C	0.66421100	3.53200000	-2.82960400
C	1.11211400	3.57945200	-4.29808500
C	-0.14810700	3.26804300	-5.10129000
C	-1.24885500	3.88240500	-4.24879700
H	1.07705800	4.38129300	-2.26035300
H	0.97805800	2.60539300	-2.33121300
H	1.90557900	2.84511200	-4.49132700
H	1.48994500	4.58263400	-4.55525900
H	-0.28606300	2.17596400	-5.14544300
H	-0.12224200	3.66725600	-6.12587400
H	-2.23466600	3.43783500	-4.43737700
H	-1.33247400	4.97348600	-4.42450300
N	-2.66278900	4.31104500	-1.14890000
C	-3.65227800	4.15057800	-0.07475400
C	-3.86099400	5.57087700	0.43527800
C	-2.46957500	6.17598400	0.29975800
C	-1.98595600	5.61401800	-1.03316800
H	-4.58541800	3.71520000	-0.46223900
H	-3.28024200	3.48627100	0.72428000
H	-4.25933500	5.59560000	1.45973000
H	-4.57161800	6.10559200	-0.21631500
H	-1.82393600	5.81181100	1.11486600
H	-2.45875800	7.27497200	0.32447200
H	-0.89222900	5.51262800	-1.07368400
H	-2.28458600	6.26933800	-1.87067300
N	-3.01673900	2.02958000	-2.34818900
C	-2.64471700	0.70320500	-2.87176300
C	-3.50878700	0.55896400	-4.11435000
C	-4.78577600	1.28959400	-3.71287000
C	-4.27133300	2.50308600	-2.93757300
H	-1.56686700	0.64122600	-3.09288200
H	-2.85703900	-0.08006500	-2.12716400
H	-3.67388400	-0.49070100	-4.39575700
H	-3.02920300	1.06108600	-4.97077600
H	-5.38453400	0.64737600	-3.04454300

H	-5.42480000	1.57840500	-4.55961000
H	-4.98927400	2.82011300	-2.16467300
H	-4.11765200	3.36808900	-3.60540100
O	2.17497100	1.46150800	-0.06989000
P	3.18798300	2.56125400	0.19555700
N	4.53043300	1.91231000	0.94785400
C	4.44680500	0.72567100	1.81387400
C	5.46528900	1.01524000	2.90546200
C	6.54251100	1.80097200	2.16276500
C	5.73841200	2.69235300	1.21549800
H	3.42853500	0.57750400	2.20165900
H	4.71896700	-0.17842800	1.24535200
H	5.84292000	0.09903900	3.38142300
H	5.00740900	1.63583100	3.69346800
H	7.17155100	1.11017700	1.57704300
H	7.20647800	2.38224500	2.81855300
H	6.29234400	2.90990400	0.28776100
H	5.49596400	3.66257900	1.68929800
N	2.67706400	3.87466900	1.12162200
P	-2.99725500	0.42673000	2.35174100
N	-3.22345200	-0.30378300	3.85033900
C	-3.37132700	-1.76633500	3.88374900
C	-2.23038000	-2.23249500	4.77482200
C	-2.17549900	-1.12983000	5.82445800
C	-2.41218500	0.13824400	5.00581000
H	-4.35177900	-2.03828200	4.31887200
H	-1.29061000	-2.22655700	4.19992300
H	-2.39284800	-3.23619700	5.19401600
H	-1.21169400	-1.08767600	6.35011600
H	-2.97538000	-1.26918300	6.57074900
H	-1.44223200	0.54111900	4.67517300
H	-2.94898400	0.90791000	5.58441100
N	-4.49639700	0.15991500	1.64244700
C	-4.67225100	0.58093000	0.24871100
C	-6.07774700	1.16674500	0.22204300
C	-6.81098900	0.32076700	1.25944700
C	-5.77119800	0.14944100	2.36497400
H	-6.53732800	1.13326000	-0.77691500
H	-6.05382900	2.21999200	0.54777600
H	-7.06847000	-0.66047000	0.82895500
H	-7.74070100	0.77719000	1.62736700
H	-5.90618400	-0.79110400	2.92086000
H	-5.84000300	0.97278500	3.09596100
H	-4.17594000	2.20319500	4.28188200
O	-1.90239000	-0.06825800	1.42611700
H	-3.32812800	-2.18649400	2.86936600
H	-3.89122500	1.28736600	-0.06178200
H	-4.59053100	-0.28790900	-0.42599100
C	-3.78631400	2.81698500	3.45094300
H	-4.65054000	3.12597300	2.83749500
N	-2.80976200	2.06654500	2.65669200
C	-1.48200100	2.69596900	2.71763800
C	-1.55626100	3.54098400	3.98052400
C	-3.00324300	4.02476100	3.97523200
H	-0.68537100	1.93804800	2.75306500
H	-1.31918800	3.31199000	1.81566200
H	-0.82817500	4.36385300	3.99239600
H	-1.35849800	2.90876700	4.85995100
H	-3.10804400	4.87242800	3.27871800
H	-3.36584300	4.35771700	4.95817100
H	-3.48334100	-5.18768200	-2.48958700
C	-2.54582700	-5.06783700	-3.05265600
H	-1.95351200	-5.98709200	-2.89567300
C	-2.21198300	-3.40900900	-4.81537200
C	-2.75905700	-4.81951900	-4.56494100
H	-1.78547700	-3.28436400	-5.82065100
H	-3.00866100	-2.65808000	-4.69150500
H	-2.19170200	-5.56000300	-5.14903500
H	-3.81510500	-4.92247400	-4.85530100
H	-0.43515600	-4.51632400	3.22656000
C	-1.18107800	-3.23415200	-3.70916800
H	-0.91910700	-2.19541300	-3.47037400
H	-0.24178400	-3.75141600	-3.97521600
N	-1.82118800	-3.89530600	-2.57601700
P	-2.07990600	-3.12563100	-1.11456200
N	-3.75834700	-3.23876900	-0.94670700
C	-4.41327000	-3.17229800	0.35754800
C	-5.86957300	-3.42213600	-0.00491100
C	-6.03247800	-2.64027500	-1.30917200
C	-4.65600600	-2.72879300	-1.98796000
H	-4.00001400	-3.93605400	1.03204200
H	-6.56674900	-3.10077500	0.78279000
H	-6.02607300	-4.49843400	-0.18085100
H	-6.27650000	-1.58967800	-1.09100200
H	-6.83584900	-3.03347600	-1.94771600
H	-4.33650200	-1.74345100	-2.36766100
H	-4.66857900	-3.41949900	-2.84664400
N	-1.49295100	-4.04881000	0.13877600
C	-1.13975300	-4.59064800	2.38626700
C	-0.95138400	-5.84215500	1.53349400
C	-1.68861700	-5.48781000	0.24538800
H	-1.33240000	-6.76198600	1.99923400
H	0.11820400	-5.98335200	1.31112000
H	-2.76285700	-5.75305800	0.31456800
H	-1.27493100	-6.01554600	-0.62870900
H	-4.29159900	-2.18456300	0.83970500
C	-0.95582800	-3.46370100	1.37800700
H	-2.16005800	-4.57096600	2.80504400
O	-1.45801000	-1.74031300	-1.09388600
H	0.10577600	-3.20199700	1.25143900
H	-1.48069600	-2.53707800	1.65153600

Basicity of LB and Reducing Power of SmI_2LB_n

$THF\text{-}H^+$

H_{corr} = 0.1361

$-TS$ = -0.0338

$E_{sol(THF)}$ = -232.7766

$G_{sol(THF)}$ = -232.6743

H	1.58810700	-0.39283100	-1.17115600
H	1.97018400	-0.76766300	0.55442000
H	-1.46120900	-0.47217400	1.21549900
H	-2.00094100	-0.81378600	-0.48308400
H	0.74224500	1.17596300	1.34516500
H	1.28797000	1.83743400	-0.21419500
H	-1.36786800	1.77811900	0.22962100
H	-0.79953200	1.13840400	-1.33105600
O	0.01095700	-1.20247400	-0.10184500
C	1.24363800	-0.31893700	-0.13317100
C	0.70165700	1.03586600	0.25459600
C	-0.74575000	1.00464900	-0.24032900
C	-1.22262900	-0.37097900	0.14799500
H	0.09189100	-1.96726700	0.49499900

MeTHF-H+

H_{corr} = 0.1651

$–TS$ = -0.0371

$E_{sol(MeTHF)}$ = -272.0795

$G_{sol(MeTHF)}$ = -271.9515

H	-1.59422200	-0.94485100	1.31290400
H	-2.08799600	-1.42322900	-0.35428100
C	-1.46208900	-0.74916400	0.24239200
C	0.79996100	0.12888400	-0.40044600
C	-0.09980200	1.21211500	0.14446900
C	-1.51625600	0.71422800	-0.13288300
H	0.80434200	0.12274500	-1.50126000
C	2.16619700	-0.03282200	0.18961500
H	0.11287400	2.16756900	-0.35403100
O	-0.02904000	-1.12266900	-0.02265600
H	0.07781500	1.34376100	1.22345700
H	-1.77332300	0.83471000	-1.19586400
H	-2.27449100	1.24169800	0.46078600
H	2.12130900	-0.11603600	1.28417900
H	2.75082700	0.86440300	-0.06569700
H	2.69856200	-0.89965800	-0.22556500
H	0.06854700	-1.84920300	-0.66226500

THP-H+

H_{corr} = 0.1665

$–TS$ = -0.0365

$E_{sol(THP)}$ = -272.0639

$G_{sol(THP)}$ = -271.9339

H	-0.55481400	-1.00776300	1.47859300
H	-1.42298000	-1.96806900	0.25113100
H	0.67282500	1.01214200	1.50931100
H	1.56240900	1.88081100	0.26534000
H	-2.24079000	0.51333200	0.67266700
O	0.45172200	-1.28353900	-0.27252400
C	1.49566700	-0.19611500	-0.08082300
C	0.83855900	1.06593900	0.42211200
C	-1.49917400	0.21938400	-0.08737300
C	-0.85391500	-1.04680600	0.42491800
C	-0.46408700	1.33755700	-0.32491000
H	-2.07116000	0.01398300	-1.00473300
H	-0.24867900	1.42701100	-1.40269100
H	-0.87661800	2.30800100	-0.02020500
H	1.93869100	-0.08468300	-1.07914300
H	2.21968600	-0.65025400	0.60580700
H	0.30536100	-1.45595000	-1.21943000

HMPA-H+

H_{corr} = 0.2841

$–TS$ = -0.0581

$E_{sol(THF)}$ = -821.2348

$G_{sol(THF)}$ = -821.0089

H	-1.85202800	1.47025200	1.10152500
C	-1.13622600	2.21158900	0.72808700
H	1.24643100	-2.11350300	-1.49410500
H	2.89884600	-1.87552500	-0.86819000
H	-0.28670500	-1.54882900	1.87362700
H	-1.93284300	-0.94503600	2.21642900
H	1.95004100	1.85789100	-0.67007900
H	-2.99704700	-1.80018600	-0.64322200
P	-0.01076200	-0.01589500	-0.34413400
O	-0.18080000	-0.15391800	-1.95336900
H	-1.70174600	-2.51261100	1.38525500
H	1.70651900	-2.79967600	0.09460000
H	-2.53022700	-0.29074800	-1.46220500
N	-1.32888300	-0.83589900	0.18306100
C	-2.62607900	-0.77623900	-0.48495500
H	1.73664200	0.54062600	1.73938800
H	2.15204000	-1.18024300	1.98819100
C	-1.31143400	-1.48845300	1.48571600
N	0.04135400	1.54807500	0.17965100
C	1.10613300	2.43871700	-0.27304500
N	1.43041200	-0.69653300	0.05966400
C	1.83737900	-1.94315800	-0.58636600
H	0.74271300	3.12888200	-1.05310300
H	-1.63363100	2.84365000	-0.02580800
C	2.17179500	-0.34720500	1.26641300
H	3.22118300	-0.13684200	1.00879900
H	-0.82730600	2.84744200	1.57115100
H	1.47532900	3.03701500	0.57381300
H	-3.35948600	-0.22584100	0.12677500
H	0.34951100	0.49205100	-2.44562300

TPPA-H+

H_{corr} = 0.3978

$–TS$ = -0.0641

$E_{sol(THF)}$ = -1053.4133

$G_{sol(THF)}$ = -1053.0795

H	-2.43115200	-0.96317400	1.36004400
H	-3.37099800	-1.85693200	-0.66711800
H	-3.42901900	-3.30711900	0.36081600
H	-2.09798000	-3.51669100	-1.98361400
H	-1.25339100	-4.07791000	-0.52244000
H	-0.93882400	-1.39184100	-1.95280800
H	0.33895100	-2.48590200	-1.36262800
H	2.07322000	1.66926500	-1.23026300
P	0.07676500	-0.03887300	0.51823600
O	0.04401200	-0.05556700	2.14206200
H	2.33843000	1.72504400	0.52832700
H	4.57325500	1.21712500	-0.20366500
H	0.01533900	1.64638200	-1.94403800
N	-0.70521100	-1.43860500	0.17718800
C	-1.91601100	-1.83717500	0.93611500
C	-2.75493100	-2.57315700	-0.09977600
C	-1.70433400	-3.19891100	-1.00932300
C	-0.66413000	-2.09466300	-1.14887600
H	-1.65380800	1.06051400	-1.70562200
H	-1.62266700	-2.49300500	1.77021100
N	-0.56848800	1.41993100	0.09606900
C	-1.28457800	2.32709700	1.02271600
C	-2.17721800	3.14793100	0.09668000
C	-1.40690900	3.16455800	-1.21924100
C	-0.87889500	1.74101900	-1.31149700
H	3.98868200	0.27657500	-1.59246700
H	3.81653000	-0.55478600	1.36235900
H	4.57423000	-1.47692900	0.04149400
H	1.98497500	-2.02109700	0.69511400
H	2.42271300	-1.87254000	-1.02815300
N	1.54893500	-0.12868400	-0.19763000
C	2.39760600	1.07852800	-0.36249600
C	3.80228800	0.50795900	-0.53202100
C	3.76286600	-0.77840600	0.28456000
C	2.39349900	-1.33992300	-0.06367100
H	-1.85982100	1.77263700	1.77762600
H	-0.55801700	2.97356000	1.54511200

H	-2.37748700	4.14872200	0.50081700
H	-3.14460000	2.63899900	-0.03849500
H	-0.56545000	3.87343800	-1.16525100
H	-2.02551500	3.43522300	-2.08473100
H	0.35665800	0.77043600	2.54093200

TMTHF-H$^+$
H_{corr} = 0.2517
$-TS$ = -0.0455
$E_{sol(TMTHF)}$ = -389.9779
$G_{sol(TMTHF)}$ = -389.7717

H	2.84913900	-1.41549900	0.30565800
O	-0.00439400	-0.00702800	-0.89375900
C	1.28952400	0.00031200	-0.02401500
C	0.69470000	0.18447100	1.36670300
C	-0.69482800	-0.43801200	1.30934700
C	-1.28820400	-0.01481300	-0.02818700
C	2.13615500	1.14784300	-0.51623100
C	1.90880400	-1.35553700	-0.26145400
C	-2.20439400	-1.02641600	-0.66792500
H	2.14059200	-1.50532200	-1.32465600
C	-1.83515000	1.39539500	-0.04368000
H	-3.09141000	-1.14695500	-0.02836600
H	-2.54735500	-0.69830800	-1.65928500
H	-2.77979600	1.41101100	0.51887700
H	-2.07011500	1.72566300	-1.06799400
H	2.47503000	0.99577000	-1.55186300
H	3.03519400	1.20818300	0.11484200
H	-0.64014500	-1.53490400	1.36392800
H	-1.33704100	-0.09886100	2.13314200
H	1.25447100	-2.17019200	0.07710100
H	1.33242900	-0.29832600	2.11931600
H	0.64001600	1.25372200	1.61844700
H	-1.71304600	-2.00407800	-0.76033000
H	-1.15409400	2.11910000	0.42607600
H	1.61294900	2.11231100	-0.42830800
H	-0.01130900	0.74345100	-1.51385800

$SmI_2(thf)_5^+$
H_{corr} = 0.6336
$-TS$ = -0.1054
$E_{sol(THF)}$ = -25797.8734
$G_{sol(THF)}$ = -25797.3452

H	4.37747500	-3.32749800	1.22379400
H	-1.71814200	-5.21199700	1.21767000
H	-2.52352400	-4.38879700	-0.13896200
H	5.21173000	-1.92999900	-0.95739200
H	3.89483700	-3.10407000	-1.18411400
H	0.47018800	-5.03654500	0.10310800
H	-2.11117300	-2.47785900	1.31361300
H	-0.58134500	-3.17117800	1.91057900
H	-0.60291500	-5.44974600	-1.25447600
H	0.88220900	-3.12161500	-1.34809300
H	-0.79928900	-3.11941500	-1.93930700
O	-2.45106100	-0.22661400	-0.02475400
Sm	0.00240300	0.00349600	-0.00010100
O	-0.52284000	-2.39241000	-0.01341400
C	-1.24483300	-3.09228200	1.03584200
C	-1.59008200	-4.44393700	0.44356300
C	-0.40261800	-4.69950400	-0.47848100
C	-0.15946700	-3.32450300	-1.06705600
I	-0.07224900	-0.03721100	3.00630900
I	0.06566900	-0.05379700	-3.00728500
O	1.80644100	1.67130200	0.07377200
C	2.76203200	1.82992600	1.15820600
C	3.51807200	3.10574400	0.84013900
C	3.48377500	3.13347900	-0.68435500
C	2.08211200	2.63921700	-0.97272300
O	-0.99524800	2.24864000	-0.05282600
C	-0.87121000	3.23419200	1.00715500
C	-1.88122200	4.31145000	0.66544400
C	-1.88547800	4.27379800	-0.85910700
C	-1.79695200	2.78747700	-1.13822400
O	2.14032500	-1.21641400	0.01638000
C	2.52360000	-2.20041400	1.01528300
C	4.01968200	-2.35854000	0.85107900
C	4.18707000	-2.18354700	-0.65440300
C	3.21097300	-1.06350600	-0.95658700
H	3.41797400	0.94443400	1.15863400
H	2.20268900	1.85036400	2.10266000
H	1.33871500	3.44969700	-0.89663500
H	1.95687600	2.13138200	-1.93815400
H	2.99004400	3.98000300	1.25288500
H	4.53354200	3.09804600	1.25759100
H	3.66750400	4.12928800	-1.10973100
H	4.23115300	2.44167600	-1.10464900
H	0.16193000	3.61784200	0.99495500
H	-1.04632000	2.72478700	1.96429000
H	-2.78639900	2.30253700	-1.10606500
H	-1.30478200	2.52072000	-2.08262100
H	-2.87524800	4.04851300	1.06169300
H	-1.59976500	5.28789300	1.08113300
H	-2.77981100	4.72610200	-1.30769900
H	-1.00280100	4.79539700	-1.26257400
H	1.98799500	-3.13800400	0.79433500
H	2.19944000	-1.82360000	1.99434500
H	3.66228800	-0.07029700	-0.80456400
H	2.76338300	-1.09975200	-1.95862700
H	4.55335500	-1.56531700	1.39837200
C	-3.35593800	0.33211000	0.96435900
C	-3.20785200	-0.94686900	-1.03342800
C	-4.64642900	-0.52303900	-0.82119600
C	-4.69481200	-0.32307800	0.68984700
H	-3.07027200	-2.02618500	-0.85817900
H	-2.78823800	-0.68883500	-2.01470600
H	-5.35792500	-1.27420300	-1.18918700
H	-4.85051600	0.42417700	-1.34536500
H	-4.76309900	-1.29375600	1.20629300
H	-5.53538700	0.29793000	1.02593700
H	-3.39467600	1.42292200	0.81376900
H	-2.93541100	0.12655600	1.95799100

$SmI_2(MeTHF)_5^+$
H_{corr} = 0.7816
$-TS$ = -0.1142
$E_{sol(MeTHF)}$ = -25994.3792
$G_{sol(MeTHF)}$ = -25993.7117

H	2.40688300	-4.54455900	-2.01078900
H	3.94756500	-0.47107500	-2.14672100
H	5.07196400	0.89372100	-1.99779200
H	0.35979100	-5.19726200	-0.19471200
H	2.06536200	-5.17912100	0.26620200
H	5.06292800	-1.24678300	-0.17510900
H	3.42737800	2.20232100	-0.74443400

H	2.37719600	1.35288600	-1.93377000
H	5.55943400	0.38525900	0.28513800
H	3.09916700	-1.01662500	1.13523500
C	3.74685300	0.85010800	1.98749000
O	0.27790400	2.47476200	-0.05637000
Sm	0.00200900	-0.00085900	-0.00527800
O	2.44359600	0.50056900	-0.06086300
C	3.10310900	1.21821000	-1.12310700
C	4.27951100	0.33736800	-1.47982700
C	4.72255800	-0.20397400	-0.11713400
C	3.49541800	-0.05091700	0.79994800
I	0.01551500	0.00726300	-3.05219400
I	-0.00503300	-0.01412100	2.97784800
O	-1.69315700	-1.82827700	-0.07238500
C	-1.79867900	-2.78875600	-1.14249600
C	-3.26602700	-2.76624900	-1.50773200
C	-3.95083300	-2.59666100	-0.14790100
C	-2.87272800	-2.00701400	0.77981700
O	-2.26363700	1.03862100	-0.06535200
C	-3.20968400	0.84754800	-1.13616900
C	-3.64660600	2.25154800	-1.49067800
C	-3.69563100	2.94618500	-0.12623100
C	-2.79731500	2.09973700	0.79403700
O	1.22373500	-2.17296500	-0.06421900
C	2.10852400	-2.57473500	-1.12926300
C	1.63256800	-3.96368200	-1.49231300
C	1.25060700	-4.55753300	-0.13265700
C	1.02468200	-3.34705100	0.79154000
H	-1.48458900	-3.77790000	-0.76843600
H	-1.12710000	-2.46550200	-1.94672600
H	-3.12023200	-0.99564300	1.12273800
C	-2.55211600	-2.89397300	1.96140700
H	-3.46933200	-1.91372700	-2.17122900
H	-3.57636300	-3.67948400	-2.03259500
H	-4.83795200	-1.95157600	-0.20737500
H	-4.28550100	-3.56739300	0.24583200
H	-4.05127700	0.23740300	-0.76695600
H	-2.69327600	0.31701600	-1.94513400
H	-1.91197700	2.64721300	1.13766700
C	-3.53717200	1.51349400	1.97488600
H	-2.90193700	2.71520400	-2.15310300
H	-4.61244800	2.26520800	-2.01295300
H	-3.35940800	3.99058000	-0.17969400
H	-4.72141900	2.95897900	0.26994500
H	3.14468800	-2.58232200	-0.75074700
H	2.01314600	-1.83794300	-1.93579800
H	-0.01418300	-3.26626200	1.13247400
C	1.96524900	-3.31646100	1.97470100
H	0.76287600	-3.89433700	-2.16102500
C	-0.19817000	3.32093400	-1.12194300
C	1.12542900	3.30611000	0.80475900
C	1.65157700	4.42492600	-0.11265800
C	1.00280800	4.16908500	-1.47647100
H	1.92055300	2.63166700	1.14336800
C	0.34338000	3.82483700	1.99013900
H	2.74863000	4.42694400	-0.16844100
H	1.34844300	5.40323400	0.28780700
H	1.67120100	3.60455300	-2.14195700
H	0.71801200	5.09396200	-1.99540400
H	-1.03621500	3.93280700	-0.74768400
H	-0.54626900	2.67005100	-1.93273800
H	-0.48421400	4.47708800	1.66952000
H	1.01012900	4.42838700	2.62448800
H	-0.05136800	3.00184900	2.60156800
H	-3.90695000	2.33210000	2.61075400
H	-2.87831700	0.88138000	2.58565400
H	-4.41230700	0.92916000	1.64938300
H	-3.44953800	-2.99356700	2.59054900
H	-1.75137500	-2.46485000	2.57924200
H	-2.26511700	-3.90627000	1.63557500
H	1.80619300	-2.42097200	2.59066700
H	3.01719700	-3.36074700	1.65152300
H	1.77821200	-4.19891600	2.60521900
H	2.84206000	0.96976300	2.59887700
H	4.11135900	1.83961300	1.66927900
H	4.52680000	0.40100200	2.62086100

$SmI_2(thp)_5^+$
H_{corr} = 0.7859
$-TS$ = -0.1138
$E_{sol(THP)}$ = -25994.3266
$G_{sol(THP)}$ = -25993.6545

I	-0.00202300	0.02387200	-2.89267100
H	4.04484300	0.35780900	-0.35153400
H	2.73745800	0.29380500	-1.55948400
H	5.10232100	-1.14862900	1.02598900
H	4.65693200	-2.72649200	1.66106500
H	4.85724100	-1.07736100	-2.08357100
O	2.36891100	-0.75203100	0.14615600
C	2.97547100	-1.49245600	1.24154200
C	4.37733400	-1.97061700	0.91174200
C	3.99578600	-1.47654700	-1.52523600
C	3.31566600	-0.30419200	-0.84470100
C	4.43809300	-2.53726600	-0.50065100
Sm	0.00433000	0.00127400	0.12915500
H	3.31794200	-1.91080300	-2.27519700
H	3.77743000	-3.41710900	-0.55952200
H	5.45046600	-2.90189700	-0.72371200
H	2.30527000	-2.34535300	1.42234800
H	2.95264800	-0.85394100	2.13815800
I	0.01663100	-0.00590000	3.11773400
H	1.58898900	-3.72195700	-0.38123500
H	1.09754800	-2.50376500	-1.58442200
H	0.51106000	-5.19093800	1.02181500
H	-1.12048500	-5.27059100	1.67209700
H	0.45913500	-4.94377300	-2.10053500
O	0.01429600	-2.47979800	0.13718500
C	-0.49246100	-3.28528700	1.23713600
C	-0.50078600	-4.76778000	0.91436600
C	-0.18355800	-4.25587400	-1.52875700
C	0.72348400	-3.23962900	-0.86225300
C	-1.03081500	-5.01573300	-0.49166900
H	-0.82380400	-3.74971100	-2.26656600
H	-2.07917100	-4.68066800	-0.54229300
H	-1.04589800	-6.09285900	-0.70905900
H	-1.51339900	-2.91998200	1.42006900
H	0.10811100	-3.05797200	2.13145400
H	0.89991000	3.96880400	-0.33332800
H	0.55690000	2.71110900	-1.54783600
H	2.66631200	4.50505900	1.04159400
H	4.02201600	3.58411100	1.67845600
H	2.52083200	4.30751000	-2.05855500
O	1.43678300	2.02952200	0.15450000
C	2.32025100	2.37814600	1.25629500

C	3.21747600	3.55756200	0.92813200
C	2.63010600	3.35907600	-1.50918900
C	1.30381800	3.07310300	-0.83118400
C	3.77566300	3.43757400	-0.48378600
H	2.83047600	2.58619800	-2.26627500
H	4.39885300	2.53095000	-0.54465000
H	4.44413900	4.28146100	-0.70433900
H	2.91682600	1.47443800	1.44716300
H	1.69930000	2.56217200	2.14671600
H	-3.49731800	2.06342900	-0.33865100
H	-2.39964100	1.36266500	-1.55326500
H	-3.47280000	3.89820600	1.05152700
H	-2.18699900	4.91247500	1.69152700
H	-3.32317700	3.71724300	-2.05796700
O	-1.48674000	1.98539500	0.15424800
C	-1.54765200	2.92992600	1.25820500
C	-2.40398400	4.14107900	0.93731000
C	-2.38919300	3.53381600	-1.50366200
C	-2.51971900	2.18004000	-0.83244300
C	-2.12513100	4.64582800	-0.47231000
H	-1.58760600	3.49513800	-2.25644800
H	-1.07530800	4.97463400	-0.53237500
H	-2.73454300	5.53464100	-0.68753700
H	-0.50527500	3.22588100	1.44502200
H	-1.90578000	2.39158400	2.14951300
H	-3.04313100	-2.67341400	-0.35742800
H	-2.04582400	-1.83226100	-1.57088300
H	-4.76699600	-2.10155000	1.05170300
H	-5.34267000	-0.57334400	1.70318700
H	-4.56860000	-1.98687800	-2.06770700
O	-2.34908800	-0.78785400	0.14752500
C	-3.26303400	-0.55794100	1.25516900
C	-4.67779600	-1.00864400	0.94196000
C	-4.10920000	-1.15949700	-1.50411900
C	-2.85660400	-1.70300500	-0.84393700
C	-5.08614000	-0.58392100	-0.46245700
H	-3.83180300	-0.39811600	-2.24838200
H	-5.09001800	0.51664900	-0.51538600
H	-6.11709600	-0.90129300	-0.67258600
H	-3.22985200	0.52545500	1.44015300
H	-2.85419200	-1.06068900	2.14530500

$SmI_2(HMPA)_4^+$

H_{corr} = 1.1018

$-TS$ = -0.1741

$E_{sol(THF)}$ = -27919.2758

$G_{sol(THF)}$ = -27918.3482

H	1.81518400	4.82933500	2.61732200
H	0.30937900	5.20396000	3.47966500
C	-1.31932200	3.73795400	1.95331100
H	-2.76008100	0.61767000	2.53455000
N	2.41202800	3.77916100	0.39891400
C	3.14078100	2.82211100	1.21227300
H	-5.10424100	3.34044700	1.84240800
C	0.82785100	4.43505600	2.88525100
P	3.68697700	-0.78772600	-0.45466000
O	2.26865700	-0.20543500	-0.55339900
H	3.52249900	3.28658300	2.13886900
H	-1.85509800	3.55986000	1.01322700
Sm	0.03646100	-0.02130800	-0.16579100
N	3.73009000	-2.20190000	-1.36164200
C	3.02388100	-2.20063300	-2.63727200
H	2.54222600	5.59163600	-0.65357600
H	3.42641800	5.58177300	0.90058600
C	4.87801000	-3.09142300	-1.32609700
I	0.50070100	0.10962300	2.91833200
I	-0.53193400	-0.01834300	-3.18732800
N	4.74315900	0.42526300	-0.95130800
C	4.24592000	1.43366300	-1.87777300
H	-5.49421700	2.75990900	0.20517300
H	-6.22094300	1.96433000	1.63195000
C	6.14330900	0.08328600	-1.14115200
N	0.19655000	4.68383200	-0.91721300
N	-4.68311500	-0.50000800	-0.42894800
C	0.36759500	4.30360000	-2.31046800
H	-1.33703500	2.80771200	2.54537900
H	-1.84865700	4.53177700	2.50497300
N	4.25751600	-1.25966800	1.03914100
C	4.73708700	-0.26520100	1.99139600
H	2.48834300	1.98364700	1.49233200
H	3.99315900	2.41924600	0.64438300
C	3.60539600	-2.39024300	1.69534500
H	3.20251600	1.67459300	-1.64492700
H	4.85590600	2.34482600	-1.76666800
H	0.95513300	3.53353200	3.50978700
H	3.69080600	-1.91944300	-3.47240100
H	5.59297500	-2.87932000	-2.14128600
H	4.31194900	1.10574600	-2.93132800
H	6.45295500	-0.67668100	-0.41162900
H	6.34971200	-0.29652900	-2.15870000
H	-0.23050200	6.24935500	0.40299500
H	0.62196900	6.76020900	-1.08806400
H	6.76331600	0.97952600	-0.98087200
H	-0.52888800	4.59288700	-2.88141300
H	5.49160100	-0.73548100	2.64118600
C	-0.13859300	6.07224400	-0.67549000
H	1.24071800	4.80290500	-2.76862000
H	0.48557400	3.21519400	-2.39442200
H	5.20364100	0.57373400	1.46404900
H	3.91511400	0.11254800	2.62369800
H	2.77075000	-2.05014300	2.33193600
H	-1.10311800	6.30999200	-1.15396600
C	3.14113700	4.97111500	0.02378600
H	4.06569800	4.68577600	-0.50486100
H	3.21597000	-3.08934500	0.94860500
H	4.34423900	-2.91647200	2.32019600
H	2.63566900	-3.21179900	-2.83863500
H	2.17281800	-1.50795000	-2.61178600
H	-3.14128200	3.78012800	-1.44872300
N	0.04749000	4.16138400	1.69042500
H	-5.32568800	2.76834800	-2.35678700
H	-5.33227800	-1.95508900	0.97242500
H	5.40117400	-3.00589000	-0.36604300
H	4.53471800	-4.13279200	-1.44288700
H	-5.20739400	-1.59076000	-2.15405400
H	-3.58981100	2.02185700	3.24474300
O	-2.18986600	0.29190200	0.18290900
P	-3.63773100	0.75220100	-0.08252500
N	-3.74564600	1.75352900	-1.39981300
C	-2.73347800	2.76729400	-1.62138600
H	-5.84634000	-0.27834000	1.30545800
H	-6.61593400	-1.19780900	-0.01003300
C	-4.84969900	1.77204100	-2.33780300
H	-1.87604000	2.60702000	-0.95863100

H	-2.35913800	2.69702500	-2.65408700
C	-5.66130000	-1.00767200	0.50823000
H	-3.53802400	-0.94679200	-2.13981400
H	-3.94317000	-2.35052600	-1.12615500
C	-4.32785900	-1.39473600	-1.51855400
H	-4.48942300	1.54206800	-3.35460000
H	-5.60482700	1.03074700	-2.05318100
N	-4.15412800	1.56102000	1.28441700
C	-3.72514000	1.13556100	2.60517300
O	0.36335600	2.21980400	-0.13411700
P	0.75480700	3.65489400	0.26651700
C	-5.30400900	2.44054600	1.23810400
H	-4.46368300	0.47424500	3.09556400
P	-0.74252500	-3.58431200	0.41900600
O	-0.03897300	-2.32203700	-0.11355600
N	-2.04029400	-3.91283400	-0.60766700
C	-1.76174600	-3.78947700	-2.03530100
C	-2.96866700	-4.98666100	-0.29056700
N	0.40670200	-4.80645400	0.51485100
C	1.55171800	-4.76714000	-0.37660100
C	-0.01222700	-6.14879400	0.88198600
N	-1.42879800	-3.54144600	1.93239900
C	-0.62229700	-3.74278300	3.13137900
C	-2.58066700	-2.67441000	2.14590100
H	1.80491400	-3.72786300	-0.61376500
H	-1.27705600	-4.69666200	-2.44242700
H	-2.63982700	-5.96134000	-0.69441500
H	1.36499600	-5.31765900	-1.31772200
H	-0.88787400	-6.11215700	1.54230400
H	-0.26040900	-6.76402500	-0.00213300
H	0.80333800	-6.65095000	1.42706200
H	-1.20070100	-4.32934400	3.86331100
H	0.29417900	-4.28912500	2.88229200
H	-0.34264300	-2.77504300	3.57890700
H	-2.27120500	-1.74710900	2.65546800
H	-3.03673500	-2.40527200	1.18584400
H	-3.32919200	-3.19216300	2.76837700
H	-2.71018400	-3.64536200	-2.57446400
H	-1.12819100	-2.91427700	-2.22861300
H	-3.09279800	-5.07819100	0.79544700
H	-3.95153600	-4.75553600	-0.73315600
H	2.41404800	-5.23831600	0.12352000

$SmI_2(TPPA)_4^+$
H_{corr} = 1.5578
$-TS$ = -0.1928
$E_{sol(THF)}$ = -28848.0149
$G_{sol(THF)}$ = -28846.6499

H	2.04095400	2.58596900	-3.16067300
H	3.63421200	3.34945500	-3.44508100
C	4.28915900	0.82806500	-1.69781900
C	4.73247200	0.65733500	-3.14293200
H	2.71824900	0.48777700	-3.92355700
H	4.96019100	-0.38892000	-3.38879100
C	3.54572200	1.21521900	-3.91780600
C	3.12770800	2.43235500	-3.09944100
P	-2.80693800	2.53106100	0.54070500
O	-1.77592900	1.44677000	0.18562800
C	5.55779700	3.35568700	2.15127500
H	5.13304700	0.86869300	-0.99371000
Sm	-0.04231000	-0.01516700	-0.00018500
N	-4.01620600	1.78938600	1.41860000
C	-3.69376500	0.59832000	2.23673100
C	-4.68740300	0.66326300	3.39702900
C	-5.00687100	2.15039900	3.52016400
C	-5.07485700	2.57867500	2.06155300
I	-0.35392100	0.29330800	-3.08169500
I	0.18809000	-0.18662700	3.11445500
N	-1.96021300	3.78687600	1.24346300
C	-0.73741500	3.58698400	2.02532900
C	-0.18115800	5.01334700	2.20804500
C	-1.27161500	5.95873900	1.67122300
C	-2.51922300	5.08386400	1.60105700
N	3.74865400	2.65261200	0.82917800
H	5.63815200	1.25614800	-3.33198200
C	3.24502600	2.78257000	2.21004600
H	3.62535800	0.00301200	-1.38691200
H	6.34187100	4.11731100	2.25957500
N	-3.62133200	3.30444800	-0.68626000
C	-2.81705300	3.99743100	-1.71597100
C	-3.72902500	4.03702300	-2.93808500
C	-4.56035400	2.76959600	-2.78050500
C	-4.85105500	2.76181500	-1.28726400
H	-0.95246700	3.11702300	3.00045200
H	-0.05040000	2.92388800	1.48600300
H	0.76141100	5.14042000	1.65577000
H	0.03249100	5.20723000	3.26853400
H	-1.01726600	6.30631800	0.65789100
H	-1.41945800	6.84758400	2.29981800
H	-3.25418300	5.40997400	0.85258600
H	-3.02191900	5.04995500	2.58773900
H	5.75839300	2.50188100	0.13896300
H	5.12412000	4.15949400	0.17647900
H	3.99557600	4.75412800	2.69329900
H	4.31293900	3.55045400	3.96412500
H	5.96170000	2.40314100	2.53031500
C	5.11120000	3.19001200	0.70215600
H	2.22461900	3.18928700	2.22614900
H	3.20071600	1.79208100	2.69226200
H	-2.51886400	5.00080300	-1.37538700
H	-1.90768900	3.41775500	-1.93830300
H	-3.15596600	4.07276900	-3.87429000
H	-4.38061400	4.92488900	-2.90246600
H	-3.94994800	1.89137500	-3.04619600
H	-5.47249400	2.76210400	-3.39336700
H	-5.07852600	1.76927600	-0.87936100
H	-5.70108700	3.42528000	-1.04907500
H	-3.80120000	-0.31921000	1.63929600
H	-2.65352200	0.63020900	2.59634500
H	-4.26956300	0.22971900	4.31588800
H	-5.60648000	0.10650800	3.15042600
H	-4.17901900	2.67683200	4.02358700
H	-5.93336900	2.35825100	4.07304900
H	-4.92110000	3.65612500	1.90806900
H	-6.05589600	2.32235800	1.62228100
C	4.26119000	3.69980200	2.87706900
H	3.78070200	1.47518400	-4.95875100
O	1.63078200	-1.54444800	-0.10937900
P	2.67999300	-2.56922400	0.36267500
N	3.67870200	-2.10848000	1.60830300
C	4.45773400	-0.86593300	1.50972700
C	5.10235100	-0.75813900	2.88377700
C	4.01158000	-1.29501100	3.80278900
C	3.42564900	-2.46481700	3.01860800

H	5.19527900	-0.93306600	0.69521800
H	3.80510300	-0.00006800	1.30605700
H	5.40561100	0.27075500	3.12249000
H	5.99999300	-1.39598000	2.93088600
H	3.22746800	-0.53451800	3.94516000
H	4.37964400	-1.60030300	4.79164000
H	2.35363500	-2.57799400	3.23036100
H	3.93795700	-3.41183300	3.26135100
N	3.71951800	-2.83868500	-0.90351400
C	3.22231300	-2.86362500	-2.28906100
C	4.39491100	-3.44239200	-3.08679500
C	5.61316200	-3.19146000	-2.20038000
C	5.05717400	-3.42069600	-0.80301800
H	2.31729300	-3.48344100	-2.38790200
H	2.95049200	-1.84573400	-2.60975000
H	4.47964200	-2.99113500	-4.08506100
H	4.25800600	-4.52629300	-3.22705200
H	5.95353400	-2.14768200	-2.29417700
H	6.46270200	-3.84836200	-2.43170300
H	5.63880100	-2.93061700	-0.01056900
H	5.02488100	-4.50228700	-0.57410600
N	1.90155300	-3.97334100	0.80165300
C	0.63544400	-3.98006400	1.56222100
C	0.78513800	-5.18553800	2.47928200
C	1.60805700	-6.15113400	1.63229000
C	2.63288000	-5.23600100	0.96811000
H	0.47674200	-3.03419400	2.10131900
H	-0.21077100	-4.11185100	0.86996400
H	-0.18430100	-5.59775300	2.79284700
H	1.34302000	-4.90796200	3.38836500
H	0.96872000	-6.61479200	0.86295400
H	2.08132900	-6.95782500	2.20825000
H	2.97540500	-5.62466100	-0.00327000
H	3.52162300	-5.10963400	1.61335500
O	1.49036600	1.65614400	0.00049100
P	2.65192500	2.57794700	-0.41500800
N	1.98052700	4.06477400	-0.74779900
C	0.63908300	4.18144100	-1.34972100
C	0.75056000	5.42343600	-2.22438000
C	1.75785300	6.28513300	-1.46944100
C	2.78710200	5.26531300	-0.99237500
H	0.36780100	3.27779600	-1.91616800
H	-0.11770800	4.30985700	-0.56056700
H	-0.21890100	5.91808700	-2.37726100
H	1.15123700	5.15473100	-3.21527800
H	1.27395100	6.76034300	-0.60036500
H	2.20867500	7.07912400	-2.07992000
H	3.30108100	5.58956900	-0.07467000
H	3.55577500	5.08865900	-1.76730700
N	3.56973600	2.11323200	-1.72286100
P	-2.58549400	-2.73725100	-0.44623900
N	-3.73088500	-2.22773400	-1.53895000
C	-4.57481600	-1.06258800	-1.23592900
C	-5.33044600	-0.85316900	-2.53896400
C	-4.26291300	-1.15709600	-3.58343200
C	-3.51458800	-2.35102700	-2.99658400
H	-5.23593900	-1.26922300	-0.38093000
H	-5.75387400	0.15641200	-2.62550100
H	-6.15980900	-1.57562100	-2.60940300
H	-3.56652400	-0.30808400	-3.67115600
H	-4.66960700	-1.37053500	-4.58111700
H	-2.44882300	-2.31138400	-3.26315800
H	-3.93218200	-3.30787700	-3.35273400
N	-3.46826700	-3.13049300	0.89828600
C	-2.88914400	-3.12967400	2.24587100
C	-4.11559100	-3.22354400	3.17719100
C	-5.33763400	-3.19763100	2.24984600
C	-4.79163400	-3.73643200	0.93424100
H	-4.13375800	-2.40399300	3.90827600
H	-4.08614900	-4.16351200	3.74732100
H	-5.68383700	-2.16296200	2.09640400
H	-6.18323800	-3.78426100	2.63463800
H	-5.37432200	-3.45272000	0.04821800
H	-4.73070800	-4.84185700	0.96019100
H	-3.37133400	-5.18179800	-2.02225800
O	-1.50928900	-1.72160400	-0.02837900
H	-3.95667900	-0.18218300	-0.98498500
H	-2.22370200	-3.99810300	2.39353800
H	-2.28600400	-2.22330500	2.39632900
C	-2.48712600	-5.31853700	-1.37225100
H	-2.82804200	-5.78502700	-0.43523200
N	-1.80593900	-4.04849700	-1.10811300
C	-0.56381200	-3.91813700	-1.89493400
C	-0.65046200	-5.08270400	-2.87330900
C	-1.41591200	-6.13972700	-2.08235900
H	-0.49147600	-2.93877800	-2.39178400
H	0.30943700	-4.01391800	-1.22960600
H	0.33929600	-5.42071500	-3.21127200
H	-1.22787700	-4.78777800	-3.76462800
H	-0.75174100	-6.60847700	-1.33743200
H	-1.84327700	-6.93752000	-2.70468100

$SmI_2(TMTHF)_4^+$
H_{corr} = 0.9715
$-TS$ = -0.1331
$E_{sol(TMTHF)}$ = -26194.2158
$G_{sol(TMTHF)}$ = -26193.3774

I	-0.55683428	2.00031572	-1.49104321
Sm	0.61562523	0.50607016	0.71153143
I	0.57965070	-2.32528859	1.37708751
H	2.96084000	-2.26697782	-4.92669818
H	1.68223815	-2.00619004	-6.13568546
H	3.15284743	-3.50221964	-2.64210357
H	2.08692456	-4.88999696	-2.28971704
O	1.20855071	-1.69487593	-2.88220890
C	1.04688195	-3.12829268	-3.01639354
C	1.16583586	-3.41787075	-4.52471858
C	1.87118961	-2.18598594	-5.06803666
C	1.32372441	-1.07144742	-4.17516807
C	-0.31090636	-3.52678904	-2.46091963
C	2.17238090	-3.79430099	-2.23622789
C	2.27639705	0.10225852	-4.04283456
H	2.13275285	-3.50507527	-1.17581040
C	-0.04827442	-0.59286165	-4.64346272
H	1.71240327	-4.35094640	-4.71932278
H	0.16726173	-3.52667222	-4.97390453
H	2.38008959	0.62543034	-5.00494168
H	3.27355168	-0.24404195	-3.73286168
H	1.89934553	0.82423523	-3.30354350
H	0.02948025	-0.05287176	-5.59937394
H	-0.48487839	0.08467554	-3.89629811
H	-0.74345794	-1.43278759	-4.78342685
H	-0.40081655	-3.24036370	-1.40332242
H	-0.45800664	-4.61516905	-2.53770072

H	-1.11597505	-3.02901938	-3.01915669
H	4.81647700	2.40427409	1.83648502
H	5.42871421	3.17645005	0.35542373
H	4.28360308	0.11943052	2.62505933
H	4.97387208	-1.31363639	1.82704732
O	2.91087934	1.02717395	0.46388077
C	4.07805091	0.06958040	0.46032418
C	5.29044668	0.98506514	0.26173549
C	4.81795007	2.34735139	0.73669110
C	3.39197498	2.41479997	0.21258641
C	3.86382240	-0.91027070	-0.67055994
C	4.13015568	-0.60861438	1.81553426
C	2.49171525	3.35119631	0.98808392
H	3.22161427	-1.19499729	2.01385600
C	3.33958503	2.71101090	-1.27671403
H	6.15426605	0.61210620	0.82719959
H	5.58071912	1.01702074	-0.79757528
H	2.85396064	4.38392440	0.88395058
H	2.47729361	3.10552125	2.05951067
H	1.46545790	3.34941751	0.58529498
H	3.68918632	3.73870922	-1.45398508
H	2.31312971	2.63265477	-1.65936283
H	3.97658016	2.03105789	-1.85623896
H	2.96148819	-1.52052154	-0.52376583
H	4.71944354	-1.59963990	-0.72628046
H	3.77171558	-0.39287334	-1.63326410
H	-5.25329937	-2.46084138	-1.52744897
H	-6.27479226	-1.05825285	-1.17885649
H	-3.51407509	-3.61762892	-0.61225094
H	-3.63970270	-4.23632831	1.05151820
O	-3.28240531	-0.99371685	0.13780864
C	-3.85511800	-2.07570279	0.89758531
C	-5.34979426	-1.98490788	0.58741193
C	-5.35561276	-1.57914303	-0.87715852
C	-4.11699302	-0.67231351	-1.00301548
C	-3.53961214	-1.83539009	2.36220233
C	-3.26267012	-3.40504221	0.43647585
C	-3.33449332	-0.94215266	-2.27790873
H	-2.16752070	-3.37927816	0.52156563
C	-4.49889010	0.79774020	-0.89619036
H	-5.88241358	-2.92570819	0.78526655
H	-5.80708272	-1.20014785	1.21111151
H	-3.93851390	-0.69529347	-3.16467103
H	-3.04992992	-2.00187723	-2.33732997
H	-2.41556230	-0.33959065	-2.30870570
H	-5.12115689	1.10078629	-1.75157471
H	-5.06477245	0.98597808	0.02879287
H	-3.94519775	-2.64858752	2.98213404
H	-3.98746176	-0.89010520	2.70310662
H	-2.45210122	-1.79830157	2.52212928
H	-3.60071177	1.43212966	-0.89270348
H	-2.38104062	2.13087267	4.66124996
H	-1.80568604	3.79426630	4.47216130
H	-1.74330812	0.05454893	4.50587762
H	-0.34136404	-0.43786536	5.48243105
O	-0.30313570	1.48842479	2.67025731
C	0.12458255	1.08461004	4.03741946
C	-0.22825802	2.31945389	4.85202234
C	-1.56141514	2.74478270	4.26251487
C	-1.40202604	2.51602888	2.75589370
C	1.60594856	0.78450722	3.97348940
C	-0.66367358	-0.13894677	4.47418033
C	-2.62973894	1.93160059	2.09519493
H	-0.48454588	-0.98535099	3.79725508
C	-0.91700359	3.77581413	2.06460556
H	-0.27941071	2.09541178	5.92578410
H	0.53625693	3.09808919	4.70364699
H	-3.46707441	2.63990335	2.18217433
H	-2.92405259	0.98604174	2.56696402
H	-2.47131934	1.74441180	1.02360252
H	-1.70978738	4.53617882	2.11308813
H	-0.70676638	3.60078912	0.99948524
H	1.81026434	-0.10361726	3.35241191
H	1.97957304	0.54055852	4.97819544
H	2.17871038	1.64525149	3.59997291
H	-0.02151451	4.18513862	2.55386160

SET of SmI_2LB_{n-1}-Ketone

ACE

H_{corr} = 0.0899
$-TS$ = -0.0335
$E_{sol(THF)}$ = -193.0971
$G_{sol(THF)}$ = -193.0407

C	0.00000000	-0.00000000	0.18380100
C	0.00000000	-1.28335500	-0.61103000
C	-0.00000000	1.28335500	-0.61103000
O	0.00000000	-0.00000000	1.39474300
H	0.88415900	1.32673000	-1.26723600
H	-0.00000000	2.14257700	0.07027600
H	-0.88415900	1.32673000	-1.26723600
H	-0.88415900	-1.32673000	-1.26723600
H	0.88415900	-1.32673000	-1.26723600
H	0.00000000	-2.14257700	0.07027600

ACE^{-}

H_{corr} = 0.0851
$-TS$ = -0.0341
$E_{sol(THF)}$ = -193.1275
$G_{sol(THF)}$ = -193.0765

C	-0.00000700	0.17247400	-0.22265500
C	-1.26347300	-0.62481900	0.02048600
C	1.26343100	-0.62487200	0.02047900
O	0.00004600	1.43191700	0.05792700
H	1.45585300	-0.83011000	1.11967500
H	2.14172500	-0.05091300	-0.32771500
H	1.27132900	-1.61510800	-0.47858900
H	-1.27156800	-1.61485300	-0.47895900
H	-1.45567200	-0.83044600	1.11963700
H	-2.14173800	-0.05060200	-0.32733000

HMAC

H_{corr} = 0.2674
$-TS$ = -0.0500
$E_{sol(THF)}$ = -428.8543
$G_{sol(THF)}$ = -428.6369

H	-2.23578300	-1.26506100	1.47392900
H	2.71784500	0.87951000	1.50091200
H	1.59518400	-0.34940800	2.12907200
H	-2.44268600	-1.79511000	-0.20306900
H	1.02335900	1.30939200	1.81187100
C	1.68129300	0.51016000	1.44679100

C	1.44599000	1.31490700	-0.92237800
H	-3.42298900	-0.43768800	0.42894500
C	2.43055500	-0.91332300	-0.44882900
H	-1.02411400	1.30849100	-1.81254300
H	-2.71842200	0.87828000	-1.50098000
O	0.00007000	-1.84446100	-0.00005000
H	2.45902300	1.74372000	-0.86202700
H	0.74033600	2.11123800	-0.65178700
H	-1.26862500	1.03994100	1.97214500
H	-2.45912600	1.74401700	0.86109400
H	-0.74052900	2.11171500	0.65045200
H	1.26873200	1.03868500	-1.97271800
H	3.42327300	-0.43755200	-0.42787000
H	2.23659200	-1.26559100	-1.47290500
H	2.44289500	-1.79483000	0.20440400
C	0.00006900	-0.63053700	-0.00004100
C	-1.36844200	0.09423500	0.00135700
C	1.36848900	0.09427600	-0.00133700
C	-1.44600900	1.31542700	0.92163400
C	-2.43018900	-0.91330300	0.44976400
C	-1.68175300	0.50925100	-1.44691700
H	-1.59553600	-0.35062400	-2.12879400

HMAC$^-$

H_{corr} = 0.2625
$-TS$ = -0.0503
$E_{sol(THF)}$ = -428.8853
$G_{sol(THF)}$ = -428.6732

H	3.45232300	-0.52684100	-0.31219500
C	-1.36481000	0.13007000	-0.03823600
C	1.60687600	1.49083400	-0.66880200
H	0.93399900	0.94915600	1.94893800
H	2.64495600	0.48604500	1.74943500
H	1.38339100	-0.77897200	1.92902300
C	2.43206100	-0.83065800	-0.61304000
C	1.60455800	0.20569100	1.48551000
C	-2.35031200	-0.49519200	-1.03701200
H	-3.40115800	-0.22771000	-0.81163400
C	-1.45162900	1.64755900	-0.17988500
O	-0.02913200	-1.83722400	-0.15250100
H	1.05095800	2.29795700	-0.17117200
H	-2.21947600	-1.58637000	-0.99735600
C	-1.82378500	-0.26523800	1.37788100
H	1.30021600	1.47085900	-1.72852000
H	2.67903100	1.76097000	-0.62326700
H	2.37527600	-0.82974200	-1.71512700
H	-2.10927200	-0.16337400	-2.06128500
H	2.20675300	-1.85129600	-0.27576900
H	-2.50088000	1.98052700	-0.06815200
H	-1.09812000	1.97990000	-1.16990300
H	-0.86305600	2.17322100	0.58916000
H	-2.89931500	-0.06422000	1.55109600
H	-1.25238000	0.27764100	2.14957700
H	-1.62313600	-1.34232800	1.49304100
C	0.00251000	-0.55060300	-0.33522500
C	1.37335500	0.11626500	-0.03383700

CPO

H_{corr} = 0.1279
$-TS$ = -0.0353
$E_{sol(THF)}$ = -270.4835
$G_{sol(THF)}$ = -270.3909

H	0.41238300	2.06134100	0.47625100
H	0.41268500	-2.06182700	-0.47488400
C	-1.37082200	-0.73054800	-0.23606000
H	0.07230400	-1.54374100	1.18343700
H	-2.17842600	-1.32616900	0.21235400
H	-1.50818200	-0.76548500	-1.33037100
H	-2.17856000	1.32587400	-0.21286900
H	-1.50877400	0.76516500	1.33005000
C	0.92111700	-0.00004800	0.00000500
C	0.02590800	1.22717400	-0.12529400
C	0.02604900	-1.22694300	0.12560700
O	2.12704500	0.00002300	-0.00017800
C	-1.37099200	0.73038400	0.23579500
H	0.07265100	1.54454600	-1.18286000

CPO$^-$

H_{corr} = 0.1225
$-TS$ = -0.0367
$E_{sol(THF)}$ = -270.5130
$G_{sol(THF)}$ = -270.4271

H	0.38394000	-2.11610400	-0.29109800
H	0.31760900	1.91490800	0.84388200
C	-1.39416200	0.73214800	0.13355300
H	0.18189100	1.88389500	-0.91563000
H	-2.13793500	1.31251800	-0.44508200
C	0.04669300	1.23641300	-0.01463600
C	-1.35775000	-0.74971500	-0.26836900
H	-1.70762300	0.80004900	1.19468200
H	-0.06595600	-1.49499100	1.31350800
H	-2.20240600	-1.31613400	0.17878900
H	-1.42925000	-0.85404100	-1.36609900
O	2.17518900	-0.01855300	-0.06331100
C	0.89911700	-0.01423500	-0.06616500
C	0.01580600	-1.20155600	0.21454000

$SmI_2(thf)_4$-ACE

H_{corr} = 0.5994
$-TS$ = -0.1114
$E_{sol(THF)}$ = -25758.7163
$G_{sol(THF)}$ = -25758.2282

H	-4.31341900	-1.60047300	3.18346700
H	-3.48992700	2.67023900	2.00873800
H	-3.48116000	4.19073500	1.10367700
H	-4.55190800	-3.03626300	0.87361300
H	-5.04347000	-1.32553400	0.84688500
H	-3.84382400	1.36403700	0.05251600
H	-1.17489800	4.11026100	0.83565400
H	-1.09092800	2.63328200	1.84267700
H	-4.49515700	2.87665300	-0.63242700
H	-2.32191800	1.66339100	-1.82446100
H	-2.30050600	3.44305900	-1.58141500
O	1.60601500	1.90956200	-0.87549700
Sm	0.08004600	0.00218400	-0.15512100
O	-1.29432900	2.30091600	-0.16767000
C	-1.59536300	3.09841000	0.98378700
C	-3.12890700	3.14908400	1.08782500
C	-3.61044000	2.41435100	-0.17344100
C	-2.38928900	2.46286700	-1.07269700
I	0.99786100	0.67899200	2.87990300
I	-0.88653800	-0.61123700	-3.12276400
O	0.08769900	-2.55096800	0.32753700
C	0.16601300	-3.11219900	1.64412500

C	1.17946800	-4.23251300	1.52514800
C	0.85097300	-4.78340300	0.13983600
C	0.52768100	-3.52009600	-0.64795200
O	2.44717000	-0.96006200	-0.72177700
C	3.46116000	-1.07315400	0.27642200
C	4.36027100	0.12609400	0.02482600
C	4.29882000	0.29624600	-1.50433300
C	3.11871400	-0.58868400	-1.93077800
O	-2.28431700	-0.64513600	0.76115800
C	-2.74741700	-0.50914600	2.11594000
C	-3.57440800	-1.75202700	2.38457100
C	-4.19345500	-2.00707300	1.01356200
C	-3.04357200	-1.66624900	0.08086700
H	-0.82758700	-3.50494600	1.93197500
H	0.44684500	-2.30668200	2.33901900
H	1.41204700	-3.09894300	-1.14787900
H	-0.26696700	-3.64673800	-1.39722200
H	2.20048900	-3.81889700	1.54865100
H	1.08910300	-4.97587700	2.32921700
H	1.67272100	-5.35484600	-0.31336800
H	-0.02884900	-5.44437200	0.19551000
H	4.00095100	-2.03029900	0.13793300
H	2.97006000	-1.06100000	1.25944200
H	2.38033700	-0.09517600	-2.57577500
H	3.46426000	-1.50886200	-2.43489700
H	3.93021900	1.00122200	0.53246200
H	5.38001200	-0.02820500	0.40397500
H	4.12890100	1.34517800	-1.78061500
H	5.22752600	-0.03249800	-1.99272200
H	-3.36988800	0.40081100	2.18624000
H	-1.86935500	-0.38413200	2.76619400
H	-2.37718300	-2.52771500	-0.08598800
H	-3.34762000	-1.27486300	-0.89982700
H	-2.92207600	-2.59078500	2.67629300
C	1.51033900	3.12959900	-0.85347400
C	0.93025300	3.87652100	-2.01290900
C	1.99395200	3.90380600	0.33530200
H	0.01572000	4.39194600	-1.67835400
H	0.68579600	3.18610100	-2.82926700
H	1.62808800	4.65763300	-2.35444700
H	1.90032000	3.28692200	1.24146500
H	1.46972700	4.86255000	0.45015300
H	3.06387100	4.12759500	0.18226700

$SmI_2(thf)_4^+$-ACE$^-$

H_{corr} = 0.5982
$-TS$ = -0.1067
$E_{sol(THF)}$ = -25758.6937
$G_{sol(THF)}$ = -25758.2021

H	4.40932400	-2.24142500	2.55525400
H	-0.30344700	-4.61809400	2.44031900
H	-1.98609900	-4.85147600	1.90213700
H	5.30061100	-1.75905900	0.02098600
H	3.98725600	-2.94509300	0.22487300
H	-0.00175900	-5.59110400	0.18862100
H	-2.19525400	-2.53944000	1.19341100
H	-1.10268900	-2.31765100	2.59988700
H	-1.32213400	-4.58127700	-0.46153500
H	1.42296700	-3.64570200	0.53301000
H	0.58388800	-3.17074000	-0.98267500
O	-2.20324300	-0.44824600	-0.15424500
Sm	-0.16318600	0.00397100	-0.05679000
O	-0.18061000	-2.34591000	0.75031200
C	-1.22605600	-2.82544200	1.63335100
C	-1.04505500	-4.33116700	1.67697800
C	-0.50746900	-4.61985600	0.27874100
C	0.43859200	-3.45445200	0.06908400
I	-0.41432200	1.00048400	2.85739200
I	0.44289000	-0.90709300	-2.94327100
O	1.63003600	1.82728200	-0.34929400
C	2.47627800	2.35521800	0.69049200
C	3.08374300	3.61772700	0.10866000
C	3.19949200	3.25219400	-1.36760700
C	1.90453900	2.49747900	-1.59887500
O	-1.07916400	2.12009900	-1.03465900
C	-1.36507900	3.25798900	-0.21266600
C	-2.85951300	3.15981800	0.03131300
C	-3.39323100	2.58007900	-1.29020000
C	-2.14218900	2.05000300	-2.00101900
O	2.18380400	-0.83319100	0.57592700
C	2.56308700	-1.27558300	1.89641200
C	4.06543300	-1.48740000	1.83397900
C	4.26959100	-1.89076700	0.37703600
C	3.29143200	-0.97566000	-0.33330800
H	3.25086000	1.60480500	0.92481300
H	1.85992200	2.50762200	1.58707500
H	1.06581100	3.17743200	-1.81699200
H	1.94774600	1.73379700	-2.38765800
H	2.39691100	4.46938900	0.23973300
H	4.04220600	3.87590200	0.57969500
H	3.30001400	4.12062000	-2.03307200
H	4.06717400	2.59284900	-1.53343600
H	-1.09166000	4.17480200	-0.76887400
H	-0.75516100	3.17908800	0.69682900
H	-2.21224900	1.00661700	-2.32924300
H	-1.85648900	2.67797700	-2.86188300
H	-3.03675700	2.46165300	0.86250400
H	-3.30556000	4.12951400	0.29186100
H	-4.11741200	1.77583800	-1.10621400
H	-3.89043400	3.34595100	-1.90260600
H	2.02049000	-2.21034800	2.11203100
H	2.23494700	-0.51470900	2.61782800
H	3.72864200	0.02196100	-0.51484800
H	2.90018400	-1.35996000	-1.28503000
H	4.59736500	-0.54605000	2.04770900
C	-3.43341400	-0.97224500	-0.11059000
C	-4.00018400	-1.44107000	-1.40773100
C	-4.32183700	-0.49335900	0.98809000
H	-4.85749100	-2.11540600	-1.25547700
H	-3.23115000	-1.96447000	-1.99696000
H	-4.36343700	-0.59792200	-2.03781000
H	-3.75752100	-0.40752400	1.92978600
H	-5.17676500	-1.17061600	1.14108900
H	-4.74497000	0.51500700	0.78025100

$SmI_2(thf)_4$-HMAC

H_{corr} = 0.7766
$-TS$ = -0.1243
$E_{sol(THF)}$ = -25994.4838
$G_{sol(THF)}$ = -25993.8315

H	4.77585500	-3.50340100	1.35069700
H	0.27151600	-4.78525100	1.80829900
H	-1.47711300	-5.12177700	1.86526500
H	5.42195900	-2.58967200	-1.13911700

H	4.00166400	-3.65354700	-0.98950200
H	-0.14995400	-5.68777200	-0.45273700
H	-2.02733900	-2.81139200	1.32962900
H	-0.57112700	-2.55334200	2.33961700
H	-1.68080500	-4.78574000	-0.56706400
H	1.16858200	-3.66000200	-0.62833800
H	-0.21651400	-3.17675700	-1.64191600
O	-2.12213500	-0.02034000	0.06135600
Sm	0.39000400	0.00447400	0.01267800
O	-0.25226600	-2.48681400	0.30765000
C	-0.95166900	-3.04177800	1.43079000
C	-0.70100500	-4.53638500	1.35300800
C	-0.65875900	-4.75999200	-0.15651500
C	0.07446400	-3.51864900	-0.63816300
I	0.62006400	0.25695200	3.20989500
I	0.34116700	-0.28386800	-3.19002400
O	2.49328500	1.53755000	-0.18140600
C	3.53762500	1.64070100	0.79881400
C	4.39504200	2.81052300	0.34863900
C	4.26518100	2.72174400	-1.16965000
C	2.80397400	2.34104700	-1.33051600
O	-0.34282400	2.47772700	-0.23428100
C	0.00180700	3.49533100	0.72116100
C	-0.77744600	4.73148800	0.30014600
C	-0.89655000	4.53436400	-1.20904700
C	-1.12161300	3.03662100	-1.30033000
O	2.55122700	-1.46475000	0.02551400
C	2.99928500	-2.24075800	1.14833700
C	4.44762300	-2.58381400	0.84668200
C	4.43027300	-2.68749000	-0.67604800
C	3.49841600	-1.54830000	-1.05030000
H	4.11166600	0.69718400	0.80091700
H	3.06911300	1.75894800	1.78630200
H	2.15265900	3.23333800	-1.32213100
H	2.57301200	1.75415000	-2.23141000
H	3.97218000	3.76060700	0.71431300
H	5.42994900	2.73774500	0.71070200
H	4.51704400	3.65497400	-1.69218400
H	4.91590000	1.92421200	-1.56427700
H	1.09148300	3.65957600	0.66791400
H	-0.23888700	3.12470300	1.72802200
H	-2.18505100	2.78262300	-1.13947000
H	-0.78940100	2.57136100	-2.23967200
H	-1.77704200	4.73361600	0.76404400
H	-0.27003700	5.66314100	0.58638300
H	-1.71053800	5.11283800	-1.66756900
H	0.04489000	4.81116300	-1.71115700
H	2.37593300	-3.15022000	1.21792600
H	2.84250400	-1.64654500	2.06020300
H	4.04102600	-0.58845200	-1.11629900
H	2.94094600	-1.69131400	-1.98694800
H	5.11171300	-1.76448500	1.16755400
C	-3.34679500	-0.02042800	0.05427200
C	-4.02076900	-0.69577800	-1.15597100
C	-4.05181700	0.64079100	1.24913800
C	-5.49954300	-1.06418400	-1.02098300
C	-3.23301500	-1.97123600	-1.48327500
C	-3.84619400	0.30177000	-2.31708000
C	-4.29386100	-0.48988800	2.26689400
C	-5.36969800	1.34954000	0.91842600
C	-3.09814000	1.66156200	1.87752200
H	-5.71224900	-1.64154800	-0.10914600
H	-5.77378300	-1.69921100	-1.87728500
H	-6.16233000	-0.19116500	-1.04690800
H	-3.34080700	-2.71690900	-0.67937600
H	-2.16845900	-1.75473400	-1.63345100
H	-3.62828900	-2.41245400	-2.41051500
H	-4.33984700	1.26381800	-2.10519800
H	-4.30509000	-0.11709800	-3.22612600
H	-2.78001500	0.47740200	-2.52289800
H	-4.78631300	-0.07338700	3.15945700
H	-3.33845600	-0.93389700	2.58371100
H	-4.94089500	-1.28432900	1.86600400
H	-5.64863100	1.98162200	1.77514000
H	-6.20151500	0.65837200	0.74349900
H	-5.27365300	2.01072900	0.04322800
H	-3.57457000	2.09879200	2.76797200
H	-2.87859300	2.47727000	1.17209100
H	-2.14696300	1.20757300	2.18623100

$SmI_2(thf)_4^+$-HMAC$^-$

H_{corr} = 0.7758
$-TS$ = -0.1187
$E_{sol(THF)}$ = -25994.4593
$G_{sol(THF)}$ = -25993.8022

H	4.40242700	-3.33323700	1.95802500
H	-2.01575800	-4.83987300	1.80852800
H	-2.69076200	-4.26732000	0.26589000
H	5.38813000	-1.62351600	-0.05060700
H	4.85906900	-3.27598500	-0.39757100
H	0.22686900	-5.03442600	0.82379500
H	-2.26873500	-2.10934500	1.18747500
H	-0.95306400	-2.71351700	2.24265700
H	-0.80026500	-5.59580700	-0.52257700
H	0.86420100	-3.45259600	-0.93968900
H	-0.81190100	-3.36311100	-1.54199100
O	-1.89467100	0.07774100	0.03224400
Sm	0.20534000	-0.04024100	-0.02643500
O	-0.43520300	-2.41837100	0.26986100
C	-1.42468700	-2.80967000	1.25234100
C	-1.79834000	-4.24578300	0.91053600
C	-0.58165900	-4.74241700	0.13398700
C	-0.19090100	-3.50150500	-0.64227300
I	0.64059400	0.30014700	3.01288700
I	0.54701900	-0.45580200	-3.05854400
O	2.50404100	1.25529100	-0.25090800
C	3.53292300	1.34860800	0.75295000
C	4.39578600	2.52358300	0.33612600
C	4.32382600	2.42865400	-1.18400500
C	2.87222600	2.04226900	-1.40255500
O	-0.04505300	2.41279600	-0.34260600
C	0.33620400	3.44613600	0.58880000
C	-0.49707800	4.65681100	0.20818200
C	-0.67512900	4.46341800	-1.29521600
C	-0.86900900	2.96282400	-1.39393700
O	2.25682800	-1.62199400	0.15698500
C	2.49973200	-2.53952100	1.22714600
C	3.99794700	-2.45476400	1.43671600
C	4.52887100	-2.30698700	0.00378500
C	3.32241300	-1.78132800	-0.79110500
H	4.11185800	0.40990000	0.75168900
H	3.04359100	1.45176700	1.73053800
H	2.21641700	2.92795100	-1.44007400
H	2.68488200	1.43858300	-2.30109900

H	3.95358200	3.47090900	0.68552300
H	5.41620500	2.45657900	0.73812100
H	4.58959900	3.36055900	-1.70178800
H	4.99440800	1.63372400	-1.54927700
H	1.41586600	3.63568400	0.46630800
H	0.16050600	3.07343000	1.60753400
H	-1.91003200	2.66670000	-1.20128100
H	-0.53904500	2.51570200	-2.34216000
H	-1.47283800	4.62222800	0.71599800
H	-0.00214200	5.60053500	0.47558500
H	-1.52694500	5.01869000	-1.71098600
H	0.23313100	4.77418700	-1.83698100
H	2.19140100	-3.55625900	0.91949400
H	1.89948000	-2.21108200	2.08483500
H	3.47681900	-0.80419600	-1.26298600
H	3.00068900	-2.48482300	-1.57658000
H	4.22806500	-1.56482800	2.04164500
C	-3.23601200	0.02379300	0.11982900
C	-3.93630400	-0.40991600	-1.16960500
C	-3.79458600	0.82461200	1.30310700
C	-5.37782200	-0.90073300	-0.98430400
C	-3.16330800	-1.58630600	-1.78125300
C	-3.92347300	0.74744800	-2.18515000
C	-3.42152600	0.08554200	2.59932300
C	-5.30792700	1.04355300	1.31384800
C	-3.13954100	2.21593500	1.33900000
H	-5.44909800	-1.62936300	-0.16146500
H	-5.70280000	-1.40683900	-1.90730600
H	-6.09231500	-0.09220500	-0.79275900
H	-3.20039500	-2.46042400	-1.11197500
H	-2.11434900	-1.33321900	-1.97752900
H	-3.62325800	-1.87628400	-2.73940100
H	-4.45003200	1.63153700	-1.79077200
H	-4.41658300	0.45211700	-3.12676300
H	-2.88827000	1.03037400	-2.42598700
H	-3.72980200	0.66859900	3.48340000
H	-2.33645900	-0.07418700	2.66725000
H	-3.92670700	-0.89275100	2.64430800
H	-5.56667400	1.65335000	2.19436800
H	-5.86902100	0.10320500	1.39277700
H	-5.65872500	1.58826400	0.42449000
H	-3.45580400	2.76551800	2.24059300
H	-3.43716000	2.81128900	0.46019600
H	-2.04612500	2.12919900	1.35507600

$SmI_2(thf)_4$-CPO

H_{corr} = 0.6376
$-TS$ = -0.1112
$E_{sol(THF)}$ = -25836.1041
$G_{sol(THF)}$ = -25835.5778

H	-4.03592800	-4.11904800	-0.22889500
H	0.15238300	-3.74658300	-2.01405900
H	1.73389600	-4.52171600	-2.21820200
H	-3.75889900	-3.46134700	2.40686000
H	-2.48831100	-4.45997600	1.65915700
H	0.19313100	-4.12943100	0.28426500
H	2.90120600	-2.46950500	-1.98900200
H	1.33420500	-1.73428700	-2.49585300
H	1.74430100	-4.98536900	0.13352300
H	1.54095000	-2.49487700	1.44302300
H	3.00653700	-2.99381500	0.54092900
O	2.12567000	1.15278600	-0.63886800
Sm	-0.14635300	0.11890700	0.01175000
O	1.67087300	-1.78455700	-0.47362500
C	1.82158400	-2.38936400	-1.76026300
C	1.18289000	-3.77191900	-1.63359700
C	1.21282900	-4.05688200	-0.11710900
C	1.91372200	-2.83300900	0.46639500
I	-1.11338500	0.00197900	-3.05451300
I	0.82007400	0.04061900	3.06577600
O	-2.52650500	0.76255600	0.84343600
C	-3.67983700	0.73181400	-0.00929600
C	-4.37565900	2.05527200	0.23493100
C	-4.15215300	2.23410600	1.73418400
C	-2.73587900	1.70452800	1.91699200
O	-0.33193100	2.73107600	0.05631300
C	-0.70292600	3.47139200	-1.10799000
C	0.61995700	3.99315500	-1.64252200
C	1.42977300	4.26422200	-0.36187300
C	0.65071900	3.52189500	0.73427100
O	-1.49538200	-2.11157700	0.41998500
C	-2.30335000	-2.82181400	-0.53643300
C	-3.55248300	-3.23865700	0.21668200
C	-2.99649600	-3.48281000	1.61592200
C	-1.99323300	-2.35115700	1.75300400
H	-4.32628800	-0.11581800	0.28587800
H	-3.33318300	0.57554400	-1.04184200
H	-1.98114400	2.49709600	1.80874300
H	-2.56630600	1.18814700	2.87268600
H	-3.87219000	2.85438400	-0.33263800
H	-5.43516800	2.03907800	-0.05561700
H	-4.25207500	3.27408400	2.07442800
H	-4.87400300	1.62292100	2.29942400
H	-1.38189600	4.29474000	-0.81190700
H	-1.22422300	2.78599500	-1.79103300
H	1.25142700	2.83744100	1.34758500
H	0.12884000	4.22681000	1.40620200
H	1.09561600	3.20405000	-2.24234900
H	0.49644400	4.88361900	-2.27436200
H	2.45571200	3.88376700	-0.45273900
H	1.48972100	5.33846500	-0.13455000
H	-1.74134200	-3.70763400	-0.88248200
H	-2.47344500	-2.16126400	-1.39865100
H	-2.46744800	-1.42294700	2.11071600
H	-1.13874600	-2.57071500	2.40755400
H	-4.28595400	-2.41668200	0.23197700
C	3.26888700	0.74344900	-0.52000300
C	4.00102100	0.54283000	0.78080700
C	4.16534400	0.33507600	-1.66275600
C	5.41957700	0.14241700	0.37942400
H	3.46319100	-0.25860700	1.31988300
H	3.90940600	1.42603800	1.42869100
H	3.59436900	-0.12979200	-2.47690800
C	5.24225700	-0.51706400	-0.99572600
H	4.60227300	1.26916800	-2.06355100
H	6.17516800	-0.57334900	-1.57328500
H	4.86843600	-1.54732900	-0.86867300
H	5.90395300	-0.51776100	1.11173900
H	6.05372500	1.03940000	0.27967700

$SmI_2(thf)_4^+$-CPO^-

H_{corr} = 0.6362
$-TS$ = -0.1083
$E_{sol(THF)}$ = -25836.0797

$G_{sol(THF)}$ = -25835.5518

H	-3.63428000	-3.34696500	-2.84423400
H	1.75984200	-4.24675900	-2.46026100
H	3.35100800	-3.75884100	-1.82817000
H	-4.78184800	-3.22134900	-0.37188300
H	-3.16212200	-3.95401700	-0.49460700
H	1.70221600	-5.18097000	-0.18430300
H	2.58851700	-1.52126800	-1.32852800
H	1.52313400	-1.85045300	-2.72478600
H	2.48118800	-3.71870600	0.48170200
H	-0.33435100	-3.93378900	-0.71787400
H	0.14178300	-3.16892800	0.83806800
O	1.98391500	0.23355200	0.29038600
Sm	-0.09472200	0.06755700	0.07449400
O	0.67020700	-2.12003500	-0.85809500
C	1.82472700	-2.20238600	-1.72859900
C	2.26715300	-3.65593100	-1.68067900
C	1.79656600	-4.09210800	-0.29638500
C	0.46440500	-3.37963200	-0.19116100
I	0.00839600	1.21828000	-2.78525400
I	-0.56654000	-1.12717300	2.88513000
O	-2.33411600	1.29459900	0.29229600
C	-3.25237200	1.58676100	-0.78023100
C	-4.27514600	2.53864800	-0.18683400
C	-4.31790900	2.08481000	1.26876500
C	-2.85433500	1.79989100	1.54092700
O	0.12894200	2.29582200	1.19497400
C	0.16219500	3.50080200	0.42024200
C	1.64410700	3.79132700	0.25870400
C	2.25309000	3.27936800	1.57725700
C	1.12586700	2.46343300	2.21892200
O	-2.05183300	-1.39942400	-0.70849900
C	-2.20139600	-1.89828900	-2.05567600
C	-3.57461400	-2.54486400	-2.09579500
C	-3.73881600	-3.02936800	-0.65881200
C	-3.12620700	-1.88260600	0.12046500
H	-3.71697200	0.64016900	-1.10415600
H	-2.67656600	1.99357200	-1.62269900
H	-2.30069800	2.71549600	1.80369000
H	-2.66423800	1.04165900	2.31308900
H	-3.91570500	3.57814800	-0.25209900
H	-5.24511900	2.48065200	-0.69964400
H	-4.73129100	2.83658900	1.95510200
H	-4.91621500	1.16442400	1.36909200
H	-0.35954100	4.29649100	0.98505300
H	-0.36494700	3.30959800	-0.52377900
H	1.42116500	1.46193100	2.55238100
H	0.65284400	2.99724200	3.06107600
H	2.02854100	3.21685800	-0.59656900
H	1.83987800	4.85674700	0.07455700
H	3.12952400	2.64528500	1.38621200
H	2.56006900	4.10294200	2.23767400
H	-1.39498800	-2.62582800	-2.23999300
H	-2.06912000	-1.05527100	-2.74779300
H	-3.85140400	-1.06343000	0.27094900
H	-2.70230500	-2.15486700	1.09636400
H	-4.34755800	-1.79718200	-2.33736100
C	3.30770300	0.26777500	0.32709200
C	4.11233500	-0.64045000	1.20421000
C	4.13437700	0.84004700	-0.79002300
C	5.55036000	-0.26005200	0.84441600
H	3.91722000	-1.71132500	0.96375200
H	3.87727500	-0.53010900	2.27681100
H	3.65334300	0.69506900	-1.77434100
C	5.47016300	0.09886400	-0.64499100
H	4.27552700	1.93663200	-0.68866900
H	6.33739600	0.67812400	-0.99660400
H	5.43858800	-0.83117600	-1.23962200
H	6.28125600	-1.05483300	1.05675100
H	5.85293700	0.62885500	1.42424000

$SmI_2(MeTHF)_4$-ACE

H_{corr} = 0.7165

$-TS$ = -0.1188

$E_{sol(MeTHF)}$ = -25915.9264

$G_{sol(MeTHF)}$ = -25915.3286

H	1.08516400	-5.15395800	1.60941900
H	-4.69146900	-3.17570100	0.22469500
H	-4.93943700	-1.83819000	-0.92135700
H	3.19018000	-4.34989400	0.02330800
H	1.80422200	-5.18246200	-0.71095100
H	-2.69790300	-3.93399300	-0.98432600
H	-3.65986100	-0.67433100	0.77766800
H	-2.71968000	-2.12427300	1.18127700
H	-3.78205600	-3.49687300	-2.32748400
H	-1.28793200	-2.33121900	-2.02322500
C	-2.78620900	-1.09440600	-2.94917400
O	-1.84772500	1.94630500	0.19423500
Sm	0.03286200	0.07979200	0.08519900
O	-2.19909300	-1.18166300	-0.59282200
C	-3.20432600	-1.58127200	0.35429100
C	-4.17458500	-2.45310100	-0.42146000
C	-3.25348000	-3.10410900	-1.44790100
C	-2.30266000	-1.96626300	-1.80964700
I	-0.68019400	-0.55147400	3.16987300
I	0.81140200	0.80680800	-2.94871900
O	2.61627700	-0.27741000	0.47463400
C	3.05059000	-0.64640800	1.78692200
C	3.72805000	0.60498600	2.30472500
C	4.47618800	1.08481600	1.06129800
C	3.62167100	0.58407300	-0.11612200
O	1.10987100	2.32549300	0.83531900
H	-1.98479200	2.36193900	-2.44315100
H	-3.81148000	-0.73150100	-2.77362800
H	-2.66196700	3.81390000	-1.64213700
H	-2.77880600	-1.66978100	-3.88767800
O	0.54660000	-2.49292600	-0.25954000
C	0.05289300	-3.47087700	0.65948200
C	1.29852200	-4.18087600	1.14546300
C	2.10600300	-4.28567700	-0.14507100
C	1.72436500	-3.02122500	-0.92733700
H	3.75581800	-1.49733900	1.72063200
H	2.16398200	-0.94891800	2.36322300
H	3.06225900	1.40046300	-0.59502100
C	4.39574400	-0.16868800	-1.17524400
H	2.95205700	1.32518700	2.60363300
H	4.38586700	0.41454900	3.16411000
H	4.60958700	2.17562400	1.03472800
H	5.47902700	0.63019200	1.01561200
H	5.17266700	0.48241700	-1.60564900
H	-3.73823800	2.69247900	-2.51164600
H	3.72558100	-0.47951700	-1.98962600
H	4.89164800	-1.05455100	-0.74643700
H	1.14799100	-2.31436500	-2.88762400

H	0.62662500	-3.99381500	-2.51936100
H	2.33370700	-3.64482600	-2.89408700
H	-2.11464200	-0.23387800	-3.08320900
H	-0.62740000	-4.16834100	0.13428100
H	-0.49699300	-2.94164100	1.45081400
H	2.49036500	-2.24193600	-0.81451900
C	1.43414000	-3.25440900	-2.39241400
H	1.81003500	-3.54909300	1.88809500
C	-2.09834900	2.61304000	1.43719300
C	-3.05759600	1.95052800	-0.61002100
C	-4.16052800	2.48206500	0.31278800
C	-3.56336700	2.35122900	1.71095700
H	-3.23431600	0.89753100	-0.87252600
C	-2.84234900	2.75166600	-1.87365900
H	-5.10233300	1.92970700	0.18538600
H	-4.36339700	3.54112400	0.08260600
H	-3.67557200	1.32984300	2.10796200
H	-3.99560600	3.05204100	2.43861300
H	-1.89409100	3.69554200	1.32457700
H	-1.42219100	2.18511800	2.19017400
C	1.29405400	3.48363800	0.47973700
C	2.22049200	4.37494500	1.24996300
C	0.62544400	4.03448900	-0.73704200
H	3.01187100	4.74819400	0.57965100
H	2.66140000	3.84189800	2.10027500
H	1.66989000	5.26095800	1.60516300
H	0.79016500	5.11067700	-0.87247900
H	-0.44677000	3.79931900	-0.68984400
H	0.99970100	3.47450800	-1.61435700

$SmI_2(MeTHF)_4^+$-ACE$^-$

H_{corr} = 0.7157
$-TS$ = -0.1155
$E_{sol(MeTHF)}$ = -25915.9011
$G_{sol(MeTHF)}$ = -25915.3009

H	2.51450800	-4.02788600	2.58228100
H	-1.94832400	-3.17058000	2.22597500
H	-3.67958200	-3.46328200	1.94396000
H	4.17192400	-2.57402000	0.79120100
H	3.53243900	-4.18341200	0.42233400
H	-1.68410200	-4.74279300	0.43485700
H	-3.81569400	-1.52137600	0.44559000
H	-2.66619300	-0.93336000	1.70107400
H	-3.27750800	-4.36837200	-0.23915600
H	-0.70533300	-3.07669200	-0.94263600
C	-2.51866500	-2.75767500	-2.05278800
O	-2.28867300	1.16054500	-0.21763700
Sm	0.02117500	0.23073000	0.00091900
O	-1.78876800	-1.62953300	-0.01809900
C	-2.84641600	-1.70660200	0.94424400
C	-2.74761100	-3.12261900	1.47203600
C	-2.38108300	-3.92154200	0.21730300
C	-1.76703400	-2.88608800	-0.74548300
I	-0.33019500	0.21158100	3.09536900
I	0.42344700	-0.21053300	-3.01131700
O	2.53884900	0.45210900	0.14157200
C	3.18217700	0.52365400	1.42108500
C	3.40201200	2.00595700	1.62927700
C	3.79486700	2.47786000	0.22758800
C	3.17228800	1.44265700	-0.72362100
O	0.30905700	2.29744400	-0.02067700
H	-2.08212000	1.49642800	-2.88646300
H	-3.58094400	-2.52053500	-1.87978000
H	-3.05715800	2.84387300	-2.22492300
H	-2.47525700	-3.71352700	-2.59775700
O	1.15106900	-2.10056500	0.30418200
C	0.99168200	-2.94764500	1.44673300
C	2.40377300	-3.12244300	1.96968800
C	3.24513100	-3.15466700	0.68632500
C	2.33053100	-2.56590700	-0.40505900
H	4.13439300	-0.03737500	1.38113800
H	2.51390900	0.06233300	2.16014900
H	2.35293800	1.87660200	-1.31005600
C	4.16038300	0.75500700	-1.63862500
H	2.45311300	2.46315500	1.94399400
H	4.16484200	2.21943600	2.39092500
H	3.41330300	3.48525000	0.01408100
H	4.88982800	2.48959900	0.10815000
H	4.65448300	1.50194300	-2.27889000
H	-3.83906200	1.57267300	-3.19603400
H	3.64561200	0.03632500	-2.29259100
H	4.94157500	0.23263700	-1.06304800
H	1.27701400	-3.09565500	-2.21481700
H	1.46186300	-4.45348300	-1.04906000
H	2.85228800	-3.89812200	-2.00761100
H	-2.06654700	-1.98119500	-2.68593400
H	0.54902700	-3.91072500	1.13287700
H	0.31610400	-2.43857900	2.14564300
H	2.75662800	-1.66589100	-0.86165800
C	1.94705900	-3.56211200	-1.47870500
H	2.66678300	-2.25801300	2.59643400
C	-2.72650300	2.21692900	0.64575000
C	-3.32773700	0.94948500	-1.20325000
C	-4.63548200	1.33899500	-0.48460900
C	-4.18773400	1.88610100	0.87775600
H	-3.28050100	-0.12164000	-1.43861200
C	-3.05374600	1.76561900	-2.44863000
H	-5.31578400	0.48217000	-0.38030300
H	-5.16588800	2.10707700	-1.06693200
H	-4.26790300	1.11524500	1.65874500
H	-4.76964800	2.75904500	1.20399900
H	-2.59062400	3.18781700	0.13906800
H	-2.10061700	2.19213400	1.54640300
C	0.48671100	3.61735700	-0.14039000
C	0.27450200	4.43726100	1.08802000
C	0.21332000	4.19883900	-1.48659100
H	0.77111500	5.41746500	1.01111300
H	0.65385800	3.91325900	1.97820000
H	-0.80365300	4.63923400	1.27593300
H	0.65688100	5.20101300	-1.59483000
H	-0.87860600	4.30545800	-1.67628600
H	0.60458300	3.54495300	-2.28155200

$SmI_2(MeTHF)_4$-HMAC

H_{corr} = 0.8943
$-TS$ = -0.1315
$E_{sol(MeTHF)}$ = -26151.6922
$G_{sol(MeTHF)}$ = -26150.9294

H	-4.30966900	-3.67733500	1.53831700
H	-4.38693800	0.59358700	2.13398500
H	-5.00508400	2.25069700	2.30859300
H	-2.69591700	-4.78238600	-0.47055800
H	-4.26123200	-4.03872400	-0.83208100
H	-5.59977500	0.65498000	0.06883100

H	-2.95867800	3.18299200	1.34687200
H	-2.29909500	1.78304700	2.27365400
H	-5.50739000	2.42005600	-0.02699300
H	-3.59172400	0.47354400	-1.18051400
C	-3.55249600	2.57291800	-1.64281000
O	0.48632700	2.47447400	0.39803500
Sm	-0.32224500	-0.01010900	-0.02283000
O	-2.55669700	1.44431100	0.27312300
C	-2.98978900	2.08340600	1.47387100
C	-4.40977200	1.58778200	1.66526700
C	-4.93423600	1.51528700	0.22746300
C	-3.67035300	1.43382000	-0.65241500
I	-0.57601200	-0.37649700	3.20896000
I	-0.53005200	0.50852300	-3.17976300
O	0.60592600	-2.51441700	-0.24722400
C	0.31251200	-3.44704800	0.80251400
C	1.58599700	-4.24706900	0.98706200
C	2.07342700	-4.36475700	-0.45094200
C	1.69183900	-3.01854300	-1.06600800
O	2.26507900	0.04297900	0.00742100
H	0.34302200	3.29337200	-2.15513200
H	-3.57718400	3.54781800	-1.12873900
H	1.44927200	4.41625800	-1.29584600
H	-4.39968700	2.54270300	-2.34576300
O	-2.39158600	-1.64344900	-0.18830100
C	-3.34211400	-1.83569800	0.85941000
C	-3.39048200	-3.33950500	1.03966600
C	-3.26697500	-3.84555300	-0.40043500
C	-2.58256400	-2.69417700	-1.16550000
H	-0.51806300	-4.10219200	0.48361500
H	0.00157100	-2.87407800	1.68872400
H	2.52006400	-2.30084600	-0.94788800
C	1.27487900	-3.07867700	-2.51586300
H	2.30385600	-3.68436400	1.60356300
H	1.40793300	-5.21675300	1.47224000
H	3.15080200	-4.56463700	-0.53890200
H	1.53814000	-5.17707200	-0.97034700
H	2.12435700	-3.40806200	-3.13489300
H	-0.16844700	4.96569300	-1.80241400
H	0.94095300	-2.09524500	-2.87975500
H	0.45263300	-3.79892800	-2.65361900
H	-2.87462300	-1.34786600	-2.83221800
H	-4.39192700	-1.86345700	-2.01870800
H	-3.51854200	-2.99367100	-3.08068800
H	-2.62391500	2.48386400	-2.22454800
H	-4.32561900	-1.43378200	0.55100200
H	-2.97760500	-1.29036000	1.74035300
H	-1.56842300	-2.96215600	-1.49480900
C	-3.38896700	-2.18721500	-2.34183200
H	-2.53522800	-3.65806200	1.65346800
C	1.18109200	2.79102400	1.60707400
C	-0.18972600	3.66477900	-0.09495600
C	-0.07157200	4.69830800	1.03256200
C	0.34500000	3.87755000	2.24923500
H	-1.23384300	3.35784600	-0.24479500
C	0.39948700	4.10515700	-1.41457400
H	-1.01035400	5.25032800	1.18108700
H	0.71025800	5.43439000	0.78299900
H	-0.52509400	3.42010600	2.74412000
H	0.90118000	4.45790900	2.99862300
H	2.19951300	3.15015900	1.36581100
H	1.24475600	1.87357000	2.20881100
C	3.48252000	0.17856100	0.01070200
C	4.25018900	-0.21934400	1.28078200
C	4.11212400	0.80058200	-1.25245700
C	5.26421800	-1.33286600	0.97095500
C	3.25607900	-0.75486500	2.30895800
C	4.95275700	1.00683900	1.88281400
C	5.62295300	0.65657200	-1.44077300
C	3.74768300	2.29492900	-1.17091600
C	3.42636000	0.18967900	-2.48022100
H	5.63766200	-1.74114300	1.92253800
H	6.12950200	-0.98752500	0.39490900
H	4.78984600	-2.16261200	0.42230500
H	2.70887100	-1.62530300	1.92409500
H	2.49798200	-0.01379900	2.59160900
H	3.79595800	-1.05772500	3.21861900
H	5.46035100	0.70506500	2.81212400
H	4.22198600	1.78727600	2.14313300
H	5.70762700	1.44785700	1.21939900
H	5.91709800	-0.38727000	-1.61899300
H	6.21661100	1.05041400	-0.60578800
H	5.90903700	1.22838700	-2.33706700
H	4.26396600	2.79714400	-0.33805400
H	2.66407600	2.41771300	-1.04422300
H	4.04882300	2.79265600	-2.10557800
H	3.81470800	0.67251800	-3.38974200
H	2.33741400	0.32819000	-2.46461100
H	3.64193700	-0.88788500	-2.55832100

$SmI_2(MeTHF)_4^+$-$HMAC^-$

H_{corr} = 0.8935
$-TS$ = -0.1269
$E_{sol(MeTHF)}$ = -26151.6623
$G_{sol(MeTHF)}$ = -26150.8958

H	-4.28275100	-3.73497600	1.17937300
H	-4.22914800	0.44753900	2.19350000
H	-4.92230000	2.07927900	2.31714500
H	-2.34910100	-4.74399900	-0.64999600
H	-3.94738500	-4.17184800	-1.14011100
H	-5.46543600	0.34900900	0.14961300
H	-2.95376800	3.06557900	1.26067800
H	-2.20312000	1.74834800	2.23272000
H	-5.52092800	2.10990900	0.00270200
H	-3.50856900	0.32528800	-1.20527700
C	-3.60260400	2.43417800	-1.60862000
O	0.20024700	2.44008000	0.44283700
Sm	-0.12103200	-0.00000200	0.00055900
O	-2.48776100	1.30305000	0.25294300
C	-2.92447400	1.97443100	1.43857300
C	-4.31216900	1.41894800	1.68535500
C	-4.86777200	1.26406400	0.26506800
C	-3.62414300	1.26268100	-0.64826000
I	-0.60832700	-0.43607500	3.05634400
I	-0.52631200	0.51698100	-2.99323000
O	0.61339300	-2.41478400	-0.13550900
C	0.22615300	-3.45410900	0.77359200
C	1.52880200	-4.10679200	1.17991000
C	2.30792300	-4.09321500	-0.13189200
C	1.80086900	-2.84845800	-0.87100700
O	1.98386300	0.15270800	-0.00861300
H	0.24231100	3.39350800	-2.09480200
H	-3.68794400	3.39340500	-1.07273400
H	1.31595600	4.42486100	-1.10168100

H	-4.46213300	2.36008500	-2.29290300
O	-2.20306900	-1.66502900	-0.28937300
C	-3.21747200	-1.89346700	0.69351400
C	-3.30408200	-3.40395800	0.80518700
C	-3.02074800	-3.87453800	-0.62745800
C	-2.40367800	-2.65031700	-1.33511800
H	-0.43036600	-4.17053500	0.24975100
H	-0.31918100	-2.98555000	1.60262300
H	2.51321400	-2.01586500	-0.78199100
C	1.44728700	-3.09590900	-2.31890300
H	2.02583700	-3.49838500	1.94914400
H	1.38137800	-5.11737600	1.58578000
H	3.39590900	-4.05630000	0.01748500
H	2.08232400	-4.99722400	-0.72043300
H	2.35223900	-3.38422300	-2.87588100
H	-0.25125600	5.05476200	-1.66667800
H	1.03057900	-2.19408000	-2.79040100
H	0.71765900	-3.91711400	-2.41082100
H	-2.85376500	-1.20948600	-2.88611600
H	-4.30055600	-1.87236800	-2.04954900
H	-3.40999700	-2.86994100	-3.21968300
H	-2.68490200	2.42812300	-2.21226300
H	-4.17221000	-1.46245700	0.34465000
H	-2.90226300	-1.39156900	1.61646500
H	-1.39959700	-2.85207200	-1.73106000
C	-3.29476000	-2.10786000	-2.43345100
H	-2.53906800	-3.76866000	1.50464600
C	1.07852000	2.80155900	1.51740300
C	-0.42146900	3.65820100	-0.05139500
C	-0.35196200	4.64944200	1.12604900
C	0.29940500	3.86153800	2.26507600
H	-1.45019000	3.36749200	-0.28885700
C	0.26825700	4.15443500	-1.30224200
H	-1.34504500	5.03494200	1.39633000
H	0.26975500	5.51357000	0.84576500
H	-0.45605700	3.37471800	2.89996800
H	0.93906500	4.48121600	2.90845500
H	2.02401000	3.18684500	1.10007300
H	1.28315900	1.89662900	2.10189100
C	3.32936600	0.12956300	-0.08106300
C	4.08823300	-0.06720000	1.23482300
C	3.89061600	0.76174900	-1.35886200
C	5.25926900	-1.05323000	1.07943800
C	3.14850300	-0.66519500	2.28085300
C	4.62037800	1.26108600	1.80804200
C	5.41596600	0.76085500	-1.49064300
C	3.39647400	2.21586500	-1.44349000
C	3.35317400	-0.00653700	-2.57450200
H	5.68522200	-1.28240400	2.07017200
H	6.07235100	-0.66392800	0.45646300
H	4.91265700	-1.99925900	0.63362600
H	2.67860500	-1.58758800	1.91439900
H	2.33457800	0.01621600	2.55431000
H	3.71076000	-0.90345200	3.19734100
H	5.11896800	1.08604800	2.77653900
H	3.79641100	1.96845000	1.98146700
H	5.34864600	1.74722600	1.14445600
H	5.82012700	-0.25962600	-1.55369200
H	5.92828100	1.28851700	-0.67482300
H	5.68421500	1.27445400	-2.42777900
H	3.82672100	2.82770100	-0.63408600
H	2.30280200	2.24638100	-1.36306500
H	3.68404700	2.67332100	-2.40458000
H	3.66611300	0.48681500	-3.50919400
H	2.25736700	-0.04823600	-2.57536500
H	3.74901100	-1.03464000	-2.58757700

$SmI_2(MeTHF)_4$-CPO

H_{corr} = 0.7545
$-TS$ = -0.1194
$E_{sol(MeTHF)}$ = -25993.3150
$G_{sol(MeTHF)}$ = -25992.6798

H	-4.87582600	-2.67872200	1.35027400
H	-4.28277100	1.74894800	0.77510100
H	-4.51214300	3.50217200	0.55981300
H	-3.39923100	-4.36253700	-0.25586900
H	-4.58697900	-3.26486000	-0.98887500
H	-5.05673200	1.80476100	-1.54724700
H	-2.14783800	3.79054900	-0.04008700
H	-2.04831200	2.56017900	1.27566700
H	-4.23254900	3.34887600	-1.84135400
H	-3.04409700	0.57394500	-1.83265900
C	-2.24923600	2.18830900	-3.01153700
O	1.06179000	2.35198900	0.20734500
Sm	-0.15989800	0.00016600	0.07843700
O	-1.95263700	1.80279100	-0.62871600
C	-2.46461600	2.78015100	0.28194800
C	-3.96748900	2.62360200	0.18687300
C	-4.15492600	2.38639200	-1.30978500
C	-2.87424300	1.65677300	-1.74151200
I	-1.11160100	0.43583300	3.13948500
I	0.94353800	-0.46527900	-2.90878400
O	0.28563000	-2.58657500	0.41102400
C	0.01739100	-3.12154800	1.71086900
C	1.39091900	-3.44572000	2.26019000
C	2.08124600	-4.02046800	1.02473100
C	1.42074300	-3.28849500	-0.15530200
O	2.22174600	-0.39828500	1.02478600
H	1.98331200	2.38361100	-2.28510000
H	-2.03308900	3.26524300	-2.91991900
H	2.95292300	3.59794600	-1.39036700
H	-2.94397600	2.05252700	-3.85537900
O	-2.43257200	-1.26945900	-0.35412600
C	-3.55308400	-1.13962700	0.52562100
C	-3.86577900	-2.56362200	0.93289700
C	-3.66461400	-3.30401300	-0.38623900
C	-2.54800200	-2.51885900	-1.08851900
H	-0.61015500	-4.02900200	1.61756300
H	-0.52884500	-2.35826200	2.28462300
H	2.07251400	-2.50569100	-0.57097800
C	0.96688600	-4.19674100	-1.27607400
H	1.87152200	-2.51102300	2.58435500
H	1.36099200	-4.14392100	3.10821600
H	3.17196700	-3.88229800	1.03446200
H	1.89006800	-5.10317900	0.94654000
H	1.82977600	-4.74280000	-1.68854200
H	1.74642600	4.12131400	-2.59367500
H	0.51950900	-3.60753800	-2.08929300
H	0.23679900	-4.93695300	-0.91050900
H	-1.97805800	-1.65687800	-2.98842700
H	-3.74954300	-1.70038500	-2.69323700
H	-2.87018900	-3.19459100	-3.10492000
H	-1.31705000	1.65193300	-3.24458200
H	-4.40242500	-0.68173800	-0.01766900

H	-3.25107400	-0.48838100	1.35830500
H	-1.57630100	-3.01494500	-0.96048200
C	-2.80075300	-2.24368600	-2.55330100
H	-3.13901200	-2.89233700	1.69189800
C	1.50783200	2.87701900	1.47174900
C	0.87914300	3.42436300	-0.75129900
C	0.89034100	4.69378600	0.09557500
C	1.85877400	4.33212600	1.21645200
H	-0.09947000	3.24658900	-1.21905100
C	1.95927600	3.37607300	-1.81149300
H	-0.11112400	4.88421600	0.51171200
H	1.19523000	5.57689800	-0.48300100
H	1.73725600	4.95110900	2.11583400
H	2.90133800	4.43324400	0.87542200
H	2.35822100	2.27209200	1.81918800
H	0.69319600	2.76157700	2.20525400
C	3.39605600	-0.22701600	0.74258100
C	3.92156800	0.53159900	-0.44487300
C	4.55792200	-0.75963800	1.55038000
C	5.41588800	0.22040700	-0.47211200
H	3.72642000	1.59942100	-0.24066200
H	3.34480900	0.28411900	-1.35162700
C	5.77237900	-0.01252500	1.00274300
H	4.62296500	-1.83991400	1.32218900
H	4.37084400	-0.67056200	2.62936600
H	6.01567000	1.01352900	-0.93894000
H	5.59150100	-0.70273700	-1.04952500
H	6.71656400	-0.55737200	1.14014200
H	5.87457800	0.95659800	1.51995500

$SmI_2(MeTHF)_4^+$-CPO^-

H_{corr} = 0.7537
$-TS$ = -0.1171
$E_{sol(MeTHF)}$ = -25993.2867
$G_{sol(MeTHF)}$ = -25992.6501

H	-4.25765600	-2.81290400	2.26398200
H	-3.55609500	1.74875000	2.14224900
H	-3.92553000	3.47117800	1.89695000
H	-2.59179000	-4.30508300	0.51017500
H	-4.21927700	-3.76490700	0.06853100
H	-5.04946000	1.45589400	0.28984800
H	-1.94683900	3.74063700	0.46296700
H	-1.33912100	2.59767200	1.71265500
H	-4.74031200	3.08689000	-0.32490500
H	-3.29693700	0.61301300	-1.06890900
C	-3.01517700	2.48301400	-2.09385200
O	0.79988100	2.37151400	0.00028100
Sm	-0.01961400	0.00594200	0.02999300
O	-1.93728900	1.72366600	-0.04647700
C	-2.09753200	2.75360600	0.93610400
C	-3.52337700	2.57082300	1.41216700
C	-4.26068700	2.20199500	0.12050400
C	-3.16242300	1.67334100	-0.82370600
I	-0.22669200	0.19917300	3.12219300
I	-0.28233000	-0.28074200	-3.01679100
O	0.35219100	-2.50365000	0.07791800
C	0.40959800	-3.20027000	1.32999200
C	1.89263600	-3.30760000	1.61086700
C	2.46886200	-3.58976200	0.22093000
C	1.42810900	-3.02202100	-0.75938900
O	2.06996300	-0.15898400	0.07974100
H	1.75791400	1.96434500	-2.48726600
H	-2.81999200	3.54388700	-1.86854100
H	2.85254700	3.12602800	-1.68153100
H	-3.94852900	2.43089900	-2.67574500
O	-2.29596400	-1.24846000	0.15092800
C	-3.21660000	-1.18375300	1.24519600
C	-3.32777100	-2.62215100	1.71058500
C	-3.22980100	-3.41730100	0.40104700
C	-2.64644700	-2.42628000	-0.62445200
H	-0.06740000	-4.19156600	1.21731200
H	-0.14327700	-2.60737400	2.07045600
H	1.81447500	-2.14914100	-1.30017100
C	0.86737900	-4.03083400	-1.73618800
H	2.24909600	-2.34098100	1.99551800
H	2.13074700	-4.08775900	2.34722800
H	3.45052800	-3.11747300	0.07852300
H	2.58982500	-4.67195300	0.05655200
H	1.68011200	-4.43629600	-2.35835900
H	1.75749800	3.67693100	-2.97558200
H	0.13586700	-3.55373700	-2.40438200
H	0.38929300	-4.87397600	-1.21190000
H	-3.14788500	-1.33922900	-2.42243500
H	-4.54938900	-1.65073200	-1.33837700
H	-3.85901900	-2.96740700	-2.31317000
H	-2.20073300	2.08339100	-2.71457200
H	-4.18638200	-0.79199700	0.88764300
H	-2.79408200	-0.50324300	1.99495700
H	-1.69826600	-2.77706800	-1.04658600
C	-3.60836600	-2.06396900	-1.73604000
H	-2.48508600	-2.85093700	2.37913700
C	1.80517600	2.82625500	0.92878400
C	0.76020300	3.29552900	-1.10718300
C	0.91928900	4.64087100	-0.40998300
C	1.93401400	4.33802400	0.70041700
H	-0.23031200	3.16006500	-1.56329000
C	1.84997800	2.99481600	-2.11687900
H	-0.04359300	4.95301900	0.02053300
H	1.25519600	5.43023200	-1.09663900
H	1.72196900	4.90989800	1.61396000
H	2.95537000	4.59481900	0.38470200
H	2.74305000	2.28534600	0.72875600
H	1.45021500	2.55065100	1.93091300
C	3.38800900	-0.03249600	0.05587800
C	4.19209300	-0.10907000	-1.20827700
C	4.24749200	-0.16806400	1.27354300
C	5.60882100	-0.40994500	-0.70007600
H	4.15840800	0.84008300	-1.78134600
H	3.81070900	-0.88113600	-1.90304800
C	5.63855300	0.15764900	0.72514800
H	4.22613400	-1.20324800	1.68411000
H	3.92744600	0.48699800	2.10237300
H	6.40296100	-0.00160400	-1.34327300
H	5.75820300	-1.50285000	-0.65558000
H	6.45858500	-0.25298200	1.33334100
H	5.77258200	1.25256900	0.68646400

$SmI_2(thp)_4$-ACE

H_{corr} = 0.7207
$-TS$ = -0.1165
$E_{sol(THP)}$ = -25915.8882
$G_{sol(THP)}$ = -25915.2840

I	0.42328400	0.38176300	3.14798100
H	0.26262400	4.34630200	0.44219000

H	0.27700800	2.97634300	1.59456200
H	-1.51961300	5.16244400	-1.10517500
H	-2.68375800	4.20530000	-2.02278600
H	-1.63768300	4.85697800	1.74916500
O	-0.39583300	2.52126100	-0.26500900
C	-0.92286600	3.09989800	-1.46600900
C	-1.98145600	4.16303900	-1.17620200
C	-1.69411800	3.85510000	1.29253900
C	-0.30313400	3.46681500	0.80099300
C	-2.69406600	3.83815500	0.13021400
Sm	-0.00022400	-0.06878900	-0.00393200
H	-2.01294000	3.16078900	2.08486700
H	-3.13584500	2.83420900	0.04584200
H	-3.52240300	4.53862000	0.31335100
H	-1.33748600	2.25580500	-2.03631400
H	-0.08783400	3.51784200	-2.05820800
I	-0.33695300	-0.55100300	-3.17160700
H	-3.95868800	1.18875500	1.37254900
H	-2.47336000	0.51201700	2.09042600
H	-5.59593000	0.21571300	-0.22021900
H	-5.30463700	-0.87636900	-1.57658600
H	-4.82364600	-0.77170200	2.35555400
O	-2.63280300	0.27173600	0.07888300
C	-3.56597400	0.25346200	-1.00939100
C	-4.85671700	-0.47985000	-0.65278000
C	-4.00611900	-0.98174500	1.64641200
C	-3.27001200	0.32385200	1.35555600
C	-4.55759200	-1.58664500	0.34892300
H	-3.32118500	-1.68645300	2.13846700
H	-3.80986400	-2.26258800	-0.09662800
H	-5.45476200	-2.19194600	0.54825300
H	-3.03275700	-0.24319300	-1.83334900
H	-3.77639500	1.29342200	-1.32131800
H	4.29427800	1.08363100	0.53871900
H	2.85531900	1.18896100	1.59325200
H	4.74269600	2.39560000	-1.72098100
H	3.52177100	2.70245300	-2.96097100
H	4.28719600	3.40337900	0.79252900
O	2.45446700	0.91377500	-0.37694800
C	3.03916500	1.03412400	-1.67822600
C	3.65519000	2.41756400	-1.90651200
C	3.29663500	3.03045800	0.48375300
C	3.27649100	1.50686000	0.62928400
C	3.00646400	3.42417600	-0.96595600
H	2.56094100	3.48875600	1.16043800
H	1.91855500	3.41682800	-1.13515600
H	3.36040100	4.44533300	-1.17219700
H	2.21658600	0.83566700	-2.38135700
H	3.79591900	0.23650800	-1.80362800
H	1.85234500	-3.90855800	1.06490800
H	1.16222100	-2.50774900	1.93750000
H	3.90274000	-4.05704800	-0.41763900
H	4.47588800	-2.84985000	-1.56981600
H	3.74518600	-3.21655200	2.29850700
O	1.60843900	-2.16427000	-0.01378300
C	2.37766400	-2.66358200	-1.11122700
C	3.81352200	-2.99472000	-0.70247800
C	3.32535200	-2.40520500	1.68116000
C	1.93039400	-2.81403200	1.21418800
C	4.22359600	-2.11723000	0.47278300
H	3.24755000	-1.51577400	2.32491700
H	4.10838300	-1.06382000	0.17709600
H	5.28340300	-2.26554000	0.72779600
H	2.33935200	-1.87227300	-1.87231900
H	1.86677200	-3.54714300	-1.53640700
O	-1.19676400	-2.26102500	0.69780000
C	-1.40248100	-3.42411400	0.38677300
C	-1.95260500	-4.38702500	1.39880400
C	-1.13141800	-3.93874300	-0.99162100
H	-3.02257500	-4.54627900	1.18242100
H	-1.85002700	-3.97657000	2.41048500
H	-1.46163900	-5.36853800	1.32407700
H	-1.96751600	-4.56342100	-1.34209400
H	-0.24646000	-4.59551400	-0.94793400
H	-0.93731100	-3.11566800	-1.69427000

$SmI_2(thp)_4^+$-ACE$^-$

H_{corr} = 0.7196
$-TS$ = -0.1135
$E_{sol(THP)}$ = -25915.8634
$G_{sol(THP)}$ = -25915.2573

I	-0.04487600	0.47203600	2.91323700
H	-0.50904800	3.97911300	-0.28345000
H	-0.14847600	2.88992100	1.08412500
H	-2.42772700	4.48720300	-1.53255100
H	-3.82874200	3.50744500	-1.95795800
H	-1.99325700	4.72757300	1.42337000
O	-1.22633900	2.04478500	-0.41164100
C	-2.08675100	2.34028000	-1.53055400
C	-2.96527400	3.55869800	-1.27709400
C	-2.17229400	3.68943800	1.09875000
C	-0.92715600	3.19944400	0.37713700
C	-3.40298500	3.60039200	0.18120100
Sm	-0.07166100	-0.26067700	-0.09337200
H	-2.31961700	3.09282300	2.01200300
H	-3.97216300	2.68444400	0.40546200
H	-4.08840500	4.44215600	0.35886600
H	-2.69816000	1.43787300	-1.66290800
H	-1.46419900	2.45719000	-2.43343600
I	0.00401500	-0.43747200	-3.15225700
H	-4.33292700	0.33106300	0.45974900
H	-2.86650000	0.71439600	1.40142700
H	-5.23457100	-1.66015100	-0.44649900
H	-4.45900300	-3.13808600	-1.01927400
H	-4.57672700	-1.01662700	2.40839800
O	-2.55700800	-0.48871700	-0.19963000
C	-3.17122500	-1.43789900	-1.09891200
C	-4.28695400	-2.22518100	-0.42884400
C	-3.68432000	-1.26366500	1.80961600
C	-3.40360300	-0.09050300	0.88268300
C	-3.89078200	-2.55584900	1.00378600
H	-2.84571100	-1.37587500	2.51319900
H	-2.95124400	-3.12961900	0.97041500
H	-4.64549600	-3.19860900	1.48038700
H	-2.35738100	-2.10657100	-1.40896600
H	-3.52467100	-0.88797900	-1.98811500
H	3.74815500	1.43933100	0.10977200
H	2.60714900	1.02569900	1.41306500
H	3.93204500	3.20417600	-1.42604300
H	2.71987500	4.24701900	-2.16406500
H	3.88359600	3.29703400	1.60123400
O	1.73117300	1.60943300	-0.32156400
C	1.93683800	2.35870600	-1.53552900
C	2.88362400	3.53512900	-1.34046200

C	2.89101500	3.16298900	1.14099900
C	2.81091900	1.74140200	0.60867400
C	2.65849100	4.18717700	0.01736700
H	2.15117400	3.29000300	1.94599300
H	1.62510800	4.56553500	0.06270200
H	3.31272800	5.06293200	0.14007300
H	0.93649400	2.70307000	-1.83446200
H	2.29067900	1.67182200	-2.32192900
H	2.36062600	-3.13610000	0.81328800
H	1.70262600	-1.94144500	1.95230000
H	4.32673100	-3.18389300	-0.58290000
H	5.12292500	-1.87245200	-1.45134400
H	4.18704400	-2.83371500	2.30933900
O	2.27079900	-1.17789100	0.15321300
C	3.05880100	-1.47437500	-1.01341700
C	4.40674900	-2.08573600	-0.64332100
C	3.88329300	-1.90507000	1.79908200
C	2.49390500	-2.11721000	1.21287700
C	4.88387200	-1.53426000	0.69391900
H	3.84952200	-1.11806700	2.56796600
H	4.96751000	-0.43766800	0.61807100
H	5.89080900	-1.90611500	0.93462900
H	3.18630000	-0.50801800	-1.52238200
H	2.47613100	-2.12991300	-1.68214100
O	-0.35038700	-2.31030000	0.19261300
C	-0.52108500	-3.63391000	0.26623000
C	-0.49517800	-4.23995400	1.62991900
C	-0.08003500	-4.42234400	-0.91994700
H	-0.98670800	-5.22579200	1.64293600
H	-0.99757600	-3.58195200	2.35550400
H	0.54087100	-4.39483400	2.00501500
H	-0.50865400	-5.43668900	-0.91759700
H	1.02751600	-4.53973900	-0.95150700
H	-0.36515900	-3.91253100	-1.85351800

$SmI_2(thp)_4$-HMAC

H_{corr} = 0.8985
$-TS$ = -0.1292
$E_{sol(THP)}$ = -26151.6503
$G_{sol(THP)}$ = -26150.8810

I	1.00119600	-0.11570200	-3.14997100
H	4.40869600	-1.72587300	-0.45154600
H	3.05588400	-1.48718300	-1.59272000
H	4.65768100	-3.37028400	1.46709400
H	3.40189800	-3.86285800	2.60619500
H	4.20638600	-3.92523400	-1.23108400
O	2.52002600	-1.55655200	0.36532900
C	2.99244900	-1.97440000	1.65312300
C	3.56580700	-3.39179800	1.62515100
C	3.25010200	-3.53615600	-0.84414600
C	3.36898600	-2.02050700	-0.68460500
C	2.91295000	-4.18478500	0.50177800
Sm	0.37156300	0.04612900	0.02605900
H	2.48367000	-3.78130700	-1.59422200
H	1.82125600	-4.17869600	0.65468100
H	3.22962000	-5.23834300	0.52055000
H	2.12093200	-1.89958700	2.31801200
H	3.74388000	-1.24801700	2.01444600
I	0.19978500	0.14547300	3.24784200
H	0.20062700	-4.18280200	-0.87830500
H	0.20404100	-2.78585800	-1.99482800
H	-1.68743700	-5.07309200	0.61215800
H	-2.85931000	-4.10876400	1.51134800
H	-1.66767500	-4.76539600	-2.16817800
O	-0.53542200	-2.39860300	-0.14702100
C	-1.05268700	-3.03035400	1.02896000
C	-2.12774200	-4.06467500	0.69005700
C	-1.75117900	-3.74492500	-1.75970900
C	-0.38330500	-3.31392100	-1.23117100
C	-2.78693600	-3.69809400	-0.63304500
H	-2.05924100	-3.09449100	-2.59232300
H	-3.19214400	-2.67782600	-0.55087400
H	-3.63681300	-4.36180200	-0.85220500
H	-1.45228300	-2.20954300	1.64183200
H	-0.21851400	-3.48527900	1.59340900
H	3.23393600	3.33736800	-0.72766400
H	2.47471300	2.06890100	-1.73668900
H	4.90757000	3.17963000	1.11416100
H	5.01519600	1.94486600	2.37086400
H	5.15411300	2.25621400	-1.56738000
O	2.43512100	1.71162200	0.26348900
C	3.05372600	2.09862200	1.49912300
C	4.57633400	2.16259500	1.38521100
C	4.47670700	1.56442000	-1.03994800
C	3.12256300	2.24624000	-0.86739500
C	5.05069600	1.17595500	0.32707400
H	4.36553200	0.67663700	-1.68053000
H	4.69317600	0.17193100	0.60273600
H	6.14921200	1.12536100	0.29319700
H	2.72895200	1.34531700	2.23034900
H	2.63500000	3.07089600	1.81906300
H	-1.64929800	3.55364800	-1.85262800
H	-0.29839300	2.56221500	-2.46848300
H	-1.58163100	5.22785500	0.25425200
H	-0.73126500	4.88341900	1.76497500
H	-0.11264800	5.29613100	-2.04879400
O	-0.60112600	2.44084700	-0.47042600
C	-1.21866000	3.11133300	0.63087100
C	-0.80863300	4.58472600	0.70883300
C	0.32363000	4.44013000	-1.50804700
C	-0.60903700	3.23360700	-1.65577200
C	0.50805200	4.78438500	-0.02903200
H	1.29669800	4.23240800	-1.97632400
H	1.26380600	4.11604900	0.41189300
H	0.87662200	5.81424900	0.08848400
H	-0.89836700	2.55553300	1.52617800
H	-2.31598300	3.00741700	0.54246800
O	-2.30289200	0.08271500	0.19761500
C	-3.50294000	0.23217600	0.01489800
C	-4.00846900	0.35962400	-1.43620700
C	-4.41670300	0.23697200	1.26004900
C	-5.14473500	-0.61905200	-1.75986500
C	-2.84362900	0.07379800	-2.38151500
C	-4.47246900	1.80697700	-1.68038700
C	-4.75677100	-1.23989700	1.53904300
C	-5.71134400	1.04583300	1.14615500
C	-3.62691700	0.78052500	2.45283700
H	-6.03669400	-0.48034800	-1.13620200
H	-4.81295800	-1.66313400	-1.66020300
H	-5.44857700	-0.47002000	-2.80760900
H	-3.19072300	0.12043700	-3.42440800
H	-2.41024700	-0.92017400	-2.20986400
H	-2.03143500	0.80149500	-2.26545700
H	-4.61181600	1.95194100	-2.76279100

H	-3.71016400	2.52799800	-1.34779400
H	-5.41988300	2.04820500	-1.18477400
H	-5.36156600	-1.29833000	2.45723200
H	-3.84015800	-1.82392700	1.70157900
H	-5.33075600	-1.70630700	0.72612500
H	-6.38011400	0.70992800	0.34357000
H	-5.50769700	2.11709900	1.00510100
H	-6.26431100	0.94376900	2.09278400
H	-3.39245400	1.84727000	2.31928500
H	-2.67763100	0.25175700	2.60580100
H	-4.23458200	0.68433300	3.36548300

$SmI_2(thp)_4^+$-HMAC$^-$

H_{corr} = 0.8972
$-TS$ = -0.1248
$E_{sol(THF)}$ = -26151.6191
$G_{sol(THF)}$ = -26150.8467

I	1.00505800	-0.22422300	-2.87903800
H	4.38461300	-0.80634700	0.32835000
H	3.35149300	-0.37146400	-1.06007000
H	4.76910700	-2.78290400	1.55163900
H	3.68257700	-4.11192400	1.94625100
H	5.09096700	-2.31530500	-1.37150200
O	2.41087000	-1.41134600	0.40321300
C	2.65536700	-2.27892700	1.52466600
C	3.80738100	-3.24725100	1.27628900
C	4.03420900	-2.43514100	-1.08071300
C	3.59746400	-1.17177300	-0.35477000
C	3.84479300	-3.66997700	-0.18585100
Sm	0.11549000	-0.03840100	0.10377600
H	3.45786000	-2.53438800	-2.01333700
H	2.89735400	-4.17288100	-0.43535400
H	4.64131900	-4.40886600	-0.35869700
H	1.71295600	-2.82151800	1.67615500
H	2.82502300	-1.66105300	2.42218600
I	0.30422700	0.03319600	3.16631400
H	0.59410700	-4.31513900	-0.41472600
H	1.01187600	-2.90875900	-1.43439000
H	-1.53388500	-5.14829800	0.44631000
H	-2.91270100	-4.22653800	1.04793600
H	-0.82680000	-4.69026000	-2.27244900
O	-0.20149900	-2.49996600	0.14390400
C	-1.06853400	-3.12619000	1.10763700
C	-1.99313700	-4.14551700	0.44930500
C	-0.99198400	-3.70873800	-1.79821200
C	0.19597000	-3.40373300	-0.89445300
C	-2.28714300	-3.70285900	-0.97802200
H	-1.04135400	-2.96273000	-2.60591300
H	-2.69619000	-2.68116900	-0.94534700
H	-3.05122700	-4.34172100	-1.44426000
H	-1.63471100	-2.29820500	1.55377200
H	-0.44270300	-3.57878700	1.89688400
H	2.24059200	3.53110600	-0.34562100
H	1.84744100	2.31485900	-1.59096100
H	3.94126500	3.73538200	1.26287700
H	4.84744400	2.51970100	2.15963700
H	4.18707200	3.46055400	-1.70874000
O	2.25670700	1.54848900	0.24022100
C	2.94756300	1.81657500	1.47680900
C	4.19885500	2.66311300	1.28172700
C	3.96676900	2.50574100	-1.20344300
C	2.51392300	2.54320200	-0.75197600
C	4.90844100	2.28327900	-0.00988100
H	4.09609500	1.71491700	-1.95801400
H	5.20285300	1.22336000	0.04421300
H	5.83930100	2.85735300	-0.12858200
H	3.19949900	0.82815700	1.88574500
H	2.24411100	2.28836900	2.18171300
H	-1.82361300	3.05616800	-1.74607200
H	-0.22907300	2.52227900	-2.30623500
H	-2.45575400	4.67948200	0.14204400
H	-1.67850500	4.78504200	1.72336300
H	-0.89312100	5.16830500	-2.07598700
O	-0.51261200	2.40665900	-0.29339300
C	-1.38542700	2.88730900	0.75011300
C	-1.54353400	4.40610700	0.69941300
C	-0.25590100	4.54431300	-1.42825200
C	-0.74463500	3.09813100	-1.52588900
C	-0.33393500	5.03273400	0.02091600
H	0.77049400	4.63911500	-1.81253400
H	0.57671500	4.73618900	0.56852600
H	-0.38107500	6.13106000	0.05866200
H	-0.90875900	2.56342100	1.68758000
H	-2.35304800	2.36771300	0.65480600
O	-2.01329300	-0.10430100	0.06328600
C	-3.33172200	0.13504600	-0.07423900
C	-3.87883100	0.26255500	-1.50624200
C	-4.20563800	-0.08880600	1.16483500
C	-4.95558900	-0.78402800	-1.85076500
C	-2.74879600	0.06934800	-2.51564500
C	-4.48062300	1.66076400	-1.75512700
C	-4.59695400	-1.57301900	1.32129500
C	-5.49067600	0.75000900	1.15439700
C	-3.43853200	0.30535000	2.42950500
H	-5.84809000	-0.70806400	-1.21542000
H	-4.56058000	-1.80702700	-1.76277300
H	-5.28469700	-0.64345300	-2.89375700
H	-3.14376600	0.15271000	-3.54025500
H	-2.27231200	-0.91520100	-2.41631600
H	-1.95713500	0.81927300	-2.40408200
H	-4.72862900	1.78302500	-2.82305600
H	-3.76503700	2.45113900	-1.48199400
H	-5.39847100	1.83341500	-1.17965800
H	-5.16203000	-1.72307300	2.25678100
H	-3.69947900	-2.20316700	1.37317200
H	-5.21847400	-1.93384300	0.49134700
H	-6.16897700	0.50067700	0.32736500
H	-5.26284800	1.82546900	1.09944200
H	-6.04351600	0.57375800	2.09120500
H	-3.16784800	1.37150100	2.42431400
H	-2.50971200	-0.26420000	2.54903700
H	-4.06674200	0.12603600	3.31663700

$SmI_2(thp)_4$-CPO

H_{corr} = 0.7589
$-TS$ = -0.1166
$E_{sol(THP)}$ = -25993.2758
$G_{sol(THP)}$ = -25992.6336

I	0.98046900	-0.09528500	3.11419900
H	4.02416500	2.16266300	0.19210200
H	2.96891800	1.40149500	1.42274000
H	3.63295700	4.11420300	-1.31828100
H	2.13183000	4.58294200	-2.11781700
H	3.52790600	4.02512500	1.55967700

O	2.08405600	1.73558800	-0.37205800
C	2.19652000	2.49942900	-1.57942600
C	2.54023000	3.96311900	-1.30485100
C	2.62292600	3.53621100	1.16294500
C	3.00884000	2.16049600	0.62965900
C	1.98610500	4.37922500	0.05169800
Sm	0.14065300	-0.00199600	0.01670800
H	1.92547800	3.42157900	2.00683600
H	0.89866900	4.21426300	0.04965900
H	2.14645200	5.45280800	0.23075400
H	1.22288800	2.39506300	-2.08016000
H	2.95355800	2.02733400	-2.23263400
I	-0.68144200	0.00669000	-3.09924600
H	-0.86428800	3.99565000	1.43531800
H	-0.64157400	2.36986500	2.13009600
H	-2.61421900	4.89244800	-0.09872300
H	-3.42418100	4.07801200	-1.43999600
H	-2.96151200	3.70802300	2.46805400
O	-0.99364700	2.39955500	0.12844100
C	-1.52825600	3.19848300	-0.93500300
C	-2.82348900	3.90091100	-0.53503000
C	-2.73113500	2.89946800	1.75500400
C	-1.24226100	2.95667000	1.41934900
C	-3.57159900	3.04919600	0.48076900
H	-2.95659800	1.94777400	2.25674900
H	-3.75350900	2.05904500	0.03145000
H	-4.55818100	3.48007000	0.70941400
H	-1.68962900	2.49447500	-1.76454500
H	-0.76499900	3.93175900	-1.25515700
H	3.48412100	-2.96445200	0.33420900
H	2.87123200	-1.68747200	1.42390000
H	4.66264700	-2.63981700	-2.03099300
H	4.16032300	-1.45440700	-3.24138600
H	5.44358100	-1.70508900	0.42225300
O	2.27907800	-1.51921900	-0.50903100
C	2.60115900	-1.95528100	-1.83383000
C	4.07496800	-1.71727300	-2.17628300
C	4.57317800	-1.07325400	0.17980800
C	3.29522400	-1.87878300	0.42817000
C	4.63427700	-0.62065200	-1.28012200
H	4.61533700	-0.20498700	0.85303000
H	4.02341700	0.28652500	-1.40706200
H	5.66455200	-0.36135900	-1.56623900
H	1.93406600	-1.38155800	-2.49435100
H	2.33951000	-3.02625800	-1.92982900
H	-2.00295100	-3.64399400	1.23083300
H	-1.18130900	-2.27538100	2.03668400
H	-1.05743100	-5.45777800	-0.26430200
H	0.21313800	-5.29309800	-1.47761300
H	-0.32125500	-4.80954000	2.41041800
O	-0.74190300	-2.49440300	0.06661900
C	-0.76924400	-3.43265500	-1.01276300
C	-0.23435300	-4.80346300	-0.59897600
C	0.09221800	-4.02347800	1.75712600
C	-1.04322100	-3.09955000	1.32355600
C	0.77447000	-4.63722300	0.52934600
H	0.81724000	-3.45227300	2.35674700
H	1.57450200	-3.96585200	0.18388600
H	1.24788600	-5.59732200	0.78287400
H	-0.16183600	-2.97627700	-1.80604300
H	-1.80235000	-3.50660300	-1.40110200
O	-2.29394800	-0.16132500	0.93878200
C	-3.39038500	-0.56954800	0.59877400
C	-4.62631700	-0.52985300	1.47153400
C	-3.74558900	-1.17509400	-0.73206300
H	-4.92327600	0.53198200	1.54620000
H	-4.39858700	-0.86623400	2.49278000
C	-5.66723900	-1.34340400	0.70379900
C	-5.27088400	-1.14300400	-0.76548400
H	-3.37684100	-2.21644500	-0.69187600
H	-3.20577800	-0.69266000	-1.56209400
H	-5.70599400	-1.89611400	-1.43637200
H	-5.61504900	-0.15508600	-1.11607200
H	-6.70075900	-1.03937200	0.91955500
H	-5.57714000	-2.41013500	0.96975300

$SmI_2(thp)_4^+$-CPO^-

H_{corr} = 0.7576
$-TS$ = -0.1143
$E_{sol(THP)}$ = -25993.2491
$G_{sol(THP)}$ = -25992.6057

I	-0.42477700	-0.03109700	-2.92223500
H	-4.22733000	0.68440200	-0.14557700
H	-3.00724300	0.20106100	-1.35699500
H	-4.75484600	2.71034200	0.95224100
H	-3.71708100	4.03882900	1.46561300
H	-4.66489100	2.16339200	-1.95925400
O	-2.28007700	1.28151900	0.19992200
C	-2.66590100	2.18441800	1.25555400
C	-3.75491900	3.15408000	0.81178100
C	-3.65457200	2.27227600	-1.53183600
C	-3.34649700	1.02487400	-0.71816000
C	-3.55913300	3.52831600	-0.65151600
Sm	0.00818100	0.08472600	0.14105500
H	-2.95450200	2.33358700	-2.37897700
H	-2.56701800	3.99228500	-0.76780200
H	-4.29439900	4.28439300	-0.96395400
H	-1.74554200	2.72189700	1.51628700
H	-2.97050100	1.59091400	2.13427600
I	-0.11068600	0.06534700	3.20247400
H	-0.21342100	4.34913800	-0.69222800
H	-0.52224000	2.83840500	-1.58799200
H	1.53552200	5.18157600	0.58200200
H	2.85198500	4.41199900	1.46709000
H	1.48840000	4.27734200	-2.38201000
O	0.29401100	2.58084900	0.24937100
C	1.14031200	3.15933600	1.26921600
C	2.06936900	4.22543400	0.71556500
C	1.55712800	3.47425200	-1.62992900
C	0.20359000	3.35923200	-0.94859400
C	2.66859600	3.75514600	-0.60314600
H	1.76239700	2.54100200	-2.17545900
H	3.23270000	2.82859700	-0.40908100
H	3.38440100	4.49545000	-0.98986700
H	1.71736000	2.31954500	1.68098700
H	0.48819500	3.55059000	2.06849100
H	-1.72705300	-3.71440700	-0.12036700
H	-1.14251400	-2.63136600	-1.40842800
H	-3.67819900	-3.79854400	1.20367800
H	-4.75034500	-2.52569200	1.78061000
H	-3.41932800	-3.86752200	-1.79410900
O	-1.89377300	-1.68036400	0.21883800
C	-2.77248200	-1.83190600	1.35113100
C	-3.95573400	-2.74235700	1.05018000

C	-3.30325900	-2.85728100	-1.36864200
C	-1.94784400	-2.79050000	-0.68278100
C	-4.43733300	-2.53982500	-0.38012700
H	-3.31478200	-2.15453000	-2.21571000
H	-4.75894500	-1.49352700	-0.50182700
H	-5.32264600	-3.16023100	-0.58344200
H	-3.10791000	-0.81298700	1.59090400
H	-2.18427700	-2.19082200	2.21167100
H	2.92525400	-2.40968900	-0.48037400
H	1.80217500	-1.80858500	-1.72071400
H	2.85984000	-4.30947000	1.01620300
H	1.48830600	-5.05480600	1.83614400
H	2.70453900	-4.31151900	-1.88857900
O	0.93036800	-2.27654600	0.05512700
C	1.13720900	-3.01345200	1.27387400
C	1.75936700	-4.38053600	1.00959500
C	1.74553900	-3.97682400	-1.46041200
C	1.93059300	-2.56017000	-0.93185600
C	1.29017800	-4.91835000	-0.33560400
H	1.01454800	-3.97858300	-2.28344900
H	0.18983900	-4.98672400	-0.32749000
H	1.66350200	-5.93952300	-0.50276400
H	0.13649800	-3.11036100	1.71935500
H	1.75077300	-2.40640300	1.96046300
O	2.08042800	0.33867200	-0.02416900
C	3.40041100	0.35986900	-0.12097600
C	4.29458000	-0.04570800	1.00844700
C	4.12193200	0.33679000	-1.43852600
H	4.11280300	-1.10441600	1.30737000
H	4.13655000	0.55079800	1.92346000
C	5.68982200	0.11287900	0.40047400
H	3.57816700	-0.24998200	-2.20050200
C	5.48824000	-0.25915900	-1.07415300
H	4.23300800	1.35214200	-1.87329800
H	6.45766500	-0.49936300	0.89695300
H	6.00813000	1.16670900	0.47698700
H	6.30756100	0.08705600	-1.72229400
H	5.44438100	-1.35870400	-1.16637900

Beyond SET

THF-based reaction **Complex**

H_{corr} = 0.7617

$-TS$ = -0.1423

$E_{sol(THF)}$ = -25993.2675

$G_{sol(THF)}$ = -25992.6481

H	-6.64439300	0.71974200	-1.37540400
H	-4.31480700	1.79502000	-2.05699300
H	-2.91558200	-1.47334200	-0.96209300
C	-3.48763700	1.47500600	1.98628100
C	-3.94421000	0.76139700	-2.15582200
H	-2.85942200	0.74501400	-1.99431800
C	-3.18476000	0.60514100	0.78623500
C	-4.27146600	-0.24078600	0.27151600
C	-4.67578900	-0.16810700	-1.22181300
C	-3.96976000	-1.36585600	-0.69723700
H	-4.53208400	-2.29215500	-0.56020800
O	-2.06669000	0.60435000	0.27490700
Sm	0.36034100	0.02188000	-0.02151800
O	0.21221000	2.56776200	-0.45728300
C	-0.67156900	3.18662700	-1.40673300
C	-0.64338100	4.67486400	-1.08794600
C	0.74151700	4.84745000	-0.47028100
C	0.87662300	3.56453800	0.32885400
I	-0.10979800	-0.57324500	-3.13309800
I	0.96071300	0.51893200	3.09945300
O	2.12501700	-1.91250900	0.00061200
C	2.89444800	-2.33192800	-1.13714600
C	3.57869200	-3.61744600	-0.70616100
C	3.79528500	-3.36769100	0.78403300
C	2.50855900	-2.65744000	1.16668500
O	-0.81811400	-2.22510300	0.56792400
C	-0.78435400	-3.39281600	-0.27005300
C	-1.67336000	-4.41479100	0.41969000
C	-1.50937100	-4.03523900	1.88899900
C	-1.47941600	-2.52029700	1.80704000
O	2.73305400	0.97227100	-0.54976600
C	3.07366500	1.60918600	-1.79168700
C	4.59130600	1.64181800	-1.81974900
C	4.92260900	1.79730900	-0.33775300
C	3.88343500	0.89523200	0.30559900
H	3.62990900	-1.54262200	-1.37409200
H	2.21229100	-2.43094400	-1.99360700
H	1.70602600	-3.37935700	1.40285100
H	2.59681000	-1.95561400	2.00860500
H	2.90804000	-4.47939100	-0.85652600
H	4.50616100	-3.80794700	-1.26378300
H	3.96039400	-4.28345300	1.36845700
H	4.66173000	-2.70409000	0.93961100
H	0.26098200	-3.73841000	-0.33623600
H	-1.11157600	-3.10544500	-1.27991100
H	-2.50200400	-2.10444200	1.77149500
H	-0.92028300	-2.02415800	2.61387100
H	-2.72226900	-4.28480500	0.10829700
H	-1.37685000	-5.44824700	0.19284400
H	-2.31674900	-4.40704700	2.53504300
H	-0.55306900	-4.41670500	2.28264300
H	2.64805300	2.62821400	-1.79292400
H	2.60574700	1.03801300	-2.60682100
H	4.22601800	-0.15393300	0.33994800
H	3.57625300	1.18921900	1.31978200
H	4.98916100	0.68828600	-2.20404700
H	4.98370200	2.45230000	-2.44943100
H	5.95025600	1.50638300	-0.07945700
H	4.77449800	2.84255500	-0.02046700
H	-1.66736200	2.73375900	-1.29991700
H	-0.29789700	2.96173100	-2.41882800
H	1.91123700	3.23062900	0.48648000
H	0.38708100	3.63745300	1.31475500
H	-0.81004000	5.29613200	-1.97868500
H	-1.41897400	4.92620900	-0.34675200
H	1.51094400	4.89339400	-1.25850300
H	0.83596800	5.74639500	0.15449400
H	-4.11540900	0.43968000	-3.19481300
H	-6.65087200	-0.95605200	-0.76632500
H	-6.34978300	-0.63432200	-2.49101700
H	-5.11701700	-0.36209700	0.95201600
C	-6.16136700	-0.26594600	-1.46976500
H	-4.54457800	1.78469900	1.89971400
C	-3.33740000	0.61432600	3.24893300
C	-2.58470700	2.69754600	2.04225600
H	-2.28703200	0.30610900	3.37216300
H	-3.62545200	1.19900500	4.13525600

H	-3.97236000	-0.28475600	3.21568300
H	-2.67948800	3.30775700	1.13129800
H	-2.85146400	3.32425600	2.90607400
H	-1.53346700	2.39263000	2.15207700

THF-based reaction **Int I**
H_{corr} = 0.7605
$-TS$ = -0.1376
$E_{sol(THF)}$ = -25993.2409
$G_{sol(THF)}$ = -25992.6180

H	-6.68229200	0.61200700	-1.15781300
H	-4.41730200	1.80074700	-1.92590300
H	-2.84882300	-1.44828300	-1.04255800
C	-3.27684600	1.52772200	2.01033000
C	-4.03464000	0.77661800	-2.07536300
H	-2.94264600	0.79149500	-1.96450000
C	-3.02862700	0.65548400	0.80805000
C	-4.10859600	-0.22947700	0.32705500
C	-4.66870700	-0.17999700	-1.10068300
C	-3.86260600	-1.36087100	-0.64424500
H	-4.38434700	-2.30697100	-0.47504600
O	-1.76082600	0.41445900	0.45951300
Sm	0.25444500	-0.00165200	0.04756400
O	0.18011800	2.40857800	-0.54096700
C	-0.89827300	2.94312000	-1.34635400
C	-0.83178400	4.45263100	-1.16864600
C	0.63084800	4.68556200	-0.80080000
C	0.91894400	3.47951700	0.07008300
I	-0.25678100	-0.77240700	-2.89942000
I	1.27779800	0.75553200	2.85683300
O	2.10661200	-1.81703800	0.04596100
C	2.75413200	-2.35770400	-1.12137700
C	3.45007000	-3.61943500	-0.64574600
C	3.81825400	-3.25127900	0.78802600
C	2.59554700	-2.47101300	1.23436300
O	-0.54309600	-2.21912100	0.83885700
C	-0.59105200	-3.43547500	0.06591300
C	-1.72907100	-4.24749200	0.65948400
C	-1.72178200	-3.79215800	2.11588700
C	-1.44119800	-2.31054700	1.96842400
O	2.55536300	0.86236700	-0.82233500
C	2.76976200	1.45442000	-2.12008600
C	4.27562500	1.57417300	-2.26347100
C	4.70823700	1.80765600	-0.81969900
C	3.78288200	0.86949000	-0.06816800
H	3.47571600	-1.61190400	-1.49743600
H	1.98971200	-2.51232000	-1.89470000
H	1.80069700	-3.13337400	1.61669100
H	2.79016500	-1.69809800	1.99027900
H	2.75166200	-4.47190800	-0.65659500
H	4.31386300	-3.88121100	-1.27226800
H	4.01677700	-4.11932800	1.43158300
H	4.71269400	-2.60727500	0.80119000
H	0.37783200	-3.95093300	0.17862400
H	-0.73051800	-3.16811700	-0.99139800
H	-2.35096600	-1.74045200	1.73102800
H	-0.93688200	-1.84757200	2.82759000
H	-2.68103200	-3.97337300	0.18053200
H	-1.57695500	-5.32822400	0.53262100
H	-2.66836500	-3.98400300	2.63936300
H	-0.91206400	-4.28919100	2.67476400
H	2.27922600	2.44191400	-2.13598200
H	2.28313100	0.81386000	-2.86796500
H	4.18555400	-0.15749900	-0.04338200
H	3.54643000	1.17417100	0.96067500
H	4.70276900	0.63201900	-2.64394800
H	4.56667700	2.38071400	-2.95042400
H	5.76837100	1.58817100	-0.63214800
H	4.52008700	2.85330000	-0.52582500
H	-1.83803800	2.49705600	-0.99527100
H	-0.71739600	2.62564300	-2.38510300
H	1.97316700	3.17648000	0.10609400
H	0.56307800	3.61837400	1.10395600
H	-1.14702600	4.98886900	-2.07396900
H	-1.48227600	4.76914800	-0.33898800
H	1.26587900	4.67148700	-1.70190900
H	0.80413200	5.63342300	-0.27289200
H	-4.25496400	0.47881400	-3.11346800
H	-6.56618800	-1.03470800	-0.49137400
H	-6.40844400	-0.77152500	-2.24438100
H	-4.88915700	-0.41308600	1.07621800
C	-6.15804400	-0.35364800	-1.25444400
H	-4.35848800	1.75304600	2.01618800
C	-2.93702100	0.79629900	3.31686900
C	-2.51541100	2.84777000	1.93064200
H	-1.86309900	0.55443000	3.34859800
H	-3.16714400	1.42036300	4.19618200
H	-3.50943400	-0.14077900	3.40978900
H	-2.81971000	3.42447900	1.04324900
H	-2.69993000	3.46685900	2.82269800
H	-1.43281500	2.65788200	1.87102700

THF-based reaction **TS I**
H_{corr} = 0.7586
$-TS$ = -0.1383
$E_{sol(THF)}$ = -25993.2262
$G_{sol(THF)}$ = -25992.6059

H	-6.72871200	0.90752500	-0.91892800
H	-4.47639900	2.09297300	-1.52806200
H	-2.97888300	-1.24679300	-1.02815100
C	-3.13756900	1.28137400	2.39925700
C	-4.10477600	1.09605500	-1.82570800
H	-3.01582300	1.06782400	-1.69182100
C	-2.95066800	0.38128000	1.20178400
C	-4.01440100	-0.34694300	0.66782700
C	-4.76830800	0.00542300	-1.05756800
C	-3.97660300	-1.15866000	-0.58254200
H	-4.49926000	-2.12480000	-0.55364100
O	-1.72431300	0.34867800	0.66128900
Sm	0.24288900	-0.05356100	0.03407200
O	0.19749200	2.40904400	-0.31551500
C	-0.89086100	3.04235200	-1.03142700
C	-0.79418400	4.52240500	-0.69596000
C	0.68481800	4.69321300	-0.36049200
C	0.97718300	3.40201700	0.37522000
I	-0.46218500	-0.43772900	-2.93458600
I	1.64738800	0.41924300	2.74686000
O	1.94955500	-1.96341800	-0.35975500
C	2.46086700	-2.40628000	-1.63426700
C	3.32866200	-3.61811200	-1.33152800
C	3.78313400	-3.34565200	0.09881400
C	2.53115500	-2.74245300	0.70161200
O	-0.54106600	-2.28902600	0.77516400
C	-0.83110900	-3.38347600	-0.11696400

C	-1.93253300	-4.17642800	0.56439700
C	-1.63785500	-3.92861000	2.04090900
C	-1.24065100	-2.46634000	2.02878500
O	2.51461600	0.81670100	-0.91092000
C	2.68890200	1.58402000	-2.10712700
C	3.87544800	2.51700300	-1.83200100
C	4.52612600	1.93322200	-0.56295900
C	3.82577600	0.59453600	-0.39056100
H	3.03783500	-1.57706200	-2.07358300
H	1.61112000	-2.61063800	-2.29914500
H	1.81223600	-3.52219500	1.00849500
H	2.70143800	-2.07125100	1.55433400
H	2.72778700	-4.54104800	-1.36895300
H	4.15528300	-3.72557600	-2.04726600
H	4.11669600	-4.24469000	0.63511100
H	4.60542600	-2.61230600	0.11316900
H	0.08882000	-3.98112700	-0.23724700
H	-1.11275200	-2.97022200	-1.09641800
H	-2.11908100	-1.80243400	2.03058100
H	-0.55411200	-2.16603400	2.83232100
H	-2.91696600	-3.76206700	0.29940000
H	-1.91571200	-5.23773100	0.28070100
H	-2.49903100	-4.11778300	2.69619400
H	-0.80031800	-4.55933000	2.38131300
H	1.73910600	2.09152900	-2.30926500
H	2.89142800	0.89341400	-2.94342000
H	4.32245800	-0.19778500	-0.98154000
H	3.71645700	0.27108700	0.65222900
H	4.56807700	2.52613700	-2.68521700
H	3.54575600	3.55318700	-1.67030000
H	5.61553300	1.82101600	-0.65059000
H	4.32703300	2.56870200	0.31269400
H	-1.82710000	2.57348300	-0.70470500
H	-0.74469100	2.83453800	-2.10313100
H	2.02385300	3.07665100	0.34839900
H	0.65485600	3.44129900	1.42867000
H	-1.13241100	5.15622700	-1.52685200
H	-1.41049900	4.75554100	0.18591700
H	1.28787400	4.75643800	-1.28141000
H	0.89628800	5.58072800	0.25158100
H	-4.29340800	0.99219000	-2.91225500
H	-6.64418700	-0.76578000	-0.31122100
H	-6.62580100	-0.45131600	-2.05773100
H	-4.91814000	-0.41440400	1.27728400
C	-6.25819400	-0.07725300	-1.08004000
H	-4.21978100	1.30130100	2.61415400
C	-2.40919300	0.74235000	3.63234200
C	-2.68565800	2.71019700	2.09792900
H	-1.32874500	0.65263400	3.43898300
H	-2.54128700	1.41423200	4.49607400
H	-2.79148900	-0.25100300	3.91500500
H	-3.23546900	3.12894900	1.24001200
H	-2.84639700	3.36951500	2.96568400
H	-1.61113300	2.71957200	1.85904600

THF-based reaction **Int II**

H_{corr} = 0.7597

$-TS$ = -0.1397

$E_{sol(THF)}$ = -25993.2483

$G_{sol(THF)}$ = -25992.6283

H	-5.99972800	-1.54598900	-2.50894600
H	-5.37634800	0.88593500	-2.55972400
H	-2.97246500	-1.07827500	-0.01420700
C	-2.71222000	2.40391200	2.21585200
C	-4.52816600	0.69005500	-1.88260900
H	-4.37531100	1.57334300	-1.24319800
C	-2.74086200	1.18616300	1.31561200
C	-3.88562500	0.50952100	1.07148100
C	-4.74728000	-0.53711200	-1.06887100
C	-3.99467200	-0.71384600	0.21869900
H	-4.48277700	-1.52685900	0.79143500
O	-1.56194900	0.81828500	0.81230600
Sm	0.26032900	0.03461000	0.09665200
O	0.16516800	2.16139000	-1.16835100
C	-1.02200900	2.53746900	-1.90737500
C	-0.90963200	4.03866400	-2.11609100
C	0.59961200	4.26426900	-2.09440000
C	1.03689800	3.29513300	-1.01408700
I	-0.91736100	-1.38400300	-2.39461500
I	1.94060300	1.35962300	2.31105700
O	1.90873400	-1.94422100	0.25447900
C	2.25969000	-2.84071700	-0.81761800
C	2.92072500	-4.02959200	-0.14537200
C	3.59535100	-3.37457400	1.05581000
C	2.55719000	-2.35134200	1.47682400
O	-0.55790000	-1.78905200	1.57810100
C	-0.88487000	-3.13198500	1.16523800
C	-1.96374300	-3.59546100	2.12708300
C	-1.59952400	-2.83755100	3.40012400
C	-1.19779500	-1.48765100	2.84124700
O	2.36986400	0.37433400	-1.38808200
C	2.34064600	0.58535400	-2.81472900
C	3.77178300	0.38954400	-3.27557200
C	4.55452700	0.92028900	-2.07931300
C	3.73214000	0.39941400	-0.91495200
H	2.95578400	-2.31723100	-1.49550100
H	1.34530500	-3.07344200	-1.37960300
H	1.79483600	-2.78791700	2.14368800
H	2.96241700	-1.45129400	1.95889600
H	2.16082200	-4.75448800	0.18871500
H	3.61742900	-4.55299700	-0.81453000
H	3.84014200	-4.07711400	1.86422900
H	4.52728100	-2.87349800	0.74704700
H	0.02661400	-3.74871900	1.24859600
H	-1.20054700	-3.10187000	0.11266300
H	-2.07375700	-0.85196000	2.64306100
H	-0.47438700	-0.93018300	3.45212200
H	-2.95396700	-3.27827600	1.76550200
H	-1.97201100	-4.68751900	2.24727500
H	-2.42932400	-2.75760500	4.11551500
H	-0.74970000	-3.32055900	3.90968800
H	1.99048200	1.61308500	-3.01004100
H	1.61461600	-0.11811200	-3.24405800
H	4.02320800	-0.62865500	-0.64136000
H	3.76677000	1.02038200	-0.00948300
H	3.98314300	-0.68112300	-3.43047900
H	3.98433900	0.91778900	-4.21522200
H	5.59695200	0.57520100	-2.04075900
H	4.56142200	2.02249000	-2.08446900
H	-1.89736100	2.22835900	-1.32124500
H	-1.01319400	1.97366600	-2.85318800
H	2.07012500	2.93541600	-1.10712600
H	0.90658300	3.71209000	-0.00224300
H	-1.38811000	4.36169300	-3.05054700

H	-1.38349000	4.57897400	-1.28262700
H	1.04681200	3.98900000	-3.06373900
H	0.88520800	5.30022200	-1.86585100
H	-3.62539700	0.58552700	-2.52154200
H	-5.61718500	-2.51155100	-1.05482100
H	-4.38623600	-2.26044300	-2.31123400
H	-4.80497400	0.90306600	1.51453100
C	-5.21790500	-1.76745100	-1.76399000
H	-3.76151700	2.65150700	2.44836900
C	-1.98277800	2.10030200	3.52407400
C	-2.07632000	3.59578000	1.50646000
H	-0.93728600	1.81475600	3.33049100
H	-1.97265600	2.98539200	4.17999800
H	-2.47505600	1.27983600	4.06910900
H	-2.62871100	3.85069700	0.58811300
H	-2.06801600	4.48308000	2.15839700
H	-1.03528400	3.36308500	1.23566300

THF-based reaction **PreTS II** with phenylacetylene
H_{corr} = 0.8811
$-TS$ = -0.1597
$E_{sol(THF)}$ = -26301.5090
$G_{sol(THF)}$ = -26300.8066

H	-0.14816300	4.94684000	1.37911300
H	1.25986500	2.60674000	-0.10045200
H	2.11849400	3.73445300	0.97627500
C	6.57896800	-1.40580900	0.95853700
H	0.60274400	3.33250100	-2.38467800
H	2.50193400	6.39396200	-1.52722000
C	-1.05532400	3.08480700	1.09142000
C	-0.01862900	3.94351400	0.96284000
C	1.27745300	3.64720200	0.26164200
C	1.51676700	4.58031900	-0.89156100
C	2.10403500	5.92220300	-0.61395300
O	-1.03109000	1.83518300	0.62941000
Sm	-0.82555600	-0.20166700	0.10157300
O	-2.68682800	0.46246400	-1.39421100
C	-2.58698400	1.63831800	-2.23320900
C	-4.01732900	1.98933700	-2.60805900
C	-4.71885800	0.63562700	-2.53877700
C	-4.05030600	0.00799700	-1.33186500
I	1.05816300	0.25292800	-2.31378100
I	-2.83703500	-1.21051300	2.20600700
O	0.51197600	-2.35671100	0.54969600
C	1.39140900	-3.02409700	-0.37846700
C	2.21137800	-3.98756300	0.46062000
C	1.24129900	-4.33678000	1.58407600
C	0.57224400	-3.00027500	1.83930400
O	0.96716100	0.09425300	1.78314000
C	2.37780400	0.00930400	1.47517300
C	3.07520900	0.86451000	2.51909700
C	2.10864700	0.79871000	3.69785200
C	0.76911800	0.85710600	2.99351300
O	-1.69281900	-2.21461500	-1.30936100
C	-1.78372500	-2.23616300	-2.74867900
C	-1.96450700	-3.69622700	-3.11466200
C	-2.78144500	-4.20850500	-1.93343800
C	-2.13495400	-3.47773300	-0.77117200
H	0.76981500	-3.54941600	-1.12368600
H	1.98294700	-2.26125300	-0.90160600
H	1.16733900	-2.36579400	2.51710100
H	-0.45180300	-3.06243300	2.23172200
H	3.10292100	-3.47992300	0.86229300
H	2.55024700	-4.85781900	-0.11873800
H	1.73120500	-4.73545900	2.48303800
H	0.50191700	-5.07889800	1.24114600
H	2.67783100	-1.04922100	1.54188200
H	2.53182800	0.35872700	0.44591000
H	0.49676700	1.88824900	2.71969600
H	-0.06382100	0.39368800	3.53992500
H	3.17130700	1.89826700	2.15433000
H	4.08074100	0.48786800	2.74827500
H	2.23600100	1.62125400	4.41497800
H	2.22047400	-0.15364700	4.24185900
H	-2.65609400	-1.63423300	-3.05482500
H	-0.87573800	-1.76607000	-3.14928200
H	-1.25284400	-4.02060900	-0.39303300
H	-2.80408800	-3.27075400	0.07503500
H	-0.98790800	-4.20564800	-3.15809200
H	-2.45958700	-3.82543900	-4.08701000
H	-2.74932400	-5.29978700	-1.80935600
H	-3.83690800	-3.90898200	-2.03898100
H	-2.06554300	2.41785500	-1.66122000
H	-1.97172600	1.36855600	-3.10554500
H	-4.03462800	-1.08989600	-1.32909200
H	-4.49836600	0.34826800	-0.38421100
H	-4.07932400	2.46529500	-3.59610400
H	-4.45162200	2.67728300	-1.86688300
H	-4.51125300	0.04487200	-3.44611800
H	-5.80837500	0.71150300	-2.41957300
H	-0.35273800	4.76427400	-1.94652100
H	1.34740100	6.62628800	-0.20444000
H	1.08674700	4.95283900	-2.97362900
H	2.91420000	5.86897800	0.13240700
C	0.68263600	4.39718700	-2.11240300
C	-2.31952000	3.48619000	1.82558600
C	4.74784700	1.40649500	-0.68203300
C	-2.41488900	2.77966700	3.17737900
C	-3.55827900	3.20792100	0.98018800
H	6.50429500	0.73670100	1.23649300
C	4.25664600	2.48856600	-0.93094500
H	-2.25207700	4.57269600	2.00289100
H	3.76266400	3.41813200	-1.15788700
H	4.07335900	-0.82417300	-1.95046700
H	-2.44535700	1.68713400	3.04566100
H	-1.55354900	3.03176300	3.81526600
H	-3.63433600	2.13152800	0.76491300
H	-3.51631300	3.75403800	0.02444900
H	4.86898900	-3.12773700	-1.42930400
H	6.46265600	-3.50151000	0.44869300
H	7.28214800	-1.56332700	1.77874900
C	6.14797800	-0.11735100	0.65846200
C	5.24247600	0.09921200	-0.39256600
C	4.78613100	-0.99820600	-1.14260900
C	5.22656500	-2.28197500	-0.83806500
C	6.11963700	-2.49196600	0.21343600
H	-4.47310300	3.51471600	1.51060500
H	-3.33174100	3.07828300	3.71039400

THF-based reaction **TS II** with phenylacetylene
H_{corr} = 0.8798
$-TS$ = -0.1563
$E_{sol(THF)}$ = -26301.5029
$G_{sol(THF)}$ = -26300.7794

H	2.07406400	4.05294000	1.74405200
H	2.20594000	1.53543700	-0.07653900
H	3.52569500	2.00935900	1.02103200
C	6.12538900	-2.02454100	1.07477200
H	1.87704000	2.73603600	-2.20688700
H	4.87588500	4.67310700	-1.12890300
C	0.39104400	2.89069500	1.32311500
C	1.71225100	3.16211000	1.22224100
C	2.70345500	2.39809400	0.39163500
C	3.29034100	3.27512600	-0.67668400
C	4.31829500	4.27591800	-0.26432500
O	-0.18749100	1.83890000	0.74466600
Sm	-0.90556500	-0.03852000	0.09226500
O	-2.37008400	1.47724300	-1.21172200
C	-1.82277000	2.56347900	-1.99699900
C	-2.97792300	3.52656800	-2.21308200
C	-4.19137900	2.60275900	-2.15696800
C	-3.79185800	1.63922400	-1.05688600
I	0.91425600	-0.17600700	-2.40858000
I	-3.10660800	-0.34287900	2.23044800
O	-0.50083200	-2.58260300	0.26133200
C	0.03368400	-3.44460600	-0.76234800
C	0.44848700	-4.71338000	-0.04138600
C	-0.58530500	-4.78904200	1.07740000
C	-0.71856300	-3.33127500	1.47619600
O	0.79365200	-0.65912600	1.78155000
C	2.08387400	-1.19963300	1.41762600
C	3.01917600	-0.83673500	2.55876400
C	2.06273800	-0.69954000	3.73946400
C	0.87095700	-0.03710900	3.08213500
O	-2.56788900	-1.37652400	-1.39482900
C	-2.73260100	-1.19328700	-2.81601800
C	-3.46665900	-2.43044200	-3.29420200
C	-4.35705600	-2.73910400	-2.09542000
C	-3.42487400	-2.44171400	-0.93563000
H	-0.75921400	-3.63631000	-1.50635700
H	0.84991600	-2.90984800	-1.26521800
H	0.05010100	-3.03182200	2.20700700
H	-1.70217600	-3.04908400	1.87609900
H	1.46167500	-4.60140600	0.37834400
H	0.44938300	-5.58915700	-0.70487500
H	-0.28091100	-5.42449200	1.92035000
H	-1.54257600	-5.17388200	0.68950100
H	1.97318900	-2.29014300	1.30460700
H	2.38569100	-0.77955800	0.44923200
H	1.03459900	1.04293100	2.94541700
H	-0.09396800	-0.20318400	3.57989200
H	3.51146600	0.12531800	2.35211300
H	3.80273600	-1.59235800	2.69749400
H	2.47000300	-0.09834200	4.56395300
H	1.78587600	-1.68948600	4.13826700
H	-3.33030900	-0.28175000	-2.98597100
H	-1.73546200	-1.04898200	-3.25295600
H	-2.79424900	-3.31292800	-0.69289600
H	-3.92283100	-2.10358200	-0.01665900
H	-2.75600600	-3.25460200	-3.46951600
H	-4.02109500	-2.25394100	-4.22623500
H	-4.73307000	-3.77132900	-2.07386400
H	-5.22480500	-2.05984600	-2.07732000
H	-0.97781000	2.98925500	-1.43912700
H	-1.44574200	2.13433800	-2.93843400
H	-4.25006200	0.64347600	-1.12475100
H	-3.99622300	2.04324200	-0.05219400
H	-2.88661200	4.07290300	-3.16171200
H	-3.02383300	4.26206800	-1.39566000
H	-4.31478200	2.06767400	-3.11290400
H	-5.13123300	3.12527100	-1.93198100
H	1.64241500	4.36378300	-1.52477800
H	3.85083700	5.15125500	0.23249500
H	2.97764300	4.07634900	-2.65938600
H	5.04218000	3.84552500	0.44538100
C	2.40978000	3.62268200	-1.82982700
C	-0.53376400	3.77923700	2.13316200
C	4.99407300	0.63921500	-1.27778200
C	-1.02027000	3.08252700	3.40273200
C	-1.71616200	4.24059300	1.28555000
C	4.83730100	1.73661000	-1.80105800
H	4.94010800	2.55063700	-2.49497100
H	0.05401300	4.66587200	2.42422100
H	3.68956900	-1.59669600	-1.91705600
H	3.93449200	-3.81335500	-0.82030100
H	-1.57024300	2.16001000	3.16068100
H	-0.17656000	2.82231500	4.06041100
H	-2.31044400	3.37234600	0.96335800
H	-1.37500600	4.78041500	0.38784800
H	5.49396800	-4.09232900	1.10688300
H	6.81368100	-2.13933600	1.91492500
H	6.57255500	0.07193600	0.81143500
C	5.99442500	-0.78434300	0.46081500
C	5.11660400	-0.61549800	-0.62810000
C	4.37710400	-1.72734800	-1.08035800
C	4.51267000	-2.96117600	-0.45494500
C	5.38516200	-3.11921700	0.62388900
H	-2.37450200	4.91059300	1.86017600
H	-1.69994600	3.73841000	3.96999500

THF-based reaction **Int III** with phenylacetylene

H_{corr} = 0.8828

$-TS$ = -0.1545

$E_{sol(THF)}$ = -26301.5550

$G_{sol(THF)}$ = -26300.8267

H	2.24210100	3.99842900	1.60840000
H	2.30850200	1.33850200	0.00597000
H	3.61473900	1.88428000	1.07448100
C	6.46325000	-1.97304800	0.90754700
H	1.97570800	2.43190000	-2.24706100
H	4.89651400	4.57695400	-1.18244700
C	0.53320100	2.86253500	1.24821100
C	1.85809900	3.10139900	1.11515700
C	2.82560500	2.23448100	0.38163100
C	3.53648900	2.91180000	-0.80595300
C	4.31879100	4.14775300	-0.34827900
O	-0.07572800	1.80010400	0.72410800
Sm	-0.92555400	-0.03532300	0.10754500
O	-2.24310100	1.55454400	-1.26062600
C	-1.58871600	2.57283100	-2.05515600
C	-2.66331400	3.60538600	-2.35248000
C	-3.94231800	2.77371900	-2.30759600
C	-3.65175200	1.82882000	-1.15850700
I	0.88262200	-0.42846700	-2.38311800
I	-3.12395400	-0.07170000	2.26015300
O	-0.80843800	-2.59612400	0.37338000
C	-0.41526000	-3.54443400	-0.63824000
C	-0.09288100	-4.82223000	0.11332200

C	-1.06784900	-4.75068300	1.28414000
C	-1.03404100	-3.27336700	1.62805600
O	0.81782800	-0.76813000	1.70900900
C	2.00258200	-1.50214400	1.32380200
C	3.06060900	-1.13360800	2.34651900
C	2.22123100	-0.82434800	3.58203900
C	1.04129100	-0.09715500	2.97004500
O	-2.71688000	-1.26000000	-1.34220100
C	-2.83164400	-1.14959500	-2.77531500
C	-3.67983400	-2.33232600	-3.19799500
C	-4.63133400	-2.46290100	-2.01371200
C	-3.71010400	-2.17900400	-0.84086500
H	-1.26130500	-3.68256900	-1.33373800
H	0.42333900	-3.11392300	-1.20131000
H	-0.20109700	-3.02672900	2.30668900
H	-1.96243400	-2.87835700	2.06280800
H	0.94731900	-4.80178000	0.47707600
H	-0.21640600	-5.71670000	-0.51263800
H	-0.78375100	-5.38282000	2.13661900
H	-2.07841900	-5.04941400	0.96096600
H	1.76082800	-2.57813200	1.35177000
H	2.26580400	-1.22786500	0.29424600
H	1.27553500	0.95911500	2.76697800
H	0.10694100	-0.15926400	3.54483100
H	3.60454700	-0.23641500	2.01700700
H	3.79490200	-1.93776700	2.48628500
H	2.74620100	-0.20817500	4.32484800
H	1.89279700	-1.75394900	4.07563500
H	-3.32735800	-0.19392400	-3.01757700
H	-1.81617100	-1.13568800	-3.19288800
H	-3.19374000	-3.09144300	-0.50022800
H	-4.19836700	-1.71465200	0.02651900
H	-3.05650100	-3.23623600	-3.29519000
H	-4.18628100	-2.16263100	-4.15817600
H	-5.11295400	-3.44741000	-1.93564500
H	-5.42485300	-1.70048800	-2.07506800
H	-0.73942500	2.95815800	-1.47501200
H	-1.20352000	2.08365100	-2.96313400
H	-4.18168700	0.86827300	-1.20351300
H	-3.85664900	2.28977100	-0.17854400
H	-2.49902400	4.10499600	-3.31683700
H	-2.68576000	4.37483600	-1.56588400
H	-4.07141700	2.21108300	-3.24678600
H	-4.84849100	3.37109200	-2.13729100
H	1.79088300	4.02075200	-1.45165100
H	3.63611600	4.92830400	0.02055100
H	3.01743000	3.80114700	-2.73132200
H	5.02528700	3.89357500	0.45769700
C	2.52014100	3.31352200	-1.87734000
C	-0.35324000	3.79052000	2.05494200
C	4.84151000	0.75145200	-1.00327900
C	-0.78951900	3.13220700	3.36311700
C	-1.56475800	4.23697700	1.24263800
C	4.54083100	1.94810900	-1.42562300
H	5.06459300	2.34087300	-2.31372600
H	0.25411700	4.67916500	2.29547300
H	3.72612700	-1.53330500	-1.82508300
H	4.28598600	-3.79640700	-0.98350600
H	-1.36521800	2.21461300	3.16613500
H	0.08204100	2.87184700	3.98360100
H	-2.17681100	3.36545600	0.96627400
H	-1.25530900	4.75312900	0.31989700
H	6.03177600	-4.09336700	0.77761800
H	7.23314400	-2.09835100	1.67229000
H	6.69063700	0.16924200	0.83585300
C	6.16691100	-0.70408100	0.44394300
C	5.16238300	-0.50895600	-0.55498400
C	4.49550700	-1.66754700	-1.06320300
C	4.80956200	-2.92564400	-0.58135000
C	5.78892000	-3.09585500	0.40735900
H	-2.19635300	4.92548600	1.82520800
H	-1.43116400	3.81133400	3.94701400

THF-based reaction **TS III** with phenylacetylene

H_{corr} = 0.8819

$-TS$ = -0.1517

$E_{sol(THF)}$ = -26301.5360

$G_{sol(THF)}$ = -26301.5515

C	-2.53075100	2.62745600	0.24747800
C	-2.90000100	1.63605600	1.30655500
C	-4.27124000	0.68113300	-0.35277900
C	-4.48767600	1.82318800	-0.97423700
C	-3.71045700	3.07055000	-0.62848200
C	-2.05287300	0.66482200	1.77945500
H	-5.45308800	-0.76679900	1.59394200
H	-2.09252200	3.53150300	0.71267600
C	-4.62710500	4.04502700	0.12079800
C	-3.18475800	3.73933200	-1.89904000
H	-4.01146100	4.07228100	-2.54715500
H	-2.58162300	4.62804300	-1.64668900
H	-5.27412800	1.91090600	-1.74188700
H	-1.75991700	2.17923700	-0.39673400
O	-0.94402100	0.31754900	1.14547700
Sm	0.85763000	-0.04460900	0.09751900
O	-0.05839700	-2.32851700	0.06950100
C	-1.40668000	-2.59515600	-0.39863000
C	-1.74775400	-3.97750200	0.12762700
C	-0.37873400	-4.64340000	0.22385600
C	0.49066700	-3.49833100	0.70424900
I	-0.35728800	0.51117300	-2.67550200
I	2.46578400	-0.76820200	2.62639000
O	3.02047200	1.06357300	-0.77549700
C	3.37850800	1.22207900	-2.16320900
C	4.54331400	2.19536700	-2.16896800
C	5.22391300	1.88027100	-0.84072600
C	4.03325300	1.64593900	0.06925400
O	1.00328600	2.37717400	0.64535900
C	1.03747100	3.47484300	-0.28932200
C	0.71330700	4.72123700	0.52283300
C	1.12251000	4.31709200	1.93734000
C	0.70757300	2.86097800	1.97220500
O	2.42057200	-1.69129300	-1.16019200
C	2.01890000	-2.49219100	-2.29140500
C	3.29709000	-3.12588100	-2.80740700
C	4.09917800	-3.29813100	-1.52157700
C	3.77382600	-2.01322100	-0.78382100
H	3.66771700	0.23462000	-2.56112100
H	2.48785600	1.56145200	-2.70889000
H	3.64173200	2.59129800	0.48197700
H	4.21399000	0.95489200	0.90367400
H	4.17743600	3.23502800	-2.17511800
H	5.19400700	2.06103600	-3.04396800
H	5.87634500	2.68517200	-0.47536400
H	5.83010800	0.96376100	-0.92646100

H	2.05061500	3.51389900	-0.72045300
H	0.32100800	3.27005600	-1.09782300
H	-0.37043000	2.73910800	2.16277100
H	1.27014100	2.23639500	2.67956800
H	-0.36620100	4.93098500	0.48492700
H	1.24452000	5.60711500	0.14836700
H	0.63000400	4.91069600	2.71958800
H	2.21272600	4.40928500	2.07097500
H	1.30101100	-3.25188900	-1.94091600
H	1.50666600	-1.83353500	-3.00583100
H	4.43253800	-1.18834800	-1.10500800
H	3.81297800	-2.08368700	0.31204700
H	3.81759600	-2.43998300	-3.49551200
H	3.10945600	-4.06664800	-3.34293200
H	5.17837100	-3.42466500	-1.68433400
H	3.73550200	-4.17107500	-0.95534300
H	-2.05966600	-1.79681700	-0.02727600
H	-1.39131000	-2.55094800	-1.49867600
H	1.54767300	-3.57255800	0.41554400
H	0.44093500	-3.36440100	1.79678600
H	-2.45155200	-4.50107500	-0.53124300
H	-2.21000300	-3.90445500	1.12318500
H	-0.04589200	-4.99151900	-0.76808700
H	-0.35375700	-5.49950900	0.91209000
H	-2.55219400	3.04459300	-2.47237300
H	-6.03870600	-3.17512100	1.48098900
H	-5.50790500	4.30228400	-0.48888000
H	-4.99224800	3.61005200	1.06341600
H	-4.09461900	4.98201400	0.35474500
H	-3.74271300	1.88308900	1.95865200
C	-4.63856400	-0.68788900	-0.40721100
C	-4.35186100	-1.45389100	-1.56254100
C	-4.69055000	-2.79943400	-1.62270900
C	-5.29889100	-3.42922700	-0.53302500
C	-5.56636000	-2.69104300	0.62305700
C	-5.24115000	-1.34227700	0.69082500
H	-3.85635500	-0.96285300	-2.40221700
H	-4.47732800	-3.36720200	-2.53193000
H	-5.56152000	-4.48778800	-0.58303300
H	-2.97218200	-1.94726400	2.17342500
C	-2.33349100	-0.06771900	3.07286000
H	-1.23326000	-1.86650300	2.58293500
C	-1.36787100	0.41397100	4.15898500
C	-2.24374100	-1.58143600	2.91217100
H	-0.32734000	0.19214500	3.87355400
H	-1.46091300	1.49933400	4.32030800
H	-3.36116600	0.19691400	3.37476000
H	-2.44294300	-2.08532600	3.87093900
H	-1.57068700	-0.09246300	5.11620100

THF-based reaction **Int IV** with phenylacetylene

H_{corr} = 0.8847

$-TS$ = -0.1509

$E_{sol(THF)}$ = -26301.5983

$G_{sol(THF)}$ = -26300.8644

C	-2.821806	2.564302	0.633062
C	-3.297533	1.168050	1.090813
C	-3.898657	0.622775	-0.205560
C	-4.099844	1.607529	-1.093061
C	-3.691921	2.964563	-0.574470
C	-2.228497	0.319488	1.714790
C	-4.937476	3.742536	-0.131038
H	-3.556208	4.015942	-2.479885
H	-2.561304	4.723703	-1.189617
H	-2.054107	3.198952	-1.986122
C	-2.918377	3.770738	-1.614965
H	-5.301660	-3.509511	1.392218
H	-4.575929	1.480111	-2.068841
H	-1.782160	2.446261	0.293435
O	-1.063584	0.212568	1.075132
Sm	0.772127	-0.029918	0.084576
O	0.036068	-2.384671	0.160783
C	-1.303398	-2.746911	-0.262709
C	-1.529423	-4.152282	0.266608
C	-0.112608	-4.710894	0.354486
C	0.667155	-3.496532	0.818034
I	-0.439485	0.436392	-2.709239
I	2.420825	-0.579422	2.642456
O	2.914096	1.153470	-0.830097
C	3.261085	1.297273	-2.220454
C	4.384659	2.316965	-2.250434
C	5.090880	2.042758	-0.926586
C	3.920714	1.765562	-0.000716
O	0.865286	2.409825	0.591299
C	0.872667	3.485377	-0.368066
C	0.377797	4.704179	0.390147
C	0.859953	4.415510	1.808843
C	0.618463	2.922890	1.919219
O	2.429954	-1.622021	-1.137631
C	2.066919	-2.470232	-2.246034
C	3.370291	-3.079022	-2.729518
C	4.169169	-3.174461	-1.433537
C	3.792473	-1.874916	-0.747484
H	3.590536	0.315210	-2.601381
H	2.355685	1.588080	-2.769795
H	3.504746	2.695876	0.422595
H	4.139065	1.080435	0.829682
H	3.976032	3.340554	-2.263002
H	5.031366	2.200042	-3.130954
H	5.713029	2.877093	-0.574509
H	5.734696	1.152194	-1.012125
H	1.907058	3.620466	-0.726049
H	0.241782	3.191270	-1.218620
H	-0.421886	2.689599	2.189023
H	1.292758	2.396059	2.608272
H	-0.721121	4.748568	0.363006
H	0.770861	5.640615	-0.029106
H	0.318107	4.980807	2.579332
H	1.933875	4.642973	1.908471
H	1.368939	-3.240033	-1.878107
H	1.542092	-1.851240	-2.986617
H	4.420768	-1.039405	-1.100945
H	3.830740	-1.899578	0.350631
H	3.872443	-2.402363	-3.440008
H	3.219160	-4.045417	-3.229858
H	5.253272	-3.268329	-1.585607
H	3.832871	-4.038048	-0.836789
H	-2.001133	-2.003186	0.138747
H	-1.327003	-2.696288	-1.362119
H	1.727947	-3.495333	0.534865
H	0.600461	-3.351887	1.908387
H	-2.196347	-4.729824	-0.386255
H	-1.987639	-4.113637	1.266728
H	0.237537	-5.039750	-0.638067

H	-0.015472	-5.556970	1.048835
H	-5.629800	3.895917	-0.974258
H	-4.696992	-1.131245	1.697994
H	-5.484254	3.201147	0.656614
H	-2.844999	3.309285	1.444232
H	-4.659804	4.734781	0.261826
H	-4.109955	1.266736	1.835140
C	-4.251109	-0.787512	-0.388275
C	-4.187954	-1.388226	-1.655012
C	-4.529259	-2.726302	-1.827224
C	-4.932813	-3.496842	-0.735871
C	-4.988383	-2.915851	0.530574
C	-4.645217	-1.577231	0.703767
H	-3.828089	-0.800102	-2.501054
H	-4.468536	-3.175131	-2.821219
H	-5.198791	-4.547424	-0.871286
H	-0.750466	-1.665712	2.958577
C	-2.290686	-0.151793	3.141813
H	-2.403931	-2.274663	2.661109
C	-1.500293	0.763564	4.087253
C	-1.800762	-1.593113	3.280534
H	-0.431315	0.744401	3.823966
H	-1.858664	1.803872	4.028671
H	-3.353135	-0.113721	3.445043
H	-1.856632	-1.932785	4.326869
H	-1.593139	0.431186	5.134291

THF-based reaction **Product** with phenylacetylene
H_{corr} = 0.8860
$-TS$ = -0.1555
$E_{sol(THF)}$ = -26301.6179
$G_{sol(THF)}$ = -26300.8874

C	-3.03143400	2.54726300	0.66146200
C	-3.51706500	1.15322200	1.10819800
C	-4.00251100	0.55477200	-0.20753300
C	-4.16495200	1.51856900	-1.12449500
C	-3.82875200	2.89476500	-0.61463600
C	-2.39787500	0.33263600	1.71911600
C	-5.11570500	3.65796400	-0.27843500
H	-3.58845800	3.93883200	-2.51036800
H	-2.63764200	4.62949000	-1.17712100
H	-2.12707100	3.09210000	-1.95274300
C	-2.99299900	3.68415400	-1.61932100
H	-5.23855000	-3.66389700	1.32109000
H	-4.54792700	1.34927900	-2.13318200
H	-1.96696200	2.45640100	0.39968700
O	-1.37606800	0.13995500	1.07570500
Sm	0.85721200	-0.03618800	-0.00587800
O	0.06160300	-2.48662800	0.24770400
C	-1.17703100	-2.99478200	-0.28062500
C	-1.34539600	-4.37936700	0.32472100
C	0.09939700	-4.80780200	0.56295200
C	0.73520200	-3.50156300	1.00260700
I	-0.47039700	0.34356700	-2.89943900
I	2.31462400	-0.36076200	2.85321500
O	2.96663800	1.28533500	-0.83723400
C	3.37906000	1.34230800	-2.21221600
C	4.53256300	2.33003400	-2.24441200
C	5.16568700	2.11296400	-0.87260400
C	3.94093900	1.92062200	0.00443700
O	0.57622000	2.50461800	0.54068700
C	0.69807300	3.52664700	-0.46239200
C	0.40832400	4.83179500	0.25629300
C	0.95746500	4.54326800	1.65075200
C	0.54213800	3.09654000	1.85094300
O	2.61827800	-1.64875500	-1.06029800
C	2.32098000	-2.54878900	-2.14053000
C	3.65241800	-3.17328800	-2.52088200
C	4.37826700	-3.19684000	-1.17827600
C	3.95211600	-1.86825400	-0.57920700
H	3.69795000	0.33220400	-2.52329700
H	2.50797500	1.62466400	-2.82072800
H	3.53509800	2.89116300	0.34385900
H	4.09518800	1.28642800	0.88925800
H	4.15400000	3.36169300	-2.33192200
H	5.21832600	2.14828200	-3.08353300
H	5.79602200	2.94833500	-0.53757400
H	5.78408200	1.20044500	-0.87520400
H	1.72555500	3.50291300	-0.86252800
H	0.00592100	3.28595000	-1.28079200
H	-0.48978000	3.02787100	2.23727700
H	1.20444600	2.51220300	2.50627900
H	-0.67777800	5.01166200	0.30265700
H	0.87736900	5.69504900	-0.23590000
H	0.55769700	5.20630400	2.43045300
H	2.05605500	4.63228300	1.65788000
H	1.60627000	-3.30600700	-1.77509900
H	1.83357900	-1.97247300	-2.94030000
H	4.59745900	-1.04493000	-0.93455700
H	3.92829300	-1.84045100	0.52034400
H	4.19043900	-2.52865000	-3.23498400
H	3.53588100	-4.16499400	-2.97944700
H	5.46972000	-3.28952000	-1.26501800
H	4.01309300	-4.03264400	-0.55911500
H	-1.98294600	-2.29903100	-0.01609000
H	-1.09673600	-3.02214900	-1.37966900
H	1.81020500	-3.42434100	0.78936200
H	0.58950600	-3.30924600	2.07873800
H	-1.90735700	-5.05410100	-0.33478900
H	-1.88713400	-4.31607100	1.28192200
H	0.55531600	-5.16208900	-0.37649300
H	0.20633700	-5.60173300	1.31516800
H	-5.74964800	3.77392500	-1.17139900
H	-4.84086100	-1.24966100	1.65378600
H	-5.70718500	3.12872100	0.48511600
H	-3.13746300	3.30685900	1.45111800
H	-4.88257200	4.66585700	0.10217600
H	-4.34700200	1.22271600	1.83265400
C	-4.25310900	-0.87777000	-0.39545200
C	-4.05274500	-1.47190500	-1.65172400
C	-4.29324400	-2.82873200	-1.83902600
C	-4.72164500	-3.62461200	-0.77560500
C	-4.90647000	-3.05046800	0.48093600
C	-4.67348600	-1.68997300	0.66854300
H	-3.65201900	-0.86729100	-2.46725200
H	-4.12260100	-3.27343000	-2.82164200
H	-4.90214500	-4.69124300	-0.92364500
H	-0.75402900	-1.43888000	3.07315000
C	-2.52310800	-0.18905200	3.13068000
H	-2.26477100	-2.29016900	2.63635400
C	-1.92032200	0.87883800	4.05565300
C	-1.82638000	-1.53100800	3.30162200
H	-0.83958500	0.96947900	3.86573000
H	-2.39256300	1.86388800	3.91302600

H	-3.59761000	-0.28158800	3.36343400
H	-1.92795600	-1.87997100	4.33991200
H	-2.06039000	0.58539700	5.10651800

THF-based reaction **PreTS II** with acrylonitrile
H_{corr} = 0.8189
$-TS$ = -0.1542
$E_{sol(THF)}$ = -26164.0181
$G_{sol(THF)}$ = -26163.3534

H	3.18456100	3.59804800	0.97050600
H	2.63639600	0.54526900	0.89319800
H	3.69738100	1.31751200	2.10262200
N	7.96999000	-1.95396600	-1.24294100
H	3.43020500	0.25526200	-1.44164100
H	6.66181200	1.39004200	-0.02521400
C	1.33608200	2.78827100	0.41672500
C	2.61842200	2.67277400	0.83071700
C	3.34330100	1.37531900	1.05502300
C	4.51913900	1.22914100	0.13278700
C	5.81727600	1.85518700	0.50940300
O	0.54298300	1.73682000	0.20703900
Sm	-0.73620400	0.06436700	0.04817300
O	-0.57213100	0.73879800	-2.32313600
C	0.71676000	0.99135700	-2.93463600
C	0.42048800	1.80509200	-4.18355900
C	-1.00103400	1.37329100	-4.53240500
C	-1.62745800	1.24930100	-3.15796100
I	1.47722400	-2.05167100	-0.52378700
I	-3.29924000	1.71090700	0.42029700
O	-1.92736200	-1.79514900	1.36952300
C	-1.72173700	-3.21468000	1.22074300
C	-2.34866900	-3.84311200	2.45128400
C	-3.49732600	-2.88331400	2.74321700
C	-2.86568500	-1.53889300	2.43636600
O	-0.11913300	0.04690200	2.46414900
C	0.55564400	-1.02332200	3.15548400
C	1.39426900	-0.34151400	4.21921000
C	0.52646900	0.86184100	4.57402700
C	0.01744200	1.28237700	3.20917300
O	-2.20280100	-1.44552300	-1.48196400
C	-1.71339700	-2.20440000	-2.60787700
C	-2.84001600	-3.15161900	-2.97293300
C	-4.06877000	-2.32001300	-2.62154400
C	-3.62548500	-1.64090000	-1.33950000
H	-2.22315600	-3.54282900	0.29423200
H	-0.64431300	-3.39476200	1.10871700
H	-2.30695800	-1.13912900	3.29916800
H	-3.56714700	-0.76759800	2.09086200
H	-1.63140100	-3.84964200	3.28787300
H	-2.67005800	-4.87834400	2.27241600
H	-3.87243600	-2.94183700	3.77413100
H	-4.34232200	-3.07599900	2.06232600
H	-0.20660000	-1.68422600	3.60399300
H	1.13567800	-1.59490900	2.41741500
H	0.74136100	1.92216500	2.68377300
H	-0.96417900	1.77615300	3.21711800
H	2.35205000	-0.00838200	3.78973300
H	1.60651600	-1.00284800	5.07041200
H	1.07841600	1.66961500	5.07350800
H	-0.30589100	0.55723900	5.22913000
H	-1.49013400	-1.50426000	-3.43029600
H	-0.77880100	-2.69449100	-2.30445900
H	-3.80206000	-2.28241700	-0.45971400
H	-4.08519100	-0.66152900	-1.15074700
H	-2.79781100	-4.05895200	-2.34854600
H	-2.79965700	-3.46180200	-4.02611000
H	-4.98312900	-2.91348400	-2.48426200
H	-4.26386700	-1.57183400	-3.40709500
H	1.34457100	1.51126500	-2.19920300
H	1.16930800	0.01304300	-3.15955800
H	-2.46955900	0.54767800	-3.09576500
H	-1.95793400	2.22339700	-2.76229000
H	1.14709000	1.61049400	-4.98385300
H	0.44162100	2.88141100	-3.95467700
H	-0.99546100	0.39658000	-5.04357900
H	-1.53357500	2.09205300	-5.17010700
H	3.95360600	1.91352800	-1.82854300
H	5.84082400	2.93531500	0.25114800
H	5.14360900	0.59143900	-1.82465800
H	6.00861600	1.78662200	1.59277100
C	4.25007000	0.97848100	-1.30939400
C	0.69737100	4.14536100	0.20217400
C	5.54434200	-2.00301200	-0.33858100
C	-0.33939200	4.44181800	1.28507400
C	0.07598200	4.24715000	-1.18759100
C	5.18137600	-1.39453200	0.80267300
H	5.91288000	-0.90051400	1.44382600
H	1.50664100	4.89061000	0.27974400
H	4.80021700	-2.50568900	-0.96215400
H	-0.36531400	5.24309600	-1.34728200
H	-1.15130500	3.69885800	1.26196600
H	0.12084800	4.43009000	2.28536500
H	-0.72402000	3.50029600	-1.30192100
H	0.83081200	4.07758800	-1.97168500
H	-0.79117700	5.43455600	1.13067700
H	4.14190600	-1.44121800	1.13070600
C	6.88214600	-1.97741600	-0.82810100

THF-based reaction **TS II** with acrylonitrile
H_{corr} = 0.8181
$-TS$ = -0.1510
$E_{sol(THF)}$ = -26164.0188
$G_{sol(THF)}$ = -26163.3518

H	3.34050400	3.27063500	1.34601600
H	2.65240700	0.28969500	0.83170500
H	3.81068600	0.82858400	2.07861100
N	8.37210800	-1.52811400	-0.96248000
H	3.30282500	0.34773000	-1.57131300
H	6.65535000	1.22101700	-0.17707400
C	1.44129600	2.62252900	0.74799100
C	2.73246000	2.40021300	1.08468500
C	3.40460400	1.05705100	1.07439200
C	4.52453400	1.00814300	0.07267200
C	5.83506700	1.61631900	0.44363000
O	0.60143700	1.64504500	0.40813800
Sm	-0.74084800	0.04837800	0.06152000
O	-0.70525000	1.09917000	-2.17429900
C	0.54674300	1.43431300	-2.82196700
C	0.18982200	2.45214900	-3.89293900
C	-1.26251400	2.10667900	-4.20946600
C	-1.79952600	1.76095700	-2.83529700
I	1.35574500	-1.99900100	-0.97862300
I	-3.21969400	1.68546000	0.84437400
O	-1.93143300	-1.96106000	1.14239600

C	-1.77472200	-3.34536700	0.77022900
C	-2.36735600	-4.14038700	1.91884900
C	-3.47048500	-3.20872200	2.41019600
C	-2.81022400	-1.84929000	2.28178100
O	0.00476000	-0.37085200	2.40043600
C	0.66807900	-1.55807600	2.87973000
C	1.56607000	-1.08316200	4.00576000
C	0.75310000	0.06987100	4.58586000
C	0.21008800	0.72220700	3.32956800
O	-2.33950800	-1.16874700	-1.59035200
C	-1.94512400	-1.73725400	-2.85776600
C	-3.14090500	-2.54408200	-3.32757700
C	-4.30310400	-1.74237500	-2.75194000
C	-3.75770900	-1.34589200	-1.39374100
H	-2.32415300	-3.51583800	-0.17130200
H	-0.70887700	-3.53107100	0.58199000
H	-2.19971300	-1.60207600	3.16660200
H	-3.50021500	-1.01565600	2.09355900
H	-1.61486700	-4.29486300	2.70896600
H	-2.72977500	-5.12697100	1.59904100
H	-3.80076900	-3.41641800	3.43715600
H	-4.35079400	-3.27273000	1.75014000
H	-0.09937200	-2.26267700	3.24469800
H	1.20201100	-2.01616100	2.03533400
H	0.93555300	1.41936100	2.88531500
H	-0.75329700	1.23491300	3.45824700
H	2.51955300	-0.71221800	3.59852300
H	1.78475700	-1.88059000	4.72905800
H	1.34965900	0.76916800	5.18728200
H	-0.06562300	-0.31420200	5.21589600
H	-1.71346400	-0.91159100	-3.55089500
H	-1.02978700	-2.32220300	-2.69719900
H	-3.91084900	-2.14562400	-0.64927200
H	-4.16170400	-0.40955500	-0.98600000
H	-3.11903400	-3.55569500	-2.89062800
H	-3.17024700	-2.64729100	-4.42096600
H	-5.24054800	-2.31027400	-2.67591000
H	-4.49594100	-0.84767300	-3.36625000
H	1.23370400	1.81359000	-2.05406200
H	0.96011500	0.50323400	-3.24012300
H	-2.65424300	1.07205500	-2.83309900
H	-2.07660400	2.65780000	-2.25786700
H	0.85635800	2.38494500	-4.76331600
H	0.25447500	3.47375700	-3.48882500
H	-1.31677200	1.23083700	-4.87673200
H	-1.81685500	2.93103700	-4.67849500
H	3.87552500	2.03143000	-1.69805400
H	5.82058700	2.71465200	0.28787000
H	5.00644300	0.69097600	-1.99861900
H	6.08549300	1.44595400	1.50276800
C	4.15876800	1.01034500	-1.37202700
C	0.84964800	4.01696900	0.78078600
C	5.81220000	-1.81034800	-0.72300100
C	-0.13024600	4.16923700	1.94349400
C	0.17858200	4.36098900	-0.54550700
C	5.12868500	-1.39921600	0.37204400
H	5.65234600	-1.05471500	1.26505400
H	1.69038600	4.71297500	0.93902300
H	5.28424200	-2.22610600	-1.58476700
H	-0.23270600	5.38197200	-0.52305100
H	-0.97108000	3.46648700	1.84006200
H	0.36907100	3.98166800	2.90681000
H	-0.65106900	3.66576700	-0.74349300
H	0.89574000	4.29945700	-1.37933500
H	-0.54783200	5.18822500	1.97009700
H	4.05573300	-1.58763500	0.43677200
C	7.21879500	-1.65738800	-0.84914700

THF-based reaction **Int III** with acrylonitrile

H_{corr} = 0.8209

$-TS$ = -0.1475

$E_{sol(THF)}$ = -26164.0653

$G_{sol(THF)}$ = -26163.3920

H	3.87207800	-2.14830300	3.27377300
H	4.27370900	-0.74162400	4.28960000
H	5.80062000	-1.17747100	2.07042700
H	5.18404000	0.47120500	2.33888100
H	-1.01274200	2.48328000	1.67243900
H	-0.30243800	1.73655800	3.13320500
H	3.00918100	2.15103800	1.61810300
H	2.15189100	3.39525200	0.67248400
H	-0.06819600	4.06490400	3.77137000
H	0.09166500	4.58679900	2.07552900
H	2.18280200	3.06662400	3.73139300
H	2.43299000	4.56778000	2.80177400
Sm	0.58513600	0.06637400	-0.09570400
O	0.96776100	1.91121000	1.51475300
C	-0.11900200	2.44373300	2.30940100
C	0.36262800	3.80030500	2.79618700
C	1.87768900	3.62019800	2.82818600
C	2.11136900	2.78203200	1.58711600
I	-0.94477100	-1.38347300	2.17569800
I	2.66513100	1.28524300	-2.00914100
O	1.67457100	-2.21394100	-0.57987900
C	1.71055200	-3.35287800	0.30437400
C	2.11165500	-4.52823100	-0.56791500
C	3.00198600	-3.85964200	-1.61010200
C	2.27186200	-2.55196600	-1.84936700
O	-0.56465500	-1.18938500	-1.88793700
C	-1.25726700	-2.44935700	-1.72480800
C	-2.31606400	-2.46637100	-2.81025700
C	-1.65612600	-1.64449300	-3.91220200
C	-0.99366100	-0.54334600	-3.10953200
O	2.65785200	-0.39758700	1.43187400
C	2.64172500	-0.41378500	2.87435600
C	3.95639900	-1.04933700	3.28369700
C	4.88618000	-0.57677600	2.17152500
C	3.98791600	-0.68604100	0.95402600
H	2.45544800	-3.15378400	1.09389500
H	0.72590200	-3.44993000	0.78019000
H	1.46536700	-2.66099100	-2.59351600
H	2.91328700	-1.71379100	-2.15381500
H	1.22401000	-4.97062100	-1.04795900
H	2.61591000	-5.31846300	0.00505300
H	3.12019600	-4.44513700	-2.53217000
H	4.00586000	-3.67071000	-1.19593100
H	-0.52243100	-3.26121500	-1.85819600
H	-1.66212900	-2.49973600	-0.70576600
H	-1.70494700	0.25516600	-2.84900200
H	-0.10424400	-0.09856500	-3.57724100
H	-3.22953400	-1.97018500	-2.45104500
H	-2.58614100	-3.48850800	-3.10492300
H	-2.36708100	-1.24674800	-4.64931400
H	-0.90246200	-2.24358800	-4.44949400

H	2.56692600	0.62590400	3.23525400
H	1.74482600	-0.95977100	3.19529500
H	3.99474000	-1.70782100	0.53907300
H	4.21706100	0.01973900	0.14425400
H	-3.59173500	2.30188000	1.53885000
H	-3.93055100	2.52549800	-1.62773800
C	-4.06349700	1.34235800	1.80235700
H	-2.38744400	4.03052700	-2.02096100
H	0.33219900	3.67250100	-0.65124500
H	-6.18618600	-0.37013900	1.79159100
C	-0.57635700	4.24489400	-0.89000100
H	-0.26770800	5.18904500	-1.36482700
H	-3.26384200	0.65933400	2.12744400
H	-1.09456900	4.48842400	0.05111900
H	-0.46632800	4.10352700	-3.62901800
C	-5.50682600	-0.55299400	0.93951400
H	-3.02279200	-0.09485200	-0.23892300
H	-6.44500900	1.43399300	-0.71788600
C	-4.58767500	-1.66615700	1.30196800
H	-6.13095600	-0.85593400	0.08412000
H	-6.67622500	1.85273200	0.99839800
C	-0.78181700	3.16225600	-3.15118300
O	-0.97601600	1.38628400	-0.65039300
C	-1.93753600	2.15157500	-1.16284200
H	-4.74554700	1.51743800	2.65048200
C	-1.47812600	3.43983100	-1.81943700
H	-4.38298500	0.02075300	-1.37261100
C	-5.92600500	1.76688100	0.19590000
H	-5.52097900	2.77201000	0.00985100
C	-3.24832100	1.81573800	-1.15263600
C	-4.81598900	0.79311600	0.59250300
C	-3.83377200	0.56768800	-0.58074300
C	-4.33427200	-2.72872100	0.44411500
N	-4.12832700	-3.61769800	-0.29715100
H	0.11836800	2.54676500	-3.00285900
H	-4.02912600	-1.64927000	2.24006500
H	-1.45594100	2.63767600	-3.84590400

THF-based reaction **TS III** with acrylonitrile

H_{corr} = 0.8202

$-TS$ = -0.1458

$E_{sol(THF)}$ = -26164.0616

$G_{sol(THF)}$ = -26163.3873

H	4.47189500	0.28736000	1.55993000
H	2.50738000	-1.34420000	-0.19278900
H	3.55563700	-2.04780200	1.06241900
N	6.50442600	2.24124700	0.07708900
H	6.23365800	-0.66531400	0.48010400
H	5.07282300	-2.87363200	-2.33785600
C	2.46616500	0.83997400	1.32038300
C	3.57890800	0.09146200	0.96054900
C	3.48790700	-1.25047300	0.29859800
C	4.59123600	-1.46745600	-0.74459200
C	4.31642900	-2.73394600	-1.54895200
O	1.26986600	0.61887500	0.79882500
Sm	-0.64548600	0.04098200	0.08305500
O	-0.47204500	2.27574100	-0.95753800
C	0.67524600	2.65243400	-1.75524900
C	0.64789900	4.16981100	-1.81295000
C	-0.84011500	4.47664800	-1.66882400
C	-1.27333200	3.43485700	-0.65638800
I	0.41039000	-1.18501800	-2.54011200
I	-2.16149600	1.24439100	2.46505800
O	-2.39461400	-1.84588000	0.09011200
C	-2.82056900	-2.60548500	-1.05928300
C	-3.57635300	-3.79437600	-0.49574600
C	-4.18982900	-3.20501300	0.77040200
C	-3.06813000	-2.31768900	1.27589000
O	0.07638100	-1.92711600	1.40600700
C	0.32058400	-3.25924100	0.90673600
C	1.17723200	-3.94204500	1.96177500
C	0.79572300	-3.19138000	3.23497400
C	0.65501600	-1.77403100	2.72102300
O	-2.76203300	0.63102000	-1.29632700
C	-2.75544200	0.98616200	-2.69525500
C	-4.21069000	0.95999300	-3.12118100
C	-4.91383100	1.41078700	-1.84526500
C	-4.10736300	0.69311500	-0.77954100
H	-3.47078600	-1.96374600	-1.67791500
H	-1.92904700	-2.85840600	-1.64835700
H	-2.34227400	-2.88147400	1.88583200
H	-3.39368900	-1.43992400	1.85044700
H	-2.87924100	-4.60930300	-0.24163100
H	-4.31665700	-4.19112500	-1.20388300
H	-4.48950800	-3.95990200	1.51012700
H	-5.07773900	-2.60091100	0.52258300
H	-0.65584100	-3.75542200	0.78379900
H	0.80084900	-3.18013900	-0.07905000
H	1.62975600	-1.27098400	2.62081600
H	-0.02197900	-1.13380300	3.30316200
H	2.24479300	-3.79474000	1.73926200
H	0.98570300	-5.02259600	2.01310300
H	1.54763800	-3.27275800	4.03159800
H	-0.16609300	-3.55624000	3.63081900
H	-2.32198100	1.99507800	-2.79638200
H	-2.10592200	0.27321700	-3.22071700
H	-4.46925000	-0.33750000	-0.62561900
H	-4.07217700	1.19819700	0.19528200
H	-4.51623400	-0.06499100	-3.38744400
H	-4.40364800	1.60781300	-3.98722700
H	-5.98009400	1.14856900	-1.80875500
H	-4.82776200	2.50295400	-1.72441400
H	1.56703100	2.23653400	-1.26859900
H	0.56122200	2.18336700	-2.74491600
H	-2.32805600	3.13694400	-0.72426600
H	-1.06837900	3.74751700	0.37998400
H	1.08704500	4.55461900	-2.74314500
H	1.20701400	4.59662100	-0.96679600
H	-1.36186400	4.32476300	-2.62796300
H	-1.04490600	5.49986400	-1.32562100
H	5.98996100	-2.42445900	0.62201500
H	4.34644700	-3.62552000	-0.90087400
H	6.74854400	-1.73622600	-0.83349000
H	3.32578100	-2.69036200	-2.02832400
C	5.96355300	-1.57320600	-0.07803500
C	2.59269600	2.02509100	2.24779400
C	4.41862400	1.04390900	-0.85735000
C	1.86010900	1.76182100	3.56454700
C	2.07997700	3.30241400	1.58706500
C	4.52242600	-0.22732800	-1.65771000
H	5.39634200	-0.21106300	-2.33238300
H	3.66734900	2.15114300	2.45921000
H	3.59883400	1.72089300	-1.10721900
H	2.17515300	4.16033300	2.27017100

H	0.78619000	1.59640000	3.38576900
H	2.26842700	0.87525900	4.07457200
H	1.01770700	3.19241100	1.32136800
H	2.65112500	3.53370100	0.67418800
H	1.96182900	2.62114900	4.24606700
H	3.62204500	-0.31220600	-2.28536900
C	5.56796100	1.70695500	-0.38032700

THF-based reaction **Int IV** with acrylonitrile

H_{corr} = 0.8230

$-TS$ = -0.1452

$E_{sol(THF)}$ = -26164.0861

$G_{sol(THF)}$ = -26163.4084

H	4.52949700	0.93059400	1.24699400
H	2.81703100	-1.35810400	0.17661100
H	4.03641600	-1.49184100	1.47182800
N	4.04039500	3.56596500	-1.03111200
H	6.47875700	-0.52611200	0.75382000
H	5.29905900	-2.58778800	-2.18354600
C	2.41784900	0.97336400	1.43594500
C	3.64845100	0.53504300	0.71514500
C	3.79005600	-0.98004100	0.52864100
C	4.86274400	-1.19974100	-0.55604400
C	4.53348100	-2.42224000	-1.40813500
O	1.24488500	0.64308400	0.89090500
Sm	-0.59275700	-0.00222300	0.08834800
O	-0.65980700	2.36481200	-0.62053900
C	0.49429300	2.98577700	-1.24153200
C	0.27119500	4.48239500	-1.11670200
C	-1.24967100	4.58768700	-1.05295100
C	-1.60715100	3.37246900	-0.22027200
I	0.67533100	-0.84924000	-2.60946800
I	-2.27617700	0.73770700	2.55162400
O	-2.17215000	-2.03363400	-0.18583300
C	-2.47249700	-2.70309200	-1.42629300
C	-3.14172900	-4.00568000	-1.02841000
C	-3.86408300	-3.60809600	0.25465600
C	-2.85481300	-2.68186400	0.90683800
O	0.25709400	-2.06114700	1.20119500
C	0.61219000	-3.30975400	0.57526400
C	1.60289500	-3.96904600	1.51949100
C	1.16237300	-3.42817000	2.87710100
C	0.80521500	-1.99499100	2.53730300
O	-2.70067400	0.52113400	-1.32857600
C	-2.67483000	1.06908500	-2.66369300
C	-4.11747700	1.05048400	-3.13458800
C	-4.87597900	1.25706300	-1.82758600
C	-4.06112700	0.41367300	-0.86598200
H	-3.14813900	-2.05888500	-2.01461300
H	-1.53622500	-2.81844300	-1.98852300
H	-2.10949900	-3.23798700	1.50047300
H	-3.28897000	-1.90096600	1.54569400
H	-2.38520300	-4.77938200	-0.81933500
H	-3.80899500	-4.38821100	-1.81294900
H	-4.12542200	-4.45939800	0.89804700
H	-4.79237100	-3.06226400	0.01987600
H	-0.30585200	-3.91088000	0.46312000
H	1.01627100	-3.09213500	-0.42392100
H	1.68771600	-1.33742300	2.51598700
H	0.04085700	-1.54409200	3.18516200
H	2.62660100	-3.63696800	1.29008700
H	1.57371500	-5.06516000	1.45045200
H	1.94363400	-3.48926100	3.64699900
H	0.27680500	-3.97279300	3.24312300
H	-2.27559900	2.09485200	-2.60746600
H	-1.98601500	0.46152400	-3.26613000
H	-4.36428500	-0.64667700	-0.90723500
H	-4.08865400	0.74442500	0.18151600
H	-4.36835900	0.07049000	-3.57209200
H	-4.31855700	1.82135200	-3.89108300
H	-5.92811700	0.94359800	-1.87048400
H	-4.84848900	2.31776800	-1.52919300
H	1.38778700	2.63635200	-0.71463600
H	0.53206600	2.64284600	-2.28732500
H	-2.61431100	2.97219900	-0.39749100
H	-1.49447400	3.55725400	0.86021900
H	0.72078300	5.03432900	-1.95224700
H	0.71973600	4.85588300	-0.18420200
H	-1.68870600	4.50715100	-2.06124300
H	-1.60595800	5.52214200	-0.59800300
H	6.28428700	-2.29551500	0.68374600
H	4.48865900	-3.33215700	-0.78610200
H	7.02966100	-1.42401900	-0.67881300
H	3.55966200	-2.30057100	-1.90796100
C	6.23923500	-1.36999400	0.08706700
C	2.42049300	1.95971000	2.57073200
C	3.72833700	0.98253800	-0.77246700
C	1.73241000	1.39072100	3.81542600
C	1.76844700	3.28695000	2.16795600
C	4.82684300	0.11030400	-1.39700900
H	5.79634700	0.62841500	-1.34678100
H	3.47790500	2.15818400	2.81860500
H	2.75642500	0.72472600	-1.22981100
H	1.76022600	3.99621800	3.01096400
H	0.68329500	1.13947900	3.59354200
H	2.23962100	0.47912500	4.16890900
H	0.72425600	3.10981000	1.86539400
H	2.30620800	3.75457600	1.32905500
H	1.73581900	2.12298400	4.63882800
H	4.61620600	-0.08576300	-2.45783600
C	3.91869800	2.41375100	-0.93344600

THF-based reaction **Product** with acrylonitrile

H_{corr} = 0.8241

$-TS$ = -0.1503

$E_{sol(THF)}$ = -26164.1031

$G_{sol(THF)}$ = -26163.4293

H	4.67395100	1.00240000	0.91829700
H	2.70835500	-1.29434900	0.44944500
H	4.00003300	-1.28057500	1.67914700
N	4.24286000	3.14928000	-1.80343500
H	6.45585900	-0.63230200	0.62520100
H	4.96851400	-3.21729900	-1.68001000
C	2.55165100	1.24754300	1.16835900
C	3.74010300	0.57653300	0.51970700
C	3.72992600	-0.94707800	0.66548300
C	4.71312800	-1.46892900	-0.40506100
C	4.25605900	-2.82392100	-0.93770600
O	1.42409100	1.00468300	0.76120000
Sm	-0.75786000	0.02034800	-0.04493400
O	-0.60129600	2.41481900	-1.05625700
C	0.41859000	2.91635400	-1.92274000
C	0.97508000	4.10969900	-1.17510000
C	-0.28905400	4.72328400	-0.56275000

C	-1.26292300	3.53883600	-0.45596400
I	0.70606700	-1.16387700	-2.65224700
I	-2.23716000	1.01627800	2.63579200
O	-2.27372600	-2.10635600	-0.02614600
C	-2.71862100	-2.76767200	-1.22393300
C	-3.62357100	-3.89507800	-0.75402600
C	-4.19961500	-3.32113600	0.53762500
C	-2.99489000	-2.60058400	1.11301000
O	0.41733000	-1.84354200	1.34604700
C	0.63206300	-3.18756500	0.87773100
C	1.30892300	-3.91449300	2.02717100
C	0.72725600	-3.19403100	3.24053400
C	0.69561900	-1.75782800	2.75277500
O	-2.91825300	0.47511900	-1.41928500
C	-2.91531200	0.98493200	-2.76588100
C	-4.36803800	1.30164100	-3.08825000
C	-4.93940400	1.61896300	-1.70889800
C	-4.22977000	0.59210100	-0.84835400
H	-3.26147500	-2.03052100	-1.83844000
H	-1.83231600	-3.09442400	-1.78634500
H	-2.34347200	-3.29779800	1.67237800
H	-3.23013000	-1.74582600	1.76350800
H	-3.03310700	-4.80030900	-0.53804300
H	-4.38549700	-4.15846800	-1.50064000
H	-4.60151200	-4.08270600	1.22010500
H	-5.00451100	-2.60160100	0.31614200
H	-0.34903500	-3.62565200	0.63284100
H	1.22455200	-3.13941200	-0.04687900
H	1.67828900	-1.27119300	2.88860800
H	-0.08034000	-1.12919600	3.21384400
H	2.40068000	-3.76950400	1.98452400
H	1.10856700	-4.99474900	2.01461000
H	1.32285300	-3.31559700	4.15583900
H	-0.29541500	-3.54874700	3.44752600
H	-2.28099200	1.88511800	-2.78255600
H	-2.45900700	0.22955700	-3.42215300
H	-4.73472200	-0.39049600	-0.90362200
H	-4.11484600	0.87328000	0.20875100
H	-4.87271400	0.41935600	-3.51399000
H	-4.46470200	2.12612400	-3.80807000
H	-6.03356400	1.53567000	-1.65338100
H	-4.65523800	2.63655200	-1.39516900
H	1.12618600	2.09976800	-2.11297100
H	-0.03179800	3.21428600	-2.88907500
H	-2.20377000	3.73330100	-1.00084900
H	-1.51367400	3.25303100	0.57630000
H	1.53281800	4.79774100	-1.82368900
H	1.66504600	3.76162100	-0.39317200
H	-0.70070100	5.50283700	-1.22082200
H	-0.09949000	5.18690900	0.41543000
H	6.15286600	-2.35354100	0.97405400
H	4.18491100	-3.56202200	-0.12152800
H	6.84768600	-1.86671400	-0.59078900
H	3.27075100	-2.73946400	-1.42234500
C	6.11870700	-1.58495500	0.18525800
C	2.79055600	2.20205500	2.31000600
C	3.72057900	0.70980900	-1.01541400
C	3.38625500	1.43237200	3.49668300
C	1.53494700	2.96288900	2.69888800
C	4.68764000	-0.37780100	-1.51317800
H	5.69203900	0.04206300	-1.67060200
H	3.56631400	2.90278400	1.94387000
H	2.70107200	0.45104500	-1.35899000
H	1.75210200	3.65708100	3.52374700
H	2.65024900	0.71895600	3.89885000
H	4.29729100	0.87743600	3.22699800
H	0.73229100	2.28032400	3.01670000
H	1.14447500	3.54631900	1.85360000
H	3.64679000	2.13451800	4.30172800
H	4.33567400	-0.77390700	-2.47535000
C	4.01943100	2.05851600	-1.47180600

HMPA-based reaction **Complex**

H_{corr} = 1.0848

$-TS$ = -0.1988

$E_{sol(THF)}$ = -27526.1940

$G_{sol(THF)}$ = -27525.3079

H	2.07465600	4.49258800	2.64221200
H	0.54026700	4.85313800	3.46596100
C	-1.00744600	3.32835000	1.87527300
C	-1.75811600	-4.62871600	-1.46805200
C	0.47110100	-4.54030600	-0.28053800
H	-1.51675100	-3.71399800	-2.03413500
H	-1.43211100	-5.49679800	-2.06097700
C	1.08036300	4.09312100	2.87589900
P	3.68698000	-1.40447100	-0.59445600
O	2.21703200	-1.02397400	-0.59080700
H	0.83545900	-5.40207300	-0.85994100
H	-1.46482200	3.01053100	0.93022100
Sm	0.01851400	-0.23640500	-0.00790200
N	3.87295900	-2.81553000	-1.49126200
C	3.02564200	-2.99781900	-2.65766200
H	2.76732100	5.61681800	-0.34592800
H	3.63652700	5.41428200	1.20341600
C	5.07808000	-3.61348200	-1.48059900
I	1.07214100	0.09821200	3.08550700
I	-0.61316500	-0.90979700	-3.15480300
N	4.58115000	-0.09048200	-1.17775700
C	3.88004500	0.89510100	-1.98688200
H	-3.31496400	-5.14555300	3.99957100
C	-1.03865200	-4.62132900	-0.11201600
C	5.94775300	-0.33736500	-1.58982200
N	0.52966100	4.58357900	-0.88058500
H	-2.85166200	-4.69020400	-1.35370000
C	0.76665100	4.35200600	-2.29106000
H	-0.99043400	2.45078000	2.54443300
H	-1.13968700	-3.69285900	4.00800600
N	4.43461900	-1.79387700	0.85659700
C	4.89976000	-0.73167700	1.74069700
H	2.92725100	1.71025600	1.16147200
H	4.42054000	2.38537700	0.44855700
C	3.82499800	-2.89959200	1.59182400
H	2.88275100	1.08354000	-1.57296100
H	4.45079000	1.83837100	-1.96533300
H	-4.73172900	-4.55940600	3.08886600
H	-3.42896500	-4.50527000	1.08886900
C	-3.86082000	-4.24253800	3.68258200
H	-1.30950700	-5.54384900	0.43269400
H	6.42669300	-1.05522400	-0.90957500
H	6.02024800	-0.72971800	-2.62304600
H	-0.04167200	5.96935900	0.56749600
H	0.82457100	6.68892100	-0.82705100
H	-3.10626200	-1.46489400	1.67488500
C	-1.75035600	-2.83779900	3.67552200

H	-1.10934000	-2.16093400	3.09740600
C	0.09770800	5.91197600	-0.51928300
H	1.61452500	4.95133900	-2.67435100
H	0.97185300	3.28676900	-2.45741900
H	5.30450400	0.09685100	1.14856200
H	4.08498200	-0.35332300	2.38350000
H	-4.68165700	-2.42895400	1.80110300
H	-2.07503800	-2.29203600	4.57574900
C	3.39916200	4.93262100	0.23431400
H	-4.23478100	-3.74321500	4.59130000
H	3.42082200	-3.63952200	0.88887200
H	4.59273800	-3.38841800	2.21339900
H	2.67639100	-4.04299200	-2.70438300
H	2.14419700	-2.34438500	-2.59897600
H	0.75537000	-3.62191900	-0.81311600
N	0.34082800	3.82571300	1.65566600
O	-0.95180300	-2.39480100	0.74237000
C	-1.56837700	-3.45770600	0.69679600
H	5.65963900	-3.40673200	-0.57343600
H	4.81474900	-4.68574100	-1.48631600
C	-2.84720600	-3.63590500	1.40360500
H	0.98251200	-4.53427400	0.69354100
O	-2.30305800	0.54029600	0.12213600
P	-3.60987100	1.00407500	-0.51087900
N	-3.46444600	1.81235400	-1.97103600
C	-2.30768900	2.67632600	-2.14586800
H	-6.34232600	0.71269100	0.03614600
H	-6.62537400	-0.11037700	-1.52653000
C	-3.98372500	1.26382000	-3.21228700
H	-1.95000200	3.04797300	-1.17751100
H	-1.47796300	2.13611900	-2.63320100
C	-6.08992300	-0.17384800	-0.55877100
H	-3.10646400	-1.43476600	-1.55543100
H	-4.41631300	-2.37133200	-0.77373900
C	-4.19240900	-1.48462900	-1.38952800
H	-3.23907300	0.62472400	-3.71856600
H	-4.89055100	0.67733500	-3.02029000
N	-4.32750900	2.12208900	0.51589000
C	-4.27758600	1.88292800	1.94368900
O	0.80155500	2.05113700	-0.33710600
P	1.09545000	3.44138500	0.21323600
C	-5.22907700	3.16063500	0.07869800
H	-5.24028300	1.50356800	2.33614900
H	-3.48658800	1.15576000	2.16598600
N	2.74785600	3.65611700	0.39314600
C	3.52733900	2.62333200	1.04973400
C	-2.96029900	-3.30806500	2.91058700
C	-3.59402800	-2.41528800	1.90409900
H	-5.17511200	3.25654400	-1.01289600
H	-6.27815400	2.95626800	0.36862400
N	-4.66178800	-0.28296700	-0.72325800
H	-1.63370400	4.12018800	2.32260600
H	1.19446300	3.17815900	3.48314500
H	3.57105800	-2.77649900	-3.59421100
H	5.71526800	-3.42034600	-2.36464300
H	3.77338600	0.58135900	-3.04259400
H	6.52158300	0.60296600	-1.54452300
H	-0.13121200	4.62198400	-2.87161300
H	5.70103900	-1.13445600	2.38173500
H	3.00834700	-2.54191700	2.24340100
H	-0.86456400	6.14317800	-1.00905100
H	4.34853600	4.80059000	-0.31304500
H	-2.59127300	3.54496400	-2.76468000
H	-4.25615100	2.09738100	-3.88123000
H	-6.47185700	-1.06093300	-0.02423000
H	-4.67322800	-1.61085000	-2.37639000
H	-4.03947800	2.82096000	2.47321000
H	-4.94044100	4.12625900	0.52978200
H	3.85401700	2.93701400	2.05798400

HMPA-based reaction **Int I**

H_{corr} = 1.0837

$-TS$ = -0.1958

$E_{sol(THF)}$ = -27526.1841

$G_{sol(THF)}$ = -27525.2962

H	0.58166500	4.51698000	3.14807400
H	-1.00455700	4.41844700	3.94348100
C	-2.20305600	2.91916700	2.08719900
C	0.16255800	-3.91975000	-2.64586000
C	1.89331300	-4.39415000	-0.88667800
H	0.36823400	-2.84011600	-2.71064300
H	0.81028300	-4.43768500	-3.37247400
C	-0.30859300	3.88868000	3.27239600
P	3.73220300	-0.25068900	-0.54078600
O	2.21038600	-0.13572700	-0.55649100
H	2.49315300	-4.92955500	-1.64258500
H	-2.64949900	2.78654600	1.09413900
Sm	-0.03034000	-0.24497300	-0.11517400
N	4.18302500	-1.48549700	-1.57858900
C	3.44641600	-1.62929600	-2.82528600
H	0.88546900	6.10835900	0.24511700
H	1.71505700	6.03126600	1.82750900
C	5.49611600	-2.09322800	-1.57228600
I	0.59421600	0.03084400	2.96944500
I	-0.37276100	0.25010400	-3.20949900
N	4.35116300	1.26785100	-0.94317200
C	3.49734000	2.16551000	-1.70970200
H	0.09012400	-6.64740300	2.70584600
C	0.39753200	-4.43714900	-1.22705300
C	5.75683000	1.33720000	-1.29279900
N	-0.92853700	4.42652500	-0.53399900
H	-0.88197800	-4.07095800	-2.95878800
C	-0.64113500	4.23116800	-1.94440200
H	-2.02055000	1.92108100	2.51856300
H	1.73779200	-4.67455300	2.35459900
N	4.48344000	-0.64925700	0.89835500
C	4.67758500	0.37887800	1.91431400
H	2.19450300	2.30301600	1.39734100
H	3.42559300	3.48263100	0.86263800
C	4.11937900	-1.94226500	1.47807700
H	2.45893700	2.08055600	-1.36951100
H	3.84045000	3.20077800	-1.54939300
H	-1.65740500	-6.30754400	2.74193600
H	-1.61406800	-5.28655000	0.59239100
C	-0.66657300	-5.89825100	2.99466600
H	0.06927400	-5.49119200	-1.17846300
H	6.34098300	0.65740600	-0.65691200
H	5.94584400	1.07835300	-2.35254500
H	-1.82088400	5.67254800	0.88418100
H	-1.21459900	6.53022400	-0.56932200
H	-1.39836100	-2.59372200	2.15135000
C	0.92551300	-3.96408400	2.58475800
H	1.07653700	-3.05503800	1.98905800
C	-1.69778400	5.60406200	-0.20364300

H	0.05744100	4.99620200	-2.33172000
H	-0.22186000	3.22935800	-2.11085200
H	4.89553400	1.34131500	1.43756400
H	3.78344300	0.48541100	2.55370600
H	-2.55824500	-3.97995800	2.52905800
H	1.01368200	-3.68229200	3.64692900
C	1.67450700	5.59569000	0.80986000
H	-0.62143400	-5.78569800	4.09183000
H	3.86814900	-2.66077100	0.68828200
H	4.97397400	-2.33412400	2.05267700
H	3.26989500	-2.69715500	-3.02937400
H	2.46980100	-1.13136100	-2.75637800
H	2.23763100	-3.34880200	-0.85150500
N	-0.96710100	3.68424800	1.99209000
O	-0.18551600	-2.36809200	-0.09739300
C	-0.41422400	-3.67579200	-0.20833400
H	5.99998000	-1.90051500	-0.61720700
H	5.39805600	-3.18478200	-1.70014500
C	-1.05177400	-4.39972200	0.91146500
H	2.09463100	-4.85013000	0.09396000
O	-2.29198000	0.03444500	0.19792800
P	-3.72082100	-0.25443700	-0.28376400
N	-4.10985600	0.71512800	-1.57753200
C	-3.49376700	2.02044800	-1.70797400
H	-5.15859300	-2.35697900	0.88707800
H	-5.20101600	-3.51175400	-0.46601800
C	-5.01917200	0.34936800	-2.64049700
H	-2.70013900	2.13888900	-0.96153600
H	-3.01698000	2.10742800	-2.69708000
C	-4.55627700	-2.81231700	0.09139600
H	-2.77224700	-1.51553000	-2.47467500
H	-2.43271900	-2.96735000	-1.50109900
C	-3.24012200	-2.33026100	-1.90362900
H	-5.45388700	-0.63690200	-2.44011700
H	-5.83695300	1.08793400	-2.72618300
N	-4.72169100	0.09294100	1.01639200
C	-4.28054300	-0.16304900	2.37429600
O	0.08327100	2.09864300	0.03208600
P	-0.07991300	3.51432300	0.58038600
C	-6.14115600	0.27509200	0.82832800
H	-4.74722700	-1.07164300	2.79931600
H	-3.19016300	-0.28436900	2.39287400
N	1.44341300	4.17140700	0.81896400
C	2.48434300	3.36136000	1.42367100
C	-0.41922800	-4.58072000	2.30453300
C	-1.59613700	-3.66789700	2.11275900
H	-6.34875700	0.57820800	-0.20664500
H	-6.72574900	-0.63917400	1.04910800
N	-4.06425800	-1.79679700	-0.82204400
H	-2.92118000	3.46711400	2.71967100
H	-0.01305000	2.93362000	3.74027000
H	4.00721500	-1.19797200	-3.67486100
H	6.13131900	-1.71026300	-2.39276000
H	3.52962900	1.95516000	-2.79487400
H	6.12783700	2.36083700	-1.12224200
H	-1.57765700	4.29966500	-2.52101000
H	5.53359500	0.09223800	2.54616800
H	3.24950400	-1.84197700	2.14912300
H	-2.69740000	5.54349000	-0.66826600
H	2.63708700	5.81034300	0.31529500
H	-4.23953500	2.82758900	-1.58487600
H	-4.48524800	0.31113900	-3.60598300
H	-3.73204500	-3.38535600	0.54819500
H	-3.86886100	-2.92621100	-2.58622600
H	-4.54705700	0.69100400	3.01977900
H	-6.50428300	1.07613500	1.49408600
H	2.66599600	3.64211500	2.47742800

HMPA-based reaction **TS I**
H_{corr} = 1.0814
$-TS$ = -0.1962
$E_{sol(THF)}$ = -27526.1695
$G_{sol(THF)}$ = -27525.2843

H	0.63489400	4.51157400	3.11509700
H	-0.94569200	4.48797900	3.92780700
C	-2.22184900	3.02578900	2.07986100
C	1.73358200	5.55593600	0.77724900
H	0.95006200	6.10366100	0.23836400
H	1.81059400	5.97316600	1.80038900
N	-0.96230100	3.75083700	1.97635500
C	-0.28245100	3.92578300	3.24992600
P	3.75186300	-0.28140800	-0.48908200
O	2.26105700	0.04687500	-0.56920000
H	-0.11698700	-6.52508700	3.07594200
H	-2.69199700	2.93603300	1.09250500
Sm	0.01358300	-0.21698900	-0.16052700
N	4.07312500	-1.58478600	-1.49282800
C	3.38454700	-1.63443400	-2.77448900
H	-2.63336000	-3.81742700	2.33112200
H	0.91551000	-3.48780300	3.80836200
C	5.31485000	-2.32850200	-1.44698200
I	0.54146200	0.06470000	2.94370700
I	-0.37712600	0.25260200	-3.24332400
N	4.57833100	1.13712500	-0.88632700
C	3.89490800	2.10800800	-1.72789500
O	-0.01092000	2.12225300	0.01003700
P	-0.09698100	3.54523700	0.55504200
C	6.00447800	1.03758500	-1.12821700
N	-0.92010400	4.49121700	-0.55044200
O	-0.03777600	-2.34940100	-0.15092500
C	-0.65268900	4.28412000	-1.96300300
H	-2.06817200	2.01218400	2.48624200
C	0.36526200	-4.45138300	-1.28059800
N	4.39767200	-0.74094700	0.98063500
C	4.64302300	0.27514900	1.99815300
C	-3.30361100	-2.32064200	-1.96697500
C	-0.54272700	-4.47054900	2.55016700
C	3.91681700	-1.99824400	1.55429300
H	4.00831600	1.88394200	-2.80522400
H	2.82602800	2.12541400	-1.48603900
N	1.45538500	4.13961000	0.76903400
C	2.48122400	3.28841600	1.34280200
H	2.17727800	2.23686300	1.26868100
H	3.42459200	3.42180600	0.78850300
H	6.47953300	2.01365500	-0.93724700
H	6.45460400	0.30387100	-0.44551700
H	-1.75517900	5.76877700	0.87433400
H	-1.12930700	6.60371100	-0.58415500
H	-1.83239100	-6.19467300	2.72230400
H	-1.52604000	-5.26396300	0.52560500
C	-0.91869200	-5.77329800	3.17313100
C	-1.64322200	5.69577500	-0.21443300
H	0.05639900	5.03380500	-2.36088800
H	-0.25234500	3.27449500	-2.12900100

H	4.94219500	1.21723200	1.52424500
H	3.74280100	0.44792400	2.61370200
C	-1.60970900	-3.59755200	1.99687000
C	-4.63535900	-2.82838800	0.01222200
H	-2.81189900	-1.50184600	-2.51180900
H	-2.51805700	-2.99774800	-1.59010900
H	3.60395800	-2.69002900	0.76313900
H	4.72993700	-2.46663200	2.13217500
H	3.12324600	-2.67769100	-3.01001000
H	2.45338000	-1.05318700	-2.73695900
H	-1.12301100	-5.66244300	4.25876400
H	2.28227900	-3.50319700	-0.91597200
H	-5.15838000	-2.37837800	0.86482800
H	-5.35994700	-3.44122000	-0.54980000
H	5.80593000	-2.19352600	-0.47562600
H	5.10338100	-3.40249900	-1.58375100
H	-0.04037800	-5.47729300	-1.23592800
C	-0.35904600	-3.64202100	-0.23125500
O	-2.25011300	-0.06890500	0.17095100
P	-3.69968000	-0.28758700	-0.27933100
N	-4.08921600	0.73418600	-1.53283300
C	-3.44623500	2.02940800	-1.62883300
C	0.15575400	-3.90101800	-2.68939900
C	1.85825900	-4.51921300	-0.93993100
C	-5.03305300	0.43012300	-2.58475000
H	-4.16832900	2.84828900	-1.45221100
H	-2.62849900	2.09962500	-0.90269600
H	0.43135600	-2.83702300	-2.74371600
H	0.76206400	-4.45341300	-3.42611700
H	-0.89804400	-3.97828600	-2.99798000
H	2.01521100	-4.97566200	0.04950200
H	-5.48533700	-0.55325400	-2.41069700
H	-5.83568200	1.18920800	-2.62178800
N	-4.65491900	0.04966900	1.05586600
C	-4.17782900	-0.23475100	2.39610100
H	2.41555300	-5.10989400	-1.68681800
H	1.59401300	-4.70239800	2.69438200
C	-6.07324100	0.27624800	0.91308900
H	-4.63192000	-1.15261200	2.81494400
H	-3.08684900	-0.35273500	2.38434500
H	-1.39862800	-2.52221300	2.03124900
C	0.82081200	-3.92028000	2.79291400
H	1.04754300	-3.11195400	2.08453100
C	-1.15471400	-4.25865000	0.73759700
H	-6.30299900	0.59802300	-0.11160700
H	-6.67780600	-0.62309900	1.14064900
N	-4.10167600	-1.79979300	-0.86090000
H	-2.90787800	3.58414800	2.73800400
H	2.69193000	5.74639000	0.26500000
H	-0.02480000	2.95872800	3.71534800
H	4.02009600	-1.24054800	-3.58930000
H	6.01343600	-2.01364800	-2.24451000
H	4.32267400	3.10667600	-1.53968400
H	6.23985600	0.74250700	-2.16900200
H	-1.59380600	4.36805600	-2.53037700
H	5.45973800	-0.06966000	2.65261100
H	-3.95426000	-2.87163700	-2.66656800
H	3.05678100	-1.81984400	2.22114600
H	2.66558900	3.52042100	2.40789700
H	-2.64807000	5.67247500	-0.67120500
H	-3.83833300	-3.49075700	0.38959600
H	-2.99643300	2.14607400	-2.62738900
H	-4.52461000	0.41724500	-3.56459500
H	-4.42931600	0.60589200	3.06459400
H	-6.39254100	1.07920100	1.59881600

HMPA-based reaction **Int II**

H_{corr} = 1.0829

$-TS$ = -0.1982

$E_{sol(THF)}$ = -27526.1894

$G_{sol(THF)}$ = -27525.3047

H	0.84864800	4.64909500	2.91650300
H	-0.79848100	4.73933100	3.57953800
C	-1.90844800	2.98109500	1.87654000
O	0.34187200	2.00212600	-0.10180000
P	0.30225700	3.43392500	0.42703300
N	1.86061200	3.95119200	0.75477000
C	2.74155200	3.09576200	1.52725800
C	-0.07806700	4.08152800	3.06458200
P	3.78440300	-0.66406900	-0.34129600
O	2.29085300	-0.38997800	-0.49729400
H	-3.81638800	-2.71222600	3.94668500
H	-2.12239300	2.44938400	0.94154600
Sm	0.02642100	-0.32314200	-0.17638300
N	4.18555400	-1.96513500	-1.31678000
C	3.52273700	-2.08576300	-2.60636400
H	-0.53650900	-4.33181800	4.65911200
H	-2.10478100	-2.25081800	1.51308000
C	5.42426000	-2.70267100	-1.19443400
I	0.45173200	0.02524200	2.93541300
I	-0.17652500	0.00501700	-3.29818900
N	4.59487500	0.77082200	-0.71173200
C	3.90129800	1.74545700	-1.54240900
C	-1.92669700	-3.48749900	3.24898200
C	-2.27609200	-3.30995000	1.79871400
C	6.02107100	0.68053600	-0.95733800
N	-0.37501200	4.41320200	-0.74696400
C	-3.30576500	-2.31222100	-1.80838000
C	-0.03676300	4.16999700	-2.13752400
H	-1.87241000	2.21829700	2.67101700
H	-1.72494400	-5.25430500	0.84936300
N	4.36471400	-1.10321000	1.16315800
C	4.58864100	-0.07718500	2.17523500
C	-0.02233300	-4.25412000	-2.39678500
C	1.67000500	-4.62616000	-0.57086100
C	3.81214900	-2.33348000	1.73123500
H	2.84143000	1.78768800	-1.26636200
H	4.35306600	2.73677800	-1.37287900
H	-3.36981500	-3.46568700	1.69443900
H	0.12924700	-3.01567000	3.66430900
H	-2.41440900	-1.67391800	4.30381800
H	2.07294900	-3.60723600	-0.67042100
H	6.47925300	-0.04615200	-0.27204900
H	6.25703400	0.38186700	-1.99701200
H	-1.26035700	5.72687700	0.61238900
H	-0.45676200	6.53776800	-0.76865700
C	-2.75070000	-2.73097800	4.23336400
H	-0.20348600	-5.66091600	-0.77547300
H	-5.84467100	-1.48798200	0.28922200
C	-1.05239700	5.65627800	-0.46279500
H	0.74117500	4.86691800	-2.50228700
H	0.30743400	3.13468900	-2.26424700
H	4.94871700	0.84387600	1.70316700
H	3.66364200	0.14073300	2.73748500

H	0.27266000	-3.20802300	-2.57302600
H	0.57236900	-4.89860400	-3.06447900
H	-1.07967000	-4.35276100	-2.68787300
H	1.82516900	-4.95333400	0.46929900
H	3.60047700	-3.05946800	0.93758400
H	4.55001400	-2.77110000	2.42218900
H	3.25607100	-3.13928800	-2.78776600
H	2.59721500	-1.49467900	-2.62087200
C	-0.55136300	-3.89306700	3.64701600
H	-0.12230100	-4.61556700	2.93645300
H	2.24741900	-5.29300000	-1.23229000
C	-1.53203400	-4.17774600	0.83479400
H	5.87392100	-2.52954000	-0.20895000
H	5.22309600	-3.78250800	-1.29967100
H	-5.99055500	-2.54177300	-1.14144700
H	-2.67507900	-3.15300200	5.24926900
O	-2.22892500	0.10465200	-0.06560400
P	-3.68170400	0.04567800	-0.56334100
N	-3.96103200	1.04048000	-1.88047800
C	-3.23440700	2.30093100	-1.91206400
C	0.18597300	-4.64121200	-0.93426300
H	1.61421400	5.84616200	-0.10338300
C	-4.30342300	0.51933200	-3.19241100
H	-3.07990900	2.67768800	-0.89309200
H	-2.25115600	2.18567700	-2.39891500
H	2.35075500	5.87461000	1.52637200
N	-0.63195400	3.67049900	1.78980800
O	-0.24719100	-2.43697900	-0.09600800
C	-0.58207000	-3.71750200	-0.01151100
H	-3.40392000	0.33133600	-3.80449500
H	-4.86904300	-0.41387300	-3.08709000
N	-4.64233500	0.64918200	0.66531200
C	-4.32575800	0.30586900	2.04031200
C	2.29520600	5.31403500	0.57262600
H	2.29406800	2.09826400	1.63960900
C	-5.95728000	1.20058600	0.43259900
H	-3.28153300	-0.02380300	2.11235500
H	-4.45284900	1.19410000	2.68120000
H	3.71148000	2.98854900	1.01402600
C	-5.29250100	-2.16515600	-0.37300300
H	-2.48212500	-1.71619900	-2.22733700
H	-2.86842700	-3.12570300	-1.20536800
H	-6.07101700	1.44167900	-0.63191800
H	-6.76521000	0.50532000	0.73106600
N	-4.17979700	-1.48130800	-0.99158500
H	-2.72467200	3.69533900	2.08592200
H	2.92287400	3.50475400	2.53816600
H	0.13349200	3.20982200	3.70847000
H	4.17817300	-1.74457200	-3.42865500
H	6.15396300	-2.41498500	-1.97413200
H	3.97689900	1.51271300	-2.62104400
H	-4.94183700	-3.02428900	0.22448800
H	6.48909900	1.66067600	-0.77094000
H	-3.87515000	-2.74427300	-2.64951100
H	-0.93444900	4.30274100	-2.76283900
H	5.35455200	-0.43984300	2.87946200
H	2.87659700	-2.13206000	2.28137100
H	-2.01146200	5.69590200	-1.00796100
H	-3.83167900	3.04621000	-2.46277000
H	-4.94029000	1.25399400	-3.71264500
H	-4.98059400	-0.49565300	2.42935000
H	3.30104400	5.32622200	0.11852500
H	-6.08125800	2.12877500	1.01637500

HMPA-based reaction **PreTS II** with phenylacetylene
H_{corr} = 1.2042
$–TS$ = -0.2182
$E_{sol(THF)}$ = -27834.4474
$G_{sol(THF)}$ = -27833.4614

H	1.43039900	5.03056400	0.28376700
H	3.11389100	4.47247900	0.12069600
C	2.73274800	1.96928000	0.89923000
H	2.67529800	-5.34041900	-1.80826000
H	0.05288200	-4.37522100	-3.51222600
C	3.21654600	-4.37932800	-1.94439700
H	-2.01500100	-4.86928400	-2.71704300
C	2.05958600	4.15908600	0.06394700
P	-4.02226500	0.97738200	-1.03160000
O	-2.77254500	0.34149500	-0.42929500
H	-2.56730900	-3.75933200	0.10086500
H	2.42701400	1.15166700	1.56372200
Sm	-0.68903300	-0.47211200	0.05818100
N	-5.20958700	-0.19879400	-1.14801000
C	-5.30879400	-1.18548500	-0.08409200
H	3.36819100	2.56638500	-3.43865700
C	4.21307200	2.80584900	-2.79219500
C	-6.39689900	-0.05589600	-1.96176000
I	0.42540600	1.27313300	-2.32469600
I	-2.14179000	-1.55692000	2.62805900
N	-4.43682000	2.29934500	-0.06540800
C	-3.95990900	2.30906300	1.31108000
C	4.65134600	0.40401800	-2.57280200
H	4.02115800	-1.74483000	-3.07002800
C	-5.76681300	2.85036700	-0.23968900
N	0.54654200	2.78092300	3.33789500
C	-2.48817300	-4.57425300	-0.63589900
C	-0.53653600	2.27287700	4.16014600
H	2.71328300	1.58338200	-0.13363200
H	-3.44597700	-5.11902000	-0.64989200
N	-3.93328200	1.59424100	-2.58253700
C	-3.36141000	2.91621700	-2.80737900
H	-3.38961100	-2.24653500	-1.91166700
H	1.55882200	-2.17786500	-1.94064400
C	-3.55919600	0.63862300	-3.62540600
H	-2.93472100	1.92492200	1.35818700
H	-3.96444200	3.34918300	1.67648800
H	6.63189400	1.23900000	-0.97183000
H	7.17608400	3.60055500	-0.40901000
H	5.83550100	5.45457500	-1.39205200
H	3.93348700	4.93325700	-2.91399700
H	-6.04609800	2.84339300	-1.30256000
H	-6.53679000	2.29551600	0.33050300
H	2.32639400	3.86857900	3.30616600
H	1.18133100	4.38044400	4.58731400
H	-1.70971800	-5.27008100	-0.28561000
H	-3.06910100	-2.80167000	-3.57268100
H	-4.24216100	-3.67919600	-2.55036900
C	1.58251200	3.51227700	4.02961900
H	-1.17193200	3.08614600	4.55927900
H	-1.15471200	1.57661700	3.57701200
H	-3.62402100	3.58457900	-1.97968700
H	-2.26210600	2.86784000	-2.89923800
C	4.34646300	-0.73818300	-2.85051700
C	4.52890300	4.12472400	-2.48488700

H	2.80304000	-3.28356500	-5.22184700
H	3.11114500	-3.82002700	-1.00178900
H	-3.94642500	-0.35869500	-3.38408200
H	-3.99446400	0.96402600	-4.58377700
H	-5.48281800	-2.18292000	-0.51918600
H	-4.37631400	-1.22464700	0.49529600
C	4.97311300	1.75605100	-2.25056400
C	6.04039100	2.05612800	-1.38816700
C	6.34249000	3.37831400	-1.07839300
C	5.59119800	4.41692800	-1.62890800
H	-6.24742800	0.72520700	-2.71725000
H	-6.60625200	-1.00664500	-2.48144700
H	4.02385800	-4.41890700	-4.57139100
C	-3.28102900	-3.13894100	-2.54601800
O	1.29180500	-1.22715800	0.97952200
P	2.17131100	-2.07244200	1.90897200
N	1.80906900	-1.97829600	3.53953000
C	1.53171100	-0.65592900	4.07923300
H	-0.12741100	6.14879100	1.59395400
N	1.83486600	3.10796700	1.04182500
C	1.12148400	-3.05436300	4.23458300
H	2.06070400	0.11298500	3.50051300
H	0.45143400	-0.43784500	4.04434200
O	-0.83239400	-2.23004500	-1.13839200
C	-0.85486000	-3.24182400	-1.99847700
H	4.27905500	-4.63944400	-2.08929100
C	-2.15168300	-4.02497400	-2.02039000
H	0.02799000	-2.90946900	4.22454500
H	1.35250200	-4.01881900	3.76779600
N	3.73342800	-1.48462600	1.81805200
C	4.25670900	-1.15612300	0.50486900
H	2.33998000	-4.93364700	-4.76981200
C	0.17770400	-3.54035400	-2.81667200
C	4.71034800	-1.58769100	2.87665200
H	3.43381700	-1.02506500	-0.20656300
H	4.81550700	-0.20690800	0.54796100
C	2.67050800	-3.60534800	-3.09493600
H	-2.36976000	4.39155900	0.07952000
C	1.45930200	-2.74948000	-2.87847100
H	-0.30897500	5.16410500	3.07542300
H	4.21871100	-1.89289500	3.80843900
H	5.50374500	-2.32022300	2.63430500
N	2.13228500	-3.66962500	1.42065800
C	0.88804400	-4.27426200	0.97012600
N	-0.68403000	4.11320700	1.30159000
C	-1.29270000	4.17500300	-0.01389700
C	3.17419300	-4.59442200	1.80746000
H	0.18185300	-3.49942500	0.65115500
H	1.09106300	-4.91220400	0.09628700
H	1.39613600	-1.99292100	-3.68409000
C	2.98071200	-4.07460800	-4.47546100
C	-0.70971200	5.33458200	2.06828400
H	-1.17323500	3.21212600	-0.52936900
H	4.09957000	-4.05421000	2.04535500
H	2.89360500	-5.21043000	2.68329300
O	-0.35799900	1.52547200	1.24556200
P	0.31773300	2.82570700	1.68080400
H	3.76268900	2.27384200	1.14974100
H	1.85638900	3.80937700	-0.96284800
H	-6.14391700	-0.95344900	0.60218000
H	-7.28478100	0.20110400	-1.35424000
H	-4.59404800	1.70238400	1.98377600
H	-5.77825200	3.89724500	0.10453800
H	-0.11895000	1.71687900	5.01457400
H	-3.77940400	3.32509600	-3.74148200
H	-2.46211400	0.57398100	-3.72708500
H	2.08679900	2.85269900	4.75710700
H	1.88324600	-0.60387200	5.12366400
H	1.47473600	-3.08753800	5.27894400
H	4.93577800	-1.93957200	0.12262800
H	5.19731400	-0.61001400	3.03784800
H	-1.75081300	5.68532700	2.17259700
H	0.41623300	-4.88477700	1.76081700
H	-0.82907500	4.95813100	-0.64129800
H	3.38582500	-5.27646400	0.96775700

HMPA-based reaction **TS II** with phenylacetylene

H_{corr} = 1.2030

$-TS$ = -0.2138

$E_{sol(THF)}$ = -27834.4432

$G_{sol(THF)}$ = -27833.4540

H	1.67090200	5.05407000	-1.56613100
C	1.88752600	3.15825100	0.27850700
H	3.70551100	-5.36216700	-2.54306500
H	1.74666800	-5.25068400	-0.76214600
C	4.37369400	-4.53488100	-2.22721500
H	-0.43610900	-5.65890500	-0.69392000
C	0.77992400	4.40964900	-1.49334400
P	-3.96820800	-0.55925800	-1.43418900
O	-2.66450200	-0.57383000	-0.63867800
H	-1.82834500	-3.73712200	1.26542900
H	2.75739100	3.83444300	0.24897300
Sm	-0.53689300	-0.44237900	0.23247600
N	-4.76401900	-2.00869900	-1.16833300
C	-4.68958000	-2.60755600	0.15427300
H	5.59794100	-2.72354500	-0.41665500
H	3.25376100	0.64873800	-2.05990300
C	-5.84360700	-2.49505800	-1.99867600
I	0.27433000	0.61337700	-2.62392800
I	-2.03093000	-0.94409100	2.95882500
N	-4.84135100	0.81154100	-0.97477500
C	-4.54619400	1.40669700	0.32142100
H	7.51891500	0.52810400	-2.70625800
C	6.56399400	1.04705600	-2.80337200
C	-6.24472200	0.84683600	-1.33917500
N	-0.78866500	3.97787000	2.04516000
C	-1.49827900	-4.73317000	0.93184200
C	-1.82677600	3.49377100	2.93847100
H	1.77357200	2.78111300	1.30235700
H	-2.37908500	-5.39546100	0.90221900
N	-3.89207500	-0.46425100	-3.10193100
C	-3.71059200	0.82319800	-3.75886500
H	-2.18721400	-3.21315300	-1.29879700
H	4.96756200	-4.91747000	-1.37930100
C	-3.18136200	-1.54523700	-3.78207200
H	-5.04887800	0.88182300	1.15447400
H	-3.46628900	1.39654000	0.50483300
C	5.40235600	0.44404200	-2.28494800
C	4.17255500	1.11622100	-2.41783100
C	4.11318600	2.35578500	-3.04270100
C	5.27074700	2.94943900	-3.54694800
H	-6.39014100	0.41909900	-2.34092300
H	-6.88502200	0.29508100	-0.62415400
H	0.44075500	5.01051400	3.39225900

H	0.59382200	5.49867500	1.67908700
H	-0.80541200	-5.12381700	1.69331200
H	-1.31985400	-4.19209500	-2.50521900
H	-2.66455200	-4.90984600	-1.57325700
C	-0.11578100	5.18927000	2.45606400
H	-2.71659800	4.15117800	2.93417800
H	-2.12135200	2.47412200	2.65460100
H	-4.22897900	1.60782600	-3.19691300
H	-2.64144000	1.08518500	-3.84365700
C	5.38229300	-1.92281900	-1.10235300
C	6.49361900	2.28696400	-3.42802500
H	3.55887100	-3.72570200	0.23554500
H	2.52887100	-1.59791300	-2.60975700
H	-3.30833400	-2.48730100	-3.23570800
H	-3.59786500	-1.66657900	-4.79490600
H	-4.49262700	-3.68810300	0.06420300
H	-3.87338100	-2.15888700	0.73580900
C	5.46580100	-0.81279000	-1.62306000
H	3.14306200	2.84566300	-3.14864800
H	5.22036200	3.92156400	-4.04170400
H	7.40285000	2.74196400	-3.82674900
H	-6.82884300	-2.35364000	-1.51647300
H	-5.84540300	-1.97497000	-2.96436800
H	3.73528100	-2.28322600	-3.73758700
C	-1.80556800	-4.22075600	-1.51735600
O	1.40744700	-0.00668600	1.40210900
P	2.26779200	-0.24282400	2.65515500
N	1.50872200	0.26321600	4.04639300
C	0.72324300	1.47944500	4.02665200
H	-1.98618500	6.11358200	-0.97455200
N	0.69802700	3.89060300	-0.13658600
C	1.42464400	-0.50889600	5.26619600
H	0.67379100	1.88616300	3.00946500
H	-0.31097000	1.25840000	4.33408300
O	0.07073200	-2.44833000	-0.15661100
C	0.36700700	-3.71898100	-0.39147700
H	5.05700500	-4.33548700	-3.06769700
C	-0.82164500	-4.65836800	-0.43564500
H	2.09014400	-1.37828400	5.21010200
H	1.73157200	0.10823000	6.12951300
N	3.64963400	0.68540100	2.48948600
C	4.31220900	0.77441500	1.20142900
N	-2.01356700	4.06365000	-0.39105800
C	-2.47558500	3.42985700	-1.61094600
C	4.41471100	1.13847400	3.62717600
H	5.24512200	0.18510700	1.17071900
H	3.64587300	0.40010600	0.41662200
H	2.59201100	-2.30911400	-0.24619700
C	2.98722700	-2.53062100	-2.96754800
C	-2.40966600	5.43895800	-0.20518500
H	-2.08580200	2.40546200	-1.66941400
H	3.80304400	1.08033700	4.53690200
H	5.33573700	0.54411100	3.78140900
N	2.67999000	-1.83476600	2.89998300
C	1.63527200	-2.84874300	2.90831200
H	-3.57728500	3.38906700	-1.61595600
H	2.17804800	-3.10315100	-3.46227300
C	4.03978100	-2.31689700	2.95450100
H	0.64200300	-2.37620400	2.92140500
H	1.69524300	-3.49268000	2.01554200
C	1.62714200	-4.18204300	-0.56302500
C	3.58940000	-3.31894900	-1.85749900
C	2.86677400	-3.33512800	-0.53821400
H	-2.09456000	5.80013000	0.78188300
H	4.74869500	-1.48475000	2.87073500
H	4.23094900	-2.84361100	3.90713700
O	-0.98736100	1.85775600	0.54351700
P	-0.76819800	3.36944600	0.48631700
H	2.08023900	2.29741500	-0.38242400
H	0.85864000	3.60018500	-2.24016100
H	-5.63556600	-2.46928000	0.70893100
H	-5.71106000	-3.57468200	-2.18597200
H	-4.89070800	2.45388500	0.31243600
H	-6.58986900	1.89311500	-1.36633400
H	-1.43504000	3.45646600	3.96699000
H	-4.14348200	0.76535100	-4.77074900
H	-2.10262000	-1.32503000	-3.85740100
H	-0.82689500	6.01700900	2.64111600
H	1.15604700	2.24170000	4.70140800
H	0.39130600	-0.86053300	5.43065300
H	4.55961200	1.82477900	0.97454500
H	-2.13903300	3.97696900	-2.51058300
H	4.71572000	2.18956300	3.47862800
H	-3.50880600	5.51757200	-0.25950200
H	1.72466200	-3.47355500	3.81421300
H	4.23838700	-3.01952300	2.12764200
H	-0.09981700	5.02306300	-1.72347400

HMPA-based reaction **Int III** with phenylacetylene

H_{corr} = 1.2057

$-TS$ = -0.2125

$E_{sol(THF)}$ = -27834.4960

$G_{sol(THF)}$ = -27833.5028

H	0.45088400	4.92448200	-1.72533700
H	-1.30113600	5.04845800	-2.00492000
C	-1.75257900	2.39313800	-2.14598300
H	-4.16862800	-3.10856500	2.28376600
H	-2.12277800	-2.48472600	4.27323700
C	-4.64320500	-2.15889100	1.99121800
H	-0.37989500	-3.88305700	4.13705700
C	-0.50703600	4.41040500	-1.58176600
P	3.91494500	0.05277600	1.48146300
O	2.61386800	-0.32854700	0.78031500
H	0.89592200	-4.09057500	1.34328100
H	-1.65704500	1.39066000	-2.58069600
Sm	0.45895100	-0.54354900	0.03437200
N	4.56093700	-1.32866300	2.17520200
C	4.41335600	-2.59221700	1.47079100
H	-6.10443000	0.12095100	2.69050000
H	-4.38044100	2.51386100	-0.11487200
C	5.60336600	-1.30584600	3.17753200
I	-0.19265400	2.09246000	1.59936400
I	1.72429000	-2.81866500	-1.73604000
N	4.90957700	0.79569700	0.33604000
C	4.65193500	0.52110800	-1.07092400
H	1.98313800	-2.31112400	2.97078100
H	-4.30383000	-1.91170000	0.97435200
C	6.31404500	0.92881100	0.67063800
N	0.84224500	1.79677800	-4.11441700
C	0.41589700	-4.51572800	2.23880400
C	1.77769400	0.77889800	-4.55800400
H	-2.01931900	2.27394200	-1.08292700
H	1.07870600	-5.29767000	2.64321800
N	3.85883400	1.13442700	2.75339700

C	3.79946100	2.56768300	2.49516200
H	-3.76857700	3.20015900	4.11078200
C	-3.75407500	3.56837700	3.08444100
C	3.04759100	0.73661500	3.90327400
H	3.57290500	0.49745700	-1.26055500
H	5.09799400	1.33015900	-1.67242900
C	-5.00509900	0.21528200	2.62320900
C	-3.35378100	4.86092300	2.80281700
H	-4.39148200	-0.69542200	5.12535200
H	-2.45079700	-0.49613100	1.91062600
H	6.43032700	1.17312700	1.73574100
H	6.89337600	0.01027000	0.45483500
H	-0.42972000	3.34146800	-4.70720200
H	0.95308400	3.06857100	-5.81447100
H	-0.52127200	-4.99982600	1.92266600
H	1.27817900	-2.10595900	4.59267700
H	2.13581900	-3.61749100	4.17771900
C	0.21251200	2.57408100	-5.15682300
H	2.69423500	1.21949000	-4.99384800
H	2.04831200	0.12814400	-3.71560000
H	4.38162000	2.81133900	1.59931600
H	2.75809200	2.90619900	2.35372700
C	-4.13195400	2.67766600	2.03497600
C	-4.08908600	3.17546300	0.69948700
C	-3.69500700	4.47546600	0.44129800
C	-3.31837200	5.33034000	1.48349700
H	3.05788000	-0.35412500	4.01638800
H	3.46839600	1.19031400	4.81508600
H	4.13907000	-3.38536600	2.18529400
H	3.61859000	-2.52307800	0.71583600
H	-3.67629400	4.83479100	-0.59029600
H	-2.99877900	6.35183500	1.27079300
C	-4.50345300	1.38108500	2.31589600
H	-3.05602600	5.51954600	3.62136800
H	5.66316900	-0.31349700	3.64117100
H	5.37635200	-2.04411300	3.96583700
H	-5.79822800	-1.54663000	4.43723100
C	1.46458300	-2.83428100	3.78814500
O	-1.51965200	-0.88021400	-1.13212200
P	-2.46943600	-1.72586300	-1.98811400
N	-1.82809500	-2.26808700	-3.42989400
C	-1.00287200	-1.35666600	-4.20374600
H	2.33452900	5.21968500	-3.03628100
N	-0.50807900	3.13598200	-2.28103300
C	-1.58238100	-3.67313700	-3.69879100
H	-1.13235400	-0.32413000	-3.85431100
H	0.06229600	-1.61687600	-4.08901600
O	-0.26397600	-1.74803000	1.63214200
C	-0.73102500	-2.33058300	2.73034600
H	-5.73538300	-2.30942900	1.97347600
C	0.15192100	-3.43262900	3.28197000
H	-0.52940600	-3.93606000	-3.50086800
H	-2.22398300	-4.30108800	-3.07009300
N	-3.75300100	-0.75072800	-2.44545600
C	-4.38546200	0.05850100	-1.42226900
N	2.21673200	3.24345300	-2.24615600
C	2.71146400	3.44740600	-0.89737600
C	-4.50274200	-0.91283600	-3.66895600
H	-3.70331800	0.18962500	-0.57289600
H	-4.62441100	1.05382900	-1.83331900
C	-4.70117900	-1.45228100	4.38809500
H	-4.27091100	-2.42061100	4.68401800

C	2.67777900	4.18601700	-3.23682600
H	2.27278300	2.70276000	-0.22064600
H	-3.96149600	-1.58162600	-4.34935200
H	-5.51080000	-1.33197300	-3.48575300
N	-3.05413400	-3.01838100	-1.11002500
C	-2.16901800	-3.78887700	-0.25142600
H	3.80856200	3.33799000	-0.88162700
C	-1.87325100	-1.95683000	3.34878900
C	-4.31333800	-3.64931400	-1.43528400
H	-1.28722800	-3.19740600	0.01842700
H	-2.69754700	-4.03207500	0.68272400
H	-2.52013700	0.07472900	3.56505000
C	-4.24730000	-1.05790300	2.97703300
C	-2.73089800	-0.80615600	2.92939600
H	2.33051900	3.89454800	-4.23609400
H	-4.94595700	-2.96995000	-2.02057300
H	-4.18211500	-4.58614900	-2.00998300
O	1.04618400	0.99132400	-1.65203900
P	0.89257400	2.25177300	-2.50294300
H	-2.56452500	2.92936600	-2.66733900
H	-0.68784900	4.27789100	-0.50141300
H	5.35448600	-2.88415900	0.96966500
H	6.59154700	-1.55945500	2.75017600
H	5.08812000	-0.43902400	-1.40347300
H	6.75435800	1.75300200	0.08626200
H	1.30056000	0.15363000	-5.32933200
H	4.23413700	3.09864200	3.35767500
H	2.00166700	1.06641700	3.78101600
H	-0.41109200	1.91942400	-5.79020400
H	-1.28227400	-1.40416400	-5.27112900
H	-1.82020900	-3.88976900	-4.75432600
H	-5.32389400	-0.39478200	-1.05334400
H	2.45229100	4.45087200	-0.51305700
H	-4.63315200	0.06472900	-4.16502700
H	3.78095500	4.19655300	-3.25200800
H	-1.83748600	-4.72557800	-0.73404600
H	-4.84900200	-3.89510000	-0.50356400

HMPA-based reaction **TS III** with phenylacetylene

H_{corr} = 1.2051

$-TS$ = -0.2104

$E_{sol(THF)}$ = -27834.4859

$G_{sol(THF)}$ = -27833.4912

H	-4.86273700	-2.21314400	2.78713100
H	-3.93668000	-3.57351000	3.45735600
C	-2.00328500	-3.59272400	1.62974800
C	4.09614300	-2.88832200	-0.24815700
P	-3.59790600	-1.93739200	0.26512200
H	-2.10438400	-4.56735200	2.13571900
H	2.17981300	-1.06384800	-2.37556300
C	-3.85556600	-2.63621300	2.88323100
P	-2.44934200	3.30961700	-0.23774500
O	-1.59583000	2.04526100	-0.31886600
H	3.31319400	-0.44439100	-1.15041100
H	-1.61031400	-3.76550100	0.62048000
Sm	-0.38348900	0.11112100	-0.06326900
N	-1.69494300	4.51422700	-1.12343900
C	-1.02701200	4.14326200	-2.36132200
H	1.19373500	-3.15320400	1.99052600
O	-2.47155800	-0.98321300	-0.13206800
C	-2.02200000	5.91834200	-1.00361400
I	-1.04251500	0.25484800	3.00703700

I	-0.47577500	0.05730600	-3.20558100
N	-4.00500500	2.92296700	-0.76906900
C	-4.15598000	1.78112200	-1.66088000
H	-5.43322700	0.99406500	0.83338700
H	-3.21709700	-1.92780000	3.43933300
C	-4.88807700	4.03310800	-1.07238000
N	-3.88652700	-2.98970400	-1.00192800
H	-1.63421000	4.42113900	-3.24213700
C	-3.89236700	-2.44299300	-2.34802900
H	-1.27623000	-2.97079300	2.17757700
H	-6.98179600	-0.84095500	0.02913400
N	-2.68394800	4.01501300	1.26132500
C	-3.68018700	3.46394400	2.17172300
H	-2.65214200	6.26712500	-1.84290800
H	-5.19936400	1.42943000	-1.60664100
C	-1.47590400	4.45044000	1.96034800
H	-3.92773100	2.03180600	-2.71316400
H	-3.49671700	0.96534500	-1.34499700
H	0.78686600	-4.87557800	2.22495600
C	-6.30092900	-1.59538600	0.45854000
H	-3.81609500	0.49754800	1.40620000
H	1.25789200	-6.44755600	0.30046900
H	-4.74509300	4.84440600	-0.34516200
H	-4.72835600	4.43910700	-2.08983400
H	-3.88473600	-5.04419800	-1.41726200
H	-4.47206600	-4.58731900	0.20836500
H	3.92628000	-1.39163200	-2.54696800
H	4.96128800	-3.11120800	-0.89646800
H	-5.93537100	3.69727000	-1.00183500
C	-4.47333400	-4.30203600	-0.85071800
H	-3.24507100	-1.55663600	-2.39840100
H	-3.48878800	-3.19232800	-3.04702200
H	-4.56017700	3.13164000	1.61025200
H	-3.27469500	2.61399700	2.74868900
H	-6.29455200	-2.46315900	-0.21304000
H	1.74655600	-5.72834000	-1.25441500
H	4.37976600	-2.06761800	0.43208300
H	-4.91286500	-2.17708900	-2.68188200
H	-0.73118200	4.81311100	1.24260900
H	-1.73625400	5.27312000	2.64515700
H	-0.05634300	4.66054800	-2.42930100
H	-0.83973300	3.06137500	-2.39107800
N	2.95552100	-2.53373600	-1.06978600
C	3.10241400	-1.29143400	-1.82161900
H	-5.26115900	0.07623000	2.36378800
H	2.94140600	-5.89884400	0.06773300
H	-2.55240400	6.10676900	-0.06217400
H	-1.09446300	6.51627000	-1.00581400
H	-3.98599700	4.25533800	2.87467800
H	-1.03155900	3.62348300	2.54096900
O	0.49090400	-2.01820100	-0.04081200
P	1.41126600	-3.04651900	-0.72106600
N	0.79167600	-3.55126900	-2.17832700
C	-0.64257800	-3.67313500	-2.33869600
H	-6.72094200	-1.91155900	1.43328100
N	-3.30685400	-2.94565700	1.57148800
C	1.57105700	-3.78953300	-3.37330700
H	-0.93568800	-4.72679300	-2.50271700
H	-1.15854200	-3.28715900	-1.45184000
H	3.88114800	-3.78518300	0.34521600
H	-5.51240100	-4.33773900	-1.22993000
H	-0.97260700	-3.06011700	-3.19194400
H	1.35815300	-4.79609000	-3.77599600
H	1.32517000	-3.04239200	-4.14771600
H	2.64062800	-3.72337400	-3.14308600
N	1.47913900	-4.36701000	0.30940500
C	1.49674800	-4.17914100	1.74695300
N	-4.97515700	-1.03766100	0.57644600
C	-4.86833900	0.19269900	1.33708000
C	1.88424600	-5.66871600	-0.16665200
H	2.49831400	-4.36567300	2.17633200
O	1.55225600	1.01412500	0.18662400
C	2.55054500	1.74531800	0.64630700
H	5.57081200	0.13096900	4.93208000
C	2.85214400	3.01061400	-0.12542200
H	3.82446000	0.35681400	5.21711200
H	3.95399500	2.14524700	2.14971900
C	4.62572100	0.45463200	4.46689400
H	3.78089300	3.43305500	0.29202500
C	3.08524100	2.69747300	-1.60230400
C	1.72661200	4.02597000	0.05971600
H	2.19866200	2.21450800	-2.04277600
H	3.29440000	3.62021200	-2.16671500
H	3.94582100	2.02343500	-1.73091000
H	1.62792200	4.31254600	1.11843100
H	1.91393800	4.93930300	-0.52904300
H	4.98602500	-2.22781300	4.19169600
H	2.25023900	0.29583000	3.27996800
C	4.10464200	-1.84214300	3.65375100
H	3.23331100	-1.93092700	4.32254600
C	3.29991600	1.37687600	1.73085900
C	4.30536300	-0.38844900	3.22501500
C	3.04304600	0.13788300	2.52649900
H	2.65201300	-0.63254600	1.84057500
H	3.92930600	-2.48885700	2.78085300
H	4.72750600	1.51969700	4.21040700
C	5.47325300	-0.27238100	2.26377800
C	5.41518700	0.42592600	1.14910200
H	6.41949100	-0.74388000	2.58214500
H	6.40768900	2.88955600	0.92417200
H	0.76757600	3.59756700	-0.26570200
C	7.12504300	2.84168100	-1.10767300
H	5.97350600	-0.85921300	-1.16227700
H	7.01234000	0.13900500	-3.17205500
H	7.74296600	2.52382000	-3.15933400
H	7.45271100	3.88371200	-1.08753700
C	6.54309300	2.28876300	0.02292200
C	6.10495100	0.93990800	0.03247000
C	6.29208800	0.18280300	-1.14982300
C	6.87670200	0.74764200	-2.27477900
C	7.29238900	2.08147800	-2.26924400

HMPA-based reaction **Int IV** with phenylacetylene

H_{corr} = 1.2082

$-TS$ = -0.2085

$E_{sol(THF)}$ = -27834.5381

$G_{sol(THF)}$ = -27833.5384

H	2.32612800	-3.19065200	-4.18880800
H	0.75128500	-3.73088500	-4.81222700
C	-0.67137100	-3.13980500	-2.62007600
N	0.76806400	-3.25690700	-2.77784400
H	4.35876400	-4.04383900	-3.26294600
O	-0.94606100	1.77396000	0.87261100
C	-1.47946600	2.93058800	1.26874100

C	1.24436500	-3.02092400	-4.12717700	O	-1.79518400	-1.29352400	0.12644500
P	3.59551600	1.49470000	0.74485100	P	-2.98750800	-2.13376000	0.60208300
O	2.18677400	0.90722500	0.71306600	N	-2.62225400	-3.41259800	1.62028000
H	4.58812000	-1.09822600	-1.92660800	C	-1.49146800	-4.25270300	1.25624800
H	-0.93966300	-3.08130400	-1.55877500	H	0.27342400	3.64835600	-0.51334400
Sm	0.09662200	0.00718500	0.29128700	H	0.66721300	3.67105500	-2.90927800
N	3.81601300	2.31158900	2.19431500	C	-2.84692500	-3.31954200	3.05432200
C	3.28938900	1.69203600	3.40154200	H	-1.37401600	-4.28353500	0.16503300
C	0.32596700	4.71694000	-2.89637400	H	-0.55709600	-3.87106200	1.70016600
H	1.17766400	5.37049100	-2.64222300	H	-2.08907200	6.53907300	-1.19434100
C	4.93098300	3.20767200	2.42259600	H	0.91968200	2.80402400	2.51688800
I	0.23047800	0.68003000	-2.78586100	C	-1.98390300	3.98520100	-2.14562800
I	0.53309900	-1.29497300	3.13620900	C	-5.82111300	2.16173400	0.19018500
N	4.69911100	0.24222400	0.47965500	H	-1.95954500	-2.92866000	3.58021700
C	4.32566100	-1.08563000	0.94748500	H	-3.69855500	-2.66194900	3.26362500
H	-1.10565700	1.39954900	3.56350400	N	-3.65441500	-2.88954000	-0.73446400
H	-1.36551400	2.71177900	4.73707600	C	-3.86043600	-2.09110600	-1.92797000
C	6.10701900	0.54760800	0.65111300	H	-4.31617800	3.48530500	0.98542800
N	1.64829400	-4.44783500	-0.57767300	C	-4.59843700	2.82861200	0.16095000
H	-1.71774100	6.60685700	-2.93249700	C	-4.37603800	-4.14005800	-0.69141000
C	2.10977400	-4.45948300	0.79859100	H	-3.23772400	-1.18854900	-1.88622100
H	-1.03093800	-2.20985800	-3.08978500	H	-3.57461100	-2.67228000	-2.82030900
C	3.67927100	-1.44902200	-2.44315500	C	-3.72760700	2.68527700	-0.92947500
N	3.99715700	2.63855000	-0.41040300	C	-4.09754700	1.81441600	-1.96618200
C	4.22593400	2.19106300	-1.78029500	C	-5.31896500	1.14923200	-1.93549400
H	-0.47027700	7.05691300	-1.74308500	C	-6.19072500	1.32218900	-0.85989100
H	-2.27226100	4.79945200	0.67055800	H	-4.21841000	-4.62689500	0.27851300
C	3.27033500	3.90408600	-0.35478600	H	-5.46219700	-3.99262000	-0.84461200
H	4.52724800	-1.22438700	2.02603700	N	-4.10915600	-1.14487000	1.33101500
H	3.26216700	-1.26546100	0.75783900	C	-3.67612100	-0.02959300	2.15719500
H	-2.69662800	2.19999700	3.66805800	C	-2.49960400	3.48013200	-1.01381000
H	0.64586700	4.53187900	2.20039600	H	-2.41154600	3.84434900	-3.14144200
H	0.59405600	3.91960200	3.87295700	C	-5.48265400	-1.56325700	1.49283400
H	0.00373300	4.97935800	-3.91762000	H	-2.71183000	0.35331200	1.80375100
H	6.32598700	1.55825100	0.28092100	H	-4.40628000	0.78485600	2.05342900
H	6.43200400	0.47866800	1.70705800	H	-3.40307700	1.65826200	-2.79439000
H	1.13307300	-5.60809000	-2.23457200	H	-5.59587600	0.49082600	-2.76196600
H	2.39549600	-6.34130500	-1.19546700	H	-7.15394000	0.80716500	-0.84228800
C	4.23164000	-3.82593700	-2.18448200	H	-6.49559700	2.30652700	1.03791400
N	3.29700400	-2.75527700	-1.94233000	H	-6.14560000	-0.70312300	1.30857100
H	2.87354800	-0.72405700	-2.25985300	H	-5.73419800	-2.34428700	0.76345200
C	1.46615400	-5.73970700	-1.19741800	O	1.29027400	-1.87190200	-0.50864300
H	3.16111000	-4.79478500	0.88279000	P	1.72349900	-3.00993200	-1.43101500
H	2.00463800	-3.45885600	1.23967200	H	-1.18096500	-4.00764700	-3.07517500
H	4.81691200	1.26844400	-1.78143900	H	1.02157000	-1.99111700	-4.45757000
H	3.27757800	2.00851500	-2.31714100	H	4.07652400	1.13630800	3.94454900
C	-1.30136800	6.35991600	-1.94258000	H	5.75462000	2.71209700	2.96998300
H	-1.72038700	4.32517400	2.84260000	H	4.90913700	-1.83424900	0.38719200
C	-1.60966100	2.36636000	3.71947200	H	6.70838800	-0.16757400	0.06614600
C	0.33504800	3.68226600	2.82737200	H	1.48483900	-5.14935800	1.38772700
H	2.93095100	4.10756500	0.66834500	H	3.87641000	-1.47412000	-3.53047400
H	3.93273800	4.72239700	-0.68123700	H	4.79074900	2.97271300	-2.31214900
H	2.88882100	2.47010900	4.07013300	H	2.38746000	3.88221700	-1.01262900
H	2.47377200	0.99736100	3.15852200	H	5.22227400	-3.55400000	-1.78005200
C	-1.72112400	3.96118200	0.20133800	H	0.69679200	-6.31321000	-0.65172200
C	-0.81259900	4.90502400	-1.89829400	H	-1.67143800	-5.28171400	1.61081800
H	0.04327800	5.31246600	0.10009200	H	-3.08659100	-4.32352900	3.44285900
C	-0.42926500	4.49471700	-0.46549900	H	-4.91701800	-1.78760000	-2.03266200
H	5.32140300	3.58715100	1.47049400	H	-4.01495300	-4.81349300	-1.48856900
H	4.59376400	4.06646500	3.02750000	H	-3.58721500	-0.30452500	3.22385400
H	3.90352800	-4.74413600	-1.68166800	H	-5.69458600	-1.95401300	2.50681900
C	-1.16404500	3.38518400	2.67350000				

HMPA-based reaction **Product** with phenylacetylene
H_{corr} = 1.2090
$-TS$ = -0.2121
$E_{sol(THF)}$ = -27834.5448
$G_{sol(THF)}$ = -27833.5479

H	1.20120200	-4.15207200	-3.95519500
H	-0.47001200	-4.16492600	-4.55571900
C	-1.61184500	-3.16173800	-2.36223600
H	2.36828000	6.40763000	-2.27958300
H	-0.11728000	5.42488100	0.35897200
C	1.31873300	6.09810400	-2.41427000
H	-0.52758800	4.84171800	2.62831000
C	0.23036800	-3.64701000	-3.87939600
P	3.83118500	0.50938500	0.82532200
O	2.31631400	0.62826100	0.74780400
H	0.07246000	1.92187200	3.45034300
H	-1.86169600	-3.07150700	-1.29807700
Sm	0.03685500	-0.10706800	0.18094100
N	4.35238700	1.21925400	2.27053600
C	3.48547400	1.05199800	3.43034400
H	-2.63482800	5.46606000	0.45109200
C	-3.13033400	4.68327800	-0.12860200
C	5.76495500	1.17774100	2.59759100
I	0.25283200	0.32439700	-3.05603100
I	0.04448600	-1.22034000	3.28428500
N	4.31264700	-1.09456700	0.63090900
C	3.53012800	-2.12102500	1.30422300
C	-0.94070700	4.13235500	-1.18964300
H	-0.75461200	4.09222900	-3.33546800
C	5.70185100	-1.45414000	0.43692600
N	0.19535600	-4.88718100	-0.20192300
C	-0.44049200	2.88975900	3.55355700
C	0.55404200	-4.86252000	1.20414500
H	-1.65184000	-2.14793600	-2.79466000
H	-0.26324400	3.27127500	4.57025400
N	4.73895300	1.30103500	-0.35173400
C	4.54729900	0.83329100	-1.72216600
H	1.67776600	3.17012600	-0.62979200
H	1.96220400	2.74217100	-3.01954900
C	4.88189900	2.74643900	-0.26872900
H	4.02298300	-2.44664200	2.23908200
H	2.52931100	-1.74920400	1.55682600
H	-2.43645800	2.23352900	-2.38207300
H	-4.90030000	1.92187000	-2.18417500
H	-6.23279600	3.39429100	-0.68255700
H	-5.08113100	5.15115900	0.65869000
H	6.25266900	-0.62684900	-0.02721400
H	6.19998500	-1.72432000	1.38735000
H	-0.61638100	-6.01898700	-1.75543400
H	0.28087800	-6.98293500	-0.53774500
H	-1.51649000	2.69188000	3.44551200
H	1.84165300	5.07002600	2.05092000
H	1.73231000	4.51283300	3.73563100
C	-0.38982800	-6.11468400	-0.68618300
H	1.40801100	-5.53012600	1.42549400
H	0.79565400	-3.83697900	1.51369400
H	4.46176700	-0.25940600	-1.73809700
H	3.63479100	1.25167800	-2.18098300
H	0.69393600	6.67646600	-1.71556000
H	2.15301900	3.35953900	2.44185500
C	-0.28566600	4.20661700	-2.35615800
C	-4.50728400	4.51073500	-0.01484100
H	4.86405900	3.06972700	0.77885300
H	5.84308400	3.04399900	-0.71955900
H	3.57775800	1.93248300	4.08720900
H	2.44114300	0.94440100	3.11279600
C	-2.37892700	3.89246900	-1.01012900
C	-3.03056100	2.88664100	-1.73731800
C	-4.40581100	2.70945500	-1.61327100
C	-5.15172000	3.52660900	-0.76435000
H	6.37403400	1.28216200	1.68986800
H	6.00882600	2.01489100	3.27243400
H	1.91318300	4.09570500	-4.16840500
C	1.53017400	4.23196700	2.69113200
O	-2.32189000	-0.62389200	0.08276000
P	-3.71186300	-1.02304000	0.56136600
N	-3.80363100	-2.23830400	1.72074400
C	-2.98610500	-3.41895600	1.46977700
N	-0.28310100	-3.73346700	-2.52346400
O	-0.47803600	2.25469000	0.86870800
C	-3.88649300	-1.88579900	3.13229700
H	-3.00452200	-3.66693700	0.39958100
H	-1.94039000	-3.26346200	1.78690900
C	-0.18636700	3.42066600	1.10945600
H	1.01591100	6.37287500	-3.43679200
H	2.19349900	-6.02700000	-1.37995300
C	0.03996800	3.89748400	2.52410300
H	-2.89195800	-1.68983500	3.56951100
H	-4.51803700	-0.99857800	3.26319900
N	-4.56931800	-1.67826200	-0.72235700
C	-4.40830900	-1.07417200	-2.02927000
H	4.07819000	-2.85196100	-1.83230900
H	3.15678300	4.08235900	-2.89946600
C	-5.67224600	-2.60050500	-0.59027900
H	-3.50605100	-0.44917900	-2.03976700
H	-4.29104200	-1.85911300	-2.79512100
C	0.00183900	4.39722500	-0.02990200
C	1.16551300	4.58736100	-2.18787300
C	1.38475900	4.22965400	-0.70341300
H	3.83258200	-5.37707900	-1.60984200
H	-5.73067300	-2.95956500	0.44445000
H	-6.63699000	-2.13028000	-0.86207600
N	-4.50093800	0.33031100	1.14804200
C	-3.76126400	1.34941400	1.86881200
N	2.29884100	-3.96066900	-1.69802600
C	3.07143600	-2.88207300	-2.28422600
C	-5.93554000	0.37628600	1.28715500
H	-2.68789700	1.23418100	1.68214600
H	-4.06416000	2.34516800	1.50996900
H	2.16855100	4.83873300	-0.22964300
C	2.10597900	3.82848300	-3.11720400
C	2.78450700	-5.29553700	-1.94569000
H	2.57299400	-1.92151000	-2.09654900
H	-6.29697500	1.38100400	1.01200100
H	-6.41041400	-0.34902500	0.61383100
O	0.71917700	-2.34055800	-0.40764800
P	0.72752600	-3.64518000	-1.18822200
H	-2.36313400	-3.80567100	-2.85214300
H	0.33723200	-2.59909800	-4.21137400
H	3.75175700	0.15264400	4.01565400
H	6.05193700	0.23916500	3.10955400
H	3.40892100	-2.99333400	0.64241700
H	5.76504200	-2.33137600	-0.23061300
H	-0.30550900	-5.19518800	1.80856500

H	5.41971400	1.12828200	-2.32660800
H	4.07291400	3.26747400	-0.80802500
H	-1.32976800	-6.32508600	-0.14569100
H	-3.40839100	-4.27329500	2.02388600
H	-4.35866500	-2.72162000	3.67447200
H	-5.28481900	-0.45806700	-2.30430300
H	-5.52602900	-3.46865600	-1.25753100
H	-3.93714400	1.29044700	2.95950600
H	3.18564300	-3.00461900	-3.37696600
H	-6.27396800	0.16411400	2.32047000
H	2.75354400	-5.57419600	-3.01777200

HMPA-based reaction **PreTS II** with acrylonitrile

H_{corr} = 1.1422

$-TS$ = -0.2107

$E_{sol(THF)}$ = -27696.9613

$G_{sol(THF)}$ = -27696.0298

H	-0.96400900	4.26027100	3.81948000
C	-1.79922300	3.08397500	1.56561000
H	-4.19959600	-3.09787500	-0.22765100
H	-1.43174400	-5.40152900	0.37021700
C	-3.96090600	-2.59783400	0.73337200
H	-0.21454300	-5.50778200	-1.48267300
C	-0.21116700	3.61806400	3.33396600
P	3.91997600	-1.01111300	0.23832700
O	2.50159900	-0.61794000	-0.16968300
H	0.16517100	-2.86521700	-3.01298800
H	-2.56468200	3.72777900	2.02881000
Sm	0.23175200	-0.31666800	-0.29198700
N	4.48077700	-2.18228900	-0.82088600
C	4.18144200	-2.00959400	-2.23396400
H	-4.33334700	-2.66860800	4.21563200
H	-2.26798100	3.82242200	-2.87127500
C	5.61507600	-3.03554200	-0.53906700
I	0.14317500	-0.32386900	2.88810800
I	0.85414400	0.19087100	-3.33064500
N	4.85138200	0.39838400	0.25616000
C	4.40070400	1.51418000	-0.56418500
H	2.74266300	2.72670200	3.40335100
H	-5.20106600	3.49671300	-0.80156000
C	6.29176200	0.23023700	0.29174600
N	0.49612900	4.47222800	-0.38761400
C	-0.15233800	-3.91573500	-2.92519500
C	1.23093500	4.33464200	-1.63265200
H	-1.95292400	3.09159400	0.47964800
H	0.37255700	-4.49206800	-3.70391700
N	4.14329600	-1.70151900	1.74282100
C	4.24696300	-0.85902800	2.92759600
H	2.05838300	-3.46730700	-1.22649800
H	-3.28009900	-1.76608900	0.50184900
C	3.39534300	-2.93502700	1.98800400
H	4.70822900	1.41146500	-1.62165600
H	3.30862800	1.59328000	-0.52173700
H	-2.80062100	-0.07339200	3.49726300
H	-1.92322700	1.17067900	-4.79405100
H	3.81926300	4.95337800	1.57621400
H	-3.19365500	-1.95068100	-3.76813200
H	6.56161900	-0.61165100	0.94450800
H	6.72474700	0.05056000	-0.71123700
H	-0.87589600	5.96523400	-0.91729900
H	-0.58836900	5.77020700	0.83602800
H	-1.23079900	-3.96967000	-3.13900800
H	1.89747800	-5.00235300	-0.33826500
H	2.20142200	-5.01836200	-2.09804700
C	-0.10844900	5.76330800	-0.15022900
H	2.16025000	4.93488300	-1.63352000
H	1.47532500	3.27847700	-1.80915700
H	4.76478800	0.07479400	2.68089400
H	3.25141300	-0.62024600	3.34088500
C	-3.49201300	-2.00991200	3.99539900
H	-5.42168700	-1.81130300	-1.80597100
H	-1.40729100	-2.66244400	1.80879200
H	-3.75770900	-5.15959300	3.07432800
H	3.25617800	-3.48825500	1.05095000
H	3.96057700	-3.56728800	2.69132800
H	3.95764400	-2.98884100	-2.68575600
H	3.29905300	-1.36816900	-2.36671000
C	-3.66838000	-0.71197500	3.69290600
H	-4.70331100	2.08895900	1.22850500
H	0.77745700	4.04938700	3.53158200
H	-2.47979000	-2.40267000	4.09718000
H	6.53091700	-2.68711400	-1.05233900
H	5.80709300	-3.06859300	0.54028200
H	-5.21502400	-4.41015700	2.35397300
C	1.66866900	-4.49378600	-1.28802600
O	-1.92278700	0.44716000	-0.65564900
P	-3.07966200	0.77263700	-1.61645500
N	-2.62131200	1.73533700	-2.89285100
C	-1.75193500	2.86873800	-2.65222300
H	2.55213900	5.29507100	2.78535500
N	-0.47705500	3.59045000	1.90466700
C	-2.86867000	1.42130500	-4.28317100
H	-1.41148400	2.87574100	-1.60932100
H	-0.85150800	2.78309500	-3.28025200
O	-0.25404600	-2.39047100	-0.40032700
C	-0.51839100	-3.69355900	-0.42926900
H	-4.90924800	-2.18485400	1.11903400
C	0.16095800	-4.47226600	-1.54030600
H	-3.54865400	0.56475200	-4.36223500
H	-3.33582000	2.28287200	-4.79285000
N	-4.22821300	1.64331100	-0.77032200
C	-4.60568000	1.21547200	0.56446800
N	2.22859500	3.59970000	1.53255400
C	2.85612500	2.55293300	2.31774300
C	-5.12993900	2.57665500	-1.40618600
H	-5.56989500	0.67803300	0.57825700
H	-3.83063600	0.55619700	0.97207400
H	-1.54610500	-4.23442000	2.61801000
C	-4.17059200	-4.70513500	2.15864400
C	2.73049200	4.93545600	1.75329900
H	2.40140200	1.58311600	2.07927200
H	-4.75341200	2.84215800	-2.40217900
H	-6.15195900	2.16586900	-1.51165600
N	-3.75532500	-0.61171300	-2.24134900
C	-2.88640600	-1.65980000	-2.74867800
H	3.93146800	2.50562500	2.08089300
H	-4.21019500	-5.51273600	1.39692700
C	-5.17577100	-0.86072500	-2.30810200
H	-1.84764000	-1.30597100	-2.80338600
H	-2.91322600	-2.54699400	-2.09542700
C	-1.27085500	-4.32731500	0.49891100
C	-3.34547200	-3.55686400	1.69004500
C	-1.85102600	-3.66655800	1.71899100
H	2.26400100	5.64204300	1.05543000

H	-5.73119900	-0.06041200	-1.80439800
H	-5.52635400	-0.92360300	-3.35506000
O	0.77142700	1.94013800	0.13623600
P	0.74961900	3.31799800	0.79783900
H	-1.93222800	2.04807500	1.91977000
H	-0.25729600	2.60908500	3.77902600
H	5.03576100	-1.56315200	-2.77632800
H	5.40071200	-4.06036600	-0.88700700
H	4.83880300	2.44467300	-0.16747800
H	6.75741100	1.13961800	0.70482600
H	0.60185100	4.67864200	-2.46884900
H	4.82951800	-1.39757600	3.69242000
H	2.40124900	-2.71726800	2.41468400
H	0.63285700	6.58407700	-0.19317700
C	-4.95418500	-0.11620900	3.56180300
N	-5.98988600	0.40166600	3.42842400

HMPA-based reaction **TS II** with acrylonitrile
H_{corr} = 1.1415
$-TS$ = -0.2079
$E_{sol(THF)}$ = -27696.9616
$G_{sol(THF)}$ = -27696.0281

H	0.85201200	4.15620800	-3.99238800
C	1.72182000	3.11746200	-1.68547400
H	3.90092500	-2.97063200	0.37001600
H	1.37545500	-5.43625200	-0.15906800
C	3.81697900	-2.56980400	-0.65995900
H	0.26337900	-5.42182500	1.75802500
C	0.12308700	3.50550000	-3.48232400
P	-3.88188200	-1.09014900	-0.19147700
O	-2.47836100	-0.63698200	0.20585800
H	-0.03506100	-2.69334800	3.14792300
H	2.46139100	3.76786100	-2.18044400
Sm	-0.21192300	-0.29928900	0.31191100
N	-4.43089200	-2.20255900	0.93525500
C	-4.16396000	-1.92283500	2.33770000
H	4.32921600	-3.03467600	-4.01320800
H	2.16630800	4.02421400	2.50548000
C	-5.53884300	-3.10155800	0.69346300
I	-0.08525100	-0.41662200	-2.86318300
I	-0.82830700	0.34744000	3.32456700
N	-4.84649000	0.29240800	-0.30904700
C	-4.43802700	1.46066500	0.45898700
H	-2.77537100	2.49390200	-3.53343800
H	5.18237900	3.61544900	0.55192700
C	-6.28155700	0.08535200	-0.35713000
N	-0.64400800	4.48907000	0.19566700
C	0.27821700	-3.74795200	3.10586500
C	-1.36983000	4.36949400	1.44782900
H	1.87132500	3.19072200	-0.60143200
H	-0.20625500	-4.27695100	3.94214800
N	-4.06561600	-1.88218800	-1.65073200
C	-4.16938600	-1.11946300	-2.88863700
H	-2.01000300	-3.38527600	1.48568200
H	3.15839200	-1.69079600	-0.60608600
C	-3.27335000	-3.10336500	-1.80272000
H	-4.76355900	1.40589100	1.51470700
H	-3.34743700	1.56383800	0.43140300
H	2.86245000	-0.35138700	-3.50024300
H	2.02488500	1.59809400	4.74665400
H	-3.97208800	4.73905100	-1.80290400
H	3.34419700	-1.66212500	3.89467100
H	-6.51927500	-0.79854700	-0.96530900
H	-6.72672900	-0.04892500	0.64761500
H	0.66175600	6.06095500	0.66134700
H	0.38331400	5.78128400	-1.08193900
H	1.36637100	-3.79268900	3.26610500
H	-1.90041800	-4.97743100	0.69511600
H	-2.11809700	-4.87364600	2.46431800
C	-0.09606500	5.79420200	-0.09545500
H	-2.32620000	4.92521300	1.42690400
H	-1.56409800	3.31139600	1.66982100
H	-4.72013100	-0.18856300	-2.71219100
H	-3.17304600	-0.87646800	-3.29758200
C	3.50195600	-2.34543800	-3.83952800
H	5.35197000	-1.63555700	1.64266400
H	1.29805100	-2.79694400	-1.76871700
H	3.76121700	-5.34131800	-2.76023200
H	-3.14274500	-3.59500300	-0.83088000
H	-3.79995100	-3.79372100	-2.48072600
H	-3.93643900	-2.86321800	2.86383100
H	-3.29241800	-1.26122500	2.44049400
C	3.71292600	-1.02969900	-3.62234400
H	4.64534500	2.11292400	-1.39182700
H	-0.88097700	3.88691600	-3.70327000
H	2.48369900	-2.70573000	-3.98790100
H	-6.47205600	-2.74324000	1.16709600
H	-5.71044900	-3.21118200	-0.38425500
H	5.17421400	-4.50396500	-2.04670900
C	-1.62295100	-4.40835200	1.59635300
O	1.93223700	0.51164400	0.62008900
P	3.08409300	0.93303700	1.55036500
N	2.58874300	1.96348600	2.75700400
C	1.67035000	3.03707400	2.44069000
H	-2.70803400	5.09130600	-3.01213700
N	0.38126800	3.55208300	-2.05205800
C	2.92910900	1.82246100	4.15524900
H	1.26085100	2.90358200	1.43231900
H	0.81700900	3.00721900	3.13522400
O	0.28155900	-2.36890500	0.50448400
C	0.52558100	-3.67322500	0.58902200
H	4.82450700	-2.24469600	-0.96805900
C	-0.10510600	-4.38232600	1.77312700
H	3.64875500	1.00589100	4.28665700
H	3.38389400	2.75388800	4.53841800
N	4.20583700	1.76680000	0.63380500
C	4.57206600	1.27344500	-0.68185600
N	-2.32398900	3.46045500	-1.69226800
C	-2.89709100	2.35890600	-2.44322900
C	5.10808100	2.73577300	1.21336600
H	5.54373000	0.75030300	-0.68353400
H	3.80105000	0.58313700	-1.04318000
H	1.49905100	-4.42020900	-2.46160900
C	4.12335500	-4.78977700	-1.87712500
C	-2.88165500	4.76337600	-1.96876800
H	-2.40704200	1.41872900	-2.16036700
H	4.72834400	3.06633800	2.18872100
H	6.12938000	2.33219000	1.34967900
N	3.80384600	-0.36361200	2.30513100
C	2.95014600	-1.37532300	2.90498800
H	-3.97269500	2.27682000	-2.21807900
H	4.12280900	-5.51032100	-1.03267800
C	5.20700600	-0.69016700	2.19232600
H	1.93237900	-0.98376600	3.04951300

H	2.88471600	-2.27300500	2.26878100
C	1.22314800	-4.36822500	-0.33842200
C	3.26533900	-3.60957000	-1.57087300
C	1.77444400	-3.77623800	-1.60614800
H	-2.45490600	5.51588200	-1.29395200
H	5.74479300	0.10021800	1.65545600
H	5.66158500	-0.79950400	3.19311900
O	-0.80196400	1.92781000	-0.21593300
P	-0.83880400	3.27445100	-0.93903700
H	1.89895500	2.07120500	-1.98525300
H	0.21303700	2.48077900	-3.88245400
H	-5.03534300	-1.45127600	2.82947100
H	-5.30546400	-4.09495700	1.11294100
H	-4.89009800	2.35819100	0.00610700
H	-6.76255700	0.95786700	-0.82803300
H	-0.75615500	4.77730400	2.26664800
H	-4.71944900	-1.72330800	-3.62824900
H	-2.27497700	-2.88033900	-2.21653000
H	-0.87241300	6.58300300	-0.08522400
C	5.01139700	-0.47336100	-3.47176900
N	6.06197600	0.01205600	-3.32499700

HMPA-based reaction **Int III** with acrylonitrile
H_{corr} = 1.1441
$-TS$ = -0.2058
$E_{sol(THF)}$ = -27697.0082
$G_{sol(THF)}$ = -27696.0699

H	0.53228400	4.25304400	-4.00327400
C	1.55357900	3.03239100	-1.85094000
H	3.83934200	-2.87885300	0.24472200
H	1.72786600	-5.28943500	-0.46905400
C	4.05148600	-2.39915600	-0.72191300
H	0.59645800	-5.44281900	1.41982700
C	-0.16984800	3.57769500	-3.48724100
P	-3.84888300	-1.20619000	-0.02766200
O	-2.41006600	-0.80445000	0.29045800
H	0.10946100	-2.82362900	2.96050500
H	2.27733800	3.77447200	-2.22778000
Sm	-0.17377800	-0.29842200	0.27943900
N	-4.32250100	-2.39224300	1.05751900
C	-3.91874400	-2.23912500	2.44631100
H	5.10364000	-2.64252200	-3.29707400
H	2.15669100	4.06765100	2.84876800
C	-5.45875300	-3.26217800	0.84527800
I	-0.32669300	-0.37869000	-2.89451800
I	-0.71817500	0.23753800	3.33571300
N	-4.79983400	0.18879500	0.02387200
C	-4.30608400	1.31824100	0.79990400
H	-3.11101300	2.59619800	-3.33968700
H	5.11834700	3.76837400	0.46133300
C	-6.23558800	-0.00343000	0.10083800
N	-0.63640700	4.46078800	0.24277900
C	0.47843500	-3.85662600	2.86533500
C	-1.30526400	4.36488800	1.52781300
H	1.73302000	2.87462400	-0.78052600
H	-0.00513600	-4.46042200	3.64990400
N	-4.16392100	-1.89282500	-1.51764200
C	-4.38571300	-1.05273200	-2.68713700
H	-1.77315800	-3.50916000	1.20131900
H	3.49523800	-1.45099100	-0.74393000
C	-3.40588900	-3.10527300	-1.82540300
H	-4.51140000	1.21104800	1.88122500
H	-3.22558300	1.42971500	0.65696400
H	2.31925400	-1.23585000	-3.52281200
H	2.01023800	1.37786500	4.61913900
H	-4.11794500	4.76725400	-1.42862900
H	3.42802100	-1.52762200	3.78952700
H	-6.54200500	-0.85108600	-0.52793700
H	-6.58641900	-0.18753100	1.13462700
H	0.72373100	6.00521000	0.63625000
H	0.36075400	5.72012100	-1.09020900
H	1.56124600	-3.85453400	3.06404500
H	-1.54928000	-5.02739600	0.29810500
H	-1.83013200	-5.07175700	2.06125100
C	-0.07356100	5.75115200	-0.08335200
H	-2.25130400	4.93856900	1.54784500
H	-1.50955700	3.31207800	1.76498200
H	-4.90542100	-0.13294800	-2.39621000
H	-3.43384600	-0.78791300	-3.17958100
C	4.00654300	-2.70286800	-3.22239400
H	5.54628000	-1.49368600	1.70604300
H	1.58613300	-2.56425400	-1.90363700
H	4.05542900	-5.34923100	-2.54344600
H	-3.19709600	-3.66589800	-0.90592200
H	-4.00051400	-3.74203500	-2.49981400
H	-3.63399200	-3.22010000	2.85885200
H	-3.04789500	-1.57348500	2.52448600
C	3.40755000	-1.36818400	-3.50121200
H	4.52963400	2.32010600	-1.49485500
H	-1.17985200	3.98934000	-3.60085000
H	3.66687800	-3.40200900	-4.01050400
H	-6.34162500	-2.93706000	1.42707400
H	-5.72602800	-3.28466100	-0.21823900
H	5.44448600	-4.51884700	-1.79634400
C	-1.33325800	-4.51594600	1.24915700
O	1.92242200	0.62768900	0.59290100
P	3.11594200	1.02549200	1.47887000
N	2.70764800	2.03685600	2.73778800
C	1.75180900	3.10088500	2.49887600
H	-2.97198500	5.15851900	-2.73936800
N	0.19636500	3.50340200	-2.08381700
C	2.93889200	1.72338500	4.13288400
H	1.52209500	3.18201500	1.42905400
H	0.80614600	2.88387900	3.02132400
O	0.50293000	-2.32051700	0.36387200
C	0.80706300	-3.61396900	0.36789900
H	5.13008200	-2.17504100	-0.77497500
C	0.17473000	-4.42562100	1.48339300
H	3.70034500	0.93970800	4.22408700
H	3.30575200	2.62250800	4.65766200
N	4.18591700	1.89632700	0.53673400
C	4.46364500	1.45447600	-0.81784300
N	-2.47051300	3.48491400	-1.51853500
C	-3.11196800	2.40913800	-2.25028300
C	5.11293900	2.85925700	1.08626700
H	5.41344000	0.89513400	-0.89193700
H	3.64936700	0.81219600	-1.16910300
H	1.80687000	-4.12717700	-2.69653800
C	4.35253300	-4.65785800	-1.73793900
C	-3.04776100	4.79592700	-1.69575200
H	-2.58263100	1.46509600	-2.06768100
H	4.80490000	3.13581900	2.10248200
H	6.14852000	2.47087700	1.12164700
N	3.86977600	-0.30889000	2.12526200

C	3.07273700	-1.34310700	2.76057900
H	-4.15601100	2.29861600	-1.91409000
H	4.12416700	-5.14066800	-0.77625400
C	5.29969800	-0.50621500	2.13004000
H	2.01890300	-1.03765700	2.82145800
H	3.12328200	-2.28305400	2.18880100
C	1.56333600	-4.21406400	-0.58052300
C	3.61909000	-3.32275400	-1.85619400
C	2.09086700	-3.53698100	-1.80406400
H	-2.55601800	5.52516900	-1.03998700
H	5.79568700	0.25663700	1.51787600
H	5.71484200	-0.45785000	3.15426700
O	-0.88121000	1.90551600	-0.17003200
P	-0.93747000	3.25750000	-0.88324000
H	1.72380400	2.07044300	-2.36259600
H	-0.13007300	2.58489800	-3.96773500
H	-4.74001200	-1.82751200	3.06203300
H	-5.20331000	-4.28765300	1.16253500
H	-4.80422500	2.23452200	0.44293800
H	-6.74673000	0.89718500	-0.27612000
H	-0.64727700	4.76675700	2.31426000
H	-5.01483100	-1.60572800	-3.40338000
H	-2.44555900	-2.85924400	-2.31018600
H	-0.82945300	6.55899400	-0.04583800
C	4.17495800	-0.23422400	-3.72658300
N	4.82324400	0.73218900	-3.90133000

HMPA-based reaction **TS III** with acrylonitrile

H_{corr} = 1.1438

$-TS$ = -0.2035

$E_{sol(THF)}$ = -27697.0014

$G_{sol(THF)}$ = -27696.0612

H	-1.77865700	4.10181600	-4.19915900
C	-0.30979900	4.03294000	-2.01078400
H	2.74048400	-3.93779900	-4.44535300
H	3.37958800	-4.13225700	0.07082500
C	3.04741800	-2.94829700	-4.06909300
H	2.61038800	-4.29059300	2.16202300
C	-2.06054700	3.28313300	-3.51874800
P	-3.07847400	-2.39231300	0.36746100
O	-2.23372000	-1.12924400	0.56538900
H	2.20006800	-1.36033200	2.97779000
H	-0.06534000	4.83718400	-2.72242300
Sm	-0.16072500	-0.14301000	0.31631000
N	-2.84570200	-3.41331600	1.67662000
C	-2.76519100	-2.80243300	2.99475600
H	3.18166200	-0.90501100	-2.45892000
H	0.69819800	4.85530500	1.23743900
C	-3.29031100	-4.79197000	1.68034000
I	-0.15529400	-0.39095800	-2.86635100
I	-0.35795500	0.60258700	3.32781100
N	-4.67811300	-1.87133100	0.17163200
C	-5.05419200	-0.62711500	0.82566100
H	-4.14988200	1.11404400	-3.16526100
H	3.23378600	4.89160000	-1.89759700
C	-5.71550600	-2.88567300	0.20385300
N	-2.56552100	3.92084600	0.32640200
C	2.76140900	-2.30760900	2.97196000
C	-2.81061800	3.44749000	1.67799500
H	-0.11931400	4.40668900	-0.99717600
H	2.89352300	-2.62154300	4.01994900
N	-2.78564400	-3.36178600	-0.95462400
C	-3.29694300	-2.98600400	-2.26758900
H	0.04752900	-2.77821400	2.90362300
H	2.19748700	-2.25844000	-4.18863700
C	-1.48258600	-4.02540600	-1.01568400
H	-5.30644900	-0.77084500	1.89372500
H	-4.23357700	0.09546700	0.74872200
H	3.22925500	-0.90411000	-0.11038800
H	2.71746900	3.89709500	3.50880500
H	-5.90711100	2.41472700	-1.09341600
H	4.75888800	1.56094300	3.13109800
H	-5.36932900	-3.79923800	-0.29811300
H	-6.02450700	-3.14539900	1.23462900
H	-2.13719900	5.93993400	0.70067200
H	-2.66619200	5.55984800	-0.96613900
H	3.75871200	-2.13731000	2.54078400
H	0.10470200	-4.45787300	2.30366200
H	0.82430000	-4.04410200	3.88499200
C	-2.82379500	5.32511600	0.09338200
H	-3.88381800	3.49639100	1.93907500
H	-2.45143900	2.41655800	1.79609400
H	-4.23727600	-2.43271600	-2.16156300
H	-2.57017600	-2.35929100	-2.81268500
C	3.91276800	-1.64258400	-2.09441400
H	5.88028400	0.70821600	0.36809200
H	1.93984900	-4.42411200	-1.98870600
H	5.03994500	-4.04461100	-1.44435800
H	-1.04665600	-4.11036900	-0.01303100
H	-1.60434700	-5.03362000	-1.44417400
H	-2.09535900	-3.39982100	3.63107800
H	-2.34510700	-1.79023200	2.92401900
C	3.92217500	-1.60193600	-0.58799100
H	2.65469400	2.81614300	-3.06897500
H	-3.14323500	3.13159000	-3.60987000
H	4.89745000	-1.37624000	-2.51647800
H	-4.26333100	-4.91319500	2.19233200
H	-3.38449800	-5.16600300	0.65371000
H	5.45566300	-3.76354600	-3.14520000
C	0.66510300	-3.68596100	2.85577100
O	1.70345800	1.18025800	0.20461400
P	2.76369600	2.26553100	0.44721400
N	2.20601500	3.40458900	1.52844000
C	0.81245400	3.79497800	1.53296800
H	-5.22787800	3.37844900	-2.43376800
N	-1.71878700	3.68452600	-2.15901900
C	3.04468000	4.10383200	2.47512500
H	0.23987300	3.16688300	0.84215900
H	0.38097900	3.64902700	2.53582300
O	0.87621100	-2.02887700	0.52230700
C	1.78922300	-2.94950800	0.74679000
H	3.87452300	-2.59338600	-4.70576000
C	1.99817300	-3.37355600	2.18409600
H	4.08803400	3.78442000	2.36803100
H	2.99523100	5.19616600	2.31145500
N	3.06909300	2.99840400	-1.02655400
C	3.07785300	2.21393600	-2.24869900
N	-3.84867000	2.16885700	-1.34597900
C	-3.90415100	0.92923400	-2.10383100
C	3.70080300	4.29596600	-1.09502900
H	4.09669100	1.89947600	-2.54245000
H	2.44639700	1.32484500	-2.12648700
H	1.46627300	-2.71168000	-1.90350300
C	4.62963900	-4.02672700	-2.46494200

C	-5.02814400	2.99943300	-1.41266000
H	-2.93407000	0.41540600	-2.05929100
H	3.56046900	4.83524000	-0.14865400
H	4.78539900	4.23586700	-1.30547900
N	4.18557700	1.71494100	1.10049500
C	4.14972600	1.02567700	2.38122200
H	-4.67157400	0.26267800	-1.68013900
H	4.29854200	-5.04750700	-2.71760400
C	5.46585900	1.72719200	0.42042000
H	3.11655800	0.96276200	2.75180400
H	4.54694300	0.00535000	2.27322000
C	2.64970900	-3.38555200	-0.25773400
C	3.47941500	-3.03002700	-2.60936800
C	2.29418900	-3.41241100	-1.71530100
H	-4.93186100	3.85823800	-0.73595400
H	5.35421200	2.11090500	-0.60029600
H	6.18771900	2.36929700	0.95738900
O	-1.49281200	1.63093200	-0.40821300
P	-2.36415400	2.79130100	-0.88213700
H	0.35193400	3.17013100	-2.19693700
H	-1.53745600	2.36221700	-3.83337400
H	-3.75658300	-2.75527200	3.48450100
H	-2.54904400	-5.41330000	2.21042000
H	-5.94349800	-0.21421900	0.32105500
H	-6.60448100	-2.51892200	-0.33448600
H	-2.25170900	4.07073800	2.39398400
H	-3.48645700	-3.90214600	-2.85017800
H	-0.78289400	-3.44852700	-1.64142000
H	-3.85782500	5.60133300	0.37292200
C	5.11507200	-1.79066700	0.13088900
N	6.09134300	-1.94804000	0.76371800

HMPA-based reaction **Int IV** with acrylonitrile
H_{corr} = 1.1462
$-TS$ = -0.2039
$E_{sol(THF)}$ = -27697.0306
$G_{sol(THF)}$ = -27696.0882

H	0.91412500	3.77032100	4.65493700
C	-0.61361900	3.07583900	2.54052800
H	-1.71596000	-5.05654500	3.92261900
H	-3.42523500	-4.00031100	-0.25661400
C	-2.08359700	-4.02067800	3.83991900
H	-2.22522500	-4.52696300	-2.20285500
C	1.37039500	3.03716200	3.96857200
P	3.41406500	-1.87452800	-0.60808600
O	2.18683900	-0.96690000	-0.65603800
H	-0.59392500	-2.19040000	-3.36113700
H	-1.12537100	3.94950600	2.98144700
Sm	0.11975400	-0.04749700	-0.34365500
N	3.31616300	-2.96619100	-1.87437900
C	2.77663100	-2.49965200	-3.14292400
H	-2.55629700	-1.68376300	2.78656600
H	-1.90671100	5.11513200	-1.55671400
C	4.13252800	-4.15805000	-1.96236400
I	0.39186300	-0.70710700	2.75585100
I	0.43190300	1.06011800	-3.24769700
N	4.76945800	-0.86901500	-0.63961200
C	4.61284400	0.46907900	-1.19251700
H	3.94469900	1.43822500	3.22399300
H	-4.48142800	4.32518800	1.57486000
C	6.05114400	-1.48694800	-0.91890100
N	1.59861900	4.43224400	0.39817100
C	-1.31361600	-3.02008800	-3.44234700
C	1.99521000	4.44250000	-0.99857200
H	-0.91968300	2.96960600	1.49311300
H	-0.97802700	-3.69029900	-4.25056900
N	3.64060700	-2.84546500	0.73260300
C	4.18610400	-2.27034200	1.95631500
H	0.68758500	-3.74926800	-1.63434600
H	-1.21989200	-3.34091200	3.90315700
C	2.60869000	-3.84754700	0.99690300
H	4.69541100	0.48316900	-2.29521900
H	3.63660100	0.88015300	-0.91108800
H	-2.88102400	-1.18384700	0.58243400
H	-2.10281600	2.76752800	-3.50918100
H	5.23074700	3.62703400	1.47644600
H	-3.77422900	0.18670100	-3.39913900
H	6.10890400	-2.47512100	-0.44171900
H	6.23893000	-1.60736100	-2.00323100
H	0.57906300	6.26099000	0.49075100
H	1.10026100	5.58966200	2.06220700
H	-2.28135200	-2.58752000	-3.73855000
H	-0.19647700	-5.09758600	-0.87954800
H	0.19356000	-5.15577500	-2.61839600
C	1.38945600	5.72233000	1.01205300
H	3.01404800	4.85064100	-1.13662400
H	1.94696000	3.42501600	-1.40989900
H	4.90948100	-1.48445400	1.71107300
H	3.38772200	-1.84334000	2.58822800
C	-3.31669900	-2.36192100	2.37184300
H	-6.09123700	0.14186300	-1.38724200
H	-1.67786100	-5.01875800	1.09188500
H	-4.63681600	-4.62014300	1.55571600
H	2.16160000	-4.19457700	0.05785300
H	3.06617300	-4.70886800	1.50960900
H	2.11927100	-3.27313500	-3.57096300
H	2.17988800	-1.58917100	-2.99820900
C	-3.41292100	-2.11070100	0.84752600
H	-3.83460500	2.24218400	2.83488400
H	2.45440200	3.20356300	3.97064700
H	-4.26650300	-2.17241100	2.89380600
H	4.97566300	-4.02827800	-2.66608800
H	4.53381400	-4.41867100	-0.97535200
H	-4.68604500	-4.64725500	3.32799400
C	-0.11163600	-4.48954500	-1.79363800
O	-1.83977900	1.14545900	-0.15143600
P	-3.10825000	1.88220100	-0.60992900
N	-2.83191700	3.23766300	-1.56091100
C	-1.74171900	4.10698800	-1.14309500
H	4.40880900	4.04233000	3.00526400
N	0.82945300	3.22577000	2.63670800
C	-3.00192600	3.16256900	-3.00546200
H	-1.72445600	4.18944400	-0.04820200
H	-0.76432600	3.72939600	-1.48806200
O	-1.00968500	-1.80802900	-0.75215600
C	-1.82032300	-2.83492600	-0.99827600
H	-2.73541200	-3.82968900	4.70838900
C	-1.42859600	-3.76674700	-2.11342100
H	-3.85823000	2.52391500	-3.25259900
H	-3.21353300	4.17509900	-3.38585000
N	-3.84808300	2.50229200	0.75652200
C	-3.94872400	1.63800800	1.92009100
N	3.33660500	2.76549400	1.67883400
C	3.76842900	1.45699000	2.13280700

C	-4.73773300	3.64231200	0.74637900
H	-4.92028500	1.11298400	1.96458000
H	-3.14087500	0.89580800	1.90186500
H	-0.98860500	-3.41651500	1.48430400
C	-4.02442400	-4.77885700	2.45677000
C	4.24900500	3.85568900	1.92534700
H	3.00145300	0.70685100	1.89852900
H	-4.63141200	4.18449100	-0.20123400
H	-5.79553700	3.34407600	0.87165600
N	-4.12436500	0.83220500	-1.40400200
C	-3.59965800	-0.13499700	-2.35658500
H	4.70396500	1.17373000	1.62271300
H	-3.67991200	-5.82579100	2.45043500
C	-5.54735600	1.09130500	-1.50522300
H	-2.52192600	-0.27373400	-2.21245000
H	-4.09283300	-1.10494700	-2.19446700
C	-2.67705600	-3.29609500	0.13981300
C	-2.83809100	-3.81556200	2.53214600
C	-1.92714800	-3.96797000	1.30824900
H	3.87493900	4.77919400	1.46577200
H	-5.87235100	1.76313600	-0.70130400
H	-5.81913700	1.54807800	-2.47600700
O	1.27673700	1.85491300	0.35433000
P	1.73499200	3.00192200	1.25285900
H	-0.93513400	2.15820500	3.06004800
H	1.16423500	2.01932500	4.34332600
H	3.58324300	-2.28569200	-3.86789300
H	3.51712800	-5.00062200	-2.32115700
H	5.40507200	1.11574300	-0.78055600
H	6.85873200	-0.86381000	-0.50172300
H	1.29515000	5.06852700	-1.57506500
H	4.70001600	-3.06456800	2.52149200
H	1.80874300	-3.43164800	1.63337000
H	2.29487600	6.35772400	0.96973400
C	-4.78027300	-1.95765200	0.37825300
N	-5.87595500	-1.84071100	0.00558800

HMPA-based reaction **Product** with acrylonitrile
H_{corr} = 1.1462
$-TS$ = -0.2047
$E_{sol(THF)}$ = -27697.0324
$G_{sol(THF)}$ = -27696.0909

H	0.22704100	3.33306200	4.78234600
C	-1.15020400	2.75545400	2.51701200
H	-1.99122300	-4.58207900	4.68179800
H	-3.25246600	-4.15814000	0.16233900
C	-2.23222100	-3.54689200	4.39120900
H	-1.37050600	-5.30309800	-0.79499400
C	0.79299700	2.81160100	3.99152600
P	3.91394400	-1.28183700	-0.63764500
O	2.52800500	-0.66105400	-0.70358600
H	-0.76191200	-3.22335100	-2.98391600
H	-1.79409500	3.29954400	3.22937900
Sm	0.24606000	0.06502900	-0.41463300
N	4.02229900	-2.49003500	-1.80239600
C	3.26228700	-2.34167700	-3.03276600
H	-2.29056100	-1.44755600	2.81212900
H	-1.91305800	4.54809700	-2.10231000
C	5.15477100	-3.38522400	-1.89705600
I	0.72463000	-0.92477400	2.71226800
I	0.10428600	0.27255700	-3.62775800
N	5.04381700	-0.03212900	-0.79161800
C	4.62527500	1.18709800	-1.46305300
H	3.68492400	1.88077800	2.99577100
H	-4.74753300	4.27923400	0.73850000
C	6.43864600	-0.37044400	-0.98487900
N	0.74419900	4.83115300	0.71384100
C	-1.21902500	-4.15945900	-2.62607000
C	1.18208100	5.16734700	-0.62586400
H	-1.49398600	2.97608500	1.49852800
H	-0.84083400	-4.99384800	-3.23572100
N	4.41394800	-2.07206000	0.75489600
C	4.77929300	-1.28398900	1.92586200
H	1.13217800	-3.56458500	-1.23804900
H	-1.29999400	-2.96116200	4.38311300
C	3.71527300	-3.30238200	1.11434100
H	4.74912600	1.13005700	-2.56101400
H	3.57206200	1.39299900	-1.24078000
H	-2.78232600	-1.26239900	0.56147900
H	-2.34989800	2.08523300	-4.03531700
H	4.41873300	4.69078400	1.89886700
H	-3.90056100	-0.50352400	-3.53944600
H	6.69199800	-1.27455900	-0.41506000
H	6.69375700	-0.53954800	-2.04902300
H	-0.66481500	6.35249400	1.02794900
H	-0.03786600	5.56225500	2.50329300
H	-2.30674400	-4.08984600	-2.78365200
H	0.87481700	-4.68065600	0.12269100
H	1.04289200	-5.32371500	-1.53348900
C	0.23489400	5.92578100	1.50495400
H	2.04833200	5.85552500	-0.61708800
H	1.45536100	4.24981100	-1.16218200
H	5.25805500	-0.34920400	1.61206200
H	3.89399900	-1.05017700	2.54320200
C	-3.16708400	-2.06588700	2.56346400
H	-6.20798000	-0.37964200	-1.52379900
H	-1.87243200	-5.10323500	1.89494500
H	-4.72943000	-4.30884600	2.08366600
H	3.38363500	-3.82539400	0.20937600
H	4.40500400	-3.95970100	1.66903600
H	2.81806200	-3.31189400	-3.31291800
H	2.44999000	-1.61396600	-2.90365900
C	-3.27917800	-2.13169200	1.02391400
H	-4.32982900	2.38394200	2.32774700
H	1.83935900	3.13277400	4.05616000
H	-4.05597500	-1.61955700	3.03289900
H	5.87545200	-3.06497700	-2.67305100
H	5.67642300	-3.44453100	-0.93362400
H	-4.89531200	-3.92382500	3.80483800
C	0.63251100	-4.49629700	-0.93487400
O	-2.07571300	0.81949200	-0.25383300
P	-3.26136000	1.50492300	-0.93270200
N	-2.85544900	2.67076400	-2.06152200
C	-1.70038600	3.50975300	-1.79289900
H	3.52505300	4.50381500	3.43222200
N	0.23468500	3.16203400	2.69726100
C	-3.18309200	2.53765300	-3.46996100
H	-1.45963800	3.50826600	-0.72223500
H	-0.81346300	3.14394700	-2.33899900
O	-0.91449400	-2.10579100	-0.50652500
C	-1.42174100	-3.21090200	-0.36806300
H	-2.89528300	-3.13043400	5.16637100
C	-0.87505700	-4.38393700	-1.14979700
H	-4.07721100	1.91563200	-3.59649900

H	-3.40269500	3.53612400	-3.88512700
N	-4.12491100	2.34106800	0.24219400
C	-4.39394400	1.66352100	1.49512100
N	2.79371200	3.37939800	1.77601300
C	3.52909400	2.13894400	1.93229500
C	-4.93338100	3.50651900	-0.02785600
H	-5.39661500	1.19671000	1.51470500
H	-3.63631500	0.88697400	1.65843600
H	-0.96594100	-3.57325700	2.09166100
C	-4.20054800	-4.32286100	3.04914300
C	3.41223600	4.55805500	2.33176600
H	2.98047000	1.31554500	1.45861700
H	-4.66715600	3.91757800	-1.00965800
H	-6.01693300	3.27955200	-0.01873000
N	-4.26541900	0.37331700	-1.64034500
C	-3.69681500	-0.67708600	-2.46745600
H	4.51467100	2.22662200	1.44448000
H	-3.99260400	-5.37415300	3.30527600
C	-5.69383800	0.56233500	-1.77117900
H	-2.60689000	-0.71884800	-2.34153600
H	-4.13577900	-1.64717400	-2.18169900
C	-2.54820100	-3.43528700	0.60172800
C	-2.90713800	-3.50728000	3.02552800
C	-1.96521800	-4.00708000	1.91905500
H	2.82097300	5.45062500	2.09005200
H	-6.04351300	1.32826500	-1.06817400
H	-5.98160200	0.86949100	-2.79530300
O	0.98921700	2.27912600	0.24110900
P	1.18006100	3.33156700	1.32791500
H	-1.26326300	1.67016400	2.68194700
H	0.74508300	1.72327500	4.16825800
H	3.90428400	-2.00504900	-3.86763500
H	4.80580400	-4.39832000	-2.16258200
H	5.23743800	2.02652900	-1.09300700
H	7.07271400	0.45190000	-0.61364700
H	0.36221200	5.65618900	-1.17824100
H	5.49446000	-1.86212300	2.53380300
H	2.83481600	-3.08809300	1.74503300
H	0.97553000	6.74192300	1.61224100
C	-4.66038600	-2.12330000	0.56094800
N	-5.76488400	-2.12264900	0.19850300

TPPA-based reaction **Complex**
H_{corr} = 1.4290
$-TS$ = -0.2137
$E_{sol(THF)}$ = -28222.7439
$G_{sol(THF)}$ = -28221.5286

H	-0.62412300	3.03879500	-3.15103000
H	-0.11667300	4.75327900	-3.30080100
C	2.04301700	3.47624600	-1.42617900
C	2.58945500	3.75905800	-2.81784500
H	1.44018700	2.18589700	-3.77375400
H	3.53972500	3.23945700	-3.00529100
C	1.45297300	3.28649700	-3.71577500
C	0.20010600	3.74558200	-2.97751400
P	-3.80892400	-1.27176700	0.38793400
O	-2.30052300	-1.34697500	0.18724000
C	0.95154500	5.94929800	2.27975900
H	2.50680500	4.10265800	-0.64789000
Sm	-0.04851700	-0.53765300	-0.18122400
N	-4.26215000	-2.57424700	1.35736000
C	-3.35107200	-3.02957300	2.41151900
C	-3.95434700	-2.51856300	3.73221300
C	-5.40793500	-2.13038700	3.38797900
C	-5.59799400	-2.61208900	1.94431300
I	-0.69899600	-0.04460000	-3.36964400
I	0.11624600	-1.03454200	3.10161500
N	-4.19576000	0.25375800	0.96146100
C	-3.26154400	0.93665600	1.88025200
C	-4.16420900	1.87381500	2.67461000
C	-5.29608800	2.17823100	1.69783200
C	-5.53975000	0.82320400	1.04239700
N	0.06775000	4.27064400	0.94278000
H	2.76216000	4.84126100	-2.94248700
C	-0.36772000	3.92711700	2.30404500
H	2.19862200	2.41908700	-1.14708500
H	0.99308500	7.02514500	2.50137900
N	-4.80921800	-1.42577300	-0.94878800
C	-4.56181200	-0.42350000	-2.01833200
C	-4.61200300	-1.20732500	-3.32937100
C	-4.21183900	-2.61519300	-2.90566500
C	-4.92570500	-2.75984700	-1.57080700
H	-2.71302200	0.22811400	2.51922400
H	-2.50412800	1.49154200	1.30403100
H	-3.63121200	2.77010600	3.02268000
H	-4.56410100	1.35814500	3.56365100
H	-4.95482300	2.90460700	0.94191400
H	-6.20053400	2.58141900	2.17541300
H	-5.99796400	0.90181900	0.04560700
H	-6.21795500	0.21836200	1.67316100
H	1.30970500	5.76742700	0.11165500
H	-0.31136400	6.34459500	0.56029000
H	-1.00369500	5.80576500	3.19124100
H	0.24929400	4.97629800	4.13383300
H	1.95590500	5.52740200	2.44402300
C	0.50650500	5.65831800	0.85357500
H	-1.43559600	3.65398600	2.33206100
H	0.19429200	3.05414100	2.67187600
H	-5.31475000	0.38013200	-1.98002800
H	-3.56367100	0.02508800	-1.89973700
H	-3.93012400	-0.77293200	-4.07331100
H	-5.63447100	-1.20933700	-3.74232300
H	-3.12098800	-2.65684800	-2.75324300
H	-4.49682700	-3.39009500	-3.63206100
H	-4.50003700	-3.52781100	-0.91237700
H	-5.99497700	-2.99613900	-1.71785900
H	-3.33064700	-4.13291200	2.39048800
H	-2.33125400	-2.66450100	2.23393600
H	-3.38284400	-1.65404200	4.10066800
H	-3.90504900	-3.29036100	4.51363900
H	-5.53537200	-1.03767300	3.43599600
H	-6.14714400	-2.57444100	4.07072400
H	-6.30633000	-2.01617100	1.35197100
H	-5.95534700	-3.65750000	1.92915200
C	-0.08756000	5.19874100	3.11147200
H	1.50327000	3.68368800	-4.73916800
O	2.37065200	-0.23984800	-0.14838700
P	3.70679200	-0.03383000	0.55638800
N	3.74742300	0.96186000	1.89962900
C	3.15150900	2.30150300	1.81781300
C	3.25430000	2.80586600	3.24952200
C	2.97317300	1.54547100	4.05823900
C	3.69114300	0.44615500	3.28064500
H	3.70423100	2.92878100	1.10064100

H	2.09946500	2.25527900	1.48644300
H	2.54791200	3.62143500	3.45753600
H	4.27375700	3.17852700	3.44568200
H	1.89350100	1.32483200	4.05572100
H	3.31073600	1.60615700	5.10233200
H	3.12743200	-0.49492600	3.35098900
H	4.71576600	0.28206200	3.65953800
N	4.74328100	0.72894000	-0.50467600
C	4.63861100	0.48761300	-1.94890000
C	5.82482200	1.26293200	-2.53380600
C	6.17308500	2.28585400	-1.45345800
C	5.93233700	1.49926400	-0.17231900
H	4.69475600	-0.58617800	-2.19224900
H	3.66805200	0.85093100	-2.32152600
H	5.58591100	1.71734800	-3.50585100
H	6.67988000	0.58563600	-2.68911900
H	5.47801200	3.14047200	-1.49423000
H	7.19886700	2.67292600	-1.53340200
H	5.75476800	2.12863300	0.71044800
H	6.80390800	0.85211600	0.05010000
N	4.34803700	-1.50803300	1.00827600
C	3.51439300	-2.58567200	1.57065100
C	4.42214300	-3.22369500	2.61286100
C	5.80894300	-3.05740800	1.99798600
C	5.75155200	-1.65442600	1.39731800
H	2.57309200	-2.20204500	1.99414400
H	3.25592300	-3.30783900	0.77957300
H	4.15589200	-4.27029200	2.81777700
H	4.35736200	-2.66719500	3.56207200
H	5.95517800	-3.79986600	1.19611500
H	6.63281200	-3.17042600	2.71677700
H	6.41865000	-1.54313400	0.52754900
H	6.05106800	-0.89711300	2.14643500
O	-0.38503300	1.85113400	0.11277000
P	-0.42117000	3.30909700	-0.33199100
N	-1.98122100	3.69931200	-0.78073400
C	-2.85267100	2.72200700	-1.45886200
C	-3.68422000	3.58381200	-2.39850900
C	-3.80442900	4.90163700	-1.63890300
C	-2.41394900	5.07422000	-1.03213200
H	-2.26949000	1.95406800	-1.99006600
H	-3.48607500	2.20218300	-0.72189900
H	-4.65423300	3.12631300	-2.63927900
H	-3.14096200	3.73928700	-3.34502600
H	-4.55199100	4.80572000	-0.83389000
H	-4.09468000	5.75504600	-2.26787700
H	-2.43697900	5.66127400	-0.10019300
H	-1.73979600	5.59424500	-1.73869200
N	0.61083400	3.78839500	-1.55904400
O	0.58258800	-2.84665100	-0.87907300
C	0.89977300	-4.03333600	-0.84198800
H	2.50442300	-6.08667900	-4.00676300
C	0.00223200	-5.03888800	-0.15261700
H	3.93011500	-5.84603100	-2.96245200
H	2.49166900	-5.49033100	-1.08277300
C	3.21363900	-5.33557100	-3.62380100
H	0.25859800	-6.04067600	-0.53828000
C	0.32095300	-4.99345900	1.34727400
C	-1.46627500	-4.73777700	-0.42788400
H	0.11615100	-3.99212800	1.75988900
H	-0.29915500	-5.72475000	1.88794800
H	1.37758100	-5.23271500	1.54781400
H	-1.69299200	-4.80930000	-1.50291200
H	-2.10748100	-5.45518700	0.10669500
H	0.73654500	-4.16781900	-4.17827600
H	2.93898400	-2.45682700	-1.63674000
C	1.49303800	-3.47781100	-3.77066300
H	0.97264300	-2.67582900	-3.23255700
C	2.16268900	-4.50830000	-1.43042100
C	2.48831600	-4.21736200	-2.91475800
C	3.21483400	-3.49546900	-1.83626700
H	4.25502200	-3.75989000	-1.63198800
H	2.01438500	-3.01473200	-4.62360500
H	3.77372300	-4.93659300	-4.48526700
H	-1.72568100	-3.72169400	-0.09827500

TPPA-based reaction **Int I**

H_{corr} = 1.4273

$-TS$ = -0.2125

$E_{sol(THF)}$ = -28222.7334

$G_{sol(THF)}$ = -28221.5185

H	0.31302600	2.76845700	3.38679800
H	-0.16282200	4.49138300	3.54156600
C	-2.16050900	3.34474100	1.42295600
C	-2.83097000	3.60276600	2.76367900
H	-1.83323900	1.96067000	3.76699500
H	-3.81077000	3.10954300	2.83447400
C	-1.80480600	3.06216700	3.75172100
C	-0.47170200	3.50304200	3.15660000
P	3.77687700	-1.03396000	-0.35933600
O	2.24770000	-0.99335300	-0.31627600
C	-1.14085900	5.57313100	-2.09037500
H	-2.52051900	4.01701700	0.62979400
Sm	0.01714400	-0.59815800	0.01431500
N	4.21470200	-2.18126700	-1.50280000
C	3.39782300	-2.35423700	-2.70808400
C	4.19113600	-1.67878700	-3.83875600
C	5.63546900	-1.56177500	-3.30486600
C	5.60254600	-2.27708300	-1.94740100
I	0.25870200	-0.31420000	3.15504500
I	-0.08332200	-0.13639800	-3.14701600
N	4.31384500	0.52852400	-0.61110300
C	3.53309800	1.42919000	-1.48489200
C	4.57679600	2.40537000	-2.01567000
C	5.62057500	2.44051200	-0.90307000
C	5.69871800	0.97904500	-0.47691700
N	-0.01493600	4.06176300	-0.74356200
H	-2.98171300	4.68511600	2.91215500
C	0.50040100	3.74994700	-2.07390000
H	-2.32463800	2.30508000	1.09405400
H	-1.13318100	6.60394100	-2.47233400
N	4.59686300	-1.50219300	1.01723300
C	4.28480900	-0.69404600	2.22595600
C	4.10602800	-1.70850400	3.35757300
C	3.71474600	-2.98834600	2.62836100
C	4.59446000	-2.93167900	1.39035300
H	3.01184800	0.88247200	-2.28536600
H	2.75830500	1.93732200	-0.89322800
H	4.14540800	3.39019700	-2.24417800
H	5.03063500	2.01541900	-2.94206500
H	5.25692500	3.05564800	-0.06363000
H	6.59528000	2.83755600	-1.22013100
H	6.05882500	0.84692000	0.55374200
H	6.38815300	0.42930700	-1.14467100

H	-1.06459400	5.67329800	0.12520200
H	0.44240200	6.13749200	-0.70744100
H	0.20635700	5.09797600	-3.76775400
H	-1.09999000	3.92908200	-3.54485400
H	-2.19258500	5.27152800	-1.97167200
C	-0.42949400	5.45374800	-0.74313800
H	1.55700600	4.06903400	-2.16788500
H	0.44692700	2.66868800	-2.26267700
H	5.09352300	0.02587700	2.43023600
H	3.34921900	-0.13407800	2.07798900
H	3.34204700	-1.37155700	4.07153000
H	5.05515100	-1.85362300	3.89921000
H	2.65419900	-2.94428600	2.33308600
H	3.87058400	-3.89811200	3.22548100
H	4.23572200	-3.54615300	0.55560300
H	5.62917200	-3.23997900	1.62329600
H	3.29168100	-3.43639900	-2.89034200
H	2.39261700	-1.93753700	-2.56839700
H	3.76893300	-0.68761000	-4.06109200
H	4.13442000	-2.26483600	-4.76685300
H	5.91114900	-0.50462200	-3.16721400
H	6.38128300	-2.00540900	-3.98029100
H	6.28851700	-1.86196100	-1.19587200
H	5.85285000	-3.34603300	-2.06586800
C	-0.39637600	4.58083700	-3.00788800
H	-1.94392300	3.42579700	4.77937900
O	-2.18965500	0.01948500	-0.03527500
P	-3.64780000	-0.00390900	-0.50972300
N	-4.02567600	1.03141600	-1.76321500
C	-3.61294300	2.43865400	-1.70290200
C	-3.98981500	2.95902400	-3.08183100
C	-3.64063700	1.77485600	-3.97565200
C	-4.09766000	0.56683700	-3.16274800
H	-4.13623600	2.96157800	-0.88707500
H	-2.52616300	2.53126700	-1.52743700
H	-3.45258900	3.88079600	-3.34600000
H	-5.07115400	3.17143900	-3.12100400
H	-2.54873900	1.71644300	-4.11224800
H	-4.11351300	1.81426800	-4.96677400
H	-3.44446000	-0.29659200	-3.34888600
H	-5.13599600	0.28113600	-3.40641200
N	-4.59792900	0.53329000	0.74709100
C	-4.34819800	0.00468000	2.09931800
C	-5.71298200	0.09614400	2.77428800
C	-6.37130400	1.27944000	2.06998800
C	-5.94349100	1.09186400	0.61828500
H	-3.95924000	-1.02383400	2.06424600
H	-3.58999600	0.61673900	2.61501200
H	-5.63521200	0.21947500	3.86328300
H	-6.29218400	-0.82193900	2.58114200
H	-5.96425000	2.22699100	2.45980800
H	-7.46398300	1.31023900	2.18431100
H	-5.92970600	2.03516000	0.05253300
H	-6.63461200	0.40428800	0.09745300
N	-4.08958000	-1.53643700	-0.95727200
C	-3.18879400	-2.46087500	-1.67580100
C	-4.15541500	-3.35432300	-2.43916600
C	-5.35798200	-3.42398700	-1.50286500
C	-5.47974000	-1.98759800	-1.00222800
H	-2.48520500	-1.92188900	-2.32855300
H	-2.59082400	-3.04059700	-0.95898800
H	-3.71746900	-4.33715100	-2.66203300
H	-4.44628500	-2.88493000	-3.39401300
H	-5.13138400	-4.09134600	-0.65577200
H	-6.28066800	-3.77813500	-1.98377200
H	-5.95016100	-1.92841600	-0.00809600
H	-6.08792500	-1.38046200	-1.70080600
O	0.51277400	1.68776900	0.15321400
P	0.41437500	3.15050600	0.58330000
N	1.90536900	3.60222800	1.18578900
C	2.74052100	2.65934200	1.95324200
C	3.40704000	3.54655900	2.99557500
C	3.54884400	4.88083100	2.26876300
C	2.23390900	4.99327300	1.50126200
H	2.13813500	1.85018200	2.39354400
H	3.48366800	2.18811900	1.28936600
H	4.36387700	3.13545600	3.34734700
H	2.74676300	3.66164300	3.87073400
H	4.39147100	4.83582900	1.55890200
H	3.71758300	5.73704300	2.93681600
H	2.33697200	5.59531600	0.58473900
H	1.45342400	5.46669700	2.12595300
N	-0.73715500	3.59512900	1.70699000
O	-0.41753700	-2.67916300	0.10590000
C	-0.69862300	-3.97558300	0.00403300
H	-1.38839500	-6.85458300	2.97112200
C	0.20534000	-4.81877700	-0.85244100
H	-3.02297900	-6.32497600	2.50336400
H	-2.25525300	-5.41859100	0.43458300
C	-2.10154500	-6.01423200	3.02105800
H	-0.13705700	-5.86488700	-0.75865600
C	0.11567500	-4.40842200	-2.32500900
C	1.65495600	-4.74184800	-0.36013300
H	0.34347600	-3.33756800	-2.44452300
H	0.82086700	-4.98191400	-2.94988600
H	-0.89928600	-4.57174900	-2.72061400
H	1.74157600	-5.10040300	0.67735900
H	2.33098000	-5.34314100	-0.99156900
H	0.53981000	-5.09690300	2.97211300
H	-2.16388500	-2.69411900	1.93817200
C	-0.22408600	-4.30194200	3.02479800
H	0.16487000	-3.41233800	2.51272900
C	-1.70487600	-4.58582600	0.89189800
C	-1.51613400	-4.76764700	2.40835600
C	-2.47433200	-3.73999600	1.87481500
H	-3.54705600	-3.91826800	1.99349600
H	-0.36338000	-4.03641700	4.08574000
H	-2.35317300	-5.85472600	4.08397600
H	1.99842800	-3.69689700	-0.38568700

TPPA-based reaction **TS I**

H_{corr} = 1.4253

$-TS$ = -0.2126

$E_{sol(THF)}$ = -28222.7209

$G_{sol(THF)}$ = -28221.5082

H	-0.47043100	2.78800500	-3.40824100
H	-0.10802100	4.54185600	-3.50189500
C	1.94910900	3.44858500	-1.40667300
C	2.61044600	3.79770300	-2.73147900
H	1.72647700	2.13675700	-3.80423900
H	3.61899100	3.36899400	-2.81570900
C	1.62582800	3.23249700	-3.74761900
C	0.26332700	3.56358600	-3.14746200
P	-3.64937200	-1.29679000	0.26960200

O	-2.12296700	-1.17537700	0.27249100
C	0.66710800	5.72336300	2.07920500
H	2.26320100	4.11167100	-0.58612000
Sm	0.07016900	-0.57125100	0.03019500
N	-4.05242400	-2.45473200	1.41561800
C	-3.26394700	-2.56220000	2.64968600
C	-4.17006000	-2.02044400	3.77344600
C	-5.56745000	-1.88068500	3.13714400
C	-5.44644200	-2.63484500	1.81093400
I	-0.11140400	-0.29806600	-3.11213500
I	0.01202600	-0.09651100	3.16911200
N	-4.27979400	0.23568500	0.49205400
C	-3.62469900	1.14580300	1.44715100
C	-4.51228000	2.38052400	1.40101700
C	-5.90553900	1.79359700	1.19319100
C	-5.66251300	0.64119400	0.21972000
N	-0.31883500	4.13418300	0.72200000
H	2.69491100	4.89215100	-2.83768700
C	-0.78490400	3.77716100	2.07032000
H	2.17140800	2.41025600	-1.11024600
H	0.76289400	6.78361700	2.35357200
N	-4.40107900	-1.82915300	-1.12221800
C	-4.12255000	-1.01449000	-2.33509500
C	-3.83304300	-2.02595900	-3.44679200
C	-3.36757800	-3.26413800	-2.68992700
C	-4.28154700	-3.25918400	-1.47586200
H	-3.60235600	0.71742900	2.46607600
H	-2.58812300	1.34108900	1.14528600
H	-4.22044100	3.01265900	0.54685400
H	-4.42896100	2.98656600	2.31419400
H	-6.63928800	2.51602800	0.80807600
H	-6.29077000	1.40303900	2.14937000
H	-5.75791300	0.98413600	-0.82663000
H	-6.37950000	-0.18135900	0.35666800
H	0.86330900	5.66682600	-0.12311600
H	-0.71739800	6.21961100	0.48122000
H	-1.20547000	5.61628700	3.15865200
H	0.07138400	4.63745300	3.90210700
H	1.66212800	5.25633400	2.15333100
C	0.12117100	5.52059900	0.67288900
H	-1.88037200	3.65104200	2.09983500
H	-0.34066100	2.81976600	2.38273500
H	-4.98227200	-0.36766700	-2.57013900
H	-3.23941100	-0.37881600	-2.17180300
H	-3.07639500	-1.64026900	-4.14360100
H	-4.75179300	-2.24905400	-4.01361300
H	-2.32169700	-3.13837300	-2.36802600
H	-3.43921000	-4.18997300	-3.27830100
H	-3.89987300	-3.83505600	-0.62371900
H	-5.28379500	-3.64725600	-1.73027000
H	-3.02854500	-3.62644800	2.81159200
H	-2.31319100	-2.02205600	2.55691100
H	-3.79739900	-1.05452800	4.14409400
H	-4.17968300	-2.70949000	4.63010100
H	-5.79509600	-0.82196300	2.93682200
H	-6.37417600	-2.27567700	3.77155200
H	-6.13091200	-2.29183300	1.02297000
H	-5.62845000	-3.71454400	1.95810900
C	-0.34439700	4.96530400	2.93943600
H	1.74751700	3.64492000	-4.75897600
O	2.21489400	0.24250600	0.03066400
P	3.65266500	0.23098200	0.57369900
N	3.81715700	1.34784000	1.79497100
C	2.85083400	2.43932400	1.97109400
C	2.87737000	2.74591400	3.47863300
C	3.63333000	1.57411200	4.10530400
C	4.60869700	1.17389600	3.00726200
H	3.14449600	3.32324900	1.37987600
H	1.85914600	2.11239100	1.63060500
H	1.85973100	2.83717000	3.88278500
H	3.40860800	3.69254300	3.66764500
H	2.93275600	0.74543400	4.29182600
H	4.13250700	1.83641100	5.04924800
H	4.97590600	0.14217400	3.08099100
H	5.48861700	1.84640300	2.99572700
N	4.64691500	0.64296500	-0.69427900
C	4.32801600	0.28800300	-2.08427300
C	5.59250300	0.68862600	-2.84581900
C	6.22202900	1.77354700	-1.97355000
C	5.95938900	1.25808200	-0.56379500
H	4.09779700	-0.78319700	-2.19903800
H	3.43686800	0.83853300	-2.42531600
H	5.37168100	1.02195500	-3.86939000
H	6.27775900	-0.17159800	-2.91625900
H	5.70032500	2.73372400	-2.11732400
H	7.29048100	1.93263400	-2.17640100
H	5.93998100	2.05230900	0.19761000
H	6.73697000	0.52568100	-0.26932200
N	4.19775200	-1.21100800	1.18446600
C	3.41658300	-1.97317000	2.17246700
C	4.35909300	-3.11276800	2.52315600
C	5.00416300	-3.42582200	1.17635300
C	5.22095200	-2.04982600	0.54562100
H	3.14045000	-1.34360300	3.03038600
H	2.48704200	-2.35573000	1.72196200
H	3.82686000	-3.97099300	2.95598600
H	5.11632000	-2.77379800	3.25050200
H	4.29317500	-4.00044000	0.56448600
H	5.94046500	-3.99616100	1.25560900
H	5.10879600	-2.08336400	-0.54829500
H	6.22943400	-1.65886100	0.77062300
O	-0.60405100	1.68513900	-0.08881000
P	-0.62012700	3.13740200	-0.57311700
N	-2.12936000	3.44532300	-1.21938900
C	-2.85145200	2.42893500	-2.00599900
C	-3.60673300	3.25359800	-3.03992700
C	-3.88180500	4.56134000	-2.30245400
C	-2.58239000	4.79965300	-1.53807600
H	-2.16225300	1.69626600	-2.45381700
H	-3.53933900	1.86870800	-1.35229200
H	-4.51743400	2.75040700	-3.39456800
H	-2.96386900	3.44319100	-3.91520500
H	-4.71331300	4.42572500	-1.59089200
H	-4.13818100	5.40057800	-2.96414200
H	-2.73550100	5.39155300	-0.62190400
H	-1.85179900	5.34127000	-2.16832900
N	0.51610300	3.62314000	-1.69496200
O	0.71984600	-2.60255900	-0.00083700
C	1.08998200	-3.87921200	0.10993800
H	1.72470700	-6.68618000	-3.22627100
C	0.25256900	-4.74785300	1.01634800
H	3.30028700	-6.23140700	-2.52760800
H	2.48004300	-5.40583900	-0.44572200
C	2.46420300	-5.87101800	-3.14977200

H	0.68166300	-5.76435700	0.97697200
C	0.27746400	-4.26270700	2.46473600
C	-1.18441900	-4.81440300	0.48886200
H	-0.05042900	-3.21426900	2.53771100
H	-0.38481100	-4.87271300	3.10106400
H	1.29381800	-4.31827400	2.88523000
H	-1.20833800	-5.21664600	-0.53596400
H	-1.81842700	-5.45274500	1.12710700
H	-0.17851800	-5.02602400	-3.15330600
H	2.42585200	-2.62914400	-1.88076500
C	0.53018900	-4.18099800	-3.10281800
H	0.09978600	-3.40285800	-2.45779100
C	2.08710300	-4.41986000	-0.70424000
C	1.86214700	-4.62057200	-2.60082200
C	2.72464000	-3.68361600	-1.83479800
H	3.81005100	-3.80714300	-1.96082500
H	0.59371400	-3.74435800	-4.11893500
H	2.87326400	-5.71366500	-4.16978700
H	-1.62251000	-3.80527100	0.46727200

TPPA-based reaction **Int II**

H_{corr} = 1.4268

$-TS$ = -0.2135

$E_{sol(THF)}$ = -28222.7411

$G_{sol(THF)}$ = -28221.5278

H	0.21618000	2.55470500	3.38192500
H	-0.25483800	4.26512700	3.64833800
C	-2.16561400	3.30811000	1.36403300
C	-2.89064400	3.47126500	2.69088400
H	-1.95112100	1.74136700	3.60166900
H	-3.87808000	2.98854700	2.67991300
C	-1.91368200	2.84042900	3.67518700
C	-0.55289200	3.31274700	3.17490500
P	3.83958200	-1.09988100	-0.28069100
O	2.31018500	-1.04604400	-0.27512600
C	-0.98321800	5.61719100	-2.02714500
H	-2.48676200	4.04057900	0.60835800
Sm	0.06887600	-0.63031500	-0.05273300
N	4.29902000	-2.25554900	-1.40807800
C	3.48297300	-2.46997700	-2.60841400
C	4.26932700	-1.82667700	-3.76574300
C	5.68636000	-1.58251000	-3.20587800
C	5.68843100	-2.30691700	-1.85584900
I	0.21264300	-0.53254300	3.09504600
I	0.11598400	-0.18619100	-3.20479100
N	4.39463500	0.45639600	-0.52774500
C	3.63073200	1.36805500	-1.40421900
C	4.66814700	2.39899200	-1.83913300
C	5.67547000	2.39643900	-0.69255300
C	5.76887700	0.91670700	-0.34045700
N	0.05806600	4.04719300	-0.68604800
H	-3.03504700	4.54078200	2.91767900
C	0.60363000	3.74521000	-2.00831300
H	-2.32454100	2.29679700	0.95490400
H	-0.96869200	6.66001600	-2.37459000
N	4.62011800	-1.56553100	1.11916300
C	4.27777600	-0.75394700	2.31687900
C	4.11457500	-1.76289700	3.45437700
C	3.73200300	-3.04867300	2.73126000
C	4.61606700	-2.99421700	1.49602500
H	3.17320700	0.84028300	-2.25524100
H	2.80744500	1.82366600	-0.83538800
H	4.21909600	3.38477700	-2.02547200
H	5.16252200	2.07613100	-2.77037400
H	5.27432500	2.95997500	0.16574000
H	6.65095800	2.82742800	-0.95887800
H	6.10917500	0.73414900	0.68891800
H	6.48001300	0.41293700	-1.02235200
H	-0.97304700	5.66683500	0.19007200
H	0.57088200	6.10969500	-0.58610700
H	0.46980000	5.20739200	-3.62495900
H	-0.88081500	4.06959200	-3.58123200
H	-2.03608100	5.30523000	-1.95245700
C	-0.31723100	5.45057600	-0.66377600
H	1.68069800	3.99721400	-2.05499800
H	0.49359800	2.67504600	-2.23248300
H	5.06537400	-0.01108600	2.52177400
H	3.32819100	-0.22189800	2.15594900
H	3.35080700	-1.43000600	4.17040800
H	5.06762400	-1.89628800	3.99226300
H	2.67121600	-3.00922700	2.43473700
H	3.89245700	-3.95430100	3.33342400
H	4.26139700	-3.61081700	0.66122800
H	5.64972900	-3.30253800	1.73353300
H	3.37893300	-3.55740900	-2.75656100
H	2.47738300	-2.05087900	-2.48041200
H	3.79357100	-0.88561100	-4.07807000
H	4.28135400	-2.48630500	-4.64504200
H	5.85617600	-0.50578500	-3.04776500
H	6.48330100	-1.94460700	-3.87151700
H	6.36348800	-1.87335600	-1.10460300
H	5.97346400	-3.36645300	-1.98164200
C	-0.20256800	4.65994400	-2.94922900
H	-2.09401000	3.12135800	4.72211100
O	-2.13049900	0.00674400	-0.18917300
P	-3.57412100	0.02260200	-0.70750400
N	-3.89984100	1.08349800	-1.95631900
C	-3.50910000	2.49420700	-1.84006800
C	-3.78472600	3.03456400	-3.23477600
C	-3.33973500	1.87462900	-4.11768700
C	-3.83389900	0.64212900	-3.36528500
H	-4.09760000	2.99697500	-1.05703600
H	-2.43959100	2.59690300	-1.58256300
H	-3.24793500	3.97203700	-3.43767900
H	-4.86372100	3.22594000	-3.35688200
H	-2.23945300	1.83989900	-4.16907000
H	-3.73218100	1.92280400	-5.14288300
H	-3.14220000	-0.20013600	-3.50572400
H	-4.83861400	0.33393700	-3.70331600
N	-4.55432900	0.56354500	0.52600500
C	-4.37999100	-0.04420900	1.85777800
C	-5.78911000	-0.04908600	2.43684900
C	-6.43069400	1.17281600	1.78563200
C	-5.89869600	1.11925100	0.35636000
H	-3.94660900	-1.05088900	1.78356300
H	-3.68791400	0.56084000	2.46629500
H	-5.79218100	-0.01808100	3.53501200
H	-6.32080800	-0.96380900	2.12627800
H	-6.07988000	2.09287600	2.28174500
H	-7.52914700	1.17016700	1.82231000
H	-5.86229200	2.11136600	-0.11835900
H	-6.54035200	0.47685500	-0.27221300
N	-4.02564300	-1.49647800	-1.19417600
C	-3.10831000	-2.44706400	-1.85692500

C	-4.03182800	-3.21760000	-2.78807100
C	-5.33249700	-3.27353400	-1.99307400
C	-5.42661400	-1.87350800	-1.39078600
H	-2.29402400	-1.92755300	-2.38307200
H	-2.65586900	-3.11513100	-1.11032900
H	-3.63112200	-4.20967200	-3.03833700
H	-4.18436600	-2.66225300	-3.72869300
H	-5.24272800	-4.01831800	-1.18605100
H	-6.21603100	-3.52702000	-2.59564600
H	-5.97883700	-1.86737300	-0.43761100
H	-5.94183600	-1.18121900	-2.08400900
O	0.55801700	1.65394200	0.17119900
P	0.44081000	3.10606000	0.63423300
N	1.90715800	3.52990200	1.31199500
C	2.71050900	2.53954900	2.05459700
C	3.33113600	3.35862600	3.17792300
C	3.49664800	4.73746000	2.54552300
C	2.21225600	4.89733400	1.73618000
H	2.09074600	1.70546500	2.41798900
H	3.48135200	2.10909700	1.39486600
H	4.27457300	2.92668500	3.54069800
H	2.63584700	3.41546100	4.03134600
H	4.36639700	4.73962200	1.86763100
H	3.63571900	5.54857400	3.27393400
H	2.34663200	5.56218800	0.86857200
H	1.40386700	5.32171700	2.36048200
N	-0.75247500	3.52044000	1.72522700
O	-0.39557400	-2.70746700	-0.01220800
C	-0.68682700	-3.99605000	0.09774300
H	-3.90654500	-3.71912500	4.87811300
C	0.22024500	-4.91585200	-0.69202600
H	-4.67824200	-3.14684200	3.37171300
H	-1.88916000	-5.54011200	0.86391000
C	-3.72259100	-3.20375800	3.92054600
H	-0.12020200	-5.94987000	-0.51409800
C	0.12104700	-4.61273100	-2.18636700
C	1.65973200	-4.77881800	-0.19845500
H	0.37092000	-3.55945200	-2.38815000
H	0.80926200	-5.24830800	-2.76704600
H	-0.90020900	-4.78959600	-2.55927600
H	1.73817700	-5.04463500	0.86736400
H	2.34040300	-5.43141900	-0.76939300
H	-1.64540500	-4.81311900	4.77231300
H	-2.28951000	-2.53282400	1.49600000
C	-1.41929200	-4.29910800	3.82279100
H	-0.80524500	-4.96110200	3.19386600
C	-1.72151500	-4.45955900	0.83587900
C	-2.66058300	-3.87535700	3.11905900
C	-2.62052800	-3.58182700	1.64833100
H	-3.66016200	-3.61840200	1.25974700
H	-0.78485900	-3.42128100	4.06806000
H	-3.43502300	-2.16055300	4.17725600
H	1.99970200	-3.73909700	-0.31382100

TPPA-based reaction **PreTS II** with phenylacetylene

H_{corr} = 1.5485

$-TS$ = -0.2312

$E_{sol(THF)}$ = -28531.0016

$G_{sol(THF)}$ = -28529.6843

H	0.87010300	3.73373600	0.27028000
H	1.49870400	4.81864400	1.55256700
C	2.30244500	1.96993000	2.54710400
C	3.49463200	2.77046000	2.04771200
H	2.90481600	2.63450900	-0.03302100
H	4.37884000	2.13662500	1.89079700
C	2.95406300	3.39062300	0.76685600
C	1.53429000	3.79268800	1.14474000
P	-4.09362900	0.13913000	-1.29912200
O	-2.66828600	-0.31952200	-0.98106900
C	-0.05256800	1.24296200	6.03085900
H	2.33302200	1.78103600	3.63074700
Sm	-0.48476700	-0.62491400	-0.36032200
N	-4.98739900	-1.22712700	-1.69059600
C	-4.69610000	-2.50840000	-1.04042300
C	-5.81950800	-2.71034800	-0.00925200
C	-6.93375900	-1.72728400	-0.42852200
C	-6.44382700	-1.14026900	-1.75818800
I	0.43549800	1.82124900	-2.12117100
I	-1.74219100	-2.68388700	1.72170700
N	-4.62893500	1.04946600	-0.00429700
C	-4.19592300	0.69713000	1.36326400
C	-5.27510500	1.31120100	2.25025800
C	-5.80170300	2.47621000	1.41691600
C	-5.82569700	1.88813300	0.01155300
N	-0.50215900	1.62137400	3.79627300
H	3.76090500	3.55423900	2.77653900
C	-1.53304600	0.65152400	4.16554600
H	2.24610000	0.99899500	2.02819400
H	-0.16983700	1.61727000	7.05776500
N	-4.33586600	1.15519200	-2.60024300
C	-3.57116400	2.42855700	-2.55497400
C	-3.07316300	2.64800900	-3.98388600
C	-3.00982500	1.23623900	-4.55431600
C	-4.25798900	0.59887700	-3.96652600
H	-4.10124900	-0.39070000	1.50256600
H	-3.20372100	1.12847300	1.55972000
H	-4.88203900	1.61589600	3.23047600
H	-6.08472900	0.58377700	2.42683800
H	-5.09495800	3.32141200	1.45629300
H	-6.78823500	2.83999000	1.73740000
H	-5.78461400	2.64930000	-0.78058600
H	-6.75367600	1.30305400	-0.13396100
H	0.81429200	2.89998000	4.83981300
H	-0.90407100	3.09664300	5.27791300
H	-2.04459100	0.35011600	6.26678600
H	-0.82663000	-0.77910100	5.66574600
H	0.93257800	0.75679800	5.96230400
C	-0.12840800	2.35769100	4.99233900
H	-2.53787800	1.11488200	4.13457700
H	-1.52911700	-0.19549500	3.46516300
H	-4.20942400	3.25381600	-2.20026200
H	-2.71169600	2.33225300	-1.87472100
H	-2.09907000	3.15588400	-3.98830700
H	-3.79205700	3.25902300	-4.55437900
H	-2.11221100	0.72160600	-4.17440900
H	-2.99142200	1.20790100	-5.65316400
H	-4.23025500	-0.49705300	-3.93497500
H	-5.16057000	0.90136700	-4.52695900
H	-4.72422500	-3.29280100	-1.81501800
H	-3.69289200	-2.50844300	-0.59659300
H	-5.45121700	-2.48844000	1.00317000
H	-6.16566200	-3.75359300	-0.00183700
H	-7.03906000	-0.92442400	0.31786100
H	-7.91775400	-2.20759600	-0.53034300

H	-6.77093700	-0.10888900	-1.95064100
H	-6.78781500	-1.75790500	-2.60656100
C	-1.16624500	0.26465500	5.61121100
H	3.54652100	4.23665000	0.39588500
O	1.52931300	-1.13000700	0.62913800
P	2.61954500	-1.98898500	1.28139700
N	2.42354600	-2.38797700	2.89155200
C	2.21784900	-1.33221100	3.89188300
C	1.91182200	-2.12400500	5.15314000
C	1.04304100	-3.25358300	4.61261800
C	1.71239300	-3.62089100	3.29104400
H	3.11456200	-0.70057000	3.98093200
H	1.36661600	-0.68123200	3.62345000
H	1.41480400	-1.51642700	5.92253800
H	2.84632000	-2.52145200	5.58269100
H	0.02694000	-2.88259900	4.40187900
H	0.96144200	-4.11376200	5.29137800
H	0.96023300	-3.92520900	2.54932700
H	2.43931700	-4.44153900	3.41984400
N	4.02780000	-1.10009100	1.28113100
C	4.39412200	-0.39585900	0.03736800
C	5.90888100	-0.53172700	-0.01892800
C	6.29890500	-0.54365100	1.45571200
C	5.21022900	-1.40275700	2.09334000
H	3.89035200	-0.83265000	-0.83545000
H	4.08295400	0.65942500	0.08556200
H	6.37526600	0.27511400	-0.59944700
H	6.18252200	-1.49077400	-0.49017900
H	6.25607500	0.47980000	1.86403600
H	7.30630500	-0.93916600	1.64754100
H	5.04127900	-1.15164800	3.15169000
H	5.48399200	-2.47092200	2.04893900
N	2.79278500	-3.41092000	0.43293500
C	1.66402100	-4.08993200	-0.23663800
C	1.94087600	-5.56240600	0.02711800
C	3.46474000	-5.63171600	0.00672500
C	3.88862300	-4.34489000	0.71515000
H	0.69701400	-3.75817700	0.16562700
H	1.67440500	-3.86249500	-1.31422300
H	1.46679700	-6.21385600	-0.72015600
H	1.55698200	-5.84989000	1.01966800
H	3.82567100	-5.61818100	-1.03335200
H	3.87612300	-6.52607600	0.49556000
H	4.84702600	-3.96151200	0.32903500
H	4.00612900	-4.50904100	1.80206000
O	-0.72722500	1.02591500	1.28510300
P	-0.38354200	2.17936600	2.22907800
N	-1.45757500	3.41895500	1.91034700
C	-1.98327400	3.63469200	0.54849500
C	-2.02692700	5.15058700	0.42200800
C	-2.32738900	5.60181900	1.84808600
C	-1.47547000	4.65730200	2.69314400
H	-1.34692700	3.15594100	-0.21090300
H	-2.99021900	3.19479800	0.45825300
H	-2.77228600	5.48970900	-0.31130100
H	-1.04204100	5.52951300	0.10324700
H	-3.39496400	5.44416000	2.07508200
H	-2.09513500	6.65859400	2.04105000
H	-1.90807900	4.49147600	3.69229400
H	-0.45638500	5.06238800	2.83334000
N	1.15307700	2.82702800	2.19970700
O	-0.10666400	-1.96038400	-1.97318700
C	0.11915000	-2.63839900	-3.08941000
H	5.37426500	-3.29163600	-2.91165900
C	-1.01767200	-3.54287200	-3.51980900
H	3.86301300	-4.20418000	-3.09199700
H	1.33894700	-3.10062800	-4.73467900
C	4.27691000	-3.20478300	-2.83818200
H	-0.68386900	-4.09007000	-4.41761500
C	-1.36038900	-4.55092600	-2.42500700
C	-2.23725400	-2.69693900	-3.88554300
H	-1.62907500	-4.03533700	-1.48975500
H	-2.21016200	-5.18559200	-2.72413200
H	-0.50437400	-5.20996900	-2.21059400
H	-2.00556300	-2.01249800	-4.71649800
H	-3.08807500	-3.33093800	-4.18487800
H	5.33901900	-2.26542400	-5.19338600
H	2.25934100	-0.62478200	-3.99576800
C	4.25089100	-2.09399900	-5.14321300
H	3.78193900	-2.87283700	-5.78239500
C	1.25646000	-2.52093000	-3.81093100
C	3.73690900	-2.15206400	-3.74438400
C	2.38086100	-1.58240800	-3.45485600
H	2.28767700	-1.33195200	-2.38471600
H	4.02602800	-1.12564000	-5.61925700
H	4.00140800	-3.01116500	-1.78978500
H	-2.54679000	-2.08880400	-3.02278400
C	4.79312200	0.97851400	-3.04370700
C	3.46806700	5.59334200	-2.18553100
C	4.77094700	2.14435000	-2.70392100
H	4.75402700	-0.06292000	-3.32306800
H	6.65207100	3.52737800	-1.38437900
H	6.49548300	5.90121000	-0.65755000
H	4.45270100	7.22840400	-1.17156200
H	2.56652800	6.16742000	-2.40862700
H	2.71979700	3.78662700	-3.11107200
C	3.55085400	4.26653700	-2.59321600
C	4.70130100	3.51328200	-2.30611000
C	5.75962100	4.11468000	-1.60656400
C	5.66715300	5.44253800	-1.20136100
C	4.52237000	6.18616300	-1.48975700

TPPA-based reaction **TS II** with phenylacetylene

H_{corr} = 1.5472

$-TS$ = -0.2285

$E_{sol(THF)}$ = -28530.9945

$G_{sol(THF)}$ = -28529.6758

H	0.09591200	2.21365000	3.36813300
H	-0.17282700	3.93689100	3.78921800
C	-1.59532100	3.64182200	1.02910300
C	-2.58126700	3.76276900	2.18231700
H	-2.16070100	1.78388200	2.95934400
H	-3.60164700	3.48255900	1.88756900
C	-1.97631900	2.83625200	3.22821900
C	-0.48271700	3.11494900	3.11970000
P	3.90260200	-1.71914500	0.45026000
O	2.46658200	-1.37578400	0.04569800
C	2.01445600	6.14888300	-1.11549700
H	-1.60967800	4.51948700	0.36649300
Sm	0.34884600	-0.55809900	-0.32431100
N	4.48409100	-2.86144500	-0.61428300
C	4.06147800	-2.84534000	-2.02585600
C	5.36941600	-3.01733100	-2.78042300
C	6.13782000	-3.97886400	-1.87875700

C	5.80490400	-3.48859900	-0.46741300
I	-0.22489900	-0.78462300	2.76872600
I	1.52283800	0.08828000	-3.21665100
N	4.74518100	-0.26907500	0.54165700
C	4.43757100	0.83509300	-0.37254400
C	5.36703300	1.96890100	0.10277500
C	6.32782100	1.32117900	1.11835600
C	6.15745800	-0.17613600	0.88102500
N	1.15350600	4.07938000	-0.39196700
H	-2.60576300	4.80139400	2.55179900
C	2.13408200	3.78996900	-1.45587600
H	-1.80840100	2.74761400	0.42044500
H	2.92774200	6.25100300	-0.50520400
N	4.18721000	-2.38960500	1.95032400
C	3.78129200	-1.56328900	3.11572700
C	3.20014200	-2.55712500	4.12096000
C	2.69354300	-3.69156500	3.23810200
C	3.78882700	-3.79105200	2.18935200
H	4.65411300	0.56592900	-1.42240700
H	3.37282700	1.08890700	-0.31042700
H	4.78669200	2.78021000	0.56556400
H	5.91189700	2.40040000	-0.74911300
H	6.02161700	1.56167400	2.14809000
H	7.37046800	1.64715100	0.99583200
H	6.39007300	-0.80143500	1.75412200
H	6.80373000	-0.50137000	0.04025800
H	-0.08574000	5.76267700	-0.72186400
H	0.88552000	5.85610700	0.76091600
H	3.19440800	5.21536000	-2.73496400
H	1.43324700	5.17663100	-2.96357400
H	1.74766700	7.14548700	-1.49490500
C	0.90109500	5.52029800	-0.28538000
H	3.11279500	3.52365400	-1.01796400
H	1.80723900	2.94251700	-2.07623400
H	4.64412100	-1.00751800	3.51593700
H	3.00678900	-0.84115300	2.81786700
H	2.40514100	-2.09498400	4.72201500
H	3.98622800	-2.92333700	4.80190200
H	1.74467100	-3.39534200	2.76203500
H	2.53534100	-4.63389300	3.78154100
H	3.46448500	-4.26733700	1.25535000
H	4.65877500	-4.35109900	2.57695400
H	3.38056900	-3.69156500	-2.21942300
H	3.51497400	-1.92554100	-2.27652300
H	5.89453100	-2.04936000	-2.84685200
H	5.22297100	-3.39577300	-3.80157000
H	7.22091400	-3.99900700	-2.06506800
H	5.75527100	-5.00285500	-2.01821300
H	6.55788900	-2.76625300	-0.11219500
H	5.77478700	-4.31439700	0.26083900
C	2.23499100	5.10900600	-2.21048100
H	-2.36329400	2.99887400	4.24355500
O	-1.55869100	0.51298600	-1.02905700
P	-2.65916200	0.94114200	-2.00880600
N	-2.30169300	2.18486800	-3.06666000
C	-1.84520700	3.47643800	-2.53328600
C	-1.42955800	4.23152400	-3.78568100
C	-0.78341000	3.13122500	-4.61908200
C	-1.69546800	1.92947100	-4.38911500
H	-2.65208600	3.97394900	-1.97377600
H	-0.98366900	3.34900400	-1.85424000
H	-0.75653900	5.07197200	-3.56305900
H	-2.31955800	4.63470500	-4.29697800
H	0.21891200	2.89458300	-4.22682400
H	-0.68401700	3.38120000	-5.68445100
H	-1.11606900	0.99558700	-4.40590500
H	-2.48798600	1.87239300	-5.15600400
N	-3.89711000	1.57941500	-1.10137400
C	-4.20943200	0.99578500	0.21548200
C	-5.55633100	1.62364100	0.58600900
C	-5.66777400	2.85038000	-0.31783100
C	-5.01501600	2.36836400	-1.60553700
H	-4.27883000	-0.10120000	0.17722400
H	-3.41218100	1.24810500	0.93020600
H	-5.62002000	1.85649400	1.65804600
H	-6.36774100	0.91419300	0.36415700
H	-5.08695400	3.69332000	0.09168000
H	-6.70254800	3.18984500	-0.46722300
H	-4.65930000	3.17915400	-2.25685800
H	-5.72998700	1.75813600	-2.19024700
N	-3.14761700	-0.36525100	-2.91951800
C	-2.22411300	-1.44264800	-3.33131600
C	-2.62803900	-1.71657600	-4.77245500
C	-4.13325100	-1.46483300	-4.76470200
C	-4.29382800	-0.26325000	-3.83145000
H	-1.17454900	-1.13155500	-3.23963100
H	-2.36776100	-2.32611400	-2.69046200
H	-2.36057400	-2.73366500	-5.09154600
H	-2.12274800	-1.00719800	-5.44800300
H	-4.65643600	-2.33651500	-4.34263300
H	-4.55464000	-1.27030800	-5.76113400
H	-5.24588000	-0.29991900	-3.27742500
H	-4.27278800	0.68706300	-4.39501600
O	1.08304200	1.57858800	0.30691500
P	1.05001100	2.99927200	0.87308000
N	2.35567000	3.13338700	1.90848600
C	2.81422600	1.97804000	2.70608900
C	3.30836100	2.61401600	3.99919100
C	3.79531000	3.98750000	3.54582900
C	2.73157600	4.40096200	2.53359000
H	2.00637000	1.24785100	2.86572400
H	3.62410800	1.45076100	2.17912900
H	4.08700500	2.01285900	4.48990100
H	2.47258400	2.72711700	4.70904000
H	4.77243900	3.89575700	3.04306600
H	3.90188200	4.71484100	4.36278400
H	3.11678900	5.11331500	1.78632800
H	1.87260100	4.87905500	3.04211300
N	-0.29327600	3.52559400	1.71071900
O	-0.55435200	-2.45691900	-0.66039100
C	-0.98676800	-3.70988400	-0.70168300
H	-5.97781900	-3.46775500	-1.85195500
C	-0.00114900	-4.69941700	-1.29242600
H	-4.32748600	-3.97245500	-2.27326900
H	-2.41372100	-5.18215400	-0.31063400
C	-4.91388000	-3.31447700	-1.60178100
H	-0.49748600	-5.68410400	-1.31371300
C	0.36751400	-4.30551000	-2.72149900
C	1.24113400	-4.79738000	-0.40899900
H	0.81219400	-3.29830900	-2.74933100
H	1.09391300	-5.01367100	-3.15273000
H	-0.52266400	-4.29568500	-3.37037800
H	0.97458200	-5.12657900	0.60748200
H	1.97133400	-5.51168500	-0.82487100

H	-6.30252500	-4.93733800	0.15020800
H	-3.25303800	-3.27506600	1.43904100
C	-5.23672200	-4.84629500	0.41510400
H	-4.73518200	-5.76419200	0.04418200
C	-2.19595600	-4.11183800	-0.25080800
C	-4.62104200	-3.61770500	-0.16944500
C	-3.26009000	-3.22199900	0.33439700
H	-3.03308300	-2.17640600	0.06778900
H	-5.15159800	-4.84957700	1.51265000
H	-4.63343900	-2.27747400	-1.84582800
H	1.72838600	-3.81451600	-0.32964500
C	-6.12244100	-2.02861600	0.77152500
C	-5.92518900	-0.66812900	5.50591100
C	-5.86956800	-1.61388700	1.90122500
H	-6.65261900	-2.10070400	-0.16229000
H	-3.36644700	-0.89838300	2.59538600
H	-2.63488200	-0.11321300	4.79913000
H	-4.26747700	0.04619900	6.69369500
H	-6.64128800	-0.61474100	6.32876300
H	-7.38541600	-1.39809800	4.09118400
C	-6.34646200	-1.10877300	4.25690500
C	-5.43623900	-1.18549000	3.18563900
C	-4.09585100	-0.81516800	3.40281200
C	-3.68528500	-0.37474500	4.65578100
C	-4.59548600	-0.29576100	5.70989100

TPPA-based reaction **Int III** with phenylacetylene

H_{corr} = 1.5500

$-TS$ = -0.2268

$E_{sol(THF)}$ = -28531.0496

$G_{sol(THF)}$ = -28529.7264

H	0.00640700	0.56065300	4.07354200
H	0.13479200	-0.53208700	5.49095200
C	1.33097300	-2.20972900	3.13467600
C	2.40399000	-1.72382200	4.09727400
H	2.23915100	0.35026000	3.48533900
H	3.41375500	-1.82049000	3.67374000
C	1.98430100	-0.28150000	4.35134500
C	0.46524600	-0.36460000	4.45035700
P	-3.73533700	1.84347500	-0.84504900
O	-2.35892700	1.18007300	-0.94106400
C	-1.46193100	-5.45891900	2.80121900
H	1.20355000	-3.30248800	3.15427000
Sm	-0.33607000	0.15009700	-0.62707900
N	-4.32704100	1.99765400	-2.40813500
C	-4.03361400	0.96475000	-3.40579100
C	-5.32616300	0.14355500	-3.53020000
C	-6.43911200	1.05811700	-2.97288100
C	-5.72210700	2.37146600	-2.62670100
I	0.60184200	2.23357300	1.52075900
I	-1.66666400	-2.19904900	-2.34204900
N	-4.65034700	0.94590800	0.22765800
C	-4.48296500	-0.52177200	0.27157000
C	-5.83690900	-1.02106300	0.76234500
C	-6.35000200	0.13938800	1.60990200
C	-5.93791700	1.35521700	0.78806500
N	-1.57771900	-3.16923900	2.51899200
H	2.36929300	-2.30808300	5.03173600
C	-2.46620000	-3.66137700	1.46559100
H	1.56445200	-1.90966300	2.09924300
H	-1.80345000	-6.32415500	3.38702900
N	-3.83365000	3.39023600	-0.22705100
C	-3.26911900	3.55249700	1.13870400
C	-2.50126500	4.87415400	1.10204300
C	-2.13191200	5.02465100	-0.36880400
C	-3.38610300	4.52220000	-1.06486200
H	-4.20136600	-0.93725300	-0.70773800
H	-3.67258800	-0.78073900	0.96833600
H	-5.75518200	-1.96704900	1.31615700
H	-6.51513600	-1.18990500	-0.09073500
H	-5.83259400	0.15313100	2.58335000
H	-7.43238300	0.10822300	1.79881700
H	-5.83426500	2.26999800	1.38961700
H	-6.69444400	1.55381100	0.00604800
H	-0.68410600	-3.97491100	4.25212300
H	-2.46048000	-4.13012900	4.20281000
H	-3.31331200	-5.66572600	1.64564100
H	-1.85867600	-5.60196600	0.64598200
H	-0.41506400	-5.64072200	2.51419200
C	-1.53914900	-4.15429200	3.58666900
H	-3.51321700	-3.35969300	1.65952600
H	-2.17090500	-3.24321800	0.49283200
H	-4.07152300	3.55156800	1.89379500
H	-2.57458400	2.72921500	1.36354400
H	-1.62446500	4.83936200	1.76297800
H	-3.14965400	5.70714300	1.42017000
H	-1.28052000	4.36723000	-0.60919700
H	-1.86858200	6.05389800	-0.65129000
H	-3.22548500	4.19537800	-2.09937600
H	-4.17480500	5.29548300	-1.06937000
H	-3.79463100	1.47296900	-4.35504000
H	-3.16125800	0.36655800	-3.11455800
H	-5.24307800	-0.78304800	-2.94292200
H	-5.50977300	-0.15467400	-4.57198200
H	-6.88345600	0.61796600	-2.06664300
H	-7.25868900	1.22136300	-3.68765000
H	-6.13158700	2.89870900	-1.75387100
H	-5.76087400	3.07073400	-3.48033000
C	-2.32450500	-5.19385500	1.55386700
H	2.43888300	0.15996400	5.24924000
O	1.43996900	-1.28651600	-0.43387100
P	2.53233600	-2.27197100	-0.86334300
N	2.13142800	-3.89367900	-0.86817600
C	1.57126600	-4.50666500	0.34389000
C	1.14385400	-5.88158500	-0.14485700
C	0.58233300	-5.56424200	-1.52573900
C	1.55167100	-4.52152100	-2.07550100
H	2.32512000	-4.54317900	1.14514900
H	0.70083500	-3.93714400	0.71698200
H	0.41733100	-6.36171200	0.52562300
H	2.02342400	-6.54172000	-0.22398900
H	-0.41553500	-5.10563700	-1.43344800
H	0.49758800	-6.44146700	-2.18188100
H	1.02003200	-3.79579700	-2.70730700
H	2.35582600	-4.98852100	-2.67013800
N	3.75140900	-2.17979400	0.26573200
C	4.14734700	-0.84359100	0.74599900
C	5.64121700	-0.98137100	1.00098100
C	5.78282700	-2.44798300	1.39732300
C	4.83081500	-3.15506900	0.43653200
H	3.91060400	-0.05698600	0.01658900
H	3.59611700	-0.59929900	1.66920800
H	6.00348200	-0.27535500	1.76067800
H	6.19657800	-0.78466800	0.06917200

H	5.44213400	-2.59334500	2.43600400
H	6.81018000	-2.83148300	1.32431300
H	4.44836300	-4.10529600	0.83911100
H	5.34191200	-3.38200100	-0.51537900
N	3.07095900	-1.88934700	-2.38922200
C	2.19283800	-1.31666000	-3.42939500
C	2.67631600	-1.99666800	-4.70171500
C	4.17309900	-2.16186400	-4.45567700
C	4.25216200	-2.53208800	-2.97444100
H	1.13265400	-1.51390800	-3.21652500
H	2.32676800	-0.22543000	-3.47239000
H	2.44657000	-1.40695700	-5.60004600
H	2.19503600	-2.98232900	-4.81193000
H	4.68878600	-1.20433200	-4.62553100
H	4.64717000	-2.91945100	-5.09551900
H	5.17868400	-2.15854900	-2.50916100
H	4.22295800	-3.62851600	-2.83458700
O	-1.19856300	-0.92888000	1.27434500
P	-1.25888500	-1.54081300	2.67329100
N	-2.45826500	-0.74703700	3.52278500
C	-2.70056400	0.69516400	3.32438400
C	-3.05598600	1.18949300	4.71953600
C	-3.74964900	-0.01790100	5.34336300
C	-2.91031100	-1.19094200	4.84289600
H	-1.81956900	1.19851500	2.89735900
H	-3.53430700	0.84290600	2.61905000
H	-3.68278800	2.09220300	4.69800700
H	-2.13788600	1.42957300	5.28031400
H	-4.77816600	-0.10387700	4.95514400
H	-3.80634000	0.01731000	6.44035900
H	-3.49652400	-2.12041600	4.77066800
H	-2.05944800	-1.38439600	5.52257100
N	0.11456400	-1.53647600	3.62252400
O	0.61397400	1.28389700	-2.16274800
C	1.10694300	2.14766000	-3.04097300
H	5.91214600	0.91004700	-3.08252300
C	0.20930500	2.42001300	-4.23299900
H	4.29287200	1.16527300	-3.79351200
H	2.56029600	3.50094000	-3.67601700
C	4.85194500	1.10326700	-2.84792400
H	0.76941400	3.07432500	-4.92216800
C	-0.15098400	1.12992400	-4.96542600
C	-1.04780300	3.15825400	-3.77431900
H	-0.65773200	0.42162500	-4.29142600
H	-0.82292000	1.33363300	-5.81475200
H	0.74950000	0.63245700	-5.35892100
H	-0.78776800	4.12157500	-3.30843800
H	-1.72760600	3.35430500	-4.61997200
H	6.34836300	3.43782600	-3.03020800
H	3.14734500	3.54008000	-1.07824800
C	5.26519300	3.56706200	-2.87356700
H	4.78948600	3.63103500	-3.86438900
C	2.29361000	2.78180800	-2.89645300
C	4.68575300	2.40246300	-2.06104200
C	3.20204400	2.63972800	-1.71937400
H	2.84175300	1.81311800	-1.08762400
H	5.10946500	4.52212200	-2.34883800
H	4.45549400	0.24618900	-2.28247300
H	-1.58962800	2.55685800	-3.03004600
C	5.50559200	2.29913500	-0.78014300
C	5.39830600	5.00372200	3.34234400
C	5.19578500	2.78046500	0.39312800
H	6.48131400	1.79297100	-0.89657600
H	3.91863200	1.53840800	2.39244900
H	3.66201500	2.39871400	4.69452300
H	4.60283300	4.62394500	5.32164300
H	5.80032300	5.98403700	3.60782800
H	6.07411500	5.12752400	1.29905400
C	5.55567400	4.53203200	2.05194000
C	5.04346200	3.25207700	1.67808100
C	4.35413200	2.49618000	2.67266700
C	4.20702100	2.98957400	3.95565000
C	4.72787500	4.24078200	4.30753900

TPPA-based reaction **TS III** with phenylacetylene

H_{corr} = 1.5495

$-TS$ = -0.2242

$E_{sol(THF)}$ = -28531.0425

$G_{sol(THF)}$ = -28529.7172

H	2.15728000	2.11207400	3.48672800
H	2.39169600	3.85123600	3.86049200
C	0.06990700	3.93621000	1.81281700
C	-0.40387300	4.20861800	3.23373300
H	-0.14250600	2.16114900	3.90393000
H	-1.49853500	4.14573400	3.31490700
C	0.33108200	3.14665700	4.04094800
C	1.71188600	3.11285300	3.39850300
P	3.23849000	-2.65319000	-0.56406200
O	1.93029800	-1.86717300	-0.45429500
C	3.28352500	5.63720500	-1.49648400
H	0.03234300	4.83591300	1.18172400
Sm	0.21308300	-0.41826300	-0.05068000
N	3.01778400	-3.85873400	-1.70418100
C	2.28410600	-3.56036900	-2.93604900
C	3.37947700	-3.46539200	-3.99280700
C	4.41486600	-4.51233700	-3.54885400
C	4.10058400	-4.77950600	-2.05833300
I	0.84776300	-0.62522100	3.05948300
I	0.44618300	0.03206300	-3.19202900
N	4.45876700	-1.53442200	-0.83321500
C	4.19440800	-0.28892500	-1.56502000
C	5.55879300	0.42890100	-1.57478700
C	6.54987200	-0.54893400	-0.92107600
C	5.85261500	-1.89962900	-1.03277800
N	2.18884000	3.80378400	-0.49820500
H	-0.09608000	5.21894800	3.55099800
C	2.66591300	3.35457700	-1.81989300
H	-0.54742200	3.15627200	1.33891300
H	4.33912200	5.46656600	-1.22531600
N	3.79598000	-3.49136800	0.76290400
C	4.13904500	-2.67830800	1.95660700
C	3.69776400	-3.52816800	3.14696100
C	2.55663800	-4.36267500	2.57695700
C	3.07010500	-4.70621000	1.18815400
H	3.84370200	-0.48162200	-2.59409400
H	3.40712200	0.28169900	-1.05758600
H	5.51019100	1.37983400	-1.02425000
H	5.85791400	0.66445300	-2.60639500
H	6.68905200	-0.30426200	0.14346400
H	7.54001300	-0.54471800	-1.39858300
H	6.17447400	-2.63026100	-0.27719500
H	6.02645500	-2.34041500	-2.03615800
H	1.40286300	5.76634900	-0.41615800
H	2.79287300	5.49703100	0.65302500

H	3.59601700	4.55412900	-3.39769200
H	1.90906900	4.97697000	-3.03729200
H	3.17528300	6.69303000	-1.78267700
C	2.37700700	5.24924400	-0.33356300
H	3.62758600	2.81839700	-1.72139200
H	1.94860200	2.66248700	-2.28516400
H	5.21662600	-2.45044300	1.97320000
H	3.57883300	-1.73113100	1.94851900
H	3.38638400	-2.89738700	3.99082300
H	4.52032200	-4.18150800	3.48226600
H	1.64946300	-3.74201500	2.49715500
H	2.32236100	-5.25353500	3.17692400
H	2.27593500	-4.95466100	0.47243600
H	3.77598700	-5.55528800	1.22087600
H	1.59915000	-4.40083900	-3.14585000
H	1.68000100	-2.64859900	-2.83305800
H	3.81422400	-2.45317200	-3.96938500
H	3.00345800	-3.63598700	-5.01127000
H	5.44524600	-4.15408800	-3.69083800
H	4.31666800	-5.44190600	-4.12877200
H	4.96165500	-4.63895700	-1.39096200
H	3.74808300	-5.81506200	-1.91490600
C	2.86189900	4.65583300	-2.58670500
H	0.37687100	3.36083600	5.11784900
O	-1.43619600	1.16664300	-0.15748700
P	-2.59587300	2.01767500	-0.69664900
N	-2.26090200	3.26446700	-1.75475300
C	-1.40013700	4.38268900	-1.34043400
C	-1.12676800	5.10794600	-2.64893900
C	-0.98573200	3.94915500	-3.62892500
C	-2.09635200	2.99825300	-3.20034900
H	-1.90504100	5.00888900	-0.59012300
H	-0.45283400	4.01457300	-0.91151500
H	-0.23799300	5.75274600	-2.58897400
H	-1.98759500	5.74090500	-2.92124700
H	-0.01365500	3.44792500	-3.49457500
H	-1.07403100	4.24435900	-4.68357500
H	-1.82622800	1.95113100	-3.39764700
H	-3.03864700	3.22832800	-3.72761400
N	-3.22143200	2.81493200	0.64145100
C	-3.49126600	1.92503400	1.79573600
C	-4.72652900	2.52650000	2.46290200
C	-5.45301000	3.21062500	1.30871200
C	-4.29692200	3.80603000	0.51630500
H	-3.69820200	0.89535000	1.45527000
H	-2.61828700	1.87820600	2.46323600
H	-4.43106800	3.27631400	3.21536600
H	-5.32781800	1.76045800	2.97191800
H	-6.18661000	3.96292300	1.63208300
H	-5.97747400	2.46141200	0.69264400
H	-3.97275100	4.76207700	0.96724900
H	-4.53192400	4.00566300	-0.53825100
N	-3.67999900	1.03098600	-1.46770300
C	-3.37476600	-0.37033900	-1.82440300
C	-4.42739800	-0.71905900	-2.87877300
C	-4.89790200	0.62689000	-3.42445100
C	-4.84638200	1.52604700	-2.19623400
H	-2.35597300	-0.44137900	-2.23324100
H	-3.43844400	-1.02016000	-0.94187600
H	-5.26878200	-1.24852000	-2.41308400
H	-4.01026700	-1.36925700	-3.65958100
H	-5.90233000	0.58250500	-3.86972500

H	-4.19729700	1.00267900	-4.18736000
H	-5.76203100	1.40845300	-1.59069700
H	-4.73529300	2.59283100	-2.44299000
O	1.69364100	1.36453100	0.21861100
P	2.24325400	2.69315100	0.74619800
N	3.81422900	2.40754900	1.23539200
C	4.21899700	1.11222900	1.81702400
C	5.31451200	1.49647800	2.80298100
C	5.93931600	2.73235300	2.16196000
C	4.72207300	3.48332800	1.63133800
H	3.36964100	0.59881000	2.29264500
H	4.60245600	0.44433600	1.02999100
H	6.03107900	0.67998400	2.97121100
H	4.87085000	1.76154100	3.77675000
H	6.58903500	2.43512700	1.32194700
H	6.53781100	3.34039200	2.85470600
H	4.96850100	4.13134500	0.77496800
H	4.28336700	4.12219300	2.42140400
N	1.46273600	3.48330300	1.98973800
O	-1.23466400	-1.99715400	0.12868300
C	-2.01296800	-3.02216800	0.40580100
H	-4.95436300	-3.20720300	5.04422800
C	-1.98352500	-4.14976800	-0.60460600
H	-3.18667600	-3.42558000	5.12210100
H	-3.27320000	-4.05041100	1.72744100
C	-4.03788400	-3.28290700	4.43682500
H	-2.72182200	-4.90208700	-0.27859100
C	-2.35336800	-3.67360200	-2.00633100
C	-0.59464900	-4.79322200	-0.59666200
H	-1.67188700	-2.87422000	-2.33739200
H	-2.28428500	-4.50105300	-2.73057500
H	-3.38161200	-3.28353500	-2.03302300
H	-0.35239000	-5.19481700	0.39998900
H	-0.53555100	-5.61779500	-1.32571200
H	-4.54976800	-0.70375600	5.18074600
H	-1.74010500	-2.44573500	3.31859600
C	-3.67441600	-0.82004800	4.52073200
H	-2.78155800	-0.94245600	5.15410300
C	-2.76316900	-3.10027800	1.54922600
C	-3.83612900	-2.02082900	3.58692800
C	-2.60065900	-2.15057300	2.69210800
H	-2.33856000	-1.15957500	2.29128600
H	-3.54897900	0.11023000	3.94769900
H	-4.12894200	-4.18085900	3.80779600
H	0.17233900	-4.04736300	-0.85514100
C	-5.04333000	-1.84674400	2.69779600
C	-7.10061500	-3.04448300	-1.54850000
C	-5.01246100	-2.03773200	1.39059900
H	-6.00348100	-1.63212600	3.20066900
H	-6.47115500	0.08327500	0.65467600
H	-8.14936100	0.17233200	-1.17888100
H	-8.55464700	-1.83444600	-2.60140600
H	-7.26791100	-3.92620300	-2.17117900
H	-5.60325300	-4.01367300	-0.33060500
C	-6.16800800	-3.09922400	-0.52024300
C	-5.93814300	-1.97918600	0.30815600
C	-6.65430700	-0.79356800	0.03042200
C	-7.59068200	-0.74920200	-0.99660900
C	-7.82276600	-1.87284800	-1.79252800

TPPA-based reaction **Int IV** with phenylacetylene
H_{corr} = 1.5524

$-TS$ = -0.2233
$E_{sol(THF)}$ = -28531.0905
$G_{sol(THF)}$ = -28529.7614

H	1.06093600	1.92167500	3.88270200
H	1.05856800	3.60953700	4.49111700
C	-1.10263400	3.63503600	2.23406700
C	-1.71914400	3.70452100	3.62321000
H	-1.23830700	1.65014000	4.12308700
H	-2.79819800	3.49657000	3.60579000
C	-0.91139500	2.66731700	4.39330200
C	0.50897500	2.87227100	3.87863000
P	3.64159900	-1.50096400	-0.76572000
O	2.16797900	-1.09893300	-0.69129100
C	0.68512000	6.34737600	-0.62867600
H	-1.23792200	4.56498700	1.66162700
Sm	0.05184000	-0.32436200	-0.24717600
N	3.85803300	-2.43339500	-2.14280900
C	3.28238900	-1.94663200	-3.40086500
C	4.47987900	-1.36434800	-4.14438100
C	5.63699700	-2.30113000	-3.76136600
C	5.16559700	-3.00986800	-2.46946300
I	0.23063900	-0.84667000	2.85928400
I	0.28777100	0.89747500	-3.19415200
N	4.54365200	-0.09516500	-0.65679500
C	4.00051700	1.17240200	-1.19573700
C	5.24748500	1.99532100	-1.51462300
C	6.31609300	1.41062800	-0.59501200
C	6.00432600	-0.07810700	-0.65127900
N	1.21140900	4.26498900	0.23150000
H	-1.57247600	4.70900400	4.05415400
C	1.69290200	4.17108400	-1.14613900
H	-1.53754200	2.80756400	1.64951200
H	0.99538600	7.39953900	-0.70043200
N	4.26733300	-2.46127300	0.44423300
C	4.26320900	-1.88729000	1.81034700
C	4.20295500	-3.10517600	2.72201800
C	3.34243500	-4.07039900	1.91804400
C	3.86348500	-3.88499800	0.50043600
H	3.36096200	1.01406200	-2.07744500
H	3.37306000	1.65267100	-0.43379900
H	5.08168100	3.07129500	-1.36303500
H	5.54487800	1.85015600	-2.56627600
H	6.17947100	1.77963300	0.43491700
H	7.34425500	1.64021100	-0.90872800
H	6.39995300	-0.64444400	0.20494200
H	6.43552000	-0.51540000	-1.57284400
H	0.63562300	5.82990600	1.52602800
H	2.26544500	6.01743500	0.82693100
H	2.02857500	5.97642300	-2.32725100
H	0.41626800	5.28565300	-2.53123300
H	-0.41456700	6.32604000	-0.59459800
C	1.23478400	5.66544200	0.62062700
H	2.79847600	4.11901000	-1.17217700
H	1.29899600	3.26461700	-1.62741800
H	5.15918800	-1.26953000	1.97594600
H	3.36904100	-1.26283500	1.96870300
H	3.77420100	-2.85476200	3.70204600
H	5.21198200	-3.52194200	2.87818900
H	2.29101000	-3.75242600	1.97113300
H	3.40550700	-5.11348200	2.25848600
H	3.11242800	-4.11478300	-0.26755400
H	4.74646200	-4.51992100	0.30979500
H	2.85891400	-2.80787200	-3.94543000
H	2.46496900	-1.23423600	-3.22056200
H	4.67104500	-0.34182500	-3.78100000
H	4.31713300	-1.30192900	-5.22949800
H	6.57760900	-1.75118500	-3.60851400
H	5.82555600	-3.04250600	-4.55185400
H	5.85771400	-2.89804700	-1.62358700
H	5.03984300	-4.09149600	-2.64504600
C	1.20564500	5.48147300	-1.79220800
H	-0.97954000	2.77332600	5.48500300
O	-1.91093300	0.84364700	-0.14556800
P	-3.25260800	1.45177200	-0.57073700
N	-3.21629600	2.83388400	-1.50779300
C	-2.50865800	4.03450000	-1.04710400
C	-2.59437900	4.95193600	-2.25686500
C	-2.37851800	3.97656200	-3.40780200
C	-3.16310500	2.73854500	-2.98226300
H	-2.99239000	4.45168500	-0.15090300
H	-1.45581400	3.81333800	-0.79525400
H	-1.85626900	5.76555100	-2.22544200
H	-3.59843700	5.40431700	-2.31251800
H	-1.31201200	3.70932700	-3.48325700
H	-2.70580600	4.36271100	-4.38301700
H	-2.65338900	1.82499300	-3.32004800
H	-4.18855000	2.74682700	-3.39047500
N	-4.02885100	1.95900200	0.81517200
C	-4.02003600	1.04166300	1.97124900
C	-5.34638000	1.32648300	2.66782500
C	-5.63087100	2.78042900	2.30301400
C	-5.19051200	2.84917500	0.84577000
H	-3.92758800	-0.00583200	1.64972100
H	-3.15599800	1.25967000	2.61995200
H	-5.30104600	1.14332400	3.75040300
H	-6.13413400	0.68009100	2.24811900
H	-5.01026600	3.45659800	2.91377800
H	-6.68166500	3.07335500	2.43753000
H	-4.91885100	3.86712500	0.52933300
H	-6.00377000	2.50205800	0.18372300
N	-4.13019900	0.31740700	-1.40150000
C	-3.49916500	-0.72027000	-2.23422700
C	-4.39056500	-0.75670200	-3.46773400
C	-5.78052400	-0.46127700	-2.90879500
C	-5.52863100	0.53840800	-1.77539100
H	-2.45846300	-0.46131800	-2.47227300
H	-3.49180900	-1.68193400	-1.69934200
H	-4.33665300	-1.72134600	-3.99168100
H	-4.08289600	0.02834500	-4.17719900
H	-6.21662300	-1.38011900	-2.49273000
H	-6.47864500	-0.06429300	-3.65947600
H	-6.19998900	0.35141600	-0.92087400
H	-5.68791700	1.57931200	-2.11005600
O	1.05816100	1.68654400	0.47374300
P	1.34349900	2.96101800	1.26542900
N	2.88531100	2.84027000	1.90083300
C	3.41841700	1.54925500	2.37486700
C	4.21931600	1.92939600	3.61169300
C	4.74144700	3.31960100	3.26157400
C	3.55062100	3.96742000	2.55816700
H	2.60997200	0.83171900	2.58206300
H	4.06370600	1.10219900	1.60133800
H	5.01646100	1.20606400	3.83506300
H	3.55623100	1.98358200	4.49053900

H	5.58800100	3.23705500	2.55975700
H	5.08058300	3.90170400	4.12994300
H	3.86646300	4.72615400	1.82505800
H	2.88428900	4.46478000	3.28722700
N	0.32414600	3.40007300	2.51238500
O	-0.81776800	-2.23246700	-0.66283600
C	-1.21315500	-3.45860600	-1.00038800
H	-1.71170300	-6.95620400	3.30178200
C	-0.75045600	-3.99902400	-2.33299900
H	-0.19485100	-7.21820200	2.40589600
H	-1.78784300	-5.38466300	-0.36367400
C	-1.14482800	-6.65819900	2.40500200
H	-1.27775100	-4.95803800	-2.49122700
C	-1.09788300	-3.06738800	-3.48904900
C	0.75751100	-4.28892900	-2.31266700
H	-0.63097800	-2.07912200	-3.35729000
H	-0.74671400	-3.47973400	-4.44882500
H	-2.18596600	-2.91878500	-3.56020400
H	1.00616000	-5.07308500	-1.58082800
H	1.11885400	-4.62425000	-3.29987700
H	-0.51981400	-5.06525800	4.54368100
H	0.43789300	-5.42768400	0.64422800
C	-0.02037400	-4.77662700	3.60369400
H	0.94606200	-5.30603300	3.56841100
C	-1.47865500	-4.43404900	0.11369700
C	-0.87955600	-5.14489200	2.39881500
C	-0.27815100	-4.69863600	1.05355500
H	0.23649300	-3.73490700	1.19338200
H	0.17424700	-3.69485000	3.63423900
H	-1.72670100	-6.96808100	1.52304700
H	1.31001600	-3.38018200	-2.03255000
C	-2.20513200	-4.42177000	2.36026500
C	-5.72966300	-3.43551000	-0.81511600
C	-2.55369100	-4.04451900	1.11950400
H	-2.84736500	-4.33853100	3.24126000
H	-4.13963800	-2.38265100	2.55528000
H	-6.43955600	-1.63908500	1.98265000
H	-7.47041500	-2.31810300	-0.18307700
H	-6.17241400	-3.74388500	-1.76526100
H	-3.87755500	-4.47120700	-1.19086800
C	-4.43982200	-3.84911300	-0.49300900
C	-3.85921000	-3.50129000	0.73696200
C	-4.59454500	-2.68474700	1.60949100
C	-5.87922100	-2.26139100	1.28112100
C	-6.45753600	-2.63989200	0.06926600

TPPA-based reaction **Product** with phenylacetylene
H_{corr} = 1.5532
$-TS$ = -0.2273
$E_{sol(THF)}$ = -28531.0967
$G_{sol(THF)}$ = -28529.7707

H	-0.49242000	-2.12366000	3.75706300
H	0.01442900	-3.63658100	4.57630000
C	2.04144000	-3.38662800	2.22433900
C	2.67361900	-3.08725600	3.57468200
H	1.64444200	-1.18670900	3.79703700
H	3.65762000	-2.60901700	3.46709600
C	1.62348300	-2.19989300	4.23120200
C	0.31185000	-2.86972700	3.83857300
P	-3.84379700	0.68253700	-0.93392300
O	-2.32901900	0.78549100	-0.82184500
C	1.04880500	-6.47676600	-0.60067800
H	2.42462800	-4.31084100	1.76597000
Sm	-0.08072800	0.16158200	-0.15246000
N	-4.31963200	1.63426600	-2.24632700
C	-3.61057500	1.38301400	-3.50701800
C	-4.58777900	0.53495500	-4.31344900
C	-5.95736100	1.12094800	-3.94322100
C	-5.73742500	1.81347400	-2.57717900
I	-0.83742300	0.78495000	2.98923100
I	0.11857100	-0.88655100	-3.30122700
N	-4.27969600	-0.93037200	-1.00131500
C	-3.33162900	-1.96327400	-1.47217800
C	-4.23984700	-3.10810500	-1.91637700
C	-5.51182500	-2.90469800	-1.09803400
C	-5.66088000	-1.38960500	-1.10986700
N	0.02246300	-4.59567400	0.25407000
H	2.80696300	-4.01900600	4.14977900
C	-0.40214800	-4.57733400	-1.14836900
H	2.22125600	-2.55186900	1.52675300
H	1.05149100	-7.57390200	-0.67180800
N	-4.79750600	1.27176000	0.30877500
C	-4.70952100	0.54408000	1.59717400
C	-5.02552900	1.60250100	2.64499800
C	-4.42285400	2.85838200	2.02969600
C	-4.80395700	2.72732100	0.56143000
H	-2.68398200	-1.60140000	-2.28485700
H	-2.66619500	-2.25990300	-0.64918800
H	-3.77228800	-4.09038900	-1.76001100
H	-4.47133100	-3.01856400	-2.99069900
H	-5.36207900	-3.25528600	-0.06350600
H	-6.39164900	-3.41930300	-1.51016500
H	-6.27246700	-1.00171800	-0.28116800
H	-6.13714400	-1.06816700	-2.05690100
H	0.90596900	-5.99903100	1.55792200
H	-0.60968500	-6.55891600	0.79968500
H	-0.42230500	-6.48030300	-2.21737700
H	1.00134200	-5.49480000	-2.56672800
H	2.09553700	-6.13876900	-0.55561400
C	0.31388100	-5.96779300	0.63292400
H	-1.49930400	-4.69021900	-1.23325400
H	-0.13210000	-3.61917000	-1.61470000
H	-5.41493700	-0.30081300	1.61570900
H	-3.69025200	0.15881700	1.76194900
H	-4.58430000	1.34718200	3.61824000
H	-6.11575400	1.71418100	2.76961400
H	-3.32849400	2.81370700	2.13791800
H	-4.78427600	3.79382700	2.48064900
H	-4.10910000	3.24989900	-0.11103000
H	-5.81547800	3.12872000	0.37135500
H	-3.42303000	2.34954400	-4.00824200
H	-2.63733200	0.90415600	-3.32664000
H	-4.50948200	-0.51389500	-3.98559900
H	-4.38563700	0.55876900	-5.39360700
H	-6.73976900	0.34917200	-3.89366800
H	-6.28278100	1.85841500	-4.69239800
H	-6.36873300	1.41288400	-1.77204800
H	-5.95505700	2.89255700	-2.65078800
C	0.31165400	-5.79675600	-1.76611400
H	1.73501900	-2.10655400	5.32049100
O	2.26531200	-0.42370100	0.02827900
P	3.67878700	-0.67952800	-0.48239400
N	3.96554100	-2.00933100	-1.45697300
C	3.57607400	-3.35023500	-1.00210300

C	3.84630100	-4.20651400	-2.22878700
C	3.36423600	-3.29932300	-3.35446200
C	3.83473500	-1.91148600	-2.92694900
H	4.16442100	-3.64953100	-0.12117100
H	2.50667700	-3.39126100	-0.72784100
H	3.32768000	-5.17473000	-2.19115500
H	4.92811300	-4.40094200	-2.31905400
H	2.26290400	-3.29807600	-3.39883700
H	3.74596800	-3.58140800	-4.34551100
H	3.10078300	-1.15049000	-3.23011800
H	4.81553200	-1.66262900	-3.36857800
N	4.66421800	-0.98187900	0.83713000
C	4.55024000	-0.05170800	1.97288100
C	5.97987200	0.07092300	2.48716000
C	6.57859200	-1.29076800	2.14775700
C	6.00125100	-1.57409800	0.76478500
H	4.12527500	0.91128600	1.65352300
H	3.87658200	-0.46577300	2.74132900
H	6.02704100	0.32055300	3.55651800
H	6.51394800	0.85960200	1.93053500
H	6.22206800	-2.04823300	2.86541300
H	7.67756000	-1.30831900	2.15609400
H	5.94785100	-2.65046700	0.54237800
H	6.62685200	-1.10779600	-0.01704900
N	4.20605900	0.65807100	-1.32450400
C	3.27919100	1.55772800	-2.02409300
C	4.02873600	1.89437500	-3.30543100
C	5.48882100	1.92030700	-2.85970100
C	5.57810600	0.81116600	-1.80536600
H	2.31868400	1.06162500	-2.22426100
H	3.08855300	2.45664200	-1.41595400
H	3.69916600	2.84606900	-3.74680800
H	3.86269000	1.10051600	-4.05113900
H	5.71703600	2.89031000	-2.39434700
H	6.20132200	1.76557300	-3.68240500
H	6.26100700	1.09160400	-0.98527800
H	5.95366000	-0.13148800	-2.24211400
O	-0.61069900	-2.09630200	0.50559400
P	-0.50159100	-3.39479400	1.29288700
N	-2.00611500	-3.72328800	1.94955900
C	-2.94630500	-2.63120400	2.25875700
C	-3.56796600	-3.06338300	3.57841600
C	-3.60113000	-4.58340200	3.45338400
C	-2.26996600	-4.90467800	2.77461600
H	-2.42816100	-1.66342200	2.32428100
H	-3.71062000	-2.54834100	1.46643300
H	-4.55822800	-2.61581300	3.74471300
H	-2.91583300	-2.76118000	4.41402900
H	-4.43571200	-4.88726300	2.79992600
H	-3.71721200	-5.10836200	4.41215700
H	-2.33162800	-5.81772500	2.16135400
H	-1.47019800	-5.06023600	3.52135500
N	0.60636800	-3.51715600	2.54174500
O	0.33243600	2.63464200	-0.56580400
C	0.00387000	3.78409300	-0.83071200
H	-0.44272200	7.24983100	3.46889000
C	-0.44328900	4.18561800	-2.21773700
H	-1.99012900	7.05736200	2.61127200
H	-0.02533600	5.83568600	-0.22972100
C	-0.91694100	6.80834700	2.57823700
H	0.04131500	5.15760400	-2.42892400
C	-0.04423000	3.17105900	-3.27325800
C	-1.96057800	4.41983500	-2.19118500
H	-0.47882400	2.18120000	-3.07395300
H	-0.38466400	3.50042300	-4.26645400
H	1.04655100	3.04040300	-3.31304900
H	-2.24781100	5.20741300	-1.47883500
H	-2.30428600	4.73072900	-3.18945300
H	-1.00484600	5.08061400	4.69688600
H	-2.12220300	5.15377500	0.84215400
C	-1.40254100	4.65653000	3.76059700
H	-2.48561000	4.85928400	3.73896400
C	-0.00233400	4.84119300	0.25227500
C	-0.71817300	5.28577700	2.55255800
C	-1.19963000	4.68562900	1.21573900
H	-1.37660200	3.60819700	1.36115000
H	-1.25414200	3.56671000	3.77773300
H	-0.47612200	7.29209000	1.69235200
H	-2.48802100	3.49257800	-1.92005600
C	0.75839000	4.98322300	2.46874200
C	4.34762800	4.95096000	-0.81321200
C	1.17009700	4.74740600	1.21368900
H	1.42140900	5.05007800	3.33467100
H	3.09747100	3.35449000	2.48959300
H	5.48177500	3.13086100	1.82487900
H	6.29044900	4.15906100	-0.29298500
H	4.68917200	5.39501400	-1.75080100
H	2.32563200	5.63795100	-1.07336100
C	3.01461600	5.08201900	-0.43314600
C	2.55425800	4.53996100	0.77601200
C	3.45928100	3.82219000	1.57174000
C	4.79023200	3.68821200	1.18950300
C	5.24342500	4.25891200	0.00041100
